# Supplementary material for: Adoption of HIV pre-exposure prophylaxis among women at high risk of HIV infection in Kenya
Source: PLoS One. 2022 Sep 9;17(9):e0273409. doi: 10.1371/journal.pone.0273409 (PMC9462728; doi:10.1371/journal.pone.0273409)
Supplement: S1 File — (DOCX) [file pone.0273409.s001.docx]

Baseline Visit Questionnaire v7.2

| **No.** | **Question** | **Responses** | |
| --- | --- | --- | --- |
| 1 | Interviewer ID | ___ ___ | |
| 2 | Date of Screening | ___ ___/___ ___ ___/ ___ ___ ___ ___  D D M M M Y Y Y Y | |
| 3 | Enter Cluster ID | ___ ___ ___ | |
| 5 | Enter Name of Cluster |  | |
| 6 | Enter Name of Hotspot or Beach |  | |
| **Verbal Screening**  Read the Verbal Screening document to the participant and obtain consent to screen. | | | |
| 7 | Did the woman give verbal consent to participate in the screening process? | O Yes (skip to SCR1)  O No (answer #8 then skip to END) | |
| 8 | If the woman gave a reason why she did not want to be verbally consented, please indicate.  *Choose all that apply* | O No reason given or disclosed  O Woman did not have time/want to wait  O Woman did not want to answer screening questions  O Woman did not want to take an HIV test  O Woman needed permission from partner  O Woman self-reported HIV positive  O Other: __________________________________________  __________________________________________________ | |
| **Section 1: Screening Questions (SCR)**  Tell the participant you are going to ask her some questions to determine if she will be enrolled in the study. | | | |
| SCR1 | What is your age?  *Interviewer: If participant does not know, ask for best guess, using schooling or other milestones to assist if necessary. Only after unsuccessful probing should you select response “Don’t Know”.* | | ___ ___ years (if less than 18, verify with SCR2)  O Don’t Know  O Refused to Answer |
| SCR2 | In what year were you born?  *Interviewer: Probe if inconsistent with age. If participant guessed age and doesn’t know birth year, use the estimated age to calculate birth year.* | | ___ ___ ___ ___  Should match age given in SCR1;  If verified <18 years by SCR1 and SCR2, then skip to END |
| SCR3 | Where do you live?  *Interviewer: determine if given locations are within the study region. Consult Study Coordinator for clarification if necessary.* | | O Within Study Region  O Outside Study Region (skip to END)  O Migratory (skip to END)  O Don’t Know (skip to END)  O Refused to Answer (skip to END) |
| SCR4 | Have you lived in this area at least 6 out of the last 12 months?  *Interviewer: Use the confines of the study area to determine based on participant’s response.* | | O Yes  O No (skip to END)  O Don’t Know (skip to END)  O Refused to Answer (skip to END) |
| SCR5 | Do you plan to reside in the local area (study region) for the next 24 months or 2 years?  *Interviewer: A response of “Don’t Know” requires probing – only* ***definitive*** *plans to relocate should be used to exclude.* | | O Yes  O No (skip to END)  O Don’t Know  O Refused to Answer (skip to END) |
| SCR6 | In the past 4 weeks, have you had two or more sexual partners?  *Interviewe*r: *Remember to introduce this question in a way that will not offend the participant.* *Only after unsuccessful probing should you select response “Don’t Know”.* | | O Yes  O No (skip to END)  O Don’t Know (skip to END)  O Refused to Answer (skip to END) |
| SCR7 | Do you have your own mobile phone or regular access to someone else’s mobile phone? | | O Yes  O No (skip to END)  O Don’t Know (skip to END)  O Refused to Answer (skip to END) |
| SCR8 | Are you currently enrolled in another HIV prevention study with this or any other organization? | | O Yes (skip to END)  O No  O Don’t Know (skip to END)  O Refused to Answer (skip to END) |
| **Section 2: HIV Rapid Test and DBS Collection**  For participants who are eligible to this point, perform HIV antibody rapid testing and enter results. Collect DBS card. | | | |
| SCR9 | What was the result of the HIV rapid test?  *Interviewer: If the HIV result is positive or indeterminate, ensure client receives any necessary confirmatory testing, proper counseling, and linkage to care.* | | O Negative (skip to SCR11)  O Positive (skip to SCR10)  O Indeterminate (skip to SCR10)  O Woman refused testing (skip to END) |
| SCR10 | Did you provide appropriate confirmatory testing, counseling, and linkage to care? | | O Yes  O No |
| **Section 3: Main Study Consent**  For eligible participants, read and obtain informed consent for the main study. | | | |
| SCR11 | After reviewing the informed consent document and asking questions, did the participant consent to participate in the main study? | | O Yes  O No (skip to END) |
| **END** | *Interviewer:* Thank you for your time and willingness to participate in the screening process for our study. At this time, however, you will not be enrolled as a participant in this study. This does not mean that you cannot participate in other research studies that are happening now or in the future. If you have any questions, I will be happy to answer them now. | | |

| **Section 4: Participant Identification** | | | | |
| --- | --- | --- | --- | --- |
| PID_GEN | Does a new PID need to be generated for this participant?  Note: Only select “Yes” if this is the first time this form is being completed for this participant. If there was a tablet error and you are re-entering data from another tablet, select “No” and enter the PID that was generated initially. | | | O Yes (skip to DEM01, ODK to generate and display ID)  O No |
| pid | Enter the participant’s ID number. Be extremely careful when entering the number. Double and triple check that the number is correct before proceeding. | | | ___ ___ ___ ___ ___ ___ ___ ___ ___ |
| pid_verify | Verify PID number | | | ___ ___ ___ ___ ___ ___ ___ ___ ___ |
| **Section 5: Baseline Questionnaire**  Screening form now complete. The Baseline survey starts here. | | | | |
| **Section B: Demographic Information (DEM)** | | | | |
| **No.** | | **Question** | **Coding** | |
| DEM01 | | What is your age? | ___ ___  Verify with SCR1 | |
| DEM02 | | What is the highest level of school you have *completed*?  Interviewer: if DEM02=98, probe | O None  O Some Primary  O Primary  O Some Secondary  O Secondary/ High School  O Post-Secondary/Training College  O University  O Don’t Know  O Refused to Answer | |
| DEM03 | | What is your current marital status? | O Married, Living Together  O Married, NOT Living Together  O Not Married, Living Together  O Relationship but Not Married, NOT Living Together (boyfriend, etc.)  O Single  O Divorced  O Widowed  O Don’t Know  O Refused to Answer | |
| DEM04 | | What is your ethnic group? | O Luo  O Luhya  O Kalenjin  O Kikuyu  O Kisii  O Ugandan  O Other: _______________________  O Don’t Know  O Refused to Answer | |
| DEM05 | | What is your **primary** source of income? | O Professional/salaried  O Rental income (landlord, rent equipment)  O Sales and Service (Non-Fish)  O Skilled Manual  O Unskilled Manual  O Domestic Service  O Agriculture  O Fishing/ Fish Trade  O Sex Work  O Informal/Seasonal/ Piece Work  O Student  O Unemployed  O Other: ________________________________  _______________________________________  O Don’t Know  O Refused to Answer | |
| DEM06 | | What is/are your other source(s) of income, if any?  Choose all that apply | O Professional/salaried  O Rental income (landlord, rent equipment)  O Sales and Service (Non-Fish)  O Skilled Manual  O Unskilled Manual  O Domestic Service  O Agriculture  O Fishing/ Fish Trade  O Sex Work  O Informal/Seasonal/ Piece Work  O Student  O Other: _______________________________  ______________________________________  O None/ Not Applicable  O Don’t Know  O Refused to Answer | |
| DEM07 | | How much income do you typically earn in one month, from all sources?  *Interviewer: Ask participant to give best estimate if not sure* | KES ___________________  O Don’t Know  O Refused to Answer | |
| DEM08 | | How many people live in your household, including yourself? | _______  O Refused to Answer | |
| DEM09 | | How many people in your household have a regular or steady income, including yourself? | _______  O Don’t Know  O Refused to Answer  Must be less than or equal to DEM08. | |
| DEM10 | | How many people do you currently support (provide with food, housing, money, etc.)?  *Interviewer: This does not have to be a member of your household.* | _______  O Don’t Know  O Refused to Answer | |
| DEM11 | | In the past month, have you regularly eaten at least two meals a day? | O Yes  O No  O Don’t Know  O Refused to Answer | |

| **Section C: Health and Sexual Behavior** | | |
| --- | --- | --- |
| **Section C1 – General Health (HLT)**  Interviewer: I have some questions about your physical and mental health. | | |
| **No.** | **Question** | **Coding** |
| HLT01 | How would you rate your overall health? | O Very Good  O Good  O Fair  O Poor  O Don’t Know  O Refused to Answer |
| HLT02 | In the past 12 months, what is your best estimate of how often you drank alcohol? | O Never (skip to HLT04)  O Less than once per month  O Once a month  O 2 to 3 times per month  O Once per week  O Twice per week  O 3 to 4 times a week  O 5 to 6 times per week  O Every Day  O Don’t Know  O Refused to Answer |
| HLT03 | In the past month, how often did you drink 5 or more alcoholic drinks in one night? | O Never  O Only once  O 2 or 3 times  O Once per week  O Twice per week  O 3 to 4 times a week  O 5 to 6 times per week  O Every Day  O Don’t Know  O Refused to Answer |

| HLT04:  Over the last 2 weeks, have you been bothered by any of the following problems?  *Interviewer: Read each option aloud one at a time.* | HLT 05:  Over the last 2 weeks, how often have you been bothered by this problem? |
| --- | --- |
| O Had little interest or pleasure in doing things | O One Day  O Less than Half the Days  O Around Half the Days  O More than Half the Days  O Every Day  O Don’t Know  O Refused to Answer |
| O Felt down, depressed, or hopeless | O One Day  O Less than Half the Days  O Around Half the Days  O More than Half the Days  O Every Day  O Don’t Know  O Refused to Answer |
| O Had trouble falling asleep/ staying asleep, or sleeping too much | O One Day  O Less than Half the Days  O Around Half the Days  O More than Half the Days  O Every Day  O Don’t Know  O Refused to Answer |
| O Felt tired or having little energy | O One Day  O Less than Half the Days  O Around Half the Days  O More than Half the Days  O Every Day  O Don’t Know  O Refused to Answer |
| O Had poor appetite or overeating | O One Day  O Less than Half the Days  O Around Half the Days  O More than Half the Days  O Every Day  O Don’t Know  O Refused to Answer |
| O Felt bad about yourself, felt that you are a failure, or felt that you let yourself or your family down | O One Day  O Less than Half the Days  O Around Half the Days  O More than Half the Days  O Every Day  O Don’t Know  O Refused to Answer |
| O Had trouble concentrating on things such as reading the newspaper or watching television | O One Day  O Less than Half the Days  O Around Half the Days  O More than Half the Days  O Every Day  O Don’t Know  O Refused to Answer |
| O Moved or spoken so slowly that other people could have noticed, or been so fidgety/restless that you have moved around a lot more than usual | O One Day  O Less than Half the Days  O Around Half the Days  O More than Half the Days  O Every Day  O Don’t Know  O Refused to Answer |
| O Had thoughts that you would be better off dead, or of hurting yourself in some way | O One Day  O Less than Half the Days  O Around Half the Days  O More than Half the Days  O Every Day  O Don’t Know  O Refused to Answer |
| O None (skip to GSA01) | Not Applicable |
| O Refused to Answer (skip to GSA01) | Not Applicable |

| HLT06 | | How difficult have these problems made it for you to do your work, take care of things at home, or get along with other people? | | O Not Difficult at All  O Somewhat Difficult  O Very Difficult  O Don’t Know  O Refused to Answer |
| --- | --- | --- | --- | --- |
| **Section C2: General Sexual Activity (GSA)**  Interviewer: Now I have some questions about your sexual activity, in order to get a better understanding of some important aspects of your life. For these questions, “sexual activity” is defined as sexual penetration of the vagina or anus. | | | | |
| GSA01 | | How old were you when you had sex for the first time?  *Interviewer: If participant doesn’t know exact age, probe for an estimate based on schooling or other life events.* | | ___ ___ years  O Don’t Know or Cannot Estimate  O Refused to Answer |
| GSA02 | | The first time you had sex, did your partner force or coerce you into any sex act against your will? | | O Yes  O No  O Don’t Know  O Refused to Answer |
| GSA03 | | During the past month, how many different sexual partners have you had? | | ___ ___  O Don’t Know  O Refused to Answer  *Interviewer: Probe if less than 2 – this is part of eligibility* |
| GSA04 | | The last time you had sex, did you use a condom? | | O Yes  O No  O Don’t Know  O Refused to Answer |
| GSA05 | | During the past month, how often did you use a contraceptive device, other than the male condom, to prevent pregnancy? | | O Never (skip to PRP01)  O Less than Half the Time  O About Half the Time  O More than Half the Time  O Don’t Know  O Refused to Answer |
| GSA06 | | In the past month, which of the following contraceptives, other than the male condom, have you used to prevent pregnancy? | | O Pill  O Hormone injection (Depo-Provera)  O Intrauterine device (IUD)  O Diaphragm  O Patch  O Implant (Norplant, Jadelle)  O Emergency pill (plan B or morning after pill)  O Spermicide  O Female condom  O Withdrawal  O Other: ____________________________________  O Don’t Know  O Refused to Answer |
| **Section C3: Primary Partner (PRP)**  Interviewer: Now I have some questions about your **one** primary or regular sexual partner. A primary partner could be your husband, boyfriend, or someone you **regularly** have sex with. A primary partner is the ONE partner you feel like you love or like the most, or the one you can confide in the most. If you would like help deciding who your primary partner is, we can talk about that now. | | | | |
| PRP01 | | Do you currently have a primary partner, or have you had one in the past month?  This is the man we will discuss in the following questions. | | O Yes  O No (skip to NPP01)  O Refused to Answer |
| PRP02 | | In the past month, how often did you use condoms with your primary partner? | | O Never  O Less than Half the Time  O About Half the Time  O More than Half of the Time  O Always (skip to PRP05)  O Don’t Know  O Refused to Answer |
| PRP03 | | Why did you not use condoms all the time with your primary partner within the last month?  *Interviewer choose all that apply based on what the participant mentioned.* | | O I Did Not Want To  O Partner Did Not Want To  O No Condom was Available  O I Am Trying To Become Pregnant  O I Have Another Form of Contraception (IUD, Implant, Injectable, Pill, etc.)  O I Knew My Primary Partner’s HIV Status and decided a condom was not necessary  O I trust my primary partner  O Other: ____________________________________  ___________________________________________  O Don’t Know  O Refused to Answer |
| PRP04 | | In the past month, have you ever had difficulty *negotiating* condom use with your primary partner? | | O Yes, always  O Yes, sometimes  O I have no difficulty negotiating  O I never try to get my partner to use condoms  O Don’t Know  O Refused to Answer |
| PRP05 | | In the past 12 months, has your primary partner gone for an HIV test? | | O Yes  O No  O Don’t Know  O Refused to Answer |
| PRP06 | | Do you know your current primary partner’s HIV status? | | O No  O Yes, Partner is Positive  O Yes, Partner is Negative  O Yes, Refuse to Disclose Partner’s Status  O Refused to Answer |
| PRP07 | | About how old is your current primary partner? (participant to give best guess) | | ___ ___ years  O Don’t Know  O Refused to Answer |
| PRP08 | | How long have you had a sexual relationship with your current primary partner?  Interviewer: For relationships <1 month, record 01 months. (Ex: If participant says one week, record 01months) | | ___ ___ years, ___ ___ months  O Don’t Know  O Refused to Answer |
| **Section C4: Non-Primary Partners (NPP)**  Interviewer: Now I have some questions about any other sexual partners you may have had other than your primary partner. For these questions, I am referring to boyfriends, casual sexual partners, or someone with whom you engage in transactional sex, i.e. sex for money, goods gifts, food or housing. | | | | |
| NPP01 | A: How many (FSW ONLY: clients or other) non-primary sexual partners have you had in the past month?  *Interviewer: probe for an estimate if participant cannot immediately recall* | | | ___ ___ (If 00, skip to TRX01)  O Don’t Know  O Refused to Answer |
| NPP02 | Of these, approximately how many did you have **multiple** sexual encounters with in the past month? | | | ___ ___ (If 00, skip to NPP05)  O Don’t Know  O Refused to Answer |
| NPP03 | Among those with whom you had multiple sexual encounters in the past month how often did you use condoms? | | | O Never  O Less than Half the Time  O About Half the Time  O More than Half of the Time  O Always (skip to NPP05)  O Don’t Know  O Refused to Answer |
| NPP04 | Why did you not always use condoms with those you shared multiple sexual encounters?  *Open ended for participants. Interviewer choose all that apply based on what the participant mentioned.* | | | O I Did Not Want To  O Partner(s) Did Not Want To  O No Condom Was Available  O I Am Trying to Become Pregnant  O I Have Another Form of Contraception (IUD, Implant, Injectable, Pill, etc.)  O I Knew My Non-Primary Partner’s HIV Status  O I trust my non-primary partner(s)  O Partner offered me more money to not use a condom  O Other: _________________________  _________________________________  O Don’t Know  O Refused to Answer |
| NPP05 | A: Approximately how many (FSW only: clients or other) non-primary sexual partners in the past month did you have sex with **only once**? | | | ___ ___ (If 00, skip to TRX01)  O Don’t Know  O Refused to Answer |
| NPP06 | Among the men with whom you had only one sexual encounter in the past month, how often did you use condoms? | | | O Never  O Less than Half the Time  O About Half the Time  O More than Half of the Time  O Always (skip to TRX01)  O Don’t Know  O Refused to Answer |
| NPP07 | Why did you not always use condoms with those you had only one sexual encounter?  *Open ended for participants. Interviewer choose all that apply based on what participant mentioned.* | | | 1: I Did Not Want To  2: Partner(s) Did Not Want To  3. No Condom Was Available  4: I Am Trying To Become Pregnant  5: I Have Another Form of Contraception (IUD, Implant, Injectable, Pill, etc.)  6: I Knew My Non-Primary Partner/Partners’ HIV Status  7: I trust my non-primary partner(s)  8: Partner(s) offered me more money to not use a condom  9: Other (specify): _________  98: Don’t Know  99: Refused to Answer |
| **Section C5: Transactional Sex (TRX)**  Interviewer: I would now like to ask questions about any sexual partners you may have had who paid you for sex or gave you something in exchange for sex. These may be men with whom you had only one sexual encounter, or men with whom you had multiple sexual encounters. Some of these questions may sound similar to ones I just asked, but please think only about those encounters where you exchanged sex. | | | | |
| TRX01 | | Have you ever exchanged sex for money, goods, gifts, food, housing, services, or influence? | | O Yes  O No  O Refused to Answer |
| TRX02 | | How old were you when you **first** exchanged sex for money, gifts, goods, food, housing or services?  *Interviewer: If participant doesn’t know, probe using life events such as schooling.* | | ___ ___ years *(Must be greater than or equal to GSA01)*  O Don’t Know  O Refused to Answer  *Must be greater than or equal to GSA01* |
| TRX03 | | In the past 12 months, have you **regularly** **or repeatedly** exchanged sex for money, goods, food, housing or services? | | O Yes  O No  O Refused to Answer |
| TRX04 | | In the past month, with approximately how many men did you exchange sex? | | _________ (If 0, skip to TRX06)  O Don’t Know  O Refused to Answer |
| TRX05 | | Of these men in the past month with whom you exchanged sex, with how many did you have **multiple** sexual encounters? | | _________ *(Must be less than or equal to TRX04)*  O Don’t Know  O Refused to Answer |
| TRX06 | | In the last 3 months, have you exchanged sex for money, goods, services, or influence in a location other than ____? (*Interviewer: insert name of cluster area*)? | | O Yes  O No (skip to TRX08)  O Refused to Answer (skip to TRX08) |
| TRX07 | | Please list the names of the other places or areas where you have exchanged sex for money, goods, services, or influence in the last 3 months.  *Interviewer: Use the cluster code for each area. See list for codes.* | | 1: ___ ___ ___  2: ___ ___ ___  3: ___ ___ ___  4: ___ ___ ___  5: ___ ___ ___ |
| TRX08 | | In the past month, have you exchanged sex for **money**? | | O Yes  O No (skip to TRX13)  O Refused to Answer |
| TRX09 | | *Ask only if client reported sex work as primary OR secondary income (DEM05 OR DEM06)*  During the past month, did you have to pay someone like a manager, administrator or pimp, or share with someone a percentage of the money that you received for sex? | | O Yes  O No  O Refused to Answer |
| TRX10 | | In a typical month, how much of your income comes from exchanging sex for money? | | O None  O Less than half  O About half  O More than half, but not all  O All  O Don’t Know  O Refused to Answer |
| TRX11 | | When you exchange sex for money, what is the average amount of money you charge per sexual encounter when a condom **is** used? | | KES _________  O I Never Use a Condom  O Don’t Know  O Refused to Answer |
| TRX12 | | When you exchange sex for money, what is the average amount of money per sexual encounter when a condom **is** **not** used? | | KES _________  O I Always Use a Condom  O Don’t Know  O Refused to Answer |
| TRX13 | | In the past month, have you exchanged sex for goods or services **other than** money? | | O Yes  O No (skip to TRX16)  O Refused to Answer (skip to TRX16) |
| TRX14 | | In the past month, what goods or services **other than** money have you exchanged for sex?  *Choose all that apply.* | | O Housing and/or utilities  O Food to eat  O Food to sell (example, fish)  O School fees  O To get a job, a work promotion, or to keep your job  O Other material goods (clothes, jewelry, makeup, electronics, etc.)  O Household items (soap, cleaning supplies, tools, etc.)  O Other (specify): __________  O Don’t Know  O Refused to Answer |
| TRX15 | | *Ask only if TRX08 and/or TRX13 = Yes*  In the past month, what is the approximate **total** *value* of the money, goods, or services you received in exchange for sex?  *Interviewer: Make sure that participant understands to include how much the non-monetary goods/services are worth.* | | KES _______ (6 digits)  O Don’t Know  O Refused to Answer |
| TRX16 | | Have you ever withheld sex in order to get something that you wanted? | | O Yes  O No (skip to SMH01)  O Don’t Know (skip to SMH01)  O Refused to Answer (skip to SMH01) |
| TRX17 | | Did you withhold sex in order to get something from a primary partner or a non-primary partner? | | O Primary Partner  O Non-Primary Partner  O Both  O Don’t Know  O Refused to Answer |
| **Section C6: Social and Mental Health (SMH)**  Interviewer: Now I would like to ask you questions about how your sexual activity has impacted how you feel about yourself, or impacted your relationships with others in your family or community. | | | | |
| SMH01 | | Which individuals have you told about your sexual activities or that you have multiple partners?  *Choose all that apply.* | O No one  O A family member  O A friend  O A healthcare provider  O A community member: specify ____  O My primary partner  O Other: specify _____  O Refused to Answer | |
| SMH02 | | In the past 12 months, have any of the following **actually** happened to you as a direct result of your sexual activity?  *Interviewer: Read each option aloud one at a time to the participant and choose all that apply before moving on to the next question.* | O Felt ashamed or thought less of myself  O Lost respect or standing in your family or community  O Been refused entry to a community event or service (such as a clinic, church, festival, meeting)  O Been physically threatened or hurt by someone in your family or community  O Been verbally insulted, harassed, and/or threatened by someone in your family or community  O Been insulted, discriminated against, or treated poorly by a health care worker  O Been denied health services  O None of the above  O Refused to Answer | |
| SMH03 | | In the past 12 months, have you been **afraid** of any of the following **possibly** happening to you as a direct result of your sexual activity?  *Interviewer: Read each option aloud one at a time to the participant and choose all that apply before moving on to the next question.* | O Been afraid to lose respect or standing in your family or community  O Been afraid to attend a community event or service (such as a clinic, church, festival, meeting)  O Been afraid of being physically abused by someone in your family or community  O Been afraid of being verbally insulted, harassed, and/or threatened by someone in your family or community  O Been afraid of being treated poorly by a health care worker or being denied health care services  O Been afraid of people assuming that I have HIV  O None of the above  O Refused to answer | |

| Interviewer to Read: For this section, I will read a statement to you. Please describe how you feel about the statement. | | |
| --- | --- | --- |
| SMH04 | Most men in this community verbally insult, harass, or threaten women who exchange sex for money, goods, gifts, food, housing, or influence. | O Strongly Disagree  O Disagree  O Neither Agree nor Disagree  O Agree  O Strongly Agree  O Don’t Know  O Refused to Answer |
| SMH05 | Most men in this community physically insult, harass, or threaten women who exchange sex for money, goods, gifts, food, housing, or influence. | O Strongly Disagree  O Disagree  O Neither Agree nor Disagree  O Agree  O Strongly Agree  O Don’t Know  O Refused to Answer |
| SMH06 | Most men in this community would not marry a woman who has exchanged sex for money, goods, gifts, food, housing, or influence. | O Strongly Disagree  O Disagree  O Neither Agree nor Disagree  O Agree  O Strongly Agree  O Don’t Know  O Refused to Answer |
| SMH07 | Most people in this community do not respect women who exchange sex for money, goods, gifts, food, housing, or influence. | O Strongly Disagree  O Disagree  O Neither Agree nor Disagree  O Agree  O Strongly Agree  O Don’t Know  O Refused to Answer |
| SMH08 | Most healthcare providers in this community insult, discriminate, or treat poorly women who exchange sex for money, goods, gifts, food, housing, or influence. | O Strongly Disagree  O Disagree  O Neither Agree nor Disagree  O Agree  O Strongly Agree  O Don’t Know  O Refused to Answer |
| SMH09 | Most healthcare providers in this community refuse services to women who exchange sex for money, goods, gifts, food, housing, or influence. | O Strongly Disagree  O Disagree  O Neither Agree nor Disagree  O Agree  O Strongly Agree  O Don’t Know  O Refused to Answer |

| **Section D: Recent Transactional Encounters (RT#)** | | |
| --- | --- | --- |
| Interviewer: Now, I will be asking you a series of questions related to your recent sexual partners from whom you received money or something else in exchange for sex. I have asked some similar questions but now I will ask for some more details. I greatly appreciate your patience through the next series of questions. I want you to think about the most recent time you exchanged sex for money, goods, gift, food, or housing. I am going to ask you several questions about this exchange. | | |
| **No.** | **Question** | **Coding** |
| RT1: Most Recent Exchange | | |
| RT1 Q1 | Would you like to tell me about the most recent time you exchanged sex? | O Yes  O No (skip to RT2 Q1) |
| RT1 Q2 | When did this sex exchange for money, goods, or gifts occur? | O I Know the Date  O Don’t Know (skip to RT1 Q4)  O Refused to Answer (skip to RT1 Q4) |
| RT1 Q3 | What was the date? | ___ ___/___ ___ ___/ ___ ___ ___ ___  D D M M M Y Y Y Y |
| RT1 Q4 | At what time of day did this encounter take place? | O Early Morning (00:01-08:00)  O Morning (8:01-12:00)  O Afternoon/Evening (12:01-18:00)  O Night (18:01-24:00) O Don’t Know  O Refused to Answer |
| RT1 Q5 | Where did you exchange sex with this person? | O Street, car, or outside  O Bar or nightclub  O Hotel room paid by sex worker  O Hotel room paid by partner  O Brothel  O Woman’s home  O Man’s home  O Other: _______________________________  O Don't Know  O Refused to Answer |
| RT1 Q6 | Please tell me which of the following activities you did during this encounter:  *Interviewer: Read the list of responses aloud to the participant and choose all that apply. Explain any of the choices that the participant does not understand.* | O Kissing  O Danced or stripped for partner  O Massage  O Performed oral sex **with** a condom  O Performed oral sex **without** a condom  O Vaginal sex **with** a condom  O Vaginal sex **without** a condom  O Anal sex **with** a condom  O Anal sex **without** a condom  O Received oral sex  O Talking/company  O Other: _______________________________  _______________________________________ |
| RT1 Q7 | Was this the first time you have exchanged sex with this person? | O Yes  O No  O Don’t Know O Refused to Answer |
| RT1 Q8 | How handsome was this person?  *Interviewer: Remember to probe for this question to determine what the participant thinks is handsome (dressed nicely, physical features, humor, etc.).* | O Not very handsome  O About average  O Handsome  O Don’t Know  O Refused to Answer |
| RT1 Q9 | Approximately how old was this person? Please estimate. | ___ ___ years  O Don’t know  O Refused to answer |
| RT1 Q10 | How wealthy was this person? | O Poor  O Average wealth  O Above-average wealth  O Very wealthy  O Don’t know  O Refused to answer |
| RT1 Q11 | Does this person live in this County, or was he visiting? | O Lives in this County  O Just visiting  O Don’t know  O Refused to answer |
| RT1 Q12 | Did this person take alcohol or drugs around the time you had sex? | O Yes  O No  O Don’t know  O Refused to answer |
| RT1 Q13 | Did YOU take alcohol or drugs around the time you had sex with this person? | O Yes  O No  O Don’t know  O Refused to answer |
| RT1 Q14 | How likely is it that this person had a sexually transmitted infection besides HIV? | O Almost impossible  O A little likely  O Somewhat likely  O Very likely  O Almost certain  O Don't Know  O Refused to Answer |
| RT1 Q15 | How likely is it that this person had HIV? | O Almost impossible  O A little likely  O Somewhat likely  O Very likely  O Almost certain  O Don't Know  O Refused to Answer |
| RT1 Q16 | What did you receive in exchange for having sex with this person?  *Interviewer: Check all that apply.* | O Housing and/or utilities  O Food to eat  O Food to sell (example, fish)  O School fees  O To get a job, a work promotion, or to keep your job  O Other material goods (clothes, jewelry, makeup, electronics, etc.)  O Household items (soap, cleaning supplies, tools, etc.)  O Other: _______________________________  O Don’t Know  O Refused to Answer |
| RT1 Q17 | What was the **total value** of money, goods and gifts that the person gave you for this encounter? | KES_________  O Don't Know  O Refused to Answer |
| Interviewer READ: I will now ask you some questions about things this same person may have done to you during this encounter. These questions are about violence, which may make you feel uncomfortable or distressed. Please remember that your answers are completely confidential and no one will be able to associate this information with you or your sexual partners. You do not have to answer any question that makes you uncomfortable. Please take your time and if you are unclear about any question, just ask me. Are you ready to continue? | | |
| RT1 Q18 | Did this partner threaten you with physical assault? | O Yes  O No  O Don’t know  O Refused to answer |
| RT1 Q19 | Did this partner hit, kick, strangle or otherwise physically assault you? | O Yes  O No  O Don’t know  O Refused to answer |
| RT1 Q20 | Did this partner force or coerce you to participate in any sex act against your will? | O Yes, to have sex with a condom  O Yes, to have sex without a condom  O Yes, to have anal sex  O Yes, Other____________________________  O No  O Don’t know  O Refused to answer |
| Interviewer: Now, I will be asking you the same questions as I just did, for your second most recent encounter where you exchanged sex for something. This could be with a different partner as the one we just discussed, or the same. | | |
| RT2: Second Most Recent Exchange | | |
| **No.** | **Question** | **Coding** |
| RT2 Q1 | Would you like to tell me about the second most recent time you exchanged sex? | O Yes  O No (skip to RT3 Q1) |
| RT2 Q2 | Is this the same person we just spoke of for the last time you exchanged sex? | O Yes *(Do not ask RT2 Q8 thru Q13 or Q15 thru Q16)*  O No |
| RT2 Q3 | When did this sex exchange for money, goods, or gifts occur? | O I Know the Date  O Don’t Know (skip to RT2 Q5)  O Refused to Answer (skip to RT2 Q5) |
| RT2 Q4 | What was the date? | ___ ___/___ ___ ___/ ___ ___ ___ ___  D D M M M Y Y Y Y |
| RT2 Q5 | At what time of day did this encounter take place? | O Early Morning (00:01-08:00)  O Morning (8:01-12:00)  O Afternoon/Evening (12:01-18:00)  O Night (18:01-24:00) O Don’t Know  O Refused to Answer |
| RT2 Q6 | Where did you exchange sex with this person? | O Street, car, or outside  O Bar or nightclub  O Hotel room paid by sex worker  O Hotel room paid by partner  O Brothel  O Woman’s home  O Man’s home  O Other: _______________________________  O Don't Know  O Refused to Answer |
| RT2 Q7 | Please tell me which of the following activities you did during this encounter:  *Interviewer: Read the list of responses aloud to the participant and choose all that apply. Explain any of the choices that the participant does not understand.* | O Kissing  O Danced or stripped for partner  O Massage  O Performed oral sex **with** a condom  O Performed oral sex **without** a condom  O Vaginal sex **with** a condom  O Vaginal sex **without** a condom  O Anal sex **with** a condom  O Anal sex **without** a condom  O Received oral sex  O Talking/company  O Other: _______________________________  _______________________________________ |
| RT2 Q8 | *Do not ask if RT2 Q2 = Yes*  Was this the first time you have exchanged sex with this person? | O Yes  O No  O Don’t Know O Refused to Answer |
| RT2 Q9 | *Do not ask if RT2 Q2 = Yes*  How handsome was this person?  *Interviewer: Remember to probe for this question to determine what the participant thinks is handsome (dressed nicely, physical features, humor, etc.).* | O Not very handsome  O About average  O Handsome  O Don’t Know  O Refused to Answer |
| RT2 Q10 | *Do not ask if RT2 Q2 = Yes*  Approximately how old was this person? Please estimate. | ___ ___ years  O Don’t know  O Refused to answer |
| RT2 Q11 | *Do not ask if RT2 Q2 = Yes*  How wealthy was this person? | O Poor  O Average wealth  O Above-average wealth  O Very wealthy  O Don’t know  O Refused to answer |
| RT2 Q12 | *Do not ask if RT2 Q2 = Yes*  Does this person live in this County, or was he visiting? | O Lives in this County  O Just visiting  O Don’t know  O Refused to answer |
| RT2 Q13 | Did this person take alcohol or drugs around the time you had sex? | O Yes  O No  O Don’t know  O Refused to answer |
| RT2 Q14 | Did **you** take alcohol or drugs around the time you had sex with this person? | O Yes  O No  O Don’t know  O Refused to answer |
| RT2 Q15 | *Do not ask if RT2 Q2 = Yes*  How likely is it that this person had a sexually transmitted infection besides HIV? | O Almost impossible  O A little likely  O Somewhat likely  O Very likely  O Almost certain  O Don't Know  O Refused to Answer |
| RT2 Q16 | *Do not ask if RT2 Q2 = Yes*  How likely is it that this person had HIV? | O Almost impossible  O A little likely  O Somewhat likely  O Very likely  O Almost certain  O Don't Know  O Refused to Answer |
| RT2 Q17 | What did you receive in exchange for having sex with this person?  *Interviewer: Check all that apply.* | O Housing and/or utilities  O Food to eat  O Food to sell (example, fish)  O School fees  O To get a job, a work promotion, or to keep your job  O Other material goods (clothes, jewelry, makeup, electronics, etc.)  O Household items (soap, cleaning supplies, tools, etc.)  O Other: _______________________________  O Don’t Know  O Refused to Answer |
| RT2 Q18 | What was the **total value** of money, goods and gifts that the person gave you for this encounter? | KES_________  O Don't Know  O Refused to Answer |
| RT2 Q19 | Did this partner threaten you with physical assault? | O Yes  O No  O Don’t know  O Refused to answer |
| RT2 Q20 | Did this partner hit, kick, strangle or otherwise physically assault you? | O Yes  O No  O Don’t know  O Refused to answer |
| RT2 Q21 | Did this partner force or coerce you to participate in any sex act against your will? | O Yes, to have sex with a condom  O Yes, to have sex without a condom  O Yes, to have anal sex  O Yes, Other__________________________  O No  O Don’t know  O Refused to answer |
| Interviewer: For the last time, I will be asking you the same questions as I just did about your third most recent encounter where you exchanged sex for something. This could be with a different partner as the one we just discussed, or the same. | | |
| RT3: Third Most Recent Exchange | | |
| **No.** | **Question** | **Coding** |
| RT3 Q1 | Would you like to tell me about the third most recent time you exchanged sex? | O Yes  O No (skip to HIV01) |
| RT3 Q2 | Is this the same person we just spoke of for the last time you exchanged sex? | O Yes *(Do not ask RT3 Q8 thru Q13 or Q15 thru Q16)*  O No |
| RT3 Q3 | When did this sex exchange for money, goods, or gifts occur? | O I Know the Date  O Don’t Know (skip to RT3 Q5)  O Refused to Answer (skip to RT3 Q5) |
| RT3 Q4 | What was the date? | ___ ___/___ ___ ___/ ___ ___ ___ ___  D D M M M Y Y Y Y |
| RT3 Q5 | At what time of day did this encounter take place? | O Early Morning (00:01-08:00)  O Morning (8:01-12:00)  O Afternoon/Evening (12:01-18:00)  O Night (18:01-24:00) O Don’t Know  O Refused to Answer |
| RT3 Q6 | Where did you exchange sex with this person? | O Street, car, or outside  O Bar or nightclub  O Hotel room paid by sex worker  O Hotel room paid by partner  O Brothel  O Woman’s home  O Man’s home  O Other: _______________________________  O Don't Know  O Refused to Answer |
| RT3 Q7 | Please tell me which of the following activities you did during this encounter:  *Interviewer: Read the list of responses aloud to the participant and choose all that apply. Explain any of the choices that the participant does not understand.* | O Kissing  O Danced or stripped for partner  O Massage  O Performed oral sex **with** a condom  O Performed oral sex **without** a condom  O Vaginal sex **with** a condom  O Vaginal sex **without** a condom  O Anal sex **with** a condom  O Anal sex **without** a condom  O Received oral sex  O Talking/company  O Other: _______________________________  _______________________________________ |
| RT3 Q8 | *Do not ask if RT3 Q2 = Yes*  Was this the first time you have exchanged sex with this person? | O Yes  O No  O Don’t Know O Refused to Answer |
| RT3 Q9 | *Do not ask if RT3 Q2 = Yes*  How handsome was this person?  *Interviewer: Remember to probe for this question to determine what the participant thinks is handsome (dressed nicely, physical features, humor, etc.).* | O Not very handsome  O About average  O Handsome  O Don’t Know  O Refused to Answer |
| RT3 Q10 | *Do not ask if RT3 Q2 = Yes*  Approximately how old was this person? Please estimate. | ___ ___ years  O Don’t know  O Refused to answer |
| RT3 Q11 | *Do not ask if RT3 Q2 = Yes*  How wealthy was this person? | O Poor  O Average wealth  O Above-average wealth  O Very wealthy  O Don’t know  O Refused to answer |
| RT3 Q12 | *Do not ask if RT3 Q2 = Yes*  Does this person live in this County, or was he visiting? | O Lives in this County  O Just visiting  O Don’t know  O Refused to answer |
| RT3 Q13 | Did this person take alcohol or drugs around the time you had sex? | O Yes  O No  O Don’t know  O Refused to answer |
| RT3 Q14 | Did **you** take alcohol or drugs around the time you had sex with this person? | O Yes  O No  O Don’t know  O Refused to answer |
| RT3 Q15 | *Do not ask if RT3 Q2 = Yes*  How likely is it that this person had a sexually transmitted infection besides HIV? | O Almost impossible  O A little likely  O Somewhat likely  O Very likely  O Almost certain  O Don't Know  O Refused to Answer |
| RT3 Q16 | *Do not ask if RT3 Q2 = Yes*  How likely is it that this person had HIV? | O Almost impossible  O A little likely  O Somewhat likely  O Very likely  O Almost certain  O Don't Know  O Refused to Answer |
| RT3 Q17 | What did you receive in exchange for having sex with this person?  *Interviewer: Check all that apply.* | O Housing and/or utilities  O Food to eat  O Food to sell (example, fish)  O School fees  O To get a job, a work promotion, or to keep your job  O Other material goods (clothes, jewelry, makeup, electronics, etc.)  O Household items (soap, cleaning supplies, tools, etc.)  O Other: _______________________________  O Don’t Know  O Refused to Answer |
| RT3 Q18 | What was the **total value** of money, goods and gifts that the person gave you for this encounter? | KES_________  O Don't Know  O Refused to Answer |
| RT3 Q19 | Did this partner threaten you with physical assault? | O Yes  O No  O Don’t know  O Refused to answer |
| RT3 Q20 | Did this partner hit, kick, strangle or otherwise physically assault you? | O Yes  O No  O Don’t know  O Refused to answer |
| RT3 Q21 | Did this partner force or coerce you to participate in any sex act against your will? | O Yes, to have sex with a condom  O Yes, to have sex without a condom  O Yes, to have anal sex  O Yes, Other__________________________  O No  O Don’t know  O Refused to answer |

| **Section E: HIV and HIV Testing** | | |
| --- | --- | --- |
| **Section E1: General HIV Knowledge (HIV)**  Interviewer: I would now like to ask you some questions about what you already know and feel about HIV. | | |
| HIV01 | Are you currently taking any HIV medication in order to **prevent** acquiring HIV (PrEP)? This is usually a pill taken daily.  *Interviewer: Ensure participant understands the principles of PrEP before proceeding.* | O Yes  O No  O Don’t Know  O Refused to Answer |
| HIV02 | What do you think your chances are of acquiring HIV in the future? | O None (Ask HIV03, not HIV04)  O Low (Ask HIV03, not HIV04)  O Moderate (skip to HIV04)  O High (skip to HIV04)  O Don’t Know (skip to HIV05)  O Refused to Answer (skip to HIV05) |
| HIV03 | *Only ask if HIV02 = None or Low*  Why do you think you have a low chance or no chance of acquiring HIV in the future?  *Choose all that apply based on what participant mentioned.* | O Is Not Having Sex  O Uses Condoms  O Has Only One Partner  O Limits the Number of Partners  O Partner Has No Other Partners  O Knows Partner(s)’ HIV Status is Negative  O Trusts partner  O My Current Status is Negative  O Other _________________________________  O Don’t Know  O Refused to Answer |
| HIV04 | *Only ask if HIV02 = Moderate or High*  Why do you think you have a moderate or high chance of acquiring HIV in the future?  *Choose all that apply based on what the participant mentioned.* | O Does Not Use Condoms Regularly or at all  O Woman Has More Than One Partner  O Has Transactional Sex  O Does Not Trust Partner  O Partner is HIV positive  O She or Partner Refuses to be Tested  O Uses Injection Drugs/ Needles  O Primary Partner has more than one partner  O Non-primary partner(s) have more than one partner  O Other _________________________________  O Don’t Know  O Refused to Answer |
| HIV05 | Is it possible for one person in a steady sexual relationship to be infected with HIV and the other person to remain uninfected? | 1: Yes  2: No  98: Don’t Know  99: Refused to Answer |
| HIV06 | If 100 HIV negative men have unprotected sex **once** with a woman who is HIV positive, how many of the men will have HIV afterwards?  *Interviewer: Remember, this question is about learning the participant’s perception about risk of getting HIV if someone is exposed to it. The details (vaginal sex, lubrication, ARVs, etc.) aren’t important for this question – exposure is.* | Number of men: ___________  O Don’t know  O Refused to answer |
| HIV07 | Do you think that a person who has HIV can take medicine to reduce her risk of transmitting the virus to a sexual partner? | O Yes  O No  O Don’t know  O Refused to answer |
| HIV8 | Do you think that HIV can be cured? | O Yes  O No  O Don’t know  O Refused to answer |
| HIV9 | How often do you believe you should test for HIV?  *Choose all that apply.* | O Every week  O Every month  O Every 3 months  O Every 6 months  O Every year  O When I have sex without a condom  O When I have a new partner  O When my primary partner informs me or I know that he was unfaithful  O During pregnancy  O Other: ________________________________  O If I have been tested once, I do not need to be tested again  O Don’t Know  O Refused to Answer |
| **Section E2: Sexual Testing History (TST)**  Interviewer: I would now like to ask you some questions about testing for HIV and other sexually transmitted infections (STI) such as syphilis, gonorrhea, chlamydia, *Trichomonas vaginalis*, or bacterial vaginosis. | | |
| TST01 | Before the test you took today, had you ever been tested for HIV? | O Yes  O No (skip to TST03)  O Don’t Know (skip to TST03)  O Refused to Answer (skip to TST03) |
| TST02 | Before the test today, how long ago was your last HIV test? | O < 3 Months  O 3-6 Months  O 7-12 Months  O 13-23 Months  O 2 Years or More  O Don’t Know  O Refused to Answer |
| TST03 | During the past 6 months, have you been diagnosed with a STI? | O Yes  O No (skip to TST05)  O Don’t Know (skip to TST05)  O Refused to answer (skip to TST05) |
| TST04 | Which STI(s) were you diagnosed with?  *Choose all that apply.*  *Interviewer: This is self-reported by the participant. If she does not remember the diagnosis, use “Don’t Know”. Do not try to diagnose her using symptoms she describes.* | O Trichomoniasis (Trich)  O Syphilis  O Gonorrhea  O Chlamydia  O Herpes  O Human papillomavirus (HPV)  O Genital warts  O Mycoplasma genitalium  O Bacterial vaginosis (BV)  O Other _______________________________  O Don’t Know  O Refused to answer |
| TST05 | Did you **EVER** go to a pharmacy and purchase treatment for an STI, without consulting a doctor or other qualified healthcare provider (ex: over the counter medication)? | O Yes  O No  O Don’t Know  O Refused to answer |
| TST06 | Did you **EVER** use a home remedy or treatment for an STI, without consulting a doctor, pharmacist or other qualified healthcare provider? | O Yes  O No  O Don’t Know  O Refused to answer |
| **Section E3: HIV Self-testing (HST)**  Interviewer: As I mentioned before this interview, this study is interested in collecting information on HIV self-testing. HIV self-testing means you can test yourself for HIV at a time and location that is convenient for you, without having to go to a clinic. This test does not require a blood sample, but uses a sample from inside your mouth. The following questions are specific to HIV self-testing. | | |
| HST01 | Have you ever heard of HIV self-testing before today? | O Yes  O No (skip to HST03)  O Don’t Know (skip to HST03)  O Refused to Answer |
| HST02 | Have you ever used an HIV self-test before today? | O Yes  O No  O Don’t Know  O Refused to Answer |
| HST03 | If HIV self-testing were available to you, how interested would you be in testing yourself for HIV? | O Very Interested  O Somewhat Interested  O Neutral, Neither Interested nor Uninterested  O Somewhat Uninterested  O Very Uninterested  O Don’t Know  O Refused to Answer |
| HST04 | If HIV self-testing were available to you, do you think you would test for HIV more frequently? | O Yes, More Frequently  O No, Less Frequently  O No change in Frequency  O Don’t Know  O Refused to Answer |
| HST05 | If you received a positive HIV self-test result, how likely do you think you would be to seek confirmatory testing with a healthcare provider? | O Very Likely  O Somewhat Likely  O Neutral, Neither Likely Nor Unlikely  O Somewhat Unlikely  O Very Unlikely  O Don’t Know  O Refused to Answer |
| HST06 | How comfortable would you be in taking an HIV self-test to give to your **primary** partner to use? | O Very Comfortable  O Somewhat Comfortable  O Neutral, Neither Comfortable Nor Uncomfortable  O Somewhat Uncomfortable  O Very Uncomfortable  O Don’t Know  O Refused to Answer |
| HST07 | How comfortable would you be in taking an HIV self-test to give to your **non-primary** partner(s) to use? | O Very Comfortable  O Somewhat Comfortable  O Neutral, Neither Comfortable Nor Uncomfortable  O Somewhat Uncomfortable  O Very Uncomfortable  O Don’t Know  O Refused to Answer |

| **Section F: Gender-Based Violence (GBV)** | | |
| --- | --- | --- |
| Interviewer: The next questions are about things that happen within some relationships, and that your primary partner, or any other partners may have done to you. These questions are about violence, which may make you feel uncomfortable or distressed. You do not have to answer any question that makes you feel uncomfortable, and your responses are confidential. If you have any questions at any time, please ask me. Are you ready to continue? | | |
| GBV01:  In the past 12 months has your **PRIMARY** sexual partner done any of the following to you?  *Interviewer: Read each option aloud one at a time and choose all that apply.* | GBV02:  In the past 12 months, has your **PRIMARY** partner done this often or only sometimes? | GBV03:  Did you ever tell anyone that your **PRIMARY** partner did this, and if so who did you tell? |
| O Insulted or made you feel bad about yourself | O Often  O Sometimes  O Don’t Know  O Refused to Answer | O Yes: ______________  O No  O Don’t Know  O Refused to Answer |
| O Belittled or humiliated you in front of other people | O Often  O Sometimes  O Don’t Know  O Refused to Answer | O Yes: ______________  O No  O Don’t Know  O Refused to Answer |
| O Done anything to scare or intimidate you on purpose (by the way he looked at you, by yelling, smashing things, etc.) | O Often  O Sometimes  O Don’t Know  O Refused to Answer | O Yes: ______________  O No  O Don’t Know  O Refused to Answer |
| O Threatened to hurt you or someone you care about | O Often  O Sometimes  O Don’t Know  O Refused to Answer | O Yes: ______________  O No  O Don’t Know  O Refused to Answer |
| O Slapped, hit, or thrown something at you that could hurt you | O Often  O Sometimes  O Don’t Know  O Refused to Answer | O Yes: ______________  O No  O Don’t Know  O Refused to Answer |
| O Pushed or shoved you | O Often  O Sometimes  O Don’t Know  O Refused to Answer | O Yes: ______________  O No  O Don’t Know  O Refused to Answer |
| O Kicked, dragged, or beaten you | O Often  O Sometimes  O Don’t Know  O Refused to Answer | O Yes: ______________  O No  O Don’t Know  O Refused to Answer |
| O Strangled or burnt you on purpose | O Often  O Sometimes  O Don’t Know  O Refused to Answer | O Yes: ______________  O No  O Don’t Know  O Refused to Answer |
| O Threatened or has actually used a gun, knife, or other weapon that could hurt you | O Often  O Sometimes  O Don’t Know  O Refused to Answer | O Yes: ______________  O No  O Don’t Know  O Refused to Answer |
| O Fondled, groped, grabbed, or touched you in a way that was unwanted or made you feel unsafe | O Often  O Sometimes  O Don’t Know  O Refused to Answer | O Yes: ______________  O No  O Don’t Know  O Refused to Answer |
| O Forced you to have sex when you did not want to or could not provide consent (ex. you were too drunk or passed out) | O Often  O Sometimes  O Don’t Know  O Refused to Answer | O Yes: ______________  O No  O Don’t Know  O Refused to Answer |
| O None (skip to GBV04) | Not Applicable | Not Applicable |
| O Refused to Answer (skip to GBV04) | Not Applicable | Not Applicable |

| GBV04:  In the past 12 months has your **NON-PRIMARY** sexual partner done any of the following to you?  *Interviewer: Read each option aloud one at a time and choose all that apply.* | GBV05:  In the past 12 months, has your **NON-PRIMARY** partner done this often or only sometimes? | GBV06:  Did you ever tell anyone that your **NON-PRIMARY** partner did this, and if so who did you tell? |
| --- | --- | --- |
| O Insulted or made you feel bad about yourself | O Often  O Sometimes  O Don’t Know  O Refused to Answer | O Yes: ______________  O No  O Don’t Know  O Refused to Answer |
| O Belittled or humiliated you in front of other people | O Often  O Sometimes  O Don’t Know  O Refused to Answer | O Yes: ______________  O No  O Don’t Know  O Refused to Answer |
| O Done anything to scare or intimidate you on purpose (by the way he looked at you, by yelling, smashing things, etc.) | O Often  O Sometimes  O Don’t Know  O Refused to Answer | O Yes: ______________  O No  O Don’t Know  O Refused to Answer |
| O Threatened to hurt you or someone you care about | O Often  O Sometimes  O Don’t Know  O Refused to Answer | O Yes: ______________  O No  O Don’t Know  O Refused to Answer |
| O Slapped, hit, or thrown something at you that could hurt you | O Often  O Sometimes  O Don’t Know  O Refused to Answer | O Yes: ______________  O No  O Don’t Know  O Refused to Answer |
| O Pushed or shoved you | O Often  O Sometimes  O Don’t Know  O Refused to Answer | O Yes: ______________  O No  O Don’t Know  O Refused to Answer |
| O Kicked, dragged, or beaten you | O Often  O Sometimes  O Don’t Know  O Refused to Answer | O Yes: ______________  O No  O Don’t Know  O Refused to Answer |
| O Strangled or burnt you on purpose | O Often  O Sometimes  O Don’t Know  O Refused to Answer | O Yes: ______________  O No  O Don’t Know  O Refused to Answer |
| O Threatened or has actually used a gun, knife, or other weapon that could hurt you | O Often  O Sometimes  O Don’t Know  O Refused to Answer | O Yes: ______________  O No  O Don’t Know  O Refused to Answer |
| O Fondled, groped, grabbed, or touched you in a way that was unwanted or made you feel unsafe | O Often  O Sometimes  O Don’t Know  O Refused to Answer | O Yes: ______________  O No  O Don’t Know  O Refused to Answer |
| O Forced you to have sex when you did not want to or could not provide consent (ex. you were too drunk or passed out) | O Often  O Sometimes  O Don’t Know  O Refused to Answer | O Yes: ______________  O No  O Don’t Know  O Refused to Answer |
| O None (skip to GBV04) | Not Applicable | Not Applicable |
| O Refused to Answer (skip to GBV04) | Not Applicable | Not Applicable |

| **NOTES:** |
| --- |
|  |

**END OF BASELINE QUESTIONNAIRE**
