# Supplementary material for: Adoption of HIV pre-exposure prophylaxis among women at high risk of HIV infection in Kenya
Source: PLoS One. 2022 Sep 9;17(9):e0273409. doi: 10.1371/journal.pone.0273409 (PMC9462728; doi:10.1371/journal.pone.0273409)
Supplement: S2 File — (DOCX) [file pone.0273409.s002.docx]

Baseline Visit Questionnaire v7.2_Dholuo

| **No.** | **Question** | **Responses** | |
| --- | --- | --- | --- |
| 1 | Interviewer ID | ___ ___ | |
| 2 | Date of Screening | ___ ___/___ ___ ___/ ___ ___ ___ ___  D D M M M Y Y Y Y | |
| 3 | Enter Cluster ID | ___ ___ ___ | |
| 5 | Enter Name of Cluster |  | |
| 6 | Enter Name of Hotspot or Beach |  | |
| **Verbal Screening**  Read the Verbal Screening document to the participant and obtain consent to screen. | | | |
| 7 | Did the woman give verbal consent to participate in the screening process? | O Yes(skip to SCR1)  O No (answer #8 then skip to END) | |
| 8 | If the woman gave a reason why she did not want to be verbally consented, please indicate.  *Choose all that apply* | O No reason given or disclosed  O Woman did not have time/want to wait  O Woman did not want to answer screening questions  O Woman did not want to take an HIV test  O Woman needed permission from partner  O Other: __________________________________________  __________________________________________________ | |
| **Section 1: Screening Questions (SCR)**  Tell the participant you are going to ask her some questions to determine if she will be enrolled in the study. | | | |
| SCR1 | In jahigni adi?  What is your age?  *Interviewer: If participant does not know, ask for best guess, using schooling or other milestones to assist if necessary. Only after unsuccessful probing should you select response “Don’t Know”.* | | ___ ___ years (if less than 18, verify with SCR2)  O Don’t Know  O Refused to Answer |
| SCR2 | Onyuoli e higa mane?  In what year were you born?  *Interviewer: Probe if inconsistent with age. If participant guessed age and doesn’t know birth year, use the estimated age to calculate birth year.* | | ___ ___ ___ ___  Should match age given in SCR1;  If verified <18 years by SCR1 and SCR2, then skip to END |
| SCR3 | Idak Kanye?  Where do you live?  *Interviewer: determine if given locations are within the study region. Consult Study Coordinator for clarification if necessary.* | | O Within Study Region  O Outside Study Region (skip to END)  O Migratory (skip to END)  O Don’t Know (skip to END)  O Refused to Answer (skip to END) |
| SCR4 | Bende isedak e aluorani kuom dweche auchiel e dweche apar kod ariyo maokalo?  Have you lived in this area at least 6 out of the last 12 months?  *Interviewer: Use the confines of the study area to determine based on participant’s response.* | | O Yes  O No (skip to END)  O Don’t Know (skip to END)  O Refused to Answer (skip to END) |
| SCR5 | Bendeintiekodparo mar dak e aluorani (ka ma itayoenonro) kuomdweche 24 mabiro kata higni 2?  Do you plan to reside in the local area (study region) for the next 24 months or 2 years?  *Interviewer: A response of “Don’t Know” requires probing – only* ***definitive*** *plans to relocate should be used to exclude.* | | O Yes  O No (skip to END)  O Don’t Know  O Refused to Answer (skip to END) |
| SCR6 | Kuomjumbe 4 ma okalo, isebedokodjoheraariyo kata ma okaloariyo ma ibetgo e ringruok?  In the past 4 weeks, have you had two or more sexual partners?  *Interviewe*r: *Remember to introduce this question in a way that will not offend the participant.Only after unsuccessful probing should you select response “Don’t Know”.* | | O Yes  O No (skip to END)  O Don’t Know (skip to END)  O Refused to Answer (skip to END) |
| SCR7 | Bende in kodsimo mar ng’weyamo ma mari kata mar nga’tmachielo ma inyalogochne ma yudi?  Do you have your own mobile phone or regular access to someone else’s mobile phone? | | O Yes  O No (skip to END)  O Don’t Know (skip to END)  O Refused to Answer (skip to END) |
| SCR8 | Bendein jachiwre e nonromachielo mar gengokute mag ayaki e migaoni kata migaomachielo?  Are you currently enrolled in another HIV prevention study with this or any other organization? | | O Yes (skip to END)  O No  O Don’t Know (skip to END)  O Refused to Answer (skip to END) |
| **Section 2: HIV Rapid Test and DBS Collection**  For participants who are eligible to this point, perform HIV antibody rapid testing and enter results. Collect DBS card. | | | |
| SCR9 | What was the result of the HIV rapid test?  *Interviewer: If the HIV result is positive or indeterminate, ensure client receives any necessary confirmatory testing, proper counseling, and linkage to care.* | | O Negative (skip to SCR11)  O Positive (skip to SCR10)  O Indeterminate (skip to SCR10)  O Woman refused testing (skip to END) |
| SCR10 | Did you provide appropriate confirmatory testing, counseling, and linkage to care? | | O Yes  O No |
| **Section 3: Main Study Consent**  For eligible participants, read and obtain informed consent for the main study. | | | |
| SCR11 | After reviewing the informed consent document and asking questions, did the participant consent to participate in the main study? | | O Yes  O No (skip to END) |
| **END** | Erokamanokuomthuoloni kendo yiemondoidoukpenjo ma irangogokairomodonjo e nonroni. E sechegii, ok bi kawi kaka jachiwre e nonroni. Ma ok nyisni ok inyal bet jachiwre e nonromamoko ma itimosani kata ma ibirotim e kinde ma biro. Ka in kodpenjomoroamora, en mornamarduokogisani.  Thank you for your time and willingness to participate in the screening process for our study. At this time, however, you will not be enrolled as a participant in this study. This does not mean that you cannot participate in other research studies that are happening now or in the future. If you have any questions, I will be happy to answer them now. | | |

| **Section 4: Participant Identification** | | | | |
| --- | --- | --- | --- | --- |
| PID_GEN | Does a new PID need to be generated for this participant?  Note: Only select “Yes” if this is the first time this form is being completed for this participant. If there was a tablet error and you are re-entering data from another tablet, select “No” and enter the PID that was generated initially. | | | O Yes (skip to DEM01, ODK to generate and display ID)  O No |
| pid | Enter the participant’s ID number. Be extremely careful when entering the number. Double and triple check that the number is correct before proceeding. | | | ___ ___ ___ ___ ___ ___ ___ ___ ___ |
| pid_verify | Verify PID number | | | ___ ___ ___ ___ ___ ___ ___ ___ ___ |
| **Section 5: Baseline Questionnaire**  Screening form now complete. The Baseline survey starts here. | | | | |
| **Section B: Demographic Information (DEM)** | | | | |
| **No.** | | **Question** | **Coding** | |
| DEM01 | | In kod higni adi?  What is your age? | ___ ___  Verify with SCR1 | |
| DEM02 | | Okang’ mamalo mogik mar somo ma isetieko en mane?  What is the highest level of school you have *completed*?  *Interviewer: if DEM02=98, probe* | O None  O Some Primary  O Primary  O Some Secondary  O Secondary/ High School  O Post-Secondary/Training College  O University  O Don’t Know  O Refused to Answer | |
| DEM03 | | Kit Keny mari en mane sani?  What is your current marital status? | O Married, Living Together  O Married, NOT Living Together  O Not Married, Living Together  O Relationship but Not Married, NOT Living Together (boyfriend, etc.)  O Single  O Divorced  O Widowed  O Don’t Know  O Refused to Answer | |
| DEM04 | | Iwuok e dho oganda mane?  What is your ethnic group? | O Luo  O Luhya  O Kalenjin  O Kikuyu  O Kisii  O Ugandan  O Other: _______________________  O Don’t Know  O Refused to Answer | |
| DEM05 | | Yoo maduong’ mari mar yuto en mane?  What is your **primary** source of income? | O Professional/salaried  O Rental income (landlord, rent equipment)  O Sales and Service (Non-Fish)  O Skilled Manual  O Unskilled Manual  O Domestic Service  O Agriculture  O Fishing/ Fish Trade  O Sex Work  O Informal/Seasonal/ Piece Work  O Student  O Unemployed  O Other: ________________________________  _______________________________________  O Don’t Know  O Refused to Answer | |
| DEM06 | | Yori machielo mimedo gi yori maduong’mar yuto en mane, ka nitie?  What is/are your other source(s) of income, if any?  *Choose all that apply* | O Professional/salaried  O Rental income (landlord, rent equipment)  O Sales and Service (Non-Fish)  O Skilled Manual  O Unskilled Manual  O Domestic Service  O Agriculture  O Fishing/ Fish Trade  O Sex Work  O Informal/Seasonal/ Piece Work  O Student  O Other: _______________________________  ______________________________________  O None/ Not Applicable  O Don’t Know  O Refused to Answer | |
| DEM07 | | En omenda morom nade ma iyudo e dwe ka dwe kao kuom yoreni duto mag yuto?  How much income do you typically earn in one month, from all sources?  *Interviewer: Ask participant to give best estimate if not sure* | KES ___________________  O Don’t Know  O Refused to Answer | |
| DEM08 | | Ji adi ma odak e odi, ka iketri e kwan?  How many people live in your household, including yourself? | _______  O Refused to Answer | |
| DEM09 | | Ji adi ma odak e odi matiyo tije ma ichulogi dwe ka dwe, ka oriwo in?  How many people in your household have a regular or steady income, including yourself? | _______  O Don’t Know  O Refused to Answer  *Must be less than or equal to DEM08.* | |
| DEM10 | | Ekindegi gin ji adi mayudo kony ka owuok kuomi (ma imiyo chiemo, kama gidakie, pesa gi mamoko)?  How many people do you currently support (provide with food, housing, money, etc.)?  *Interviewer: This does not have to be a member of your household.* | _______  O Don’t Know  O Refused to Answer | |
| DEM11 | | E dwe mokalo, be isebedo ka ichiemo madirom diriyo e odiochieng’?  In the past month, have you regularly eaten at least two meals a day? | O Yes  O No  O Don’t Know  O Refused to Answer | |

| **Section C: Health and Sexual Behavior** | | |
| --- | --- | --- |
| **Section C1 – General Health (HLT)**  Interviewer: I have some questions about your physical and mental health.  Antie kod penjo moko ma oluwore kod ngimani e ringruok kod paro | | |
| **No.** | **Question** | **Coding** |
| HLT01 | Ere kaka inyalo pimo chal mar ngimani kanyakla?  How would you rate your overall health? | O Very Good  O Good  O Fair  O Poor  O Don’t Know  O Refused to Answer |
| HLT02 | E dweche 12 mokalo, inyalo pimo nadi kwan mar ndalo ma ne imadho kong’o?  In the past 12 months, what is your best estimate of how often you drank alcohol? | O Never (skip to HLT04)  O Less than once per month  O Once a month  O 2 to 3 times per month  O Once per week  O Twice per week  O 3 to 4 times a week  O 5 to 6 times per week  O Every Day  O Don’t Know  O Refused to Answer |
| HLT03 | Ei dwe mokalo, nyadidi mane imadhoe kong’o chupe madirom abich otieno achiel?  In the past month, how often did you drink 5 or more alcoholic drinks in one night? | O Never  O Only once  O 2 or 3 times  O Once per week  O Twice per week  O 3 to 4 times a week  O 5 to 6 times per week  O Every Day  O Don’t Know  O Refused to Answer |

| HLT04:  Ei jumbe ariyo mokalo, be weche machalo kod magi osechando pachi?  Over the last 2 weeks, have you been bothered by any of the following problems?  *Interviewer: Read each option aloud one at a time.* | HLT 05:  Ei jumbe ariyo mokalo, nyadidi ma weche machalo kod magi osechando pachi?  Over the last 2 weeks, how often have you been bothered by this problem? |
| --- | --- |
| O Ne ingi gombo matin kata siso mar timo gik moko  Had little interest or pleasure in doing things | O One Day  O Less than Half the Days  O Around Half the Days  O More than Half the Days  O Every Day  O Don’t Know  O Refused to Answer |
| O Ne ikuyo, ingi chuny machandore, kendo ionge geno.  Felt down, depressed, or hopeless | O One Day  O Less than Half the Days  O Around Half the Days  O More than Half the Days  O Every Day  O Don’t Know  O Refused to Answer |
| O Ne ok inyal chako nindo, kata nindo moyware kata ne inindo mangeny  Had trouble falling asleep/ staying asleep, or sleeping too much | O One Day  O Less than Half the Days  O Around Half the Days  O More than Half the Days  O Every Day  O Don’t Know  O Refused to Answer |
| O Ne iwinjo ka iol kata ionge teko  Felt tired or having little energy | O One Day  O Less than Half the Days  O Around Half the Days  O More than Half the Days  O Every Day  O Don’t Know  O Refused to Answer |
| O Ne iwinjo ka iol kata ionge teko  Had poor appetite or overeating | O One Day  O Less than Half the Days  O Around Half the Days  O More than Half the Days  O Every Day  O Don’t Know  O Refused to Answer |
| ONe iwinjo marach in iwuon kata ni ok ichopo dwachi kata ok i  Felt bad about yourself, felt that you are a failure, or felt that you let yourself or your family down | O One Day  O Less than Half the Days  O Around Half the Days  O More than Half the Days  O Every Day  O Don’t Know  O Refused to Answer |
| O Ne ingi chandruok e keto pachi e gik moko kaka somo oboke mar weche manyien kata neno televisen.  Had trouble concentrating on things such as reading the newspaper or watching television | O One Day  O Less than Half the Days  O Around Half the Days  O More than Half the Days  O Every Day  O Don’t Know  O Refused to Answer |
| O Ne idar kata iwuoyo mos ma jomamoko ne nyalo fwenyo ni ingi luoro kata ionge kwe kata wuodhi ngeny ma ok kaka pile  Moved or spoken so slowly that other people could have noticed, or been so fidgety/restless that you have moved around a lot more than usual | O One Day  O Less than Half the Days  O Around Half the Days  O More than Half the Days  O Every Day  O Don’t Know  O Refused to Answer |
| O Ne ingi paro ni nyalo bet maber ka itho, kata kelo hinyruok ne in e yoo moro amora.  Had thoughts that you would be better off dead, or of hurting yourself in some way | O One Day  O Less than Half the Days  O Around Half the Days  O More than Half the Days  O Every Day  O Don’t Know  O Refused to Answer |
| O None (skip to GSA01) | Not Applicable |
| O Refused to Answer (skip to GSA01) | Not Applicable |

| HLT06 | | Chandruokgi osekeloni pek marom nadi e timo tijeni, rito gikmoko e dala, kata winjruok gi jok ma moko?  How difficult have these problems made it for you to do your work, take care of things at home, or get along with other people? | | O Not Difficult at All  O Somewhat Difficult  O Very Difficult  O Don’t Know  O Refused to Answer |
| --- | --- | --- | --- | --- |
| **Section C2: General Sexual Activity (GSA)**  Ja taa penjo: Koro an kod penjo moko e wi yoreni mag bet e I achiel e ringruok, mondo abedgi ng’eyo matut e wii moko kuom gik madongo e kik ngimani. Ne penjogi “yore mag bet e achiel e ringruok” ohulo ni soyo duong’ dichuo e duong’ miyo kata olunde.  Interviewer: Now I have some questions about your sexual activity, in order to get a better understanding of some important aspects of your life. For these questions, “sexual activity” is defined as sexual penetration of the vagina or anus. | | | | |
| GSA01 | | Ne in jahigni adi mane ihango bedo e achiel e ringruok?  How old were you when you had sex for the first time?  *Interviewer: If participant doesn’t know exact age, probe for an estimate based on schooling or other life events.* | | ___ ___ years  O Don’t Know or Cannot Estimate  O Refused to Answer |
| GSA02 | | Chieng mokwongo mane ibedo e achiel e ringruok,bende jaherani ne ochuni mondo ibed kode e achiel e ringruok e yoo mora amora ka ok idwar?  The first time you had sex, did your partner force or coerce you into any sex act against your will? | | O Yes  O No  O Don’t Know  O Refused to Answer |
| GSA03 | | E dwe ma okalo, gin johera mopogore opogore adi ma isebedogo e achiel e ringruok?  During the past month, how many different sexual partners have you had? | | ___ ___  O Don’t Know  O Refused to Answer  *Interviewer: Probe if less than 2 – this is part of eligibility* |
| GSA04 | | Chieng mogik mane ibet achiel e ringruok, be ne itiyo gi rabo yunga?  The last time you had sex, did you use a condom? | | O Yes  O No  O Don’t Know  O Refused to Answer |
| GSA05 | | E kinde mar dwe achiel mokalo, nyadidi mane itiyo gi yo mar komo nyuol ma ok en rabo yunga mar dichuo mondo igeng mako ich?  During the past month, how often did you use a contraceptive device, other than the male condom, to prevent pregnancy? | | O Never (skip to PRP01)  O Less than Half the Time  O About Half the Time  O More than Half the Time  O Don’t Know  O Refused to Answer |
| GSA06 | | E dwe mokalo, gin yore mage mag komo nyuol mane itiyo go e gengo mako ich kopogore gi rabo yunga mar chuo?  In the past month, which of the following contraceptives, other than the male condom, have you used to prevent pregnancy? | | O Pill  O Hormone injection (Depo-Provera)  O Intrauterine device (IUD)  O Diaphragm  O Patch  O Implant (Norplant, Jadelle)  O Emergency pill (plan B or morning after pill)  O Spermicide  O Female condom  O Withdrawal  O Other:____________________________________  O Don’t Know  O Refused to Answer |
| **Section C3: Primary Partner (PRP)**  Ja taa penjo: Koro an kodi penjo e wi jaherani achiel mahie kata ngat ma ubetga godo e achiel e ringruok kinde ka kinde. Ma nyalo bedo jaodi, osiepni ma wuoyi, dichuo ma udak godo, kata ng’ama ubetgago e achiel e ringruok mang’eny. Jahera mahie en ng’at ma iwnjo kai hero moloyo, kata ngat ma inyalo nyiso wecheni ma iye/ opondo ka in thuolo. Ka inyalo dwaro kony e yeiro nf’ama jaherani mahie en, wanyalo wuoyo kuom mano e sechegi Section C3: Primary Partner (PRP).  Interviewer: Now I have some questions about your **one** primary or regular sexual partner. A primary partner could be your husband, boyfriend, or someone you **regularly** have sex with. A primary partner is the ONE partner you feel like you love or like the most, or the one you can confide in the most. If you would like help deciding who your primary partner is, we can talk about that now. | | | | |
| PRP01 | | Bende in kod johera mahie e kindeni, bende nyocha in kod moro e dwe ma okalo?Ma e ng’at ma wadwaro wuoyo e wiyi e penjo ma luwegi.  Do you currently have a primary partner, or have you had one in the past month? This is the man we will discuss in the following questions. | | O Yes  O No (skip to NPP01)  O Refused to Answer |
| PRP02 | | Edwe ma okalo, nyadidi mane itiyo kod rabo Oyunga kod jaherani mahie?  In the past month, how often did you use condoms with your primary partner? | | O Never  O Less than Half the Time  O About Half the Time  O More than Half of the Time  O Always (skip to PRP05)  O Don’t Know  O Refused to Answer |
| PRP03 | | E dwe mokalo ang’o ma omiyo ne ok utiyo gi rabo yunga e kindego duto gi jaherani mahie?  Why did you not use condoms all the time with your primary partner within the last month?  *Interviewer choose all that apply based on what the participant mentioned.* | | O I Did Not Want To  O Partner Did Not Want To  O No Condom was Available  O I Am Trying To Become Pregnant  O I Have Another Form of Contraception (IUD, Implant, Injectable, Pill, etc.)  O I Knew My Primary Partner’s HIV Status and decided a condom was not necessary  O I trust my primary partner  O Other: ____________________________________  ___________________________________________  O Don’t Know  O Refused to Answer |
| PRP04 | | E dwe mokalo, be isegabedo kod pek wuoyo kod jaherani ma hie mondo uti kod rabo yunga?  In the past month, have you ever had difficulty *negotiating* condom use with your primary partner? | | O Yes, always  O Yes, sometimes  O I have no difficulty negotiating  O I never try to get my partner to use condoms  O Don’t Know  O Refused to Answer |
| PRP05 | | Ei dweche 12 mokalo, be jaherani ma hie osedhi pimo kute mag ayaki?  In the past 12 months, has your primary partner gone for an HIV test? | | O Yes  O No  O Don’t Know  O Refused to Answer |
| PRP06 | | Bende ingeyo chal mar jaherani mar kute mag Ayaki?  Do you know your current primary partner’s HIV status? | | O No  O Yes, Partner is Positive  O Yes, Partner is Negative  O Yes, Refuse to Disclose Partner’s Status  O Refused to Answer |
| PRP07 | | Jaherani mahie ma in go sani nyalo bedo jahigni adi?(participant to give best guess)  About how old is your current primary partner? (participant to give best guess) | | ___ ___ years  O Don’t Know  O Refused to Answer |
| PRP08 | | Isebedo e tudruok mar bedo e achiel e ringruok kod jaherani mahie e kinde ma rom nade?  How long have you had a sexual relationship with your current primary partner?  *Interviewer: For relationships <1 month, record 01 months. (Ex: If participant says one week, record 01months)* | | ___ ___ years, ___ ___ months  O Don’t Know  O Refused to Answer |
| **Section C4: Non-Primary Partners (NPP)**  Ja taa penjo: Koro an kod penjo moko ewi jomoko ma ubetgago achiel e ringruok ka opogre gi jaherani ma hie. Ne penjogi, Awuoyo ewi osiepeni ma chuo ma ibetgo e achiel e ringruok, johera ma iromogo dichiel to ibetgo e acheil e ringruok, kata nga’t ma ibetgo e acheil e ringruok nikech chudo, i.e bet e achiel e ringruok ne pesa, mwandu, chiemo kata ot ma idakie.  Interviewer: Now I have some questions about any other sexual partners you may have had other than your primary partner. For these questions, I am referring to boyfriends, casual sexual partners, or someone with whom you engage in transactional sex, i.e. sex for money, goods gifts, food or housing. | | | | |
| NPP01 | E dwe ma okalo, ne ibet e achiel e ringruok kod johera ma nyalo romo adi ma ok gin joherani mahie?  How many clients (Don’t say clients if not a known FSW) non-primary sexual partners have you had in the past month?  *Interviewer: probe for an estimate if participant cannot immediately recall* | | | ___ ___ (If 00, skip to TRX01)  O Don’t Know  O Refused to Answer |
|  |  | | |  |
| NPP02 | E dwe ma okalo, ne ibet e achiel e ringruok kod johera ma nyalo romo adi ma ok gin joherani mahie, kendo ibet kodgi e ringruok **ding’eny**?  Of these, approximately how many did you have **multiple** sexual encounters with in the past month? | | | ___ ___ (If 00, skip to NPP05)  O Don’t Know  O Refused to Answer |
| NPP03 | Kuom jogo ma ne ibetgo e achiel e ringruok ding’eny e dwe ma okalo, nyadidi mane itiyo kod rabo yunga?  Among those with whom you had multiple sexual encountersin the past month how often did you use condoms? | | | O Never  O Less than Half the Time  O About Half the Time  O More than Half of the Time  O Always (skip to NPP05)  O Don’t Know  O Refused to Answer |
| NPP04 | E dwe mokalo ang’o momiyo ne ok utiyo gi rabo yunga gi johera ma ok mahie mane ubetgo achiel e ringruok ding’eny?  Why did you not always use condoms with those you shared multiple sexual encounters?  *Choose all that apply based on what the participant mentioned*  *.* | | | O I Did Not Want To  O Partner(s) Did Not Want To  O No Condom Was Available  O I Am Trying to Become Pregnant  O I Have Another Form of Contraception (IUD, Implant, Injectable, Pill, etc.)  O I Knew My Non-Primary Partner’s HIV Status  O I trust my non-primary partner(s)  O Partner offered me more money to not use a condom  O Other: ___________________________________  ___________________________________________  O Don’t Know  O Refused to Answer |
| NPP05 | Gin madirom johera adi ma ok gin joherani mahie ma ibetgago e achiel e ringruok kata johera moko ma ok ihiny betgo e achiel e ringruok mane ibetgo e achiel e ringruok dichiel kende e dwe mokalo?  Approximately how many (FSW only: clients or other) non-primary sexual partners in the past month did you have sex with **only once**? | | | ___ ___ (If 00, skip to TRX01)  O Don’t Know  O Refused to Answer |
| NPP06 | E kind chuo mane ibetgo e achiel e ringrouk dichiel kende e dwe ma okalo, ne itiyo kod rabo yunga mang’eny marom nadi ka ne ibete achiel e ringruok kodgi?  Among the men with whom you had only one sexual encounterin the past month, how often did you use condoms? | | | O Never  O Less than Half the Time  O About Half the Time  O More than Half of the Time  O Always (skip to TRX01)  O Don’t Know  O Refused to Answer |
| NPP07 | En ang’o mamiyo ne ok itiyo gi rabo yunga kod jogo mane iromogo kinde mane ibet e achiel e ringruok dichiel kende?  Why did you not always use condoms with those you had only one sexual encounter?  *Open ended for participants. Interviewer choose all that apply based on what participant mentioned.*  *.* | | | O I Did Not Want To  O Partner(s) Did Not Want To  O No Condom Was Available  O I Am Trying to Become Pregnant  O I Have Another Form of Contraception (IUD, Implant, Injectable, Pill, etc.)  O I Knew My Non-Primary Partner’s HIV Status  O I trust my non-primary partner(s)  O Partner offered me more money to not use a condom  O Other: _________________________  _________________________________  O Don’t Know  O Refused to Answer |
| **Section C5: Transactional Sex (TRX)**  Ja taa penjo: Koro daher penji penjo e wi johera mane nyalo bet ni ochuli pesa kata omiyi gimoro nikech ibet kodgi e achiel e ringruok. Magi nyalo bet chou mane ibetgo e achiel e ringruok dichiel kende, kata thuo mane ibetgo e achiel e rinruok ding’eny. Moko kuom penjogi nyalo nenore ni chalre gi moko ma asepenjo, to kata kamano par mana kuom ndalo mane ibet e achiel e ringruok mondo ochuli.  Interviewer: I would now like to ask questions about any sexual partners you may have had who paid you for sex or gave you something in exchange for sex. These may be men with whom you had only one sexual encounter, or men with whom you had multiple sexual encounters. Some of these questions may sound similar to ones I just asked, but please think only about those encounters where you exchanged sex. | | | | |
| TRX01 | | Be isega loko bet e achiel e ringruok mondo ochuli, omiyi muandu, otimni gimoro kata mondo omi gimoro timre?  Have you ever exchanged sex for money, goods, gifts, food, housing, services, or influence? | | O Yes  O No  O Refused to Answer |
| TRX02 | | Ne in kod higni adi mane ikuongo loko bet e achiel e ringruok ne pesa, mich, mwandu, chiemo, kar dak kata kony ma moko?  How old were you when you **first** exchanged sex for money, gifts, goods, food, housing or services?  *Interviewer: If participant doesn’t know, probe using life events such as schooling.* | | ___ ___ years *(Must be greater than or equal to GSA01)*  O Don’t Know  O Refused to Answer  *Must be greater than or equal to GSA01* |
| TRX03 | | E dweche 12 mokalo, bende isebet e achiel e ringruok ding’eny kata ma oluwore mondo ochuli pesa, omiyi mwandu, chiemo, kar dak kata kony mamoko?  In the past 12 months, have you **regularlyor repeatedly** exchanged sex for money, goods, food, housing or services? | | O Yes  O No  O Refused to Answer |
| TRX04 | | E dwe ma okalo, ne ibet e achiel e ringruok kod chuo madirom adi nikech chudo?  In the past month, with approximately how many men did you exchange sex? | | _________ (If 0, skip to TRX06)  O Don’t Know  O Refused to Answer |
| TRX05 | | Kuom chuo mane ibetgo e achiel e ringruok nikech chudo e dwe ma okalo, adi kuomgi ma ne ibetgo e achiel e ringruok nyading’eny?  Of these men in the past month with whom you exchanged sex, with how many did you have **multiple** sexual encounters? | | _________ *(Must be less than or equal to TRX04)*  O Don’t Know  O Refused to Answer |
| TRX06 | | E dweche adek ma okalo, bende isebet e achiel e ringruok nikech pesa, mwandu, kony ma moko kata imiyo gimoro otimora e aluora moro ka opogore gi______  *(Interviewer: insert name of cluster area)*?  In the last 3 months, have you exchanged sex for money, goods, services, or influence in a location other than ____? | | O Yes  O No (skip to TRX08)  O Refused to Answer (skip to TRX08) |
| TRX07 | | Yie mondo indik nying kuonde mamoko ma isebedoe e achiel eringruok mondo iyud chudo, omiyi mwandu, kony or imi gimoro timre e dweche 3 mokalo*.*  Please list the names of the other places or areas where you have exchanged sex for money, goods, services, or influence in the last 3 months.  *Interviewer: Use the cluster code for each area. See list for codes.* | | 1: ___ ___ ___  2: ___ ___ ___  3: ___ ___ ___  4: ___ ___ ___  5: ___ ___ ___ |
| TRX08 | | E dwe mokalo, bende isebedo e achiel e ringruok nikech idwaro pesa?  In the past month, have you exchanged sex for **money**? | | O Yes (skip to TRX13)  O No  O Refused to Answer |
| TRX09 | | *Ask only if client reported sex work as primary OR secondary income (DEM05 OR DEM06)*  E dwe ma okalo, bende ne imiyo ng’ato kaka manager, administraitor or pimp, kata ne ipogone ng’at machielo ata malo mar pesa ma ne ochuli nikech bet e achiel e ringruok?  During the past month, did you have to pay someone like a manager, administrator or pimp, or share with someone a percentage of the money that you received for sex? | | O Yes  O No  O Refused to Answer |
| TRX10 | | E dwe achiel, en pesa maromo nade ma iyudoga ka owuok kuom bet e achiel e ringruok nikech chudo?  In a typical month, how much of your income comes from exchanging sex for money? | | O None  O Less than half  O About half  O More than half, but not all  O All  O Don’t Know  O Refused to Answer |
| TRX11 | | Ka ibet e achiel e ringruok nikech chudo to itiyo kod rabo oyunga, en pesa madirom adi ma idwaroga ni ochuli kuom sa asaya ma ibet e achiel e ringruok?  When you exchange sex for money, what is the average amount of money you charge per sexual encounter when a condom **is** used? | | KES _________  O I Never Use a Condom  O Don’t Know  O Refused to Answer |
| TRX12 | | Ka ibet e achiel e ringruok nikech chudo to ok itiyo kod rabo oyunga, en pesa madirom adi ma idwaroga ni ochuli kuom sa asaya ma ibet e achiel e ringruok?  When you exchange sex for money, what is the average amount of money per sexual encounter when a condom**isnot** used? | | KES _________  O I Always Use a Condom  O Don’t Know  O Refused to Answer |
| TRX13 | | E dwe ma okalo, bende isebet e achiel e ringruok mondo iyud mwandu kata kony ma moko to ok pesa?  In the past month, have you exchanged sex for goods or services **other than** money? | | O Yes  O No (skip to TRX16)  O Refused to Answer (skip to TRX16) |
| TRX14 | | E dwe ma okalo, gin mwandu mage kata kony mage mane iyudo nikech bedo e achiel e ringruok?  In the past month, what goods or services have you exchanged for sex?  *Choose all that apply.* | | O Housing and/or utilities  O Food to eat  O Food to sell (example, fish)  O School fees  O To get a job, a work promotion, or to keep your job  O Other material goods (clothes, jewelry, makeup, electronics, etc.)  O Household items (soap, cleaning supplies, tools, etc.)  O Other (specify): ___________________________  O Don’t Know  O Refused to Answer |
| TRX15 | | *Ask only if TRX08 and/or TRX13 = Yes*  E dwe ma okalo, nyalo bedo nengo madirom pesa adi, mwandu kata kony madirom nade ma iyudo nikech bedo e achiel e ringruok?  In the past month, what is the approximate **total***value* of the money, goods, or services you received in exchange for sex?  *Interviewer: Make sure that participant understands to include how much the non-monetary goods/services are worth.* | | KES _______ (6 digits)  O Don’t Know  O Refused to Answer |
| TRX16 | | Bende isetamoriga bet e achiel e ringruok mondo mi iyud gima idwaro? Ka en kamano, ne en gi jaherani mahie kose jaherani ma ok irom go mang’eny?  Have you ever withheld sex in order to get something that you wanted? | | O Yes  O No (skip to SMH01)  O Don’t Know (skip to SMH01)  O Refused to Answer (skip to SMH01) |
| TRX17 | | Bende isetamoriga bet e achiel e ringruok kod jaherani mahie kata jaherani ma ok irom go mangeny mondo omi iyud gimoro mane idwaro  Did you withhold sex in order to get something from a primary partner or a non-primary partner? | | O Primary Partner  O Non-Primary Partner  O Both  O Don’t Know  O Refused to Answer |
| **Section C6: Social and Mental Health (SMH)**  Ja taa penjo: Koro daher penji penjo ewi kaka timbeni mag kisera osekelo lokruok ewi kaka inenori iwuon, kata okelo lokruok ewi kaka itudri gi jomamoko e anywolani kata ogandani.  Interviewer: Now I would like to ask you questions about how your sexual activity has impacted how you feel about yourself, or impacted your relationships with others in your family or community. | | | | |
| SMH01 | | Gin jok mage ma isepimonegi weche mag timbeni mag kisera, ka oriwo weche e wi jogo ma ibetgo e achiel e ringruok nikech chudo kata jok ma ok gin jaherani mahie ma ibetgo e achiel e ringruok?  Which individuals have you told about your sexual activities or that you have multiple partners?  *Choose all that apply.* | O No one  O A family member  O A friend  O A healthcare provider  O A community member: specify ___________________________  O My primary partner  O Other: specify ________________________________________  O Refused to Answer | |
| SMH02 | | E dweche 12 mokalo be achiel kuom gik maluwogi osetimoreni nikech Timbeni mag kisera?  In the past 12 months, have any of the following **actually** happened to you as a direct result of your sexual activity?  *Interviewer: Read each option aloud one at a time to the participant and choose all that apply before moving on to the next question.* | O Ne awinjo wichkuot kata ne kawora matin  O Jii ok omiya luor kata ok dewa e anyuola kata e oganda  O osetama donjo e romo kata riwruoge mag oganda kod kony (kaka kar thieth, kanisa, bura)  O osebuoga ni inyalo goya kata hinya kod ng’ato e anyuolana kata ogandana  O oseyanyi ayanga, sand kata buogi e yoo marach gi ng’ato e anyuolani kata e ogandani  O yanyi, kendo okwedi, kata jorit ngima ne ok otimoni kaka owinjore  O ose tamruok thietha  O onge moro amora kuom magi  O otamore duoko | |
| SMH03 | | E dweche 12 mokalo, bende isebet ka iluoro moro kuom gik ma nyalo bet ni timoreni nikech timbeni mag kisera  In the past 12 months, have you been **afraid** of any of the following **possibly** happening to you as a direct result of your sexual activity?  *Interviewer: Read each option aloud one at a time to the participant and choose all that apply before moving on to the next question.* | O Asebet ka aluor ni ji ok bi miya luor e anyuolana kata ogandana  O Asebet ka aluo dhi e romo kata kama ichiwoe kony (kaka kar theith, kanisa, festival, bura)  O Asebet ka aluor ni inyalo goya gi ng’ato e anyuolana kata ogandana  O Asebet ka aluor ni inyalo yanya, kata buoga kod ng’ato e anyuolana kata ogandana  O Asebet ka aluor ni anyalo yudo thieth ma ok owinjore koa kuom jochiw kony mag thieth kata ginyalo tamore miya thieth  O Asebet ka aluor ni ji nyalo paro ni an kod kute mag Ayaki  O Onge moro amora kuom magi  O Otamore duoko | |

| Interviewer to Read: Kuom penjogi, abiro somoni andiko. Akwayi mondo iler kaka iwinjo ewi andikogi  For this section, I will read a statement to you. Please describe how you feel about the statement. | | |
| --- | --- | --- |
| SMH04 | Chuo mang’eny e ogandani yanyoga, harass, kata buogo mine mabet e achiel e ringruok nikech pesa, mwandu, mich, chiemo, kar dak kata miyo gomoro otimre.  Most men in this community verbally insult, harass, or threaten women who exchange sex for money, goods, gifts, food, housing, or influence. | O Ok ayiego ahinya  O Ok Ayiego  O Ok en ni ayiego kata ok ayiego  O Ayiego  O Ayiego ahinya  O Ok angeyo  O Otamore duoko |
| SMH05 | Chuo mang’eny e ogandani goyoga, harass, kata buogo mine mabet e achiel e ringruok nikech pesa, mwandu, mich, chiemo, kar dak kata miyo gimoro otimre.  Most men in this community physically insult, harass, or threaten women who exchange sex for money, goods, gifts, food, housing, or influence. | O Ok ayiego ahinya  O Ok Ayiego  O Ok en ni ayiego kata ok ayiego  O Ayiego  O Ayiego ahinya  O Ok angeyo  O Otamore duoko |
| SMH06 | Chuo mang’eny e ogandani ok nyal kendo mine mabet e achiel e ringruok nikech pesa, mwandu, mich, chiemo, kar dak kata miyo gimoro otimre.  Most men in this community would not marry a woman who has exchanged sex for money, goods, gifts, food, housing, or influence. | O Ok ayiego ahinya  O Ok Ayiego  O Ok en ni ayiego kata ok ayiego  O Ayiego  O Ayiego ahinya  O Ok angeyo  O Otamore duoko |
| SMH07 | Ji mang’eny e ogandani ok omiyo luor mine mabet e achiel e ringruok nikech pesa, mwandu, mich, chiemo, kar dak kata miyo gimoro otimre.  Most people in this community do not respect women who exchange sex for money, goods, gifts, food, housing, or influence. | O Ok ayiego ahinya  O Ok Ayiego  O Ok en ni ayiego kata ok ayiego  O Ayiego  O Ayiego ahinya  O Ok angeyo  O Otamore duoko |
| SMH08 | Jogo machiwo kony mag thieth mang’eny e ogandani yanyoga, kwedo kata ok chiw kony ma owinjore ne mine mabet e achiel e ringruok nikech pesa, mwandu, mich, chiemo, kar dak kata miyo gimoro otimre.  Most healthcare providers in this community insult, discriminate, or treat poorly women who exchange sex for money, goods, gifts, food, housing, or influence. | O Ok ayiego ahinya  O Ok Ayiego  O Ok en ni ayiego kata ok ayiego  O Ayiego  O Ayiego ahinya  O Ok angeyo  O Otamore duoko |
| SMH09 | Jogo machiwo kony mag thieth mang’eny e ogandani tamorega chiwo kony ne mine mabet e achiel e ringruok nikech pesa, mwandu, mich, chiemo, kar dak kata miyo gimoro otimre.  Most healthcare providers in this community refuse services to women who exchange sex for money, goods, gifts, food, housing, or influence. | O Ok ayiego ahinya  O Ok Ayiego  O Ok en ni ayiego kata ok ayiego  O Ayiego  O Ayiego ahinya  O Ok angeyo  O Otamore duoko |

| **Section D: Recent Transactional Encounters (RT#)** | | |
| --- | --- | --- |
| Koro adhi penji penjo moko maluwore gi bedoni e achiel e ringruok gijoherani, motimore machiegni, mane omiyi pesa kata gimoro machielo nikech bet e achiel e ringruok. Ne asepenji penjo moko machal kodgi to koro adwaro ng’eyo weche moko matut. Adwoko erokamano maduong, ne horuok ma idhi bedogo e penjo maluwe, kata obedo ni nyalo nenore ni onwoyogi. Adwaro ni mondo ipar kuom ndalo macheigni mogik mane ibet e acheil e ringruok ne pesa, mwandu, mich, chiemo kata kar dak. Adhi penji penjo moko ewi lokoni.  Interviewer: Now, I will be asking you a series of questions related to your recent sexual partners from whom you received money or something else in exchange for sex. I have asked some similar questions but now I will ask for some more details. I greatly appreciate your patience through the next series of questions. I want you to think about the most recent time you exchanged sex for money, goods, gift, food, or housing. I am going to ask you several questions about this exchange. | | |
| **No.** | **Question** | **Coding** |
| RT1: Most Recent Exchange | | |
| RT1 Q1 | Be diher mar nyisa ewi bedo e achiel e ringruok manitimo mogik mondo iyud pesa, mwandu kata mich?  Would you like to tell me about the most recent time you exchanged sex? | O Yes  O No (skip to RT2 Q1) |
| RT1 Q2 | Ne en kar ang’o mogik mane iloko bedo e achiel e ringruok ne pesa, mwandu kata mich?  When did this sex exchange for money, goods, or gifts occur? | O I Know the Date  O Don’t Know (skip to RT1 Q4)  O Refused to Answer (skip to RTI Q34) |
| RT1 Q3 | Ne en tarik mane?  What was the date? | ___ ___/___ ___ ___/ ___ ___ ___ ___  D D M M M Y Y Y Y |
| RT1 Q4 | En seche mage e odiechieng’ mane mae otimore?  At what time of day did this encounter take place? | O Early Morning (00:01-08:00)  O Morning (8:01-12:00)  O Afternoon/Evening (12:01-18:00)  O Night (18:01-24:00) O Don’t Know  O Refused to Answer |
| RT1 Q5 | En kanye mane mae otimore?  Where did you exchange sex with this person? | O Street, car, or outside  O Bar or nightclub  O Hotel room paid by sex worker  O Hotel room paid by partner  O Brothel  O Woman’s home  O Man’s home  O Other: _______________________________  O Don't Know  O Refused to Answer |
| RT1 Q6 | E kindego, ne itimo mane kuom gik ma okwan kaegi?  Please tell me which of the following activities you did during this encounter:  *Interviewer: Read the list of responses aloud to the participant and choose all that apply. Explain any of the choices that the participant does not understand.* | O Nyodhruok  O Ne imiel kata igloo lepi ni jaherani.  O Rwayo.  O Bedo e achiel e ringruok e yor dhok ka itiyo kod rabo yunga.  O Bedo e achiel e ringruok e yor dhok ka ok itiyo kod rabo yunga.  O Bedo e achiel e ringruok ka okalo e duong’ miyo ka itiyo kod rabo yunga.  O Bedo e achiel e ringruok ka okalo e duong’ miyo ka ok itiyo kod rabo yunga.  O Bedo e achiel e ringruok ka okalo e olund miyo ka itiyo kod rabo yunga.  O Bedo e achiel e ringruok ka okalo e olund miyo ka ok itiyo kod rabo yunga.  O Otimni bedo e achiel e ringruok kokalo e dhok  O Wuoyo / goyo mbaka  O Other: _______________________________  _______________________________________ |
| RT1 Q7 | Bende ma e chieng mokwongo mane ibedo e achiel e ringruok kod ngatni?  Was this the first time you have exchanged sex with this person? | O Yes  O No  O Don’t Know O Refused to Answer |
| RT1 Q8 | Nga’tni be ne nigi chia (jaber)?  How handsome was this person?  *Interviewer: Remember to probe for this question to determine what the participant thinks is handsome (dressed nicely, physical features, humor, etc.).* | O Not very handsome  O About average  O Handsome  O Don’t Know  O Refused to Answer |
| RT1 Q9 | Ng’ani ne en jahigni adi?  Approximately how old was this person? Please estimate. | ___ ___ years  O Don’t know  O Refused to answer |
| RT1 Q10 | Ng’ani ne nigi mwandu marom nadi?  How wealthy was this person? | O Poor  O Average wealth  O Above-average wealth  O Very wealthy  O Don’t know  O Refused to answer |
| RT1 Q11 | Ng’atni ae e Siaya County koso ne timo limbe?  Does this person live in this County, or was he visiting? | O Lives in this County  O Just visiting  O Don’t know  O Refused to answer |
| RT1 Q12 | Bende ng’atni ne otiyo kod kong’o ka pok ubedo e achiel e ringruok, seche ma ubet e achiel e ringruok kata bang’ ka usebedo e achiel e ringruok?  Did this person take alcohol or drugs around the time you had sex? | O Yes  O No  O Don’t know  O Refused to answer |
| RT1 Q13 | Bende ne itiyo kod yedhe ma mero ji ka pok ubet e achiel e ringruok, seche ma ubet e achiel e ringruok kata bang’ ka usebedo e achiel e ringruok?  Did YOU take alcohol or drugs around the time you had sex with this person? | O Yes  O No  O Don’t know  O Refused to answer |
| RT1 Q14 | Bende nyalore ni jaherani ne nyalo bedo gi twoche mag nyeye?  How likely is it that this person had a sexually transmitted infection besides HIV? | O Almost impossible  O A little likely  O Somewhat likely  O Very likely  O Almost certain  O Don't Know  O Refused to Answer |
| RT1 Q15 | Bende nyalore ni jaherani ne nyalo bedo gi kute mag Ayaki?  How likely is it that this person had HIV? | O Almost impossible  O A little likely  O Somewhat likely  O Very likely  O Almost certain  O Don't Know  O Refused to Answer |
| RT1 Q16 | En ang’o mane iyudo e loko bedo e achiel e ringruok kod ngatni?  What did you receive in exchange for having sex with this person?  *Interviewer: Check all that apply.* | O Housing and/or utilities  O Food to eat  O Food to sell (example, fish)  O School fees  O To get a job, a work promotion, or to keep your job  O Other material goods (clothes, jewelry, makeup, electronics, etc.)  O Household items (soap, cleaning supplies, tools, etc.)  O Other: _______________________________  O Don’t Know  O Refused to Answer |
| RT1 Q17 | Duto,pesa, mwandu kod mich mane jaherani omiyi chieng’no ne gin madirom pesa adi?  What was the **total value** of money, goods and gifts that the person gave you for this encounter? | KES_________  O Don't Know  O Refused to Answer |
| Interviewer: Koro adhi penji penjo moko e wi gik mane jaherani otimoni e kindeno. Penjogi gin e wi tulo, manyalo miyo ok ibed thuolo kata ibuogi. Akwayi ni mondo ing’e ni duoko ma ichiwo ibiro rito e yor maling’ ling’ kendo onge ng’ama biro tudo duokoni kodi. Ok ochuno ni nyaka iduok penjo moro amora ma miyo ok ibed thuolo. Akwayi ni ikaw thuolo ni mar penja penjo moro amora ma ok winjre ni maber. Bende in thuolo mar dhi nyime?  Interviewer READ: I will now ask you some questions about things this same person may have done to you during this encounter. These questions are about violence, which may make you feel uncomfortable or distressed. Please remember that your answers are completely confidential and no one will be able to associate this information with you or your sexual partners. You do not have to answer any question that makes you uncomfortable. Please take your time and if you are unclear about any question, just ask me. Are you ready to continue? | | |
| RT1 Q18 | Bende jaherani ne obuogi ni onyalo hinyo dendi?  Did this partner threaten you with physical assault? | O Yes  O No  O Don’t know  O Refused to answer |
| RT1 Q19 | Bende jaherani ne ogoyi, ogweyi, odeyi kata ohinyo dendi e yoo moro amora?  Did this partner hit, kick, strangle or otherwise physically assault you? | O Yes  O No  O Don’t know  O Refused to answer |
| RT1 Q20 | Bende jaherani ne ochuni mondo ibed e achiel e ringruok e yoo moro amora ma ok ne idwar?  Did this partner force or coerce you to participate in any sex act against your will? | O Yes, to have sex with a condom  O Yes, to have sex without a condom  O Yes, to have anal sex  O Yes, Other____________________________  O No  O Don’t know  O Refused to answer |
| Interviewer: Koro adhi penji penjo machalre kod masepenji, kuom bedo e acheil e ringruok mane itimo machiegni mar ariyo mondo iyud gimoro. Mae nyalo bedo kod johera ma opogore kod ma wawuoyo e wiye, kata ma wesewuoyo e wiye.  Interviewer: Now, I will be asking you the same questions as I just did, for your second most recent encounter where you exchanged sex for something. This could be with a different partner as the one we just discussed, or the same. | | |
| RT2: Second Most Recent Exchange | | |
| **No.** | **Question** | **Coding** |
| RT2 Q1 | Bende diher mar nyisa ewi bedo e achiel e ringruok manitimo machiegni mogik mar ariyo mondo iyud pesa, mwandu kata mich?  Would you like to tell me about the second most recent time you exchanged sex? | O Yes  O No (skip to RT3 Q1) |
| RT2 Q2 | Bende ma en ngat manende wawuoyo e wiyemane ibedo godo e achiel e ringruok mogik?  Is this the same person we just spoke of for the last time you exchanged sex? | O Yes *(Do not ask RT2 Q8 thru Q13 or Q15 thru Q16)*  O No |
| RT2 Q3 | Ne en karang’o mane loko bedo e achiel e ringruok ne pesa, mwandu kata mich otimore?  When did this sex exchange for money, goods, or gifts occur? | O I Know the Date  O Don’t Know (skip to RT1 Q4)  O Refused to Answer (skip to RTI Q34) |
| RT2 Q4 | Ne en Tarik mane?  What was the date? | ___ ___/___ ___ ___/ ___ ___ ___ ___  D D M M M Y Y Y Y |
| RT2 Q5 | Ne en saa adi mar odiechieng’ mane mae otimore?  At what time of day did this encounter take place? | O Early Morning (00:01-08:00)  O Morning (8:01-12:00)  O Afternoon/Evening (12:01-18:00)  O Night (18:01-24:00) O Don’t Know  O Refused to Answer |
| RT2 Q6 | En kanye mane mae otimore?  Where did you exchange sex with this person? | O Street, car, or outside  O Bar or nightclub  O Hotel room paid by sex worker  O Hotel room paid by partner  O Brothel  O Woman’s home  O Man’s home  O Other: _______________________________  O Don't Know  O Refused to Answer |
| RT2 Q7 | Yie I nyisa mane kuom magi mane itimo e kindeno?  Please tell me which of the following activities you did during this encounter:  *Interviewer: Read the list of responses aloud to the participant and choose all that apply. Explain any of the choices that the participant does not understand.* | O Nyodhruok  O Ne imiel kata igloo lepi ni jaherani.  O Rwayo.  O Bedo e achiel e ringruok e yor dhok ka itiyo kod rabo yunga.  O Bedo e achiel e ringruok e yor dhok ka ok itiyo kod rabo yunga.  O Bedo e achiel e ringruok ka okalo e duong’ miyo ka itiyo kod rabo yunga.  O Bedo e achiel e ringruok ka okalo e duong’ miyo ka ok itiyo kod rabo yunga.  O Bedo e achiel e ringruok ka okalo e olund miyo ka itiyo kod rabo yunga.  O Bedo e achiel e ringruok ka okalo e olund miyo ka ok itiyo kod rabo yunga.  O Otimni bedo e achiel e ringruok kokalo e dhok  O Wuoyo / goyo mbaka  O Other: _______________________________  _______________________________________ |
| RT2 Q8 | *Do not ask if RT2 Q2 = Yes*  Bende ma e chieng mokwongo mane ibedo e achiel e ringruok kod ngatni?  Was this the first time you have exchanged sex with this person? | O Yes  O No  O Don’t Know O Refused to Answer |
| RT2 Q9 | *Do not ask if RT2 Q2 = Yes*  Nga’tni be ne nigi chia (jaber)?  How handsome was this person?  *Interviewer: Remember to probe for this question to determine what the participant thinks is handsome (dressed nicely, physical features, humor, etc.).* | O Not very handsome  O About average  O Handsome  O Don’t Know  O Refused to Answer |
| RT2 Q10 | *Do not ask if RT2 Q2 = Yes*  Ng’ani ne en jahigni adi?  Approximately how old was this person? Please estimate. | ___ ___ years  O Don’t know  O Refused to answer |
| RT2 Q11 | *Do not ask if RT2 Q2 = Yes*  Ng’ani ne nigi mwandu marom nadi  How wealthy was this person? | O Poor  O Average wealth  O Above-average wealth  O Very wealthy  O Don’t know  O Refused to answer |
| RT2 Q12 | *Do not ask if RT2 Q2 = Yes*  Ng’atni ae e Siaya County koso ne timo limbe?  Does this person live in this County, or was he visiting? | O Lives in this County  O Just visiting  O Don’t know  O Refused to answer |
| RT2 Q13 | Bende ng’atni ne otiyo kod kong’o ka pok ubedo e achiel e ringruok, seche ma ubet e achiel e ringruok kata bang’ ka usebedo e achiel e ringruok?  Did this person take alcohol or drugs around the time you had sex? | O Yes  O No  O Don’t know  O Refused to answer |
| RT2 Q14 | Bende ne itiyo kod kong’o ka pok ubet e achiel e ringruok, seche ma ubet e achiel e ringruok kata bang’ ka usebedo e achiel e ringruok?  Did **you** take alcohol or drugs around the time you had sex with this person? | O Yes  O No  O Don’t know  O Refused to answer |
| RT2 Q15 | *Do not ask if RT2 Q2 = Yes*  Bende nyalore ni jaherani ne nyalo bedo gi twoche mag nyeye?  How likely is it that this person had a sexually transmitted infection besides HIV? | O Almost impossible  O A little likely  O Somewhat likely  O Very likely  O Almost certain  O Don't Know  O Refused to Answer |
| RT2 Q16 | *Do not ask if RT2 Q2 = Yes*  Bende nyalore ni jaherani ne nyalo bedo gi kute mag Ayaki?  How likely is it that this person had HIV? | O Almost impossible  O A little likely  O Somewhat likely  O Very likely  O Almost certain  O Don't Know  O Refused to Answer |
| RT2 Q17 | En ang’o mane iyudo e loko bedo e achiel e ringruok kod ng’atni?  What did you receive in exchange for having sex with this person?  *Interviewer: Check all that apply.* | O Housing and/or utilities  O Food to eat  O Food to sell (example, fish)  O School fees  O To get a job, a work promotion, or to keep your job  O Other material goods (clothes, jewelry, makeup, electronics, etc.)  O Household items (soap, cleaning supplies, tools, etc.)  O Other: _______________________________  O Don’t Know  O Refused to Answer |
| RT2 Q18 | Duto, pesa, mwandu kod mich mane jaheni omiyi chieng’no ne gin madirom pesa adi?  What was the **total value** of money, goods and gifts that the person gave you for this encounter? | KES_________  O Don't Know  O Refused to Answer |
| RT2 Q19 | Bende jaherani ne obuogi ni onyalo hinyo dendi?  Did this partner threaten you with physical assault? | O Yes  O No  O Don’t know  O Refused to answer |
| RT2 Q20 | Bende jaherani ne ogoyi, ogweyi, odeyi kata ohinyo dendi e yoo moro amora?  Did this partner hit, kick, strangle or otherwise physically assault you? | O Yes  O No  O Don’t know  O Refused to answer |
| RT2 Q21 | Bende jaherani ne ochuni mondo ibed e achiel e ringruok e yoo moro amora ma ok ne idwar?  Did this partner force or coerce you to participate in any sex act against your will? | O Yes, to have sex with a condom  O Yes, to have sex without a condom  O Yes, to have anal sex  O Yes, Other__________________________  O No  O Don’t know  O Refused to answer |
| Interviewer: Mogik, adhi penji penjo moko kaka ne apenji kuom bedo e acheil e ringruok mane itimo machiegni mar adek mondo iyud gimoro. Mae nyalo bedo kod johera ma opogore kod ma wawuoyo e wiye, kata ma wesewuoyo e wiye.  Interviewer: For the last time, I will be asking you the same questions as I just did about your third most recent encounter where you exchanged sex for something. This could be with a different partner as the one we just discussed, or the same. | | |
| RT3: Third Most Recent Exchange | | |
| **No.** | **Question** | **Coding** |
| RT3 Q1 | Bende diher mar nyisa ewi bedo e achiel e ringruok manitimo mar adek mogik mondo iyud pesa, mwandu kata mich  Would you like to tell me about the third most recent time you exchanged sex? | O Yes  O No (skip to HIV01) |
| RT3 Q2 | Bende ma en ngat manende wawuoyo e wiye mane ibedo godo e achiel e ringruok mogik?  Is this the same person we just spoke of for the last time you exchanged sex? | O Yes *(Do not ask RT3 Q8 thru Q13 or Q15 thru Q16)*  O No |
| RT3 Q3 | Ne en kar ang’o mane loko bedo e achiel e ringruok ne pesa, mwandu kata mich otimore  When did this sex exchange for money, goods, or gifts occur? | O I Know the Date  O Don’t Know (skip to RT1 Q4)  O Refused to Answer (skip to RTI Q34) |
| RT3 Q4 | Ne en tarik mane  What was the date? | ___ ___/___ ___ ___/ ___ ___ ___ ___  D D M M M Y Y Y Y |
| RT3 Q5 | En seche mage mane mae otimore?    At what time of day did this encounter take place? | O Early Morning (00:01-08:00)  O Morning (8:01-12:00)  O Afternoon/Evening (12:01-18:00)  O Night (18:01-24:00) O Don’t Know  O Refused to Answer |
| RT3 Q6 | En kanye mane mae otimore?  Where did you exchange sex with this person? | O Street, car, or outside  O Bar or nightclub  O Hotel room paid by sex worker  O Hotel room paid by partner  O Brothel  O Woman’s home  O Man’s home  O Other: _______________________________  O Don't Know  O Refused to Answer |
| RT3 Q7 | Yie inyisa gin ang’o kuom magi mane otimore e kindeno?  Please tell me which of the following activities you did during this encounter:  *Interviewer: Read the list of responses aloud to the participant and choose all that apply. Explain any of the choices that the participant does not understand.* | O Nyodhruok  O Ne imiel kata igloo lepi ni jaherani.  O Rwayo.  O Bedo e achiel e ringruok e yor dhok ka itiyo kod rabo yunga.  O Bedo e achiel e ringruok e yor dhok ka ok itiyo kod rabo yunga.  O Bedo e achiel e ringruok ka okalo e duong’ miyo ka itiyo kod rabo yunga.  O Bedo e achiel e ringruok ka okalo e duong’ miyo ka ok itiyo kod rabo yunga.  O Bedo e achiel e ringruok ka okalo e olund miyo ka itiyo kod rabo yunga.  O Bedo e achiel e ringruok ka okalo e olund miyo ka ok itiyo kod rabo yunga.  O Otimni bedo e achiel e ringruok kokalo e dhok  O Wuoyo / goyo mbaka  O Other: _______________________________  __________________________________________ |
| RT3 Q8 | *Do not ask if RT3 Q2 = Yes*  Bende ma e chieng mokwongo mane ibedo e achiel e ringruok kod ngatni?  Was this the first time you have exchanged sex with this person? | O Yes  O No  O Don’t Know O Refused to Answer |
| RT3 Q9 | *Do not ask if RT3 Q2 = Yes*  Nga’tni be ne nigi chia (jaber)?  How handsome was this person?  *Interviewer: Remember to probe for this question to determine what the participant thinks is handsome (dressed nicely, physical features, humor, etc.).* | O Not very handsome  O About average  O Handsome  O Don’t Know  O Refused to Answer |
| RT3 Q10 | *Do not ask if RT3 Q2 = Yes*  Ng’ani ne en jahigni adi?  Please estimate.  Approximately how old was this person? Please estimate. | ___ ___ years  O Don’t know  O Refused to answer |
| RT3 Q11 | *Do not ask if RT3 Q2 = Yes*  Ng’ani ne nigi mwandu marom nadi  How wealthy was this person? | O Poor  O Average wealth  O Above-average wealth  O Very wealthy  O Don’t know  O Refused to answer |
| RT3 Q12 | *Do not ask if RT3 Q2 = Yes*  Ng’atni ae e Siaya County koso ne timo limbe?  Does this person live in this County, or was he visiting? | O Lives in this County  O Just visiting  O Don’t know  O Refused to answer |
| RT3 Q13 | Bende ng’atni ne otiyo kod kong’o ka pok ubedo e achiel e ringruok, seche ma ubet e achiel e ringruok kata bang’ ka usebedo e achiel e ringruok?  Did this person take alcohol or drugs around the time you had sex? | O Yes  O No  O Don’t know  O Refused to answer |
| RT3 Q14 | Bende ne itiyo kod kong’o ka pok ubet e achiel e ringruok, seche ma ubet e achiel e ringruok kata bang’ ka usebedo e achiel e ringruok?  Did **you** take alcohol or drugs around the time you had sex with this person? | O Yes  O No  O Don’t know  O Refused to answer |
| RT3 Q15 | *Do not ask if RT3 Q2 = Yes*  Bende nyalore ni jaherani ne nyalo bedo gi twoche mag nyeye?  How likely is it that this person had a sexually transmitted infection besides HIV? | O Almost impossible  O A little likely  O Somewhat likely  O Very likely  O Almost certain  O Don't Know  O Refused to Answer |
| RT3 Q16 | *Do not ask if RT3 Q2 = Yes*  Bende nyalore ni jaherani ne nyalo bedo gi kute mag Ayaki?  How likely is it that this person had HIV? | O Almost impossible  O A little likely  O Somewhat likely  O Very likely  O Almost certain  O Don't Know  O Refused to Answer |
| RT3 Q17 | En ang’o mane iyudo e loko bet e achiel kod ng’atni?  What did you receive in exchange for having sex with this person?  *Interviewer: Choose all that apply.* | O Housing and/or utilities  O Food to eat  O Food to sell (example, fish)  O School fees  O To get a job, a work promotion, or to keep your job  O Other material goods (clothes, jewelry, makeup, electronics, etc.)  O Household items (soap, cleaning supplies, tools, etc.)  O Other: _______________________________  O Don’t Know  O Refused to Answer |
| RT3 Q18 | Duto pesa, mwandu kod mich mane jaheni omiyi chieng’no ne gin madirom pesa adi?  What was the **total value** of money, goods and gifts that the person gave you for this encounter? | KES_________  O Don't Know  O Refused to Answer |
| RT3 Q19 | Bende jaherani ne obuogi ni onyalo hinyo dendi?  Did this partner threaten you with physical assault? | O Yes  O No  O Don’t know  O Refused to answer |
| RT3 Q20 | Bende jaherani ne ogoyi, ogweyi, odeyi kata ohinyo dendi e yoo moro amora?  Did this partner hit, kick, strangle or otherwise physically assault you? | O Yes  O No  O Don’t know  O Refused to answer |
| RT3 Q21 | Bende jaherani ne ochuni mondo ibed e achiel e ringruok e yoo moro amora ma ok ne idwar?  Did this partner force or coerce you to participate in any sex act against your will? | O Yes, to have sex with a condom  O Yes, to have sex without a condom  O Yes, to have anal sex  O Yes, Other__________________________  O No  O Don’t know  O Refused to answer |

| **Section E: HIV and HIV Testing** | | |
| --- | --- | --- |
| **Section E1: General HIV Knowledge (HIV)**  Ja taa nonro: Koro daher penji penjo ewi gik ma ing’eyo kata kaka iparo ewi kute mag Ayaki.  Interviewer: I would now like to ask you some questions about what you already know and feel about HIV. | | |
| HIV01 | E kindegi, bende itiyogi yedhe mag gayo kute mag ayaki (PrEP)? Ma en yath ma imuonyo pile pile.  Are you currently taking any HIV medication in order to **prevent** acquiring HIV (PrEP)? This is usually a pill taken daily.  *Interviewer: Ensure participant understands the principles of PrEP before proceeding.* | O Yes  O No  O Don’t Know  O Refused to Answer |
| HIV02 | Iparo ni thuoloni mag gamo kute mag Ayaki; onge, nipiny, nidiere, koso nimalo?  What do you think your chances are of acquiring HIV in the future? | O None (Ask HIV03, not HIV04)  O Low (Ask HIV03, not HIV04)  O Moderate (skip to HIV04)  O High (skip to HIV04)  O Don’t Know (skip to HIV05)  O Refused to Answer (skip to HIV05) |
| HIV03 | *Only ask if HIV02 = None or Low*  Ang’o ma omiyo iparo ni in kod thuolo ma piny kata ionge thuolo mar gamo kute mag ayaki?  Why do you think you have a low chance or no chance of acquiring HIV in the future?  *Choose all that apply based on what participant mentioned.* | O Is Not Having Sex  O Uses Condoms  O Has Only One Partner  O Limits the Number of Partners  O Partner Has No Other Partners  O Knows Partner(s)’ HIV Status is Negative  O Trusts partner  O My Current Status is Negative  O Other _________________________________  O Don’t Know  O Refused to Answer |
| HIV04 | *Only ask if HIV02 = Moderate or High*  Ang’o ma omiyo iparo ni in kod thuolo man e diere kata ma malo mar gamo kute mag Ayaki?  Why do you think you have a moderate or high chance of acquiring HIV in the future?  *Choose all that apply based on what the participant mentioned.* | O Does Not Use Condoms Regularly or at all  O Woman Has More Than One Partner  O Has Transactional Sex  O Does Not Trust Partner  O Partner is HIV positive  O She or Partner Refuses to be Tested  O Uses Injection Drugs/ Needles  O Primary Partner has more than one partner  O Non-primary partner(s) have more than one partner  O Other _________________________________  O Don’t Know  O Refused to Answer |
| HIV05 | Bende nyalore ni ngat manie tudruok motegno nyalo bedo gi kute mag ayaki, to ngat machielo onge gi kute mag ayaki?  Is it possible for one person in a steady sexual relationship to be infected with HIV and the other person to remain uninfected? | O Yes  O No  O Don’t Know  O Refused to Answer |
| HIV06 | Ka chuo 100 ma onge kod kute mag Ayaki obet e achiel e ringruok dichiel kod miyo man kod kute mag Ayaki to ok gitiyo kod rabo oyunga, iparo ni ji adi kuomgi ma nyalo yudo kute mag Ayaki bang’e?  If 100 HIV negative men have unprotected sex **once** with a woman who is HIV positive, how many of the men will have HIV afterwards?  *Interviewer: Remember, this question is about learning the participant’s perception about risk of getting HIV if someone is exposed to it. The details (vaginal sex, lubrication, ARVs, etc.) aren’t important for this question – exposure is.* | Number of men: ___________  O Don’t know  O Refused to answer |
| HIV07 | Bende iparo ni ng’at ma ni kod kute mag ayaki nyalo tiyo gi yath ma duok chien thuolo mar miyo jaherane kute?  Do you think that a person who has HIV can take medicine to reduce her risk of transmitting the virus to a sexual partner? | O Yes  O No  O Don’t know  O Refused to answer |
| HIV08 | Be iparo ni kute mag Ayaki inyalo thiedhi?  Do you think that HIV can be cured? | O Yes  O No  O Don’t know  O Refused to answer |
| HIV09 | Iparo ni onego bedi ni ipimo kute mag Ayaki bang’ thuolo marom nadi?  How often do you believe you should test for HIV?  *Choose all that apply.* | O Every week  O Every month  O Every 3 months  O Every 6 months  O Every year  O When I have sex without a condom  O When I have a new partner  O When my primary partner informs me or I know that he was unfaithful  O During pregnancy  O Other: ________________________________  O If I have been tested once, I do not need to be tested again  O Don’t Know  O Refused to Answer |
| **Section E2: Sexual Testing History (TST)**  Interviewer: Daher penji penjo moko e wi pim mar kute mag Ayaki kod touché mag nyeye moko kaka syphilis, gonorrhea, chlamydia, Trichomonas vaginalis, or bacterial vaginosis.  Interviewer: I would now like to ask you some questions about testing for HIV and other sexually transmitted infections (STI) such as syphilis, gonorrhea, chlamydia, *Trichomonas vaginalis*, or bacterial vaginosis. | | |
| TST01 | Ka waweyo pim makawuononi, bende osega pimi kute mag ayaki?  Before the test you took today, had you ever been tested for HIV? | O Yes  O No (skip to TST03)  O Don’t Know (skip to TST03)  O Refused to Answer (skip to TST03) |
| TST02 | Ka waweyo pim ma kawuononi, en kar ang’o mane ipimoe kute mag Ayaki mogik?  Before the test today, how long ago was your last HIV test? | O < 3 Months  O 3-6 Months  O 7-12 Months  O 13-23 Months  O 2 Years or More  O Don’t Know  O Refused to Answer |
| TST03 | E kinde mar dweche 6 mokalo bende osepimi ma oyudi gi tuoché mag nyeye?  During the past 6 months, have you been diagnosed with a STI? | O Yes  O No (skip to TST05)  O Don’t Know (skip to TST05)  O Refused to answer (skip to TST05) |
| TST04 | En tuo mane mar nyach ma ne oyudigo?  Which STI(s) were you diagnosed with?  *Choose all that apply.*  *Interviewer: This is self-reported by the participant. If she does not remember the diagnosis, use “Don’t Know”. Do not try to diagnose her using symptoms she describes.* | O Trichomoniasis (Trich)  O Syphilis  O Gonorrhea  O Chlamydia  O Herpes  O Human papillomavirus (HPV)  O Genital warts  O Mycoplasma genitalium  O Bacterial vaginosis (BV)  O Other _______________________________  O Don’t Know  O Refused to answer |
| TST05 | Bende dine idhi kar uso yedhe ma inyiewo yath mar tuo mar nyach, ma ok idhi ir daktari kata ng’at moro amora man kod tiegruok mar chiwo kony mar ngima?  Did you ever go to a pharmacy and purchase treatment for an STI, without consulting a doctor or other qualified healthcare provider (ex: over the counter medication)? | O Yes  O No  O Don’t Know  O Refused to answer |
| TST06 | Bende dine iyudo thieth mar nyaluo kata thieth moro amora mar touché mag nyeye ma ok idhi ir daktari, ng’ama nigi tiegruok mar uso yedhe kata ng’at moro amora man kod tiegruok mar chiwo kony mar ngima?  Did you ever use a home remedy or treatment for an STI, without consulting a doctor, pharmacist or other qualified healthcare provider? | O Yes  O No  O Don’t Know  O Refused to answer |
| **Section E3: HIV Self-testing (HST)**  Interviewer: Kaka ne awacho ka ne pok wachako penjo, nonroni dwaro choko weche kuom rapim mar kute mag ayaki ma ng’ato pimorego kende en owuon. Pimo kute mag ayaki kendi iwuon tiende ni, ipimo kute mag ayaki saa ma berni kata kama berni, ma ok idhi e kar thieth moro amora. Pimni ok ti gi remo, to itiyogi olaw ma owuok e dhok. Penjo maluwegi gin mag pim mar kute mag ayaki ma ng’ato timo kende en owuon  Interviewer: As I mentioned before this interview, this study is interested in collecting information on HIV self-testing. HIV self-testing means you can test yourself for HIV at a time and location that is convenient for you, without having to go to a clinic. This test does not require a blood sample, but uses a sample from inside your mouth. The following questions are specific to HIV self-testing. | | |
| HST01 | Be isega winjo e wi pimo mar kute mag Ayaki ma ng’ato pimorego kende owuon e ndalo mokalo ma ok kawuono?  Have you ever heard of HIV self-testing before today? | O Yes  O No (skip to HST03)  O Don’t Know (skip to HST03)  O Refused to Answer |
| HST02 | Be isetiyoga kod gir pim mar kute mag Ayaki ma ng’ato pimorego kende owuon ka opogore gi kawuono?  Have you ever used an HIV self-test before today? | O Yes  O No  O Don’t Know  O Refused to Answer |
| HST03 | Ka nyalo bet ni gik pim mar kute mag Ayaki ma ng’ato pimorego kende owuon ne nyalo yudore, bende inyalo hero mondo ipimri Kute mag Ayaki?  If HIV self-testing were available to you, how interested would you be in testing yourself for HIV? | O Very Interested  O Somewhat Interested  O Neutral, Neither Interested nor Uninterested  O Somewhat Uninterested  O Very Uninterested  O Don’t Know  O Refused to Answer |
| HST04 | Ka bed ni gik pimo kute mag Ayaki ma ng’ato pimorego kende owuon ne nyalo yudore, iparo ni ne inyalo pimo kute mag ayaki ding’eny?  If HIV self-testing were available to you, do you think you would test for HIV more frequently? | O Yes, More Frequently  O No, Less Frequently  O No change in Frequency  O Don’t Know  O Refused to Answer |
| HST05 | Ka po ni iyudo duoko mar gir pim mar kute mag ayaki ma ng’ato pimorego kende owuon manyiso ni in kod kute mag Ayaki, iparo ni nyalore marom nadi mondo idhi inon duoko kuom jachiw kony mar ngima?  If you received a positive HIV self-test result, how likely do you think you would be to seek confirmatory testing with a healthcare provider? | O Very Likely  O Somewhat Likely  O Neutral, Neither Likely Nor Unlikely  O Somewhat Unlikely  O Very Unlikely  O Don’t Know  O Refused to Answer |
| HST06 | Nyalo bedoni mayot marom nadi mondo iter gir pim mar kute mag Ayaki ma ng’ato pimorego kende owuon ne jaherani mahie mondo otigo?  How comfortable would you be in taking an HIV self-test to give to your **primary** partner to use? | O Very Comfortable  O Somewhat Comfortable  O Neutral, Neither Comfortable Nor Uncomfortable  O Somewhat Uncomfortable  O Very Uncomfortable  O Don’t Know  O Refused to Answer |
| HST07 | Nyalo bedoni mayot marom nadi mondo iter gir pim mar kute mag Ayaki ma ng’ato pimorego kende owuon ne jaherani ma ok en mahie mondo otigo?  How comfortable would you be in taking an HIV self-test to give to your **non-primary** partner(s) to use? | O Very Comfortable  O Somewhat Comfortable  O Neutral, Neither Comfortable Nor Uncomfortable  O Somewhat Uncomfortable  O Very Uncomfortable  O Don’t Know  O Refused to Answer |

| **Section F: Gender-Based Violence (GBV)** | | |
| --- | --- | --- |
| Interviewer: Penjo maluwo gin kuom gik matimre e tudruoge moko, kod gima jaherani mahie kata joherani mamoko nyalo bedo ni osetimoni. Penjogi gin ewi tulo, manyalo miyo itho dich kata chunyi chandre. Ok ochuno ni nyaka iduok penjo moro amora mamiyo itho dich, kendo duokogi gin e yor maling’ ling’. Ka in kod penjo moro amora e saa moro amora, bed thuolo mar penjo. Bende ihikori mondo wadhi nyime?  Interviewer: The next questions are about things that happen within some relationships, and that your primary partner, or any other partners may have done to you. These questions are about violence, which may make you feel uncomfortable or distressed. You do not have to answer any question that makes you feel uncomfortable, and your responses are confidential. If you have any questions at any time, please ask me. Are you ready to continue? | | |
| GBV01:  E kinde mar dweche 12 mokalo, bende jaherani ma hie osetimo gik maluwogi ne in?  In the past 12 months has your **PRIMARY** sexual partner done any of the following to you?  *Interviewer: Read each option aloud one at a time and choose all that apply.* | GBV02:  E dweche 12 mokalo, be jaherani ma hie osega (insert coding category from GBV01) mang’eny koso seche moko?  In the past 12 months, has your **PRIMARY** partner done this often or only sometimes? | GBV03:  Be isega nyiso ng’at moro amora ni jaherani ma hie (insert coding category from GBV01), ka kamano en ng’a?  Did you ever tell anyone that your **PRIMARY** partner did this, and if so who did you tell? |
| O Yanyi kata miyo iwinj marach kuomi in iwuon  Insulted or made you feel bad about yourself | O Often  O Sometimes  O Don’t Know  O Refused to Answer | O Yes: ______________  O No  O Don’t Know  O Refused to Answer |
| O Okawi matin, kata onjawi onyisi achaye e nyim ji  Belittled or humiliated you in front of other people | O Often  O Sometimes  O Don’t Know  O Refused to Answer | O Yes: ______________  O No  O Don’t Know  O Refused to Answer |
| O Otimo gimoro mondo obuogi kata omiyi luoro kuom gimoro (Kaka ne ong’iyi, ka ogoni koko, kata otoyo gik moko gi mamoko.)  Done anything to scare or intimidate you on purpose (by the way he looked at you, by yelling, smashing things, etc.) | O Often  O Sometimes  O Don’t Know  O Refused to Answer | O Yes: ______________  O No  O Don’t Know  O Refused to Answer |
| O Obuogi ni onyalo hinyi kata hinyo ng’atmoro ma igeno.  Threatened to hurt you or someone you care about | O Often  O Sometimes  O Don’t Know  O Refused to Answer | O Yes: ______________  O No  O Don’t Know  O Refused to Answer |
| O Opami kata obai gi gimoro ma ne nyalo hinyi  Slapped, hit, or thrown something at you that could hurt you | O Often  O Sometimes  O Don’t Know  O Refused to Answer | O Yes: ______________  O No  O Don’t Know  O Refused to Answer |
| O Podhiri kata owiti oko  Pushed or shoved you | O Often  O Sometimes  O Don’t Know  O Refused to Answer | O Yes: ______________  O No  O Don’t Know  O Refused to Answer |
| O Ogweyi, oywayi piny kata ogoyi  Kicked, dragged, or beaten you | O Often  O Sometimes  O Don’t Know  O Refused to Answer | O Yes: ______________  O No  O Don’t Know  O Refused to Answer |
| O Odeyi kata owang’i nikech gimoroka ong’eyo  Strangled or burnt you on purpose | O Often  O Sometimes  O Don’t Know  O Refused to Answer | O Yes: ______________  O No  O Don’t Know  O Refused to Answer |
| O Obwogi kata otiyo kod bunde, pala kata gir lweny moro ma ne nyalo hinyi  Threatened or has actually used a gun, knife, or other weapon that could hurt you | O Often  O Sometimes  O Don’t Know  O Refused to Answer | O Yes: ______________  O No  O Don’t Know  O Refused to Answer |
| O Ne omaki, oywayi, orundi kata omuli e yoo ma ok owinjore ma omiyo ibedo ma onge kwe.  Fondled, groped, grabbed, or touched you in a way that was unwanted or made you feel unsafe | O Often  O Sometimes  O Don’t Know  O Refused to Answer | O Yes: ______________  O No  O Don’t Know  O Refused to Answer |
| O Ne obet e achiel e ringruok kodi kata ne otemo bedo e achiel e ringruok kodi kane ok inyal chiwo yieni nikech ne imer ahinya, ne imer matin, oketni gima meri, kata pachi ne olal  Forced you to have sex when you did not want to or could not provide consent (ex. you were too drunk or passed out) | O Often  O Sometimes  O Don’t Know  O Refused to Answer | O Yes: ______________  O No  O Don’t Know  O Refused to Answer |
| O None (skip to GBV04) | Not Applicable | Not Applicable |
| O Refused to Answer (skip to GBV04) | Not Applicable | Not Applicable |

| GBV04:  Ei dweche 12 mokalo, bende moro kuom jaherani ma ok mahie osetimo gik maluwogi ne in?  In the past 12 months has your **NON-PRIMARY** sexual partner done any of the following to you?  *Interviewer: Read each option aloud one at a time and choose all that apply.* | GBV05:  E dweche 12 mokalo, bende jaherani moro amora ma ok en mahie osega (insert coding category from Q73) mang’eny koso seche moko?  In the past 12 months, has your **NON-PRIMARY** partner done this often or only sometimes? | GBV06:  Bende isega nyiso ng’at moro amora ni jaherani ma ok mahie (insert coding category from Q70), ka kamano en ng’a?  Did you ever tell anyone that your **NON-PRIMARY** partner did this, and if so who did you tell? |
| --- | --- | --- |
| O Yanyi kata miyo iwinj marach kuomi in iwuon  Insulted or made you feel bad about yourself | O Often  O Sometimes  O Don’t Know  O Refused to Answer | O Yes: ______________  O No  O Don’t Know  O Refused to Answer |
| O Okawi matin, kata onjawi onyisi achaye e nyim ji  Belittled or humiliated you in front of other people | O Often  O Sometimes  O Don’t Know  O Refused to Answer | O Yes: ______________  O No  O Don’t Know  O Refused to Answer |
| O Otimo gimoro mondo obuogi kata omiyi luoro kuom gimoro (Kaka ne ong’iyi, ka ogoni koko, kata otoyo gik moko gi mamoko.)  Done anything to scare or intimidate you on purpose (by the way he looked at you, by yelling, smashing things, etc.) | O Often  O Sometimes  O Don’t Know  O Refused to Answer | O Yes: ______________  O No  O Don’t Know  O Refused to Answer |
| O Obuogi ni onyalo hinyi kata hinyo ng’atmoro ma igeno.  Threatened to hurt you or someone you care about | O Often  O Sometimes  O Don’t Know  O Refused to Answer | O Yes: ______________  O No  O Don’t Know  O Refused to Answer |
| O Opami kata obai gi gimoro ma ne nyalo hinyi  Slapped, hit, or thrown something at you that could hurt you | O Often  O Sometimes  O Don’t Know  O Refused to Answer | O Yes: ______________  O No  O Don’t Know  O Refused to Answer |
| O Podhiri kata owiti oko  Pushed or shoved you | O Often  O Sometimes  O Don’t Know  O Refused to Answer | O Yes: ______________  O No  O Don’t Know  O Refused to Answer |
| O Ogweyi, oywayi piny kata ogoyi  Kicked, dragged, or beaten you | O Often  O Sometimes  O Don’t Know  O Refused to Answer | O Yes: ______________  O No  O Don’t Know  O Refused to Answer |
| O Odeyi kata owang’i nikech gimoroka ong’eyo  Strangled or burnt you on purpose | O Often  O Sometimes  O Don’t Know  O Refused to Answer | O Yes: ______________  O No  O Don’t Know  O Refused to Answer |
| O Obwogi kata otiyo kod bunde, pala kata gir lweny moro ma ne nyalo hinyi  Threatened or has actually used a gun, knife, or other weapon that could hurt you | O Often  O Sometimes  O Don’t Know  O Refused to Answer | O Yes: ______________  O No  O Don’t Know  O Refused to Answer |
| O Ne omaki, oywayi, orundi kata omuli e yoo ma ok owinjore ma omiyo ibedo ma onge kwe.  Fondled, groped, grabbed, or touched you in a way that was unwanted or made you feel unsafe | O Often  O Sometimes  O Don’t Know  O Refused to Answer | O Yes: ______________  O No  O Don’t Know  O Refused to Answer |
| O Ne obet e achiel e ringruok kodi kata ne otemo bedo e achiel e ringruok kodi kane ok inyal chiwo yieni nikech ne imer ahinya, ne imer matin, oketni gima meri, kata pachi ne olal  Forced you to have sex when you did not want to or could not provide consent (ex. you were too drunk or passed out) | O Often  O Sometimes  O Don’t Know  O Refused to Answer | O Yes: ______________  O No  O Don’t Know  O Refused to Answer |
| O None (skip to GBV04) | Not Applicable | Not Applicable |
| O Refused to Answer (skip to GBV04) | Not Applicable | Not Applicable |

| **NOTES** |
| --- |
|  |

**END OF BASELINE QUESTIONNAIRE**
