# Supplementary material for: Adoption of HIV pre-exposure prophylaxis among women at high risk of HIV infection in Kenya
Source: PLoS One. 2022 Sep 9;17(9):e0273409. doi: 10.1371/journal.pone.0273409 (PMC9462728; doi:10.1371/journal.pone.0273409)
Supplement: S3 File — (DOCX) [file pone.0273409.s003.docx]

Baseline Visit Questionnaire v7.2_Kiswahili

| **No.** | **Question** | | | **Responses** | | | |  |
| --- | --- | --- | --- | --- | --- | --- | --- | --- |
| 1 | Interviewer ID | | | ___ ___ | | | |  |
| 2 | Date of Screening | | | ___ ___/___ ___ ___/ ___ ___ ___ ___  D D M M M Y Y Y Y | | | |  |
| 3 | Enter Cluster ID | | | ___ ___ ___ | | | |  |
| 5 | Enter Name of Cluster | | |  | | | |  |
| 6 | Enter Name of Hotspot or Beach | | |  | | | |  |
| **Verbal Screening**  Read the Verbal Screening document to the participant and obtain consent to screen. | | | | | | | |  |
| 7 | Did the woman give verbal consent to participate in the screening process? | | | O Yes(skip to SCR1)  O No (answer #8 then skip to END) | | | |  |
| 8 | If the woman gave a reason why she did not want to be verbally consented, please indicate.  *Choose all that apply* | | | O No reason given or disclosed  O Woman did not have time/want to wait  O Woman did not want to answer screening questions  O Woman did not want to take an HIV test  O Woman needed permission from partner  O Other: __________________________________________  __________________________________________________ | | | |  |
| **Section 1: Screening Questions (SCR)**  Tell the participant you are going to ask her some questions to determine if she will be enrolled in the study. | | | | | | | |  |
| SCR1 | Una umri wa miaka mingapi?  What is your age?  *Interviewer: If participant does not know, ask for best guess, using schooling or other milestones to assist if necessary. Only after unsuccessful probing should you select response “Don’t Know”.* | | | | ___ ___ years (if less than 18, verify with SCR2)  O Don’t Know  O Refused to Answer | | |  |
| SCR2 | Je, ulizaliwa mwaka gani?  In what year were you born?  *Interviewer: Probe if inconsistent with age. If participant guessed age and doesn’t know birth year, use the estimated age to calculate birth year.* | | | | ___ ___ ___ ___  Should match age given in SCR1;  If verified <18 years by SCR1 and SCR2, then skip to END | | |  |
| SCR3 | Je, unaishi wapi?  Where do you live?  *Interviewer: determine if given locations are within the study region. Consult Study Coordinator for clarification if necessary.* | | | | O Within Study Region  O Outside Study Region (skip to END)  O Migratory (skip to END)  O Don’t Know (skip to END)  O Refused to Answer (skip to END) | | |  |
| SCR4 | Je, umeishi eneo hili kwa takriban miezi sita kati ya miezi kumi na mbili lililopita?  Have you lived in this area at least 6 out of the last 12 months?  *Interviewer: Use the confines of the study area to determine based on participant’s response.* | | | | O Yes  O No (skip to END)  O Don’t Know (skip to END)  O Refused to Answer (skip to END) | | |  |
| SCR5 | Je, una mpango ya kuishi katika eneo hili (Eneo la utafiti) kwa muda wa takriban miezi 24 zijaazo au miaka 2?  Do you plan to reside in the local area (study region) for the next 24 months or 2 years?  *Interviewer: A response of “Don’t Know” requires probing – only* ***definitive*** *plans to relocate should be used to exclude.* | | | | O Yes  O No (skip to END)  O Don’t Know  O Refused to Answer (skip to END) | | |  |
| SCR6 | Je kwa muda wa wiki 4 zilizopita, ushakuwa na washiriki wa ngono 2 au zaidi?  In the past 4 weeks, have you had two or more sexual partners?  *Interviewe*r: *Remember to introduce this question in a way that will not offend the participant.Only after unsuccessful probing should you select response “Don’t Know”.* | | | | O Yes  O No (skip to END)  O Don’t Know (skip to END)  O Refused to Answer (skip to END) | | |  |
| SCR7 | Je, unayo simu yako binafsi ya rununu au simu ya mtu ya mtu mwingine unayoweza fikia mara kwa mara?  Do you have your own mobile phone or regular access to someone else’s mobile phone? | | | | O Yes  O No (skip to END)  O Don’t Know (skip to END)  O Refused to Answer (skip to END) | | |  |
| SCR8 | Je, kwa wakati huu umashiriki kwenye utafiti mwingine inayozuia uambukizaji wa virusi vya ukimwi na shirika hili au shirika yoyote mwingine?  Are you currently enrolled in another HIV prevention study with this or any other organization? | | | | O Yes (skip to END)  O No  O Don’t Know (skip to END)  O Refused to Answer (skip to END) | | |  |
| **Section 2: HIV Rapid Test and DBS Collection**  For participants who are eligible to this point, perform HIV antibody rapid testing and enter results. Collect DBS card. | | | | | | | |  |
| SCR9 | What was the result of the HIV rapid test?  *Interviewer: If the HIV result is positive or indeterminate, ensure client receives any necessary confirmatory testing, proper counseling, and linkage to care.* | | | | O Negative (skip to SCR11)  O Positive (skip to SCR10)  O Indeterminate (skip to SCR10)  O Woman refused testing (skip to END) | | |  |
| SCR10 | Did you provide appropriate confirmatory testing, counseling, and linkage to care? | | | | O Yes  O No | | |  |
| **Section 3: Main Study Consent**  For eligible participants, read and obtain informed consent for the main study. | | | | | | | |  |
| SCR11 | After reviewing the informed consent document and asking questions, did the participant consent to participate in the main study? | | | | O Yes  O No (skip to END) | | |  |
| **END** | *Interviewer:* Asante kwa muda wako na kuwa na nia ya kushiriki kwenye mkondo wa mchujo ya utafiti yetu. Kwa wakati huu, ingawapo, hauta andikishwa kama mshiriki kwenye utafiti huu. Hii haimanishi hauwezi shiriki utafiti zingine zinazoendelea kwa wakati huu au siku zijaazo.Ukiwa una swali yoyote , nitafurahia kuyajibu wakati huu.  Thank you for your time and willingness to participate in the screening process for our study. At this time, however, you will not be enrolled as a participant in this study. This does not mean that you cannot participate in other research studies that are happening now or in the future. If you have any questions, I will be happy to answer them now. | | | | | | |  |
| **Section 4: Participant Identification** | | | | | | | | |
| PID_GEN | | Does a new PID need to be generated for this participant?  Note: Only select “Yes” if this is the first time this form is being completed for this participant. If there was a tablet error and you are re-entering data from another tablet, select “No” and enter the PID that was generated initially. | | | | | O Yes (skip to DEM01, ODK to generate and display ID)  O No | |
| pid | | Enter the participant’s ID number. Be extremely careful when entering the number. Double and triple check that the number is correct before proceeding. | | | | | ___ ___ ___ ___ ___ ___ ___ ___ ___ | |
| pid_verify | | Verify PID number | | | | | ___ ___ ___ ___ ___ ___ ___ ___ ___ | |
| **Section 5: Baseline Questionnaire**  Screening form now complete. The Baseline survey starts here. | | | | | | | | |
| **Section B: Demographic Information (DEM)** | | | | | | | | |
| **No.** | | | **Question** | | | **Coding** | | |
| DEM01 | | | Una umri wa miaka mingapi?  What is your age? | | | ___ ___  Verify with SCR1 | | |
| DEM02 | | | Kiwango cha juu cha masomo uliyokamilisha ni gani?  What is the highest level of school you have *completed*?  *Interviewer: if DEM02=98, probe* | | | O None  O Some Primary  O Primary  O Some Secondary  O Secondary/ High School  O Post-Secondary/Training College  O University  O Don’t Know  O Refused to Answer | | |
| DEM03 | | | Hali yako ya ndoa kwa wakati huu ni upi?  What is your current marital status? | | | O Married, Living Together  O Married, NOT Living Together  O Not Married, Living Together  O Relationship but Not Married, NOT Living Together (boyfriend, etc.)  O Single  O Divorced  O Widowed  O Don’t Know  O Refused to Answer | | |
| DEM04 | | | Kundi lako la kikabila/kabila ni lipi?  What is your ethnic group? | | | O Luo  O Luhya  O Kalenjin  O Kikuyu  O Kisii  O Ugandan  O Other: _______________________  O Don’t Know  O Refused to Answer | | |
| DEM05 | | | Njia yako kuu ya mapato ni ipi?  What is your **primary** source of income? | | | O Professional/salaried  O Rental income (landlord, rent equipment)  O Sales and Service (Non-Fish)  O Skilled Manual  O Unskilled Manual  O Domestic Service  O Agriculture  O Fishing/ Fish Trade  O Sex Work  O Informal/Seasonal/ Piece Work  O Student  O Unemployed  O Other: ________________________________  _______________________________________  O Don’t Know  O Refused to Answer | | |
| DEM06 | | | Njia yako nyingine ∕zako zingine za mapato ni zipi? Kama ziko?  What is/are your other source(s) of income, if any?  *Choose all that apply* | | | O Professional/salaried  O Rental income (landlord, rent equipment)  O Sales and Service (Non-Fish)  O Skilled Manual  O Unskilled Manual  O Domestic Service  O Agriculture  O Fishing/ Fish Trade  O Sex Work  O Informal/Seasonal/ Piece Work  O Student  O Other: _______________________________  ______________________________________  O None/ Not Applicable  O Don’t Know | | |
| DEM07 | | | Je,mapato yako kwa jumla ni ngapi unapata kwa mwezi moja, kutokana na njia zako zote za mapato ?  How much income do you typically earn in one month, from all sources?  *Interviewer: Ask participant to give best estimate if not sure* | | | KES ___________________  O Don’t Know  O Refused to Answer | | |
| DEM08 | | | Je, ni watu wangapi wanaoishi kwa nyumba yako, pamoja na wewe?  How many people live in your household, including yourself? | | | _______  O Refused to Answer | | |
| DEM09 | | | Je, ni watu wangapi wanaoishi kwa nyumba yako wanaopata mapato ya kila mara /Mshahara, pamoja na wewe?  How many people in your household have a regular or steady income, including yourself? | | | _______  O Don’t Know  O Refused to Answer  *Must be less than or equal to DEM08.* | | |
| DEM10 | | | Ni watoto wangapi kwa sasa wanaokutegemea kwa chakula, mahali pa kuishi, pesa na kadhalika)?  How many people do you currently support (provide with food, housing, money, etc.)?  *Interviewer: This does not have to be a member of your household.* | | | _______  O Don’t Know  O Refused to Answer | | |
| DEM11 | | | Kwa mwezi uliopita, je umeweza kupata chakula mara mbili kwa siku?  In the past month, have you regularly eaten at least two meals a day? | | | O Yes  O No  O Don’t Know  O Refused to Answer | | |

| **Section C: Health and Sexual Behavior** | | |
| --- | --- | --- |
| **Section C1 – General Health (HLT)**  Interviewer: Nikon a maswali machache kuhusu afya yako ya kimwili na fikira  I have some questions about your physical and mental health. | | |
| **No.** | **Question** | **Coding** |
| HLT01 | Je unaweza sema afya yako kwa ujumla iko vipi?  How would you rate your overall health? | O Vizuri sana  O Vizuri  O Si mbaya/Si nzuri  O Mbaya  O Hajui  O Amekataa Kujibu |
| HLT02 | Je, kwa miezi 12 iliyopita unaweza kadiria umekunywa pombe mara ngapi?  In the past 12 months, what is your best estimate of how often you drank alcohol? | O Never (skip to HLT04)  O Less than once per month  O Once a month  O 2 to 3 times per month  O Once per week  O Twice per week  O 3 to 4 times a week  O 5 to 6 times per week  O Every Day  O Don’t Know  O Refused to Answer |
| HLT03 | Je kwa mwezi moja uliyopita, ni mara ngapi ulikunywa vinywaji vya kulewesha chupa 5 au zaidi kwa usiku mmoja?  In the past month, how often did you drink 5 or more alcoholic drinks in one night? | O Never  O Only once  O 2 or 3 times  O Once per week  O Twice per week  O 3 to 4 times a week  O 5 to 6 times per week  O Every Day  O Don’t Know  O Refused to Answer |

| HLT04:  Kwa wiki 2 zilizopita, je, ushawai sumbuliwa na shida zifuatazo?  Over the last 2 weeks, have you been bothered by any of the following problems?  *Interviewer: Read each option aloud one at a time.* | HLT 05:  Kwa wiki 2 zilizopita, je, ushawai sumbuliwa na shida zifuatazo?  Over the last 2 weeks, how often have you been bothered by this problem? |
| --- | --- |
| O Kutokua na hamu ya kufanya kazi.  Had little interest or pleasure in doing things | O One Day  O Less than Half the Days  O Around Half the Days  O More than Half the Days  O Every Day  O Don’t Know  O Refused to Answer |
| O Kujihisi uko chini, kuhuzunika, au kukosa matumaini  Felt down, depressed, or hopeless | O One Day  O Less than Half the Days  O Around Half the Days  O More than Half the Days  O Every Day  O Don’t Know  O Refused to Answer |
| O Kua na shida kulala aukulala sana  Had trouble falling asleep/ staying asleep, or sleeping too much | O One Day  O Less than Half the Days  O Around Half the Days  O More than Half the Days  O Every Day  O Don’t Know  O Refused to Answer |
| O Kujisikia mchovu au kuwa na nguvu kidogo.  Felt tired or having little energy | O One Day  O Less than Half the Days  O Around Half the Days  O More than Half the Days  O Every Day  O Don’t Know  O Refused to Answer |
| O Kutokua na hamu wa kula chakula au kula zaidi.  Had poor appetite or overeating | O One Day  O Less than Half the Days  O Around Half the Days  O More than Half the Days  O Every Day  O Don’t Know  O Refused to Answer |
| O Kuhisi vibaya juu ya nafsi yako mwenyewe ama kuwa haujafikisha matarajio yako kwa familia yako.  Felt bad about yourself, felt that you are a failure, or felt that you let yourself or your family down | O One Day  O Less than Half the Days  O Around Half the Days  O More than Half the Days  O Every Day  O Don’t Know  O Refused to Answer |
| O Kuwa na shida kusoma gazeti au kutazama runinga kwa umakini  Had trouble concentrating on things such as reading the newspaper or watching television | O One Day  O Less than Half the Days  O Around Half the Days  O More than Half the Days  O Every Day  O Don’t Know  O Refused to Answer |
| O Kutembea au kuongea kwa upole mpaka watu wengine wanajua au wanaweza kutambua na kuongea mingi kuliko kawaida mpaka watu wanajua hauko sawa  Moved or spoken so slowly that other people could have noticed, or been so fidgety/restless that you have moved around a lot more than usual | O One Day  O Less than Half the Days  O Around Half the Days  O More than Half the Days  O Every Day  O Don’t Know  O Refused to Answer |
| O Kuwa na fikira kwamba heri ufe au kujiumiza kwa njia yoyote.  Had thoughts that you would be better off dead, or of hurting yourself in some way | O One Day  O Less than Half the Days  O Around Half the Days  O More than Half the Days  O Every Day  O Don’t Know  O Refused to Answer |
| O None (skip to GSA01) | Not Applicable |
| O Refused to Answer (skip to GSA01) | Not Applicable |

| HLT06 | | Je shida hizi zimekuzuia aje kufanya kazi zako, kufanya vitu za nyumbani ama uhusiano wako na watu wengine?  How difficult have these problems made it for you to do your work, take care of things at home, or get along with other people? | | O Not Difficult at All  O Somewhat Difficult  O Very Difficult  O Don’t Know  O Refused to Answer |
| --- | --- | --- | --- | --- |
| **Section C2: General Sexual Activity (GSA)**  Interviewer: Kwa sasa niko na maswali kadhaa kuhusu ushiriki wako wa ngono, ili nipate kuelewa vizuri maneno kadhaa muhimu ya maisha yako. Kwa maswali haya, “kushiriki ngono” inafafanuliwa kama ngono kupitia sehemu ya mwanamke ya siri au sehemu ya haja kubwa.  Now I have some questions about your sexual activity, in order to get a better understanding of some important aspects of your life. For these questions, “sexual activity” is defined as sexual penetration of the vagina or anus. | | | | |
| GSA01 | | Je, ulikuwa na umri wa miaka mingapi uliposhiriki ngono kwa mara ya kwanza?  How old were you when you had sex for the first time?  *Interviewer: If participant doesn’t know exact age, probe for an estimate based on schooling or other life events.* | | ___ ___ years  O Don’t Know or Cannot Estimate  O Refused to Answer |
| GSA02 | | Mara ya kwanza uliposhiriki ngono, je mpenzi wako alikulazimisha katika njia yoyote ya ngono pasipo idhini yako?  The first time you had sex, did your partner force or coerce you into any sex act against your will? | | O Yes  O No  O Don’t Know  O Refused to Answer |
| GSA03 | | Kwa mwezi uliopita umekuwa na wapenzi wangapi tofauti wa kushiriki ngono?  During the past month, how many different sexual partners have you had? | | ___ ___  O Don’t Know  O Refused to Answer  *Interviewer: Probe if less than 2 – this is part of eligibility* |
| GSA04 | | Je mara ya mwisho uliposhiriki ngono, ulitumia mpira wa condomu?  The last time you had sex, did you use a condom? | | O Yes  O No  O Don’t Know  O Refused to Answer |
| GSA05 | | Katika mwezi moja uliopita ni mara ngapi ulitumia njia ya upangaji uzazi , tofauti na mpira wa kondomu kuzuia mimba?  During the past month, how often did you use a contraceptive device, other than the male condom, to prevent pregnancy? | | O Never (skip to PRP01)  O Less than Half the Time  O About Half the Time  O More than Half the Time  O Don’t Know  O Refused to Answer |
| GSA06 | | Katika mwezi moja uliyopita ni njia zipi zifuatazo za upangaji uzazi uliyotumiakuzuia mimba mbali na mpira wa kondomu ya wanaume?  In the past month, which of the following contraceptives, other than the male condom, have you used to prevent pregnancy? | | O Pill  O Hormone injection (Depo-Provera)  O Intrauterine device (IUD)  O Diaphragm  O Patch  O Implant (Norplant, Jadelle)  O Emergency pill (plan B or morning after pill)  O Spermicide  O Female condom  O Withdrawal  O Other:____________________________________  O Don’t Know  O Refused to Answer |
| **Section C3: Primary Partner (PRP)**  Interviewer: Kwa sasa niko na maswali kuhusu mshiriki wako mmoja wa karibu na wa mara kwa mara wa ngono. Mshiriki wa karibu anaweza huenda akawa mumeo , mpenzi wako au mtu unayeshiriki naye mara kwa mara kwa njia ya ngono. Mshiriki wa karibu ni Yule mshiriki unahisi unampenda au kuwa naye karibu zaidi au unayeweza muelezea mambo yako ya kindani zaidi. Ikiwa unahitaji usaidizi kuamua ni nani mshiriki wako wa karibu , tunaweza zungumzia hiyo wakati huu.  Now I have some questions about your **one** primary or regular sexual partner. A primary partner could be your husband, boyfriend, or someone you **regularly** have sex with. A primary partner is the ONE partner you feel like you love or like the most, or the one you can confide in the most. If you would like help deciding who your primary partner is, we can talk about that now. | | | | |
| PRP01 | | Je una mpenzi mkuu wa ngono au umewahi kuwa na mmoja katika mwezi mojo uliyopita?  Do you currently have a primary partner, or have you had one in the past month?  *This is the man we will discuss in the following questions.* | | O Yes  O No (skip to NPP01)  O Refused to Answer |
| PRP02 | | Je ni mara ngapi umetumia condomu na mshiriki wako mkuu kataika mwezi moja uliyopita?  In the past month, how often did you use condoms with your primary partner? | | O Never  O Less than Half the Time  O About Half the Time  O More than Half of the Time  O Always (skip to PRP05)  O Don’t Know  O Refused to Answer |
| PRP03 | | Je ni kwa sababu gani haukutumia mpira wa kondomu na mpenzi wako mkuu kwa mwezi mmoja uliyopita?  Why did you not use condoms all the time with your primary partner within the last month?  *Interviewer choose all that apply based on what the participant mentioned.* | | O I Did Not Want To  O Partner Did Not Want To  O No Condom was Available  O I Am Trying To Become Pregnant  O I Have Another Form of Contraception (IUD, Implant, Injectable, Pill, etc.)  O I Knew My Primary Partner’s HIV Status and decided a condom was not necessary  O I trust my primary partner  O Other: ____________________________________  ___________________________________________  O Don’t Know  O Refused to Answer |
| PRP04 | | Je kwa mwezi mmoja uliopita, umekuwa na ugumu wakujadili /kushauriana juu ya matumizi ya mpira wa kondomu na mshiriki wako mkuu?  In the past month, have you ever had difficulty *negotiating* condom use with your primary partner? | | O Yes, always  O Yes, sometimes  O I have no difficulty negotiating  O I never try to get my partner to use condoms  O Don’t Know  O Refused to Answer |
| PRP05 | | Je katika miezi 12 zilizopita, mpenzi wako mkuu amewahi enda kupimwa virusi vya ukimwi?  In the past 12 months, has your primary partner gone for an HIV test? | | O Yes  O No  O Don’t Know  O Refused to Answer |
| PRP06 | | Je, wajua hali ya virusi vya ukimwi ya mpenzi wako mkuu kwa sasa?  Do you know your current primary partner’s HIV status? | | O No  O Yes, Partner is Positive  O Yes, Partner is Negative  O Yes, Refuse to Disclose Partner’s Status  O Refused to Answer |
| PRP07 | | Je mpenzi wako mkuu wa sasa ana miaka ngapi?  About how old is your current primary partner? (participant to give best guess) | | ___ ___ years  O Don’t Know  O Refused to Answer |
| PRP08 | | Je umekuwa na uhusiano wa kimapenzi na mshiriki wako mkuu wa sasa kwa muda gani?  How long have you had a sexual relationship with your current primary partner?  *Interviewer: For relationships <1 month, record 01 months. (Ex: If participant says one week, record 01months)* | | ___ ___ years, ___ ___ months  O Don’t Know  O Refused to Answer |
| **Section C4: Non-Primary Partners (NPP)**  Interviewer: Kwa sasa niko na maswali kadhaa kuhusu mshiriki wako yoyote mwingine ambaye ulikuwa naye isipokuwa mshiriki wako wa karibu. Kwa maswali haya, namaanisha mpenzi wako, washiriki wa ngono usio na usiano wowote au mtu unayeshiriki ngono ya kufaidika ,i.e pesa kwa ngono , bidhaa, zawadi, chakula au nyumba.  Now I have some questions about any other sexual partners you may have had other than your primary partner. For these questions, I am referring to boyfriends, casual sexual partners, or someone with whom you engage in transactional sex, i.e. sex for money, goods gifts, food or housing. | | | | |
| NPP01 | Wateja au wengine Je katika mwezi mmoja uliyopita umekuwa na washirik wangapi wasio washiriki wakuu wa ngono?  How many clients (Don’t say clients if not a known FSW) non-primary sexual partners have you had in the past month?  *Interviewer: probe for an estimate if participant cannot immediately recall* | | | ___ ___ (If 00, skip to TRX01)  O Don’t Know  O Refused to Answer |
| NPP02 | Je kati ya hawa , ni kadri wangapi ulishiriki nao ngono **mara kadhaa kwa miezi mmoja uliyopita?**  Of these, approximately how many did you have **multiple** sexual encounters with in the past month? | | | ___ ___ (If 00, skip to NPP05)  O Don’t Know  O Refused to Answer |
| NPP03 | Je ni mara ngapi ulitumia mpira wa kondomu kwa wale ambao ulishiriki nao ngono kwa mara kadhaa kwa mwezi moja uliyopita?  Among those with whom you had multiple sexual encountersin the past month how often did you use condoms? | | | O Never  O Less than Half the Time  O About Half the Time  O More than Half of the Time  O Always (skip to NPP05)  O Don’t Know  O Refused to Answer |
| NPP04 | Je ni kwa nini haukutumia mpira wa kondomu kila mara na wale ulioshiriki nao ngono kwa mara kadhaa?  Why did you not always use condoms with those you shared multiple sexual encounters?  *Open ended for participants. Interviewer choose all that apply based on what the participant mentioned.* | | | O I Did Not Want To  O Partner(s) Did Not Want To  O No Condom Was Available  O I Am Trying to Become Pregnant  O I Have Another Form of Contraception (IUD, Implant, Injectable, Pill, etc.)  O I Knew My Non-Primary Partner’s HIV Status  O I trust my non-primary partner(s)  O Partner offered me more money to not use a condom  O Other: _________________________  _________________________________  O Don’t Know  O Refused to Answer |
| NPP05 | Je, ni kadri washiriki wangapi wasio washiriki wakuu wa ngono ulishiriki nao ngono mara**moja tu** kwa mwezi mmoja uliopita?  Approximately how many (FSW only: clients or other) non-primary sexual partners in the past month did you have sex with **only once**? | | | ___ ___ (If 00, skip to TRX01)  O Don’t Know  O Refused to Answer |
| NPP06 | Je,kati ya wanaume ambao ulishiriki nao ngono mara moja tu kwa mwezi, ni mara ngapi ulitumia kondomu?  Among the men with whom you had only one sexual encounterin the past month, how often did you use condoms? | | | O Never  O Less than Half the Time  O About Half the Time  O More than Half of the Time  O Always (skip to TRX01)  O Don’t Know  O Refused to Answer |
| NPP07 | Je, mbona haukutumia kondomu kila wakati na wale ambao ulishiriki nao ngono mara moja tu?  Why did you not always use condoms with those you had only one sexual encounter?  *Open ended for participants. Interviewer choose all that apply based on what participant mentioned.* | | | O I Did Not Want To  O Partner(s) Did Not Want To  O No Condom Was Available  O I Am Trying to Become Pregnant  O I Have Another Form of Contraception (IUD, Implant, Injectable, Pill, etc.)  O I Knew My Non-Primary Partner’s HIV Status  O I trust my non-primary partner(s)  O Partner offered me more money to not use a condom  O Other: _________________________  _________________________________  O Don’t Know  O Refused to Answer |
| **Section C5: Transactional Sex (TRX)**  Interviewer: Kwa sasa ningependa kuuliza maswali kuhusu mshiriki wa ngono wowote ina uwezekano ulikuwa naye aliyekulipa kwa kushiriki ngono au alikupa chochote kwa kushiriki ngono. Wanaweza kuwa wanaume ambayo ulishiriki ngono mara moja tu au wanaume uliyeshiriki ngono mara kadhaa. Baadhi ya maswali haya yanaweza fanana nay ale niliyouliza hapo awali lakini fikiria juu ya wakati ulishiriki ngono kwa kitu fulani peke yake.  I would now like to ask questions about any sexual partners you may have had who paid you for sex or gave you something in exchange for sex. These may be men with whom you had only one sexual encounter, or men with whom you had multiple sexual encounters. Some of these questions may sound similar to ones I just asked, but please think only about those encounters where you exchanged sex. | | | | |
| TRX01 | | Je umeshawahi badilisha ngono kwa pesa, bidhaa, zawadi, chakula, makaazi, (malipo ya: stima,maji nyumba nkt) huduma au kushawishi?  Have you ever exchanged sex for money, goods, gifts, food, housing, services, or influence? | | O Yes  O No  O Refused to Answer |
| TRX02 | | Je, ulikuwa wa umri gani mara ya kwanza ulipo badilisha ngono kwa pesa, zawadi, chakula, makaazi, au huduma?  How old were you when you **first** exchanged sex for money, gifts, goods, food, housing or services?  *Interviewer: If participant doesn’t know, probe using life events such as schooling.* | | ___ ___ years *(Must be greater than or equal to GSA01)*  O Don’t Know  O Refused to Answer |
| TRX03 | | Je, kwa miezi 12 iliyopita, umebadilisa ngono kwa pesa, zawadi, makaazi au kitu chochote **mara kwa mara** au **kwa kurudia rudia**?  In the past 12 months, have you **regularlyor repeatedly** exchanged sex for money, goods, food, housing or services? | | O Yes  O No  O Refused to Answer |
| TRX04 | | Je, kwa mwezi mmoja uliyopita, ni kadri wanaume wangapi ambao ulibadilisha nao ngono?  In the past month, with approximately how many men did you exchange sex? | | _________ (If 0, skip to TRX06)  O Don’t Know  O Refused to Answer |
| TRX05 | | Je,kati ya hawa wanaume ulioshiriki nao ngono kwa mwezi mmoja uliopita, ni wangapi ulishiriki nao ngono zaidi ya mara moja?  Of these men in the past month with whom you exchanged sex, with how many did you have **multiple** sexual encounters? | | _________ *(Must be less than or equal to TRX04)*  O Don’t Know  O Refused to Answer |
| TRX06 | | Je, kwa miezi 3 iliyopita, umebadilisha ngono kwa pesa, bidhaa, chochote, au ushawishi katika eneo mbali na________(*Interviewer: insert name of cluster area*)?  In the last 3 months, have you exchanged sex for money, goods, services, or influence in a location other than ____? | | O Yes  O No (skip to TRX08)  O Refused to Answer (skip to TRX08) |
| TRX07 | | Tafadhali taja mahala pengine ambapo umewahi shiriki ngono kwa pesa,bidhaa, huduma au umaarufu kwa miezi 3 zilizopita.  Please list the names of the other places or areas where you have exchanged sex for money, goods, services, or influence in the last 3 months.  *Interviewer: Use the cluster code for each area. See list for codes. List the most recent 5.* | | 1: ___ ___ ___  2: ___ ___ ___  3: ___ ___ ___  4: ___ ___ ___  5: ___ ___ ___ |
| TRX08 | | Je, kwa mwezi mmoja uliyopita ume badilisha ngono kwa pesa?  In the past month, have you exchanged sex for **money**? | | O Yes (skip to TRX13)  O No  O Refused to Answer |
| TRX09 | | *Ask only if client reported sex work as primary OR secondary income (DEM05 OR DEM06)*  Je, kwa mwezi mmoja uliyopita, ilibidi umlipe mtu yeyote kama vile, meneja, administreta,pimp, au ugawe na mtu asilimia ya pesa uliyopata kwa kubadilisha ngono?  During the past month, did you have to pay someone like a manager, administrator or pimp, or share with someone a percentage of the money that you received for sex? | | O Yes  O No  O Refused to Answer |
| TRX10 | | Je,kwa kawaida ni kiasi kipi cha mapato yako hutokana kwa kubadilisha ngono kwa pesa?  In a typical month, how much of your income comes from exchanging sex for money? | | O None  O Less than half  O About half  O More than half, but not all  O All  O Don’t Know  O Refused to Answer |
| TRX11 | | Je unapobadilisha ngono kwa pesa, ni takriban pesa ngapi unalipisha kwa kila wakati umeshiriki ngono ikiwa mpira wa kondomu **umetumika**?  When you exchange sex for money, what is the average amount of money you charge per sexual encounter when a condom **is** used? | | KES _________  O I Never Use a Condom  O Don’t Know  O Refused to Answer |
| TRX12 | | Je unapobadilisha ngono kwa pesa, ni takriban pesa ngapi unalipisha kwa kila wakati umeshiriki ngono ikiwa mpira wa kondomu **umetumika**?  When you exchange sex for money, what is the average amount of money per sexual encounter when a condom**isnot** used? | | KES _________  O I Always Use a Condom  O Don’t Know  O Refused to Answer |
| TRX13 | | Je katika mwezi mmoja uliyopita ushawahi badilisha ngono kwa bidhaa au huduma **mbali na pesa**?  In the past month, have you exchanged sex for goods or services **other than** money? | | O Yes  O No (skip to TRX16)  O Refused to Answer (skip to TRX16) |
| TRX14 | | Je katika mwezi mmoja uliyopita ni bidhaaau huduma zipi ulibadilisha kwa ngono?  In the past month, what goods or services have you exchanged for sex?  *Choose all that apply.* | | O Housing and/or utilities  O Food to eat  O Food to sell (example, fish)  O School fees  O To get a job, a work promotion, or to keep your job  O Other material goods (clothes, jewelry, makeup, electronics, etc.)  O Household items (soap, cleaning supplies, tools, etc.)  O Other (specify): ____________________________  O Don’t Know  O Refused to Answer |
| TRX15 | | *Ask only if TRX08 and/or TRX13 = Yes*  Je kwa mwezi mmoja uliyopita, ni takriban pesa ngapi kwa ujumla , bidhaa, au huduma uliopata ukibadilisha kwa ngono?  In the past month, what is the approximate **total***value* of the money, goods, or services you received in exchange for sex?  *Interviewer: Make sure that participant understands to include how much the non-monetary goods/services are worth.* | | KES ___________  O Don’t Know  O Refused to Answer |
| TRX16 | | Je umewahi kukataa kushiriki ngono ili upate chochote ulichokitaka, na kama ni hivyo, ilikuwa na mshiriki wako mkuu wa ngono mkuu au mshiriki wako asiye mkuu*?*  Have you ever withheld sex in order to get something that you wanted? | | O Yes  O No (skip to SMH01)  O Don’t Know (skip to SMH01)  O Refused to Answer (skip to SMH01) |
| TRX17 | | Je uliwahi kataa kushiriki ngono na mshiriki wako mkuu au mshiriki wako asiye wa ngono ili upate kitu?  Did you withhold sex in order to get something from a primary partner or a non-primary partner? | | O Primary Partner  O Non-Primary Partner  OBoth  O Don’t Know  O Refused to Answer |
| **Section C6: Social and Mental Health (SMH)**  Interviewer: Kwa sasa ningependa kukuuliza maswali kuhusu jinsi ushiriki wako wa ngono imefanya ufikirie juu yako mwenyewe au uusiano wako na wengine kwenye jamii yako au jamii.  Now I would like to ask you questions about how your sexual activity has impacted how you feel about yourself, or impacted your relationships with others in your family or community. | | | | |
| SMH01 | | Ni watu gani umewahi waambia kuhusu mienendo yako ya ngono au kwamba una washiriki wengi wa ngono?  Which individuals have you told about your sexual activities or that you have multiple partners?  *Choose all that apply.* | O No one  O A family member  O A friend  O A healthcare provider  O A community member: specify ____________________________  O My primary partner  O Other: specify _________________________________________  O Refused to Answer | |
| SMH02 | | Kwa miezi 12 iliyopita, je yoyote kati ya hizi imewhai kukutendekea kutokana na kwa kushiriki ngono?  In the past 12 months, have any of the following **actually** happened to you as a direct result of your sexual activity?  *Interviewer: Read each option aloud one at a time to the participant and choose all that apply before moving on to the next question.* | O Nilihisi aibu au kujihisi sifai.  O Nilipoteza heshima au maadili kwa familiayangu au kijiji.  O Nilikatazwa kuingia kwenye matukio ya kijiji (k.v kliniki, kanisa, pahali pa michezo, mikutano)  O Nilishtuliwa na kuumizwa na mtu yeyote kutoka kwa familia.  O Nilitukanwa, nyanyaswa au kutishiwa na mtu yeyote katika familia yako au kijiji.  O Kudhulumiwa, kutengwa au kushugulikiwa vibaya na mfanyikazi wa afya.  O Kunyimwa huduma za afya  O None of the above  O Refused to Answer | |
| SMH03 | | Je, kwa miezi 12 iliyopita, umewahi kuwa na hofu ya uwezekano wa haya kukutendekea kutokana na miendeno yako ya ngono?  In the past 12 months, have you been **afraid** of any of the following **possibly** happening to you as a direct result of your sexual activity?  *Interviewer: Read each option aloud one at a time to the participant and choose all that apply before moving on to the next question.* | O Nilikuwa na uoga wa kupoteza heshima au kusimama kwenye familia au kijiji.  O Nimekuwa na uoga ya kuingia kwenye matukio ya kijiji(k.v kliniki, kanisa, pahali pa michezo, mikutano)  O Kuwa na uoga wa kudhulumiwa na mtu kwa familia yako au kijiji  O Kuwa na uoga wa kudhulumiwa, kunyanyaswa na/au kushtuliwa na mtu kutoka kwa familia au kijiji  O Nimekuwa na uoga kuwa nitahudumiwa vibaya na mhudumu wa afya au kunyimwa huduma za afya  O Nimekuwa na uoga ya kuwa watu watadhani niko na VVU  O None of the above  O Refused to answer | |

| Interviewer to Read: For this section, Nitakusomea maandiko Fulani. Tafadhali eleza jinsi unavyohisi kufuatana na maandiko hiyo  I will read a statement to you. Please describe how you feel about the statement. | | |
| --- | --- | --- |
| SMH04 | Wanaume wengi katika kijiji hiki wana watusi kwa maneno kuwanyanyasa au kuwatisha wanawake wanao badilisha ngono kwa pesa, bidhaa, zawadi, chakula, makaazi (malipo ya: stima, maji, nyumba nkt) au kuwashawishi.  Most men in this community verbally insult, harass, or threaten women who exchange sex for money, goods, gifts, food, housing, or influence. | OSikubaliani nayo kabisa  OSikubaliani nayo  ONiko kati kati  ONinakubaliana nayo  ONinakubaliana nayo kabisa  O Don’t Know  O Refused to Answer |
| SMH05 | Wanaume wengi katika kijiji hiki wanawadhulumu, kuwanyanyasa au kushtua wanawake wanao badilisha ngono kwa pesa, bidhaa, zawadi, chakula, makaazi (malipo ya: stima, maji, nyumba nkt ) au kushawishi.  Most men in this community physically insult, harass, or threaten women who exchange sex for money, goods, gifts, food, housing, or influence. | O Sikubaliani nayo kabisa  O Sikubaliani nayo  O Niko kati kati  O Ninakubaliana nayo  O Ninakubaliana nayo kabisa  O Don’t Know  O Refused to Answer |
| SMH06 | Wanaume wengi katika jamii hii hawawezi kumoa mwanamke ambaye anabadilisha ngono kwa pesa, bidhaa, zawadi, chakula, makaazi (malipo ya: stima,maji, nyumba nkt), au kushawishi.  Most men in this community would not marry a woman who has exchanged sex for money, goods, gifts, food, housing, or influence. | O Sikubaliani nayo kabisa  O Sikubaliani nayo  O Niko kati kati  O Ninakubaliana nayo  O Ninakubaliana nayo kabisa  O Don’t Know  O Refused to Answer |
| SMH07 | Watu wengi katika kijiji hiki hawaheshimu wanawake ambao wanabadilisha ngono kwa pesa, bidhaa, chakula, makaazi (malipo ya: stima.maji nyumba nkt) au kushawishi.  Most people in this community do not respect women who exchange sex for money, goods, gifts, food, housing, or influence. | O Sikubaliani nayo kabisa  O Sikubaliani nayo  O Niko kati kati  O Ninakubaliana nayo  O Ninakubaliana nayo kabisa  O Don’t Know  O Refused to Answer |
| SMH08 | Wengi wa wahudumu wa afya katika kijij hiki wanawatusi, wanawabagua au wanawadharau, wanawake ambao wanabadilisha ngono kwa pesa, bidhaa, chakula, makaazi (malipo ya: stima, maji, nyumba nkt) au kushawishi.  Most healthcare providers in this community insult, discriminate, or treat poorly women who exchange sex for money, goods, gifts, food, housing, or influence. | O Sikubaliani nayo kabisa  O Sikubaliani nayo  O Niko kati kati  O Ninakubaliana nayo  O Ninakubaliana nayo kabisa  O Don’t Know  O Refused to Answer |
| SMH09 | Wengi wa wahudumu wa afya katika kijii hiki wanakataa kupatiana huduma kwa wanawake ambao wanabadilisha ngono kwa pesa, bidhaa, chakula, makaazi (malipo ya: stima, maji, nyumba nkt) au kushawishi.  Most healthcare providers in this community refuse services to women who exchange sex for money, goods, gifts, food, housing, or influence. | O Sikubaliani nayo kabisa  O Sikubaliani nayo  O Niko kati kati  O Ninakubaliana nayo  O Ninakubaliana nayo kabisa  O Don’t Know  O Refused to Answer |

| **Section D: Recent Transactional Encounters (RT#)** | | |
| --- | --- | --- |
| Interviewer:Kwa sasa , nitakuuliza baadhi ya maswali inayo ambatana na ushiriki wako wa ngono ya hivi majuzi aliyekupatia pesa au kitu nyingine kwa kushiriki ngono. Nimeuliza maswali mengine sawa na hayo lakini kwa wakati huu nitauliza ufafanue zaidi. Nashukuru sana utulivu wako kupitia baadhi ya maswali yafuatayo. Ningependa ufikirie kuhusu ushiriki wako wa ngono ya hivi majuzi zaidi ulipobadilisha ngono na pesa, bidhaa, zawadi, chakula au nyumba. Niatkuuliza baadhi ya maswali kuhusu kubadilisha hiki.  Now, I will be asking you a series of questions related to your recent sexual partners from whom you received money or something else in exchange for sex. I have asked some similar questions but now I will ask for some more details. I greatly appreciate your patience through the next series of questions. I want you to think about the most recent time you exchanged sex for money, goods, gift, food, or housing. I am going to ask you several questions about this exchange. | | |
| **No.** | **Question** | **Coding** |
| RT1: Most Recent Exchange | | |
| RT1 Q1 | Je ungependa kunijulisha kuhusu wakati wa hivi karibuni ulipobadilisha ngono?  Would you like to tell me about the most recent time you exchanged sex? | O Yes  O No (skip to RT2 Q1) |
| RT1 Q2 | Huku kubadilisha ngono kwa pesa, bidhaa au zawadi ilitendeka lini?  When did this sex exchange for money, goods, or gifts occur? | O I Know the Date  O Don’t Know (skip to RT1 Q4)  O Refused to Answer (skip to RTI Q34) |
| RT1 Q3 | Ilikuwa tarehe gani?  What was the date? | ___ ___/___ ___ ___/ ___ ___ ___ ___  D D M M M Y Y Y Y |
| RT1 Q4 | Ni wakati gani hiki kitendo  kilitendeka?  At what time of day did this encounter take place? | O Early Morning (00:01-08:00)  O Morning (8:01-12:00)  O Afternoon/Evening (12:01-18:00)  O Night (18:01-24:00) O Don’t Know  O Refused to Answer |
| RT1 Q5 | Ni pahali gani ulibadilisha ngono na mtu huyu?  Where did you exchange sex with this person? | O Street, car, or outside  O Bar or nightclub  O Hotel room paid by sex worker  O Hotel room paid by partner  O Brothel  O Woman’s home  O Man’s home  O Other: _______________________________  O Don't Know  O Refused to Answer |
| RT1 Q6 | Tafadhali nielezee , ni vitendo vipi vifuatavyo ulivyotenda wakati wa tukio hiki:  Please tell me which of the following activities you did during this encounter:  *Interviewer: Read the list of responses aloud to the participant and choose all that apply. Explain any of the choices that the participant does not understand.* | O Kubusu  O Nilicheza ngoma na kuvulia mpenzi nguo  O Kukanda (massage)  O Kushiriki ngono kupitia mdomo **kutumia** kondomu  O Kushiriki ngono kupitia mdomo **bila kutumia** kondomu  O Ngono ya kupitia uke **nikitumia** kondomu  O Ngono ya kupitia uke **bila kutumia** kondomu  O Ngono ya mkundu **ukitumia** kondomu  O Ngono ya mkundu **bila kutumia** kondomu  O Nilipokea ngono kwa mdomo  O Kuongea/ au kupumzika pamoja  O Ingine: _______________________________  _______________________________________ |
| RT1 Q7 | Je hii ilikuwa mara yako ya kwanza kibadilisha ngono na mtu huyu?  Was this the first time you have exchanged sex with this person? | O Yes  O No  O Don’t Know O Refused to Answer |
| RT1 Q8 | Je, huyu mtu alikuwa na maumbile ya kuvutia kiasi gani?  How handsome was this person?  *Interviewer: Remember to probe for this question to determine what the participant thinks is handsome (dressed nicely, physical features, humor, etc.).* | O Not very handsome  O About average  O Handsome  O Don’t Know  O Refused to Answer |
| RT1 Q9 | Kwa takriban huyu mtu alikuwa na miaka mingapi? Tafadhali kadiri.  Approximately how old was this person? Please estimate. | ___ ___ years  O Don’t know  O Refused to answer |
| RT1 Q10 | Huyu mtu alikuwa tajiri kiasi gani?  How wealthy was this person? | O Poor  O Average wealth  O Above-average wealth  O Very wealthy  O Don’t know  O Refused to answer |
| RT1 Q11 | Je, huyu mtu anaishi katika eneo ya kaunti hii, au alikuja kwa matembezi?  Does this person live in this County, or was he visiting? | O Lives in this County  O Just visiting  O Don’t know  O Refused to answer |
| RT1 Q12 | Je, huyu mtu alitumia pombe au miadarati kwa wakati mlishiriki ngono?  Did this person take alcohol or drugs around the time you had sex? | O Yes  O No  O Don’t know  O Refused to answer |
| RT1 Q13 | Je, ulikunywa pombe au kutumia miadarati wakati uliposhiriki ngono na mtu huyu?  Did YOU take alcohol or drugs around the time you had sex with this person? | O Yes  O No  O Don’t know  O Refused to answer |
| RT1 Q14 | Je, kuna uwezekano gani kuwa mtu huyu alikuwa anaugua na ugonjwa wa zinaa kando na VVU?  How likely is it that this person had a sexually transmitted infection besides HIV? | O Almost impossible  O A little likely  O Somewhat likely  O Very likely  O Almost certain  O Don't Know  O Refused to Answer |
| RT1 Q15 | Je, kuna uwezekano gani kuwa huyu mtu alikuwa na VVU?  How likely is it that this person had HIV? | O Almost impossible  O A little likely  O Somewhat likely  O Very likely  O Almost certain  O Don't Know  O Refused to Answer |
| RT1 Q16 | Ni nini ulipokea kwa kubadilisha ngono na mtu huyu?  What did you receive in exchange for having sex with this person?  *Interviewer: Choose all that apply.* | O Housing and/or utilities  O Food to eat  O Food to sell (example, fish)  O School fees  O To get a job, a work promotion, or to keep your job  O Other material goods (clothes, jewelry, makeup, electronics, etc.)  O Household items (soap, cleaning supplies, tools, etc.)  O Other: _______________________________  O Don’t Know  O Refused to Answer |
| RT1 Q17 | Ulipokea jumla ya pesa ngapi, bidhaa au zawadi ambayo mshiriki wako wa ngono alikupa katika tukio hiki?  What was the **total value** of money, goods and gifts that the person gave you for this encounter? | KES_________  O Don't Know  O Refused to Answer |
| Interviewer READ: Kwa sasa nitakuuliza baadhi ya maswali kuhusu vitu mtu huyu kuna uwezekano alikufanyia wakati wa ushiriki huu. Maswali haya ni juu ya vita, ambazo zinaweza kukufanya usihisi vizuri au kutaabika. Tafadhali kumbuka yakuwa jibu lako zimewekwa kwa hali ya siri na hakuna atakaye husisha ujumbe huu na wewe au mshiriki wako wa ngono. Siyo lazima ujibu swali lolote inayokufanya usihisi vizuri. Tafadhali chukua muda wako na usipoelewa swali lolote, niulize. Uko tayari tuendelee?  I will now ask you some questions about things this same person may have done to you during this encounter. These questions are about violence, which may make you feel uncomfortable or distressed. Please remember that your answers are completely confidential and no one will be able to associate this information with you or your sexual partners. You do not have to answer any question that makes you uncomfortable. Please take your time and if you are unclear about any question, just ask me. Are you ready to continue? | | |
| RT1 Q18 | Je, mpenzi huyu alikutishia kwa kukupiga  Did this partner threaten you with physical assault? | O Yes  O No  O Don’t know  O Refused to answer |
| RT1 Q19 | Je huyu mpenzi alikugonga, kukupiga teke, kukunyonga ama kukuumiza?  Did this partner hit, kick, strangle or otherwise physically assault you? | O Yes  O No  O Don’t know  O Refused to answer |
| RT1 Q20 | Je, huyu mpenzi alikulazimisha kushiriki katika tendo cha ngono hiari yako?  Did this partner force or coerce you to participate in any sex act against your will? | O Yes, to have sex with a condom  O Yes, to have sex without a condom  O Yes, to have anal sex  O Yes, Other____________________________  O No  O Don’t know  O Refused to answer |
| Interviewer: Sasa, nitakuuliza maswali sawa na niliyokuuliza hapo awali, kwa ushiriki wako wako wa ngono ya karibu zaidi ya pili ulipobadilisha ngono na kitu. Hii inaweza kuwa na mshiriki tofauti kama taliyezungumzia au huyo.  Now, I will be asking you the same questions as I just did, for your second most recent encounter where you exchanged sex for something. This could be with a different partner as the one we just discussed, or the same. | | |
| RT2: Second Most Recent Exchange | | |
| **No.** | **Question** | **Coding** |
| RT2 Q1 | Je ungependa kunielezea kuhusu wakati wa pili wa hivi karibuni ulipobadilisha ngono?  Would you like to tell me about the second most recent time you exchanged sex? | O Yes  O No (skip to RT3 Q1) |
| RT2 Q2 | Je huyu ni Yule mtu tumejadili kumhusu kwa wakati wa mwisho ulipobadilisha ngono?  Is this the same person we just spoke of for the last time you exchanged sex? | O Yes *(Do not ask RT2 Q8 thru Q13 or Q15 thru Q16)*  O No |
| RT2 Q3 | Huku kubadilisha ngono kwa pesa, bidhaa au zawadi ilitendeka lini?  When did this sex exchange for money, goods, or gifts occur? | O I Know the Date  O Don’t Know (skip to RT1 Q4)  O Refused to Answer (skip to RTI Q34) |
| RT2 Q4 | Ilikuwa tarehe gani?  What was the date? | ___ ___/___ ___ ___/ ___ ___ ___ ___  D D M M M Y Y Y Y |
| RT2 Q5 | Ni wakati gani hiki kitendo  kilitendeka?  At what time of day did this encounter take place? | O Early Morning (00:01-08:00)  O Morning (8:01-12:00)  O Afternoon/Evening (12:01-18:00)  O Night (18:01-24:00) O Don’t Know  O Refused to Answer |
| RT2 Q6 | Ni pahali gani ulibadilisha ngono na mtu huyu?  Where did you exchange sex with this person? | O Street, car, or outside  O Bar or nightclub  O Hotel room paid by sex worker  O Hotel room paid by partner  O Brothel  O Woman’s home  O Man’s home  O Other: _______________________________  O Don't Know  O Refused to Answer |
| RT2 Q7 | Tafadhali nielezee , ni vitendo vipi vifuatavyo ulivyotenda wakati wa tukio hiki:  Please tell me which of the following activities you did during this encounter:  *Interviewer: Read the list of responses aloud to the participant and choose all that apply. Explain any of the choices that the participant does not understand.* | O Kubusu  O Nilicheza ngoma na kuvulia mpenzi nguo  O Kukanda (massage)  O Kushiriki ngono kupitia mdomo **kutumia** kondomu  O Kushiriki ngono kupitia mdomo **bila kutumia** kondomu  O Ngono ya kupitia uke **nikitumia** kondomu  O Ngono ya kupitia uke **bila kutumia** kondomu  O Ngono ya mkundu **ukitumia** kondomu  O Ngono ya mkundu **bila kutumia** kondomu  O Nilipokea ngono kwa mdomo  O Kuongea/ au kupumzika pamoja  O Ingine: _______________________________  _______________________________________ |
| RT2 Q8 | *Do not ask if RT2 Q2 = Yes*  Je hii ilikuwa mara yako ya kwanza kibadilisha ngono na mtu huyu?  Was this the first time you have exchanged sex with this person? | O Yes  O No  O Don’t Know O Refused to Answer |
| RT2 Q9 | *Do not ask if RT2 Q2 = Yes*  Je, huyu mtu alikuwa na maumbile ya kuvutia kiasi gani?  How handsome was this person?  *Interviewer: Remember to probe for this question to determine what the participant thinks is handsome (dressed nicely, physical features, humor, etc.).* | O Not very handsome  O About average  O Handsome  O Don’t Know  O Refused to Answer |
| RT2 Q10 | *Do not ask if RT2 Q2 = Yes*  Kwa takriban huyu mtu alikuwa na miaka mingapi? Tafadhali kadiri.  Approximately how old was this person? Please estimate. | ___ ___ years  O Don’t know  O Refused to answer |
| RT2 Q11 | *Do not ask if RT2 Q2 = Yes*  Huyu mtu alikuwa tajiri kiasi gani?  How wealthy was this person? | O Poor  O Average wealth  O Above-average wealth  O Very wealthy  O Don’t know  O Refused to answer |
| RT2 Q12 | *Do not ask if RT2 Q2 = Yes*  Je, huyu mtu anaishi katika eneo la kaunti hii, au alikuja kwa matembezi?  Does this person live in this County, or was he visiting? | O Lives in this County  O Just visiting  O Don’t know  O Refused to answer |
| RT2 Q13 | Je, huyu mtu alitumia pombe au miadarati kwa wakati mlishiriki ngono?  Did this person take alcohol or drugs around the time you had sex? | O Yes  O No  O Don’t know  O Refused to answer |
| RT2 Q14 | Je, ulikunywa pombe au kutumia miadarati wakati uliposhiriki ngono na mtu huyu?  Did **you** take alcohol or drugs around the time you had sex with this person? | O Yes  O No  O Don’t know  O Refused to answer |
| RT2 Q15 | *Do not ask if RT2 Q2 = Yes*  Je, kuna uwezekano gani kuwa mtu huyu alikuwa anaugua na ugonjwa wa zinaa kando na VVU?  How likely is it that this person had a sexually transmitted infection besides HIV? | O Almost impossible  O A little likely  O Somewhat likely  O Very likely  O Almost certain  O Don't Know  O Refused to Answer |
| RT2 Q16 | *Do not ask if RT2 Q2 = Yes*  Je, kuna uwezekano gani kuwa huyu mtu alikuwa na VVU?  How likely is it that this person had HIV? | O Almost impossible  O A little likely  O Somewhat likely  O Very likely  O Almost certain  O Don't Know  O Refused to Answer |
| RT2 Q17 | Ni nini ulipokea kwa kubadilisha ngono na mtu huyu?  What did you receive in exchange for having sex with this person?  *Interviewer: Check all that apply.* | O Housing and/or utilities  O Food to eat  O Food to sell (example, fish)  O School fees  O To get a job, a work promotion, or to keep your job  O Other material goods (clothes, jewelry, makeup, electronics, etc.)  O Household items (soap, cleaning supplies, tools, etc.)  O Other: _______________________________  O Don’t Know  O Refused to Answer |
| RT2 Q18 | Ulipokea jumla ya pesa ngapi, bidhaa au zawadi ambayo mshiriki wako wa ngono alikupa katika tukio hiki?  What was the **total value** of money, goods and gifts that the person gave you for this encounter? | KES_________  O Don't Know  O Refused to Answer |
| RT2 Q19 | Je, mpenzi huyu alikutisha kwa kukupiga  Did this partner threaten you with physical assault? | O Yes  O No  O Don’t know  O Refused to answer |
| RT2 Q20 | Je huyu mpenzi alikugonga, kukupiga teke, kukunyonga ama kukuumiza?  Did this partner hit, kick, strangle or otherwise physically assault you? | O Yes  O No  O Don’t know  O Refused to answer |
| RT2 Q21 | Je, huyu mpenzi alikulazimisha kushiriki katika tendo cha ngono hiari yako?  Did this partner force or coerce you to participate in any sex act against your will? | O Yes, to have sex with a condom  O Yes, to have sex without a condom  O Yes, to have anal sex  O Yes, Other__________________________  O No  O Don’t know  O Refused to answer |
| Interviewer: Kwa mara ya mwisho, nitakuuliza maswali sawa na niliyeuliza kuhusu mshiriki wako wa karibu zaidi wa tatu uliyebadilisha ngono na kitu. Hii ianweza kuwa na mshiriki tofauti kama tuliyezungumzia au huyo.  For the last time, I will be asking you the same questions as I just did about your third most recent encounter where you exchanged sex for something. This could be with a different partner as the one we just discussed, or the same. | | |
| RT3: Third Most Recent Exchange | | |
| **No.** | **Question** | **Coding** |
| RT3 Q1 | Je ungependa kunielezea kuhusu wakati wa tatu wa hivi karibuni ulipobadilisha ngono?  Would you like to tell me about the third most recent time you exchanged sex? | O Yes  O No (skip to HIV01) |
| RT3 Q2 | Je huyu ni Yule mtu tumejadili kumhusu kwa wakati wa mwisho ulipobadilisha ngono?  Is this the same person we just spoke of for the last time you exchanged sex? | O Yes *(Do not ask RT3 Q8 thru Q13 or Q15 thru Q16)*  O No |
| RT3 Q3 | Huku kubadilisha ngono kwa pesa, bidhaa au zawadi ilitendeka lini?  When did this sex exchange for money, goods, or gifts occur? | O I Know the Date  O Don’t Know (skip to RT1 Q4)  O Refused to Answer (skip to RTI Q34) |
| RT3 Q4 | Ilikuwa tarehe gani?  What was the date? | ___ ___/___ ___ ___/ ___ ___ ___ ___  D D M M M Y Y Y Y |
| RT3 Q5 | Ni wakati gani hiki kitendo  kilitendeka?  At what time of day did this encounter take place? | O Early Morning (00:01-08:00)  O Morning (8:01-12:00)  O Afternoon/Evening (12:01-18:00)  O Night (18:01-24:00) O Don’t Know  O Refused to Answer |
| RT3 Q6 | Ni pahali gani ulibadilisha ngono na mtu huyu?  Where did you exchange sex with this person? | O Street, car, or outside  O Bar or nightclub  O Hotel room paid by sex worker  O Hotel room paid by partner  O Brothel  O Woman’s home  O Man’s home  O Other: _______________________________  O Don't Know  O Refused to Answer |
| RT3 Q7 | Tafadhali nielezee , ni vitendo vipi vifuatavyo ulivyotenda wakati wa tukio hiki:  Please tell me which of the following activities you did during this encounter:  *Interviewer: Read the list of responses aloud to the participant and choose all that apply. Explain any of the choices that the participant does not understand.* | O Kubusu  O Nilicheza ngoma na kuvulia mpenzi nguo  O Kukanda (massage)  O Kushiriki ngono kupitia mdomo **kutumia** kondomu  O Kushiriki ngono kupitia mdomo **bila kutumia** kondomu  O Ngono ya kupitia uke **nikitumia** kondomu  O Ngono ya kupitia uke **bila kutumia** kondomu  O Ngono ya mkundu **ukitumia** kondomu  O Ngono ya mkundu **bila kutumia** kondomu  O Nilipokea ngono kwa mdomo  O Kuongea/ au kupumzika pamoja  O Ingine: _______________________________  _______________________________________ |
| RT3 Q8 | *Do not ask if RT3 Q2 = Yes*  Je hii ilikuwa mara yako ya kwanza kibadilisha ngono na mtu huyu?  Was this the first time you have exchanged sex with this person? | O Yes  O No  O Don’t Know O Refused to Answer |
| RT3 Q9 | *Do not ask if RT3 Q2 = Yes*  Je, huyu mtu alikuwa na maumbile ya kuvutia kiasi gani?  How handsome was this person?  *Interviewer: Remember to probe for this question to determine what the participant thinks is handsome (dressed nicely, physical features, humor, etc.).* | O Not very handsome  O About average  O Handsome  O Don’t Know  O Refused to Answer |
| RT3 Q10 | *Do not ask if RT3 Q2 = Yes*  Kwa takriban huyu mtu alikuwa na miaka mingapi? Tafadhali kadiri.  Approximately how old was this person? Please estimate. | ___ ___ years  O Don’t know  O Refused to answer |
| RT3 Q11 | *Do not ask if RT3 Q2 = Yes*  Huyu mtu alikuwa tajiri kiasi gani?  How wealthy was this person? | O Poor  O Average wealth  O Above-average wealth  O Very wealthy  O Don’t know  O Refused to answer |
| RT3 Q12 | *Do not ask if RT3 Q2 = Yes*  Je, huyu mtu anaishi katika eneo la kaunti hii, au alikuja kwa matembezi?  Does this person live in this County, or was he visiting? | O Lives in this County  O Just visiting  O Don’t know  O Refused to answer |
| RT3 Q13 | Je, huyu mtu alitumia pombe au miadarati kwa wakati mlishiriki ngono?  Did this person take alcohol or drugs around the time you had sex? | O Yes  O No  O Don’t know  O Refused to answer |
| RT3 Q14 | Je, ulikunywa pombe au kutumia miadarati wakati uliposhiriki ngono na mtu huyu?  Did **you** take alcohol or drugs around the time you had sex with this person? | O Yes  O No  O Don’t know  O Refused to answer |
| RT3 Q15 | *Do not ask if RT3 Q2 = Yes*  Je, kuna uwezekano gani kuwa mtu huyu alikuwa anaugua na ugonjwa wa zinaa kando na VVU?  How likely is it that this person had a sexually transmitted infection besides HIV? | O Almost impossible  O A little likely  O Somewhat likely  O Very likely  O Almost certain  O Don't Know  O Refused to Answer |
| RT3 Q16 | *Do not ask if RT3 Q2 = Yes*  Je, kuna uwezekano gani kuwa huyu mtu alikuwa na VVU?  How likely is it that this person had HIV? | O Almost impossible  O A little likely  O Somewhat likely  O Very likely  O Almost certain  O Don't Know  O Refused to Answer |
| RT3 Q17 | Ni nini ulipokea kwa kubadilisha ngono na mtu huyu?  What did you receive in exchange for having sex with this person?  *Interviewer: Choose all that apply.* | O Housing and/or utilities  O Food to eat  O Food to sell (example, fish)  O School fees  O To get a job, a work promotion, or to keep your job  O Other material goods (clothes, jewelry, makeup, electronics, etc.)  O Household items (soap, cleaning supplies, tools, etc.)  O Other: _______________________________  O Don’t Know  O Refused to Answer |
| RT3 Q18 | Ulipokea jumla ya pesa ngapi, bidhaa au zawadi ambayo mshiriki wako wa ngono alikupa katika tukio hiki?  What was the **total value** of money, goods and gifts that the person gave you for this encounter? | KES_________  O Don't Know  O Refused to Answer |
| RT3 Q19 | Je, mpenzi huyu alikutisha kwa kukupiga  Did this partner threaten you with physical assault? | O Yes  O No  O Don’t know  O Refused to answer |
| RT3 Q20 | Je huyu mpenzi alikugonga, kukupiga teke, kukunyonga ama kukuumiza?  Did this partner hit, kick, strangle or otherwise physically assault you? | O Yes  O No  O Don’t know  O Refused to answer |
| RT3 Q21 | Je, huyu mpenzi alikulazimisha kushiriki katika tendo cha ngono bila hiari yako?  Did this partner force or coerce you to participate in any sex act against your will? | O Yes, to have sex with a condom  O Yes, to have sex without a condom  O Yes, to have anal sex  O Yes, Other__________________________  O No  O Don’t know  O Refused to answer |

| **Section E: HIV and HIV Testing** | | |
| --- | --- | --- |
| **Section E1: General HIV Knowledge (HIV)**  Interviewer: Ningependa kukuuliza baadhi ya maswali kuhusu kile tayari unafahamu na kuhisi juu ya Ukimwi  I would now like to ask you some questions about what you already know and feel about HIV. | | |
| HIV01 | Je kwa sasa unameza dawa zozote za kuzuia maambukizi ya VVU (PrEP)? Hii kwa kawaida ni tembe inayomezwa kila siku.  Are you currently taking any HIV medication in order to **prevent** acquiring HIV (PrEP)? This is usually a pill taken daily.  *Interviewer: Ensure participant understands the principles of PrEP before proceeding.* | O Yes  O No  O Don’t Know  O Refused to Answer |
| HIV02 | Je, unafikiri nafasi yako ya kuambukizwa virusi vya VVU ni ipi katika siku zijazo?  What do you think your chances are of acquiring HIV in the future? | O None (Ask HIV03, not HIV04)  O Low (Ask HIV03, not HIV04)  O Moderate (skip to HIV04)  O High (skip to HIV04)  O Don’t Know (skip to HIV05)  O Refused to Answer (skip to HIV05) |
| HIV03 | *Only ask if HIV02 = None or Low*  Je, ni kwa nini unafikiri kuwa una nafasi ya chini au hauna nafasi ya kuambukizwa VVU?  Why do you think you have a low chance or no chance of acquiring HIV in the future?  *Choose all that apply based on what participant mentioned.* | O Is Not Having Sex  O Uses Condoms  O Has Only One Partner  O Limits the Number of Partners  O Partner Has No Other Partners  O Knows Partner(s)’ HIV Status is Negative  O Trusts partner  O My Current Status is Negative  O Other _________________________________  O Don’t Know  O Refused to Answer |
| HIV04 | *Only ask if HIV02 = Moderate or High*  Je, ni kwa nini unafikiri kuwa nafasi yako ya kuambukizwa VVU ni ya kadri au ya juu?  Why do you think you have a moderate or high chance of acquiring HIV in the future?  *Choose all that apply based on what the participant mentioned.* | O Does Not Use Condoms Regularly or at all  O Woman Has More Than One Partner  O Has Transactional Sex  O Does Not Trust Partner  O Partner is HIV positive  O She or Partner Refuses to be Tested  O Uses Injection Drugs/ Needles  O Primary Partner has more than one partner  O Non-primary partner(s) have more than one partner  O Other _________________________________  O Don’t Know  O Refused to Answer |
| HIV05 | Je kuna uwezekano kuwa mtu mmoja aliye katika uhusiano wa imara anauwezekano wa kuambukizwa VVU na mwenzake anabaki bila kuambukizwa?  Is it possible for one person in a steady sexual relationship to be infected with HIV and the other person to remain uninfected? | 1: Yes  2: No  98: Don’t Know  99: Refused to Answer |
| HIV06 | Ikiwa wanaume 100 wasio na VVU watashiriki ngono bila kutumia mpira wa kondomu**mara moja**na mwanamke aliye na VVU, je ni wanaume wangapi kati yao watakuwa na VVU hapo baadaye?  If 100 HIV negative men have unprotected sex **once** with a woman who is HIV positive, how many of the men will have HIV afterwards?  *Interviewer: Remember, this question is about learning the participant’s perception about risk of getting HIV if someone is exposed to it. The details (vaginal sex, lubrication, ARVs, etc.) aren’t important for this question – exposure is.* | Number of men: ___________  O Don’t know  O Refused to answer |
| HIV07 | Unafiki mtu aliye na VVU anaweza meza dawa kupunguza hatari ya kuambukizwa virusi kwa mpenzi wake wa ngono?  Do you think that a person who has HIV can take medicine to reduce her risk of transmitting the virus to a sexual partner? | O Yes  O No  O Don’t know  O Refused to answer |
| HIV08 | Unafikiria VVU vina tiba?  Do you think that HIV can be cured? | O Yes  O No  O Don’t know  O Refused to answer |
| HIV09 | Je unaamini unafaa kupimwa vvu mara kwa mara mara ngapi?  How often do you believe you should test for HIV?  *Choose all that apply.* | O Every week  O Every month  O Every 3 months  O Every 6 months  O Every year  O When I have sex without a condom  O When I have a new partner  O When my primary partner informs me or I know that he was unfaithful  O During pregnancy  O Other: ________________________________  O If I have been tested once, I do not need to be tested again  O Don’t Know  O Refused to Answer |
| **Section E2: Sexual Testing History (TST)**  Interviewer: Ningependa kukuuliza baadhi ya maswali kuhusu upimaji wa virusi vya ukimwi na magonjwa mengine ya kisonono (STI) kama syphilis, gonorrhea, Chlamydia, Trichomonas vaginalis au bacterial vaginosis  I would now like to ask you some questions about testing for HIV and other sexually transmitted infections (STI) such as syphilis, gonorrhea, chlamydia, *Trichomonas vaginalis*, or bacterial vaginosis. | | |
| TST01 | Kabla ya kupimwa VVU leo, je, ushawahi pimwa hapo awali?  Before the test you took today, had you ever been tested for HIV? | O Yes  O No (skip to TST03)  O Don’t Know (skip to TST03)  O Refused to Answer (skip to TST03) |
| TST02 | Kabla ya kupimwa leo mara ya mwisho ulipimwa VVU?  Before the test today, how long ago was your last HIV test? | O < 3 Months  O 3-6 Months  O 7-12 Months  O 13-23 Months  O 2 Years or More  O Don’t Know  O Refused to Answer |
| TST03 | Katika miezi 6 zilizopita, je umewahi patikana na magonjwa ya zinaa?  During the past 6 months, have you been diagnosed with a STI? | O Yes  O No (skip to TST05)  O Don’t Know (skip to TST05)  O Refused to answer (skip to TST05) |
| TST04 | Ni ugonjwa/ magonjwa ipi/zipi ya zinaaulipatikana nayo?  Which STI(s) were you diagnosed with?  *Choose all that apply.*  *Interviewer: This is self-reported by the participant. If she does not remember the diagnosis, use “Don’t Know”. Do not try to diagnose her using symptoms she describes.* | O Trichomoniasis (Trich)  O Syphilis  O Gonorrhea  O Chlamydia  O Herpes  O Human papillomavirus (HPV)  O Genital warts  O Mycoplasma genitalium  O Bacterial vaginosis (BV)  O Other _______________________________  O Don’t Know  O Refused to answer |
| TST05 | Je, uliwahi kwenda katika duka la dawa na kuknunua dawa ya kutibu ugonjwa wa zinaa, bila kuuliza daktari ama mhudumu mwingine wa afya aliyehitimu?  Did you ever go to a pharmacy and purchase treatment for an STI, without consulting a doctor or other qualified healthcare provider (ex: over the counter medication)? | O Yes  O No  O Don’t Know  O Refused to answer |
| TST06 | Je, uliwahi tumia njia ya kienyeji au njia nyingine ya kutibu magonjwa ya zinaa, bila kuuliza daktari, kwenye duka la dawa ama mhudumu mwingine wa afya aliyehitimu?  Did you ever use a home remedy or treatment for an STI, without consulting a doctor, pharmacist or other qualified healthcare provider? | O Yes  O No  O Don’t Know  O Refused to answer |
| **Section E3: HIV Self-testing (HST)**  Interviewer: Jinsi nilivyo sema tukianza majadiliano haya, utafiti huu inanuia kukusanya maneno kuhusu upimaji kibinafsi wa ukimwi. Upimaji kibinafsi wa virusi wa ukimwi inamaanisha unaweza jipima mwenyewe ukiwa na virusi vya ukimwi kwa wakati au mahali unayopenda bila kuelekea kituo cha afya. Kipimo hiki haliitaji sampuli ya damu lakini inatumia sampuli kutoka ndani ya mdomo. Maswali yafuatayo aniaambatana na kujipima kwa virusi ya ukimwi mwenyewe.  As I mentioned before this interview, this study is interested in collecting information on HIV self-testing. HIV self-testing means you can test yourself for HIV at a time and location that is convenient for you, without having to go to a clinic. This test does not require a blood sample, but uses a sample from inside your mouth. The following questions are specific to HIV self-testing. | | |
| HST01 | Je, umewahi kuskia upimaji wa kibinafsi ya VVU kabla ya leo?  Have you ever heard of HIV self-testing before today? | O Yes  O No (skip to HST03)  O Don’t Know (skip to HST03)  O Refused to Answer |
| HST02 | Je, ushawai tumia kifaa cha kujipima virusi vya ukimwi kibinafsi kabla ya leo?  Have you ever used an HIV self-test before today? | O Yes  O No  O Don’t Know  O Refused to Answer |
| HST03 | Iwapo vifaa vya kujipima kibinafsi zingekuwa zinapatikana, je ungekuwa na hamu ya kujipima VVU kibinafsi?  If HIV self-testing were available to you, how interested would you be in testing yourself for HIV? | O Very Interested  O Somewhat Interested  O Neutral, Neither Interested nor Uninterested  O Somewhat Uninterested  O Very Uninterested  O Don’t Know  O Refused to Answer |
| HST04 | Iwapo vifaa vya kujipima VVU kibinafsi zingekuwa zinapatikana je unafikiria utajipima virusi vya Ukimwi mara kwa mara?  If HIV self-testing were available to you, do you think you would test for HIV more frequently? | O Yes, More Frequently  O No, Less Frequently  O No change in Frequency  O Don’t Know  O Refused to Answer |
| HST05 | Ikiwa ulipokea matokeo kuwa una virisi vya Ukimwi, unafikiria unauwezekano upi wa kwenda kuhakikisha matokeo kwa mhudumu wa afya?  If you received a positive HIV self-test result, how likely do you think you would be to seek confirmatory testing with a healthcare provider? | O Very Likely  O Somewhat Likely  O Neutral, Neither Likely Nor Unlikely  O Somewhat Unlikely  O Very Unlikely  O Don’t Know  O Refused to Answer |
| HST06 | Unaweza kuwa huru kiasi gani kuchukua kifaa cha kujipima kibinafsi na kumpa mshiriki wako mkuu wa ngono kutumia?  How comfortable would you be in taking an HIV self-test to give to your **primary** partner to use? | O Very Comfortable  O Somewhat Comfortable  O Neutral, Neither Comfortable Nor Uncomfortable  O Somewhat Uncomfortable  O Very Uncomfortable  O Don’t Know  O Refused to Answer |
| HST07 | Unaweza kuwa huru kiasi gani kuchukua kifaa cha kujipima kibinafsi na kumpa mshiriki wako **asiye mshiriki/ washiriki mkuu/ wakuu wa ngono** kutumia?  How comfortable would you be in taking an HIV self-test to give to your **non-primary** partner(s) to use? | O Very Comfortable  O Somewhat Comfortable  O Neutral, Neither Comfortable Nor Uncomfortable  O Somewhat Uncomfortable  O Very Uncomfortable  O Don’t Know  O Refused to Answer |

| **Section F: Gender-Based Violence (GBV)** | | |
| --- | --- | --- |
| Interviewer:Maswali yafuatatyo ni kuhusu vitu zinazofanyika ndani ya baadhi ya ushiriano na kuwa mshiriki wako wa karibu au mshiriki yeyote anaweza kuwa alikufanyia. Maswali haya ni kuhusu vita amabazo zaweza kukufanya usihisi vizuri au kutaabika. Siyo lazima kujibu maswali yoyote inayokufanya usihisi vizuri na majibu yako yako kwa hali ya siri. Ikiwa una swali yoyote kwa wakati wowote tafadhali niulize. Uko tayari tuendelee?  The next questions are about things that happen within some relationships, and that your primary partner, or any other partners may have done to you. These questions are about violence, which may make you feel uncomfortable or distressed. You do not have to answer any question that makes you feel uncomfortable, and your responses are confidential. If you have any questions at any time, please ask me. Are you ready to continue? | | |
| GBV01:  Je, kwa miezi 12 iliyopita, ni mara ngapi **mpenzi wako wa kawaida** amekufanyia haya?  In the past 12 months has your **PRIMARY** sexual partner done any of the following to you?  *Interviewer: Read each option aloud one at a time and choose all that apply.* | GBV02:  Kwa miezi 12 uliyopita je mshiriki wako mkuu amewahi fanya hivi mara kwa mara au wakati mwingine?  In the past 12 months, has your **PRIMARY** partner done this often or only sometimes? | GBV03:  Je umewahi kumwambia mtu yeyote kwamba mshiriki wako mkuu alikutendea na kama ni hivyo ulimjulisha nani?  Did you ever tell anyone that your **PRIMARY** partner did this, and if so who did you tell? |
| O Alikutusi au kukufanya hujihisi vibaya.  Insulted or made you feel bad about yourself | O Often  O Sometimes  O Don’t Know  O Refused to Answer | O Yes: ______________  O No  O Don’t Know  O Refused to Answer |
| O Kufanywa usiwe kama binadamu na kuaibishwa mbele ya watu  Belittled or humiliated you in front of other people | O Often  O Sometimes  O Don’t Know  O Refused to Answer | O Yes: ______________  O No  O Don’t Know  O Refused to Answer |
| O Kufanya kitu ya kukushtua kwa kupenda kwake (vile alikutazama, kukupigia kelele, kuvunja vitu na kadhalalika)  Done anything to scare or intimidate you on purpose (by the way he looked at you, by yelling, smashing things, etc.) | O Often  O Sometimes  O Don’t Know  O Refused to Answer | O Yes: ______________  O No  O Don’t Know  O Refused to Answer |
| O Kutishia kukuumiza au mtu mwengine unayemjali  Threatened to hurt you or someone you care about | O Often  O Sometimes  O Don’t Know  O Refused to Answer | O Yes: ______________  O No  O Don’t Know  O Refused to Answer |
| O Kuzabwa kofi, au kukutupia kitu yenye inaweza kukuumiza  Slapped, hit, or thrown something at you that could hurt you | O Often  O Sometimes  O Don’t Know  O Refused to Answer | O Yes: ______________  O No  O Don’t Know  O Refused to Answer |
| O Kukuskuma au kukububurusha  Pushed or shoved you | O Often  O Sometimes  O Don’t Know  O Refused to Answer | O Yes: ______________  O No  O Don’t Know  O Refused to Answer |
| O Kukupiga teke, kukuvuta au kukupiga.  Kicked, dragged, or beaten you | O Often  O Sometimes  O Don’t Know  O Refused to Answer | O Yes: ______________  O No  O Don’t Know  O Refused to Answer |
| O kukunyonga au kukuchoma kwa kwakusudia  Strangled or burnt you on purpose | O Often  O Sometimes  O Don’t Know  O Refused to Answer | O Yes: ______________  O No  O Don’t Know  O Refused to Answer |
| O Kukutishia ama ashatumia bastola, kwa hakika, kisu ama kifaa chochote yenye yaweza kukuumiza.  Threatened or has actually used a gun, knife, or other weapon that could hurt you | O Often  O Sometimes  O Don’t Know  O Refused to Answer | O Yes: ______________  O No  O Don’t Know  O Refused to Answer |
| O Kukushika shika kwa njia isiyofaa ama kukugusa kwa njia ambayo ilikufanya ujisikie hauko salama  Fondled, groped, grabbed, or touched you in a way that was unwanted or made you feel unsafe | O Often  O Sometimes  O Don’t Know  O Refused to Answer | O Yes: ______________  O No  O Don’t Know  O Refused to Answer |
| O Alikulazimisha kufanya ngono kwa nguvu wakati hakutaka au bila idhini (kwa mfano ulikuwa mlevi au ulikuwa mlevi wa kupindukia)  Forced you to have sex when you did not want to or could not provide consent (ex. you were too drunk or passed out) | O Often  O Sometimes  O Don’t Know  O Refused to Answer | O Yes: ______________  O No  O Don’t Know  O Refused to Answer |
| O None (skip to GBV04) | Not Applicable | Not Applicable |
| O Refused to Answer (skip to GBV04) | Not Applicable | Not Applicable |

| GBV04:  Je, kwa miezi 12 iliyopita mshiriki wako asiye mkuu amewahi kukufanyia vitu zifuatavyo?  In the past 12 months has your **NON-PRIMARY** sexual partner done any of the following to you?  *Interviewer: Read each option aloud one at a time and choose all that apply.* | GBV05:  Kwa muda wa miezi 12 iliyopita,je mshiriki wako asiye wa karibu amekufanyia haya kila mara au mara zingine tu?  In the past 12 months, has your **NON-PRIMARY** partner done this often or only sometimes? | GBV06:  Je ulielezea mtu y kuwa mshiriki wako asiye wa karibu alikufanyia haya na ikiwa ulieleza ni nani ulielezea?  Did you ever tell anyone that your **NON-PRIMARY** partner did this, and if so who did you tell? |
| --- | --- | --- |
| O Insulted or made you feel bad about yourself  Alikutusi au kukufanya hujihisi vibaya. | O Often  O Sometimes  O Don’t Know  O Refused to Answer | O Yes: ______________  O No  O Don’t Know  O Refused to Answer |
| O Belittled or humiliated you in front of other people  Kufanywa usiwe kama binadamu na kukuaibisha mbele ya watu. | O Often  O Sometimes  O Don’t Know  O Refused to Answer | O Yes: ______________  O No  O Don’t Know  O Refused to Answer |
| O Done anything to scare or intimidate you on purpose (by the way he looked at you, by yelling, smashing things, etc.)  Kufanya kitu ya kukushtua kwa kusudia ( vile alivyo kutizama, kukupigia kelele , kuvunja vitu na kadhalalika) | O Often  O Sometimes  O Don’t Know  O Refused to Answer | O Yes: ______________  O No  O Don’t Know  O Refused to Answer |
| O Threatened to hurt you or someone you care about  Kutishia kukuumiza au mtu mwengine unaye mjali. | O Often  O Sometimes  O Don’t Know  O Refused to Answer | O Yes: ______________  O No  O Don’t Know  O Refused to Answer |
| O Slapped, hit, or thrown something at you that could hurt you  Kukuzaba kofi, au kukutupia kitu kinachoweza kukuumiza. | O Often  O Sometimes  O Don’t Know  O Refused to Answer | O Yes: ______________  O No  O Don’t Know  O Refused to Answer |
| O Pushed or shoved you  Kukuskuma au kukububurusha . | O Often  O Sometimes  O Don’t Know  O Refused to Answer | O Yes: ______________  O No  O Don’t Know  O Refused to Answer |
| O Kicked, dragged, or beaten you  Kukupiga teke, kukuvuta au kukupiga. | O Often  O Sometimes  O Don’t Know  O Refused to Answer | O Yes: ______________  O No  O Don’t Know  O Refused to Answer |
| O Strangled or burnt you on purpose  kukunyonga au kukuchoma kwa kwakusudia . | O Often  O Sometimes  O Don’t Know  O Refused to Answer | O Yes: ______________  O No  O Don’t Know  O Refused to Answer |
| O Threatened or has actually used a gun, knife, or other weapon that could hurt you  Kukutishia ama ashatumia bastola, kwa hakika , kisu ama kifaa chochote yenye yaweza kukuumiza. | O Often  O Sometimes  O Don’t Know  O Refused to Answer | O Yes: ______________  O No  O Don’t Know  O Refused to Answer |
| O Fondled, groped, grabbed, or touched you in a way that was unwanted or made you feel unsafe  Kukushika shika kwa njia isiyofaa ama kukugusa kwa njia ambayo ilikufanya ujisikie hauko salama | O Often  O Sometimes  O Don’t Know  O Refused to Answer | O Yes: ______________  O No  O Don’t Know  O Refused to Answer |
| O Forced you to have sex when you did not want to or could not provide consent (ex. you were too drunk or passed out)  Alikulazimisha kufanya ngono kwa nguvu wakati hakutaka au bila idhini ( kwa mfano ulikuwa mlevi au ulikuwa mlevi wa kupindukia). | O Often  O Sometimes  O Don’t Know  O Refused to Answer | O Yes: ______________  O No  O Don’t Know  O Refused to Answer |
| O None (skip to GBV04) | Not Applicable | Not Applicable |
| O Refused to Answer (skip to GBV04) | Not Applicable | Not Applicable |

| **NOTES** |
| --- |
|  |

**END OF BASELINE QUESTIONNAIRE**
