# Supplementary material for: Adoption of HIV pre-exposure prophylaxis among women at high risk of HIV infection in Kenya
Source: PLoS One. 2022 Sep 9;17(9):e0273409. doi: 10.1371/journal.pone.0273409 (PMC9462728; doi:10.1371/journal.pone.0273409)
Supplement: S4 File — (DOC) [file pone.0273409.s004.doc]

**6-monthly follow-up visit questionnaire**

| **Section A1** | | |
| --- | --- | --- |
| **No.** | **Question** | **Responses** |
| Intid | Interviewer ID | ___ ___ |
| date | Date of Interview | ___ ___/___ ___ ___/ ___ ___ ___ ___  D D M M M Y Y Y Y |
| Cluster id | Enter Cluster ID | ___ ___ ___ |
| cluster | Enter Name of Cluster |  |
| location | Enter Name of Hotspot or Beach |  |
| arm | Intervention or control cluster? | O Intervention  O Control |
| month | What month of follow-up is this appointment? | O Month 6  O Month 12  O Month 18  O Month 24 |
| **Section A2: Participant Identification** | | |
| pid | *Enter the participant’s ID number. Be extremely careful when entering the number. Double and triple check that the number is correct before proceeding.* | ___ ___ ___ ___ ___ ___ ___ ___ ___ |
| pid2 | Verify the PID number | ___ ___ ___ ___ ___ ___ ___ ___ ___ |

| **Section B: Demographic questions (DMG)** | | |
| --- | --- | --- |
| **No.** | **Question** | **Coding** |
| DMG01 | I’d like to ask you again, what is your age?  Daher mar penji kendo, in ja higni adi? | ___ ___  O Refused to Answer |
| DMG02 | Have you permanently moved or relocated away from this study area in the past 6 months?  Bende isewuok kata dar chuth e aluora mar kama itimoe nonro kuom dweche auchiel ma osekalo?  *Interviewer: ensure that “study area” is clearly explained to mean the cluster/location where the participant was enrolled* | O Yes  O No 🡪 Skip to DMG04  O Refused to Answer 🡪 Skip to DMG04 |
| DMG03 | Where do you reside now?  Idak Kanye e kindeni? | Cluster code: ___ ___ ___  O Outside of study area: __________________  O Don’t Know  O Refused to Answer |
| DMG04 | Besides Jikinge, have you enrolled in any other research studies in the past 6 months where you were receiving an intervention such as income, medicine, HIV test kits, condoms, or counseling? Your answer to this question will not impact your continued participation in the Jikinge study.  Ka opogore kod JIKINGE, bende isebet jachiwre e nonro moro amoro kuom dueche auchiel mosekalo mane iyudoe kony kaka omenda, yath, gik pim mar kute mag ayaki, rabo yunga, kata hocho? Duoko ma ichiwo e penjoni ok bikelo lokruok e chiwruokni e nonro mar JIKINGE. | O Yes  O No 🡪 Skip to DMG06  O Don’t Know 🡪 Skip to DMG06  O Refused to Answer 🡪 Skip to DMG06 |
| DMG05 | What did you receive as part of your participation in the other HIV research study?  Gin gik mane iyudo kuom chiwruokni e nonro mar kute mag Ayaki machielono?  Choose all that apply. | O HIV test kits  O Condoms and/or lubricant  O PrEP  O Cash transfers  O Other (specify): _________________________  O Don’t Know  O Refused to Answer |
| DMG06 | What is your current marital status?  Kit Keny mari en mane sani? | O Married, Living Together  O Married, NOT Living Together  O Not Married, Living Together  O Relationship but Not Married, NOT Living Together (boyfriend, etc.)  O Single  O Divorced  O Widowed  O Don’t Know  O Refused to Answer |
| DMG07 | Has your marital status changed in the last 6 months?  Bende kit keny mari oselokore kuom dweche auchiel ma osekalo? | O No change 🡪Skip to DMG10  O I am no longer in a relationship  O I am now married  O I am now in a relationship but not married  O I am still married, but now live with my husband  O I am still married or in a relationship, but no longer live with my partner (separated)  O I have divorced  O I have become a widow 🡪Skip to DMG10  O Refused to Answer |
| DMG08 | Was the change in your marriage related to HIV testing in any way, such as finding out your HIV status or a partner’s HIV status?  Bende lokruok mar kenyni ne otudore kod pimo kute mag Ayaki e yo moro amora, kaka ng’eyo chal mari kata chal mar jaherani?  Choose all that apply. | O Yes, my HIV status  O Yes, my partner’s HIV status  O Yes, partner’s refusal to test for HIV  O Yes, other (specify)  O No  O Don’t Know  O Refused to Answer |
| DMG09 | Do you think your participation in Jikinge study played some role in the change in your marital status?  Bende iparo ni chiwruokni e nonro mar JIKINGE ne nyalo kelo lokruok e kit keny mari? | O Yes (specify)  O No  O Don’t Know  O Refused to Answer |
| DMG10 | What is your **primary** source of income?  Yoo maduong’ mari mar yuto en mane? | O Professional/salaried  O Rental income (landlord, rent equipment)  O Sales and Service (Non-Fish)  O Skilled Manual  O Unskilled Manual  O Domestic Service  O Agriculture  O Fishing/ Fish Trade  O Sex Work  O Informal/Seasonal/ Piece Work  O Student  O Unemployed  O Other (specify)  98 Don’t Know  99 Refused to Answer |
| DMG11 | What is/are your OTHER source(s) of income, if any?  Yori machielo mimedo gi yori maduong’mar yuto en mane, ka nitie?  *Interviewer: mark all that apply* | O Professional/salaried  O Rental income (landlord, rent equipment)  O Sales and Service (Non-Fish)  O Skilled Manual  O Unskilled Manual  O Domestic Service  O Agriculture  O Fishing/ Fish Trade  O Sex Work  O Informal/Seasonal/ Piece Work  O Student  O Other (specify)  O None/ Not Applicable  98 Don’t Know  99 Refused to Answer |
| DMG12 | How much income do you typically earn in one month, from all sources?  En omenda marom nade ma iyudo e dwe ka dwe kao kuom yoreni duto mag yuto?  *Interviewer: Ask participant to give best estimate if not sure* | KES ___________________  O Don’t Know  O Refused to Answer |

| **Section C1: General Health (HLT)** | | |
| --- | --- | --- |
| *Interviewer:* I have some questions about your physical and mental health.  Antie gi penjo moko kuom chal mar ngimani kuom ngimani mar dendi kod pachi | | |
| **No.** | **Question** | **Coding** |
| HLT01 | How would you rate your overall health?  Ere kaka inyalo pimo chal mar ngimani kanyakla? | O Very Good  O Good  O Fair  O Poor  O Don’t Know  O Refused to Answer |
| HLT02 | In the past 6 months, what is your best estimate of how often you drank alcohol?  E dweche 6 mokalo, inyalo pimo nadi kwan mar ndalo ma ne imadho kong’o? | O Never (skip to HLT04)  O Less than once per month  O Once a month  O 2 to 3 times per month  O Once per week  O Twice per week  O 3 to 4 times a week  O 5 to 6 times per week  O Every Day  O Don’t Know  O Refused to Answer |
| HLT03 | In the past month, how often did you drink 5 or more alcoholic drinks in one night?  Ei dwe mokalo, nyadidi mane imadhoe kong’o chupe madirom abich otieno achiel? | O Never  O Only once  O 2 or 3 times  O Once per week  O Twice per week  O 3 to 4 times a week  O 5 to 6 times per week  O Every Day  O Don’t Know  O Refused to Answer |
| HLT04 | In the past 6 months, have you become pregnant?  Kuom dweche auchiel ma osekalo, bende isegamo remo? | O Yes  O No  O Don’t Know  O Refused to Answer |

| **Section C2: Mental health (PHQ-9)**  ***Skip this section if month=6 or 18; ask only if month=12 or 24*** | |
| --- | --- |
| HLT05:  Over the last 2 weeks, have you been bothered by any of the following problems?  Ei jumbe ariyo mokalo, be weche machalo kod magi osechando pachi?    O No 🡪 skip to next item  O Yes 🡪 ask HLT06  O Don’t know 🡪 skip to next item  O Refused to answer 🡪 skip to next item  ***Interviewer:****Read each item aloud one at a time and read out the 5 frequency choices for each item.* | HLT 06:  Over the last 2 weeks, how often have you been bothered by this problem?  Ei jumbe ariyo mokalo, nyadidi ma weche machalo kod magi osechando pachi?  ***Interviewer:*** *Read each item aloud one at a time and read out the 5 frequency choices:* One Day, Less than Half the Days, Around Half the Days, More than Half the Days, Every Day  O One Day  O Less than Half the Days  O Around Half the Days  O More than Half the Days  O Every Day  O Don’t Know  O Refused to Answer |
| 1. Had little interest or pleasure in doing things  Ne ingi gombo matin kata siso mar timo gik moko |  |
| 2. Felt down, depressed, or hopeless  Ne ikuyo, ingi chuny machandore, kendo ionge geno. |  |
| 3. Had trouble falling asleep/staying asleep, or sleeping too much  Ne ok inyal chako nindo, kata nindo moyware kata ne inindo mangeny |  |
| 4. Felt tired or having little energy  Ne iwinjo ka iol kata ionge teko |  |
| 5. Had poor appetite or overeating  Ne iwinjo ka iol kata ionge teko |  |
| 6. Felt bad about yourself, that you are a failure, or felt that you let yourself or your family down  Ne iwinjo marach in iwuon kata ni ok ichopo dwachi kata ne iwinjo ni ikelo wichkuot ne in iwuon kata anyuolani |  |
| 7. Had trouble concentrating on things such as reading the newspaper or watching television  Ne ingi chandruok e keto pachi e gik moko kaka somo oboke mar weche manyien kata neno televisen. |  |
| 8.Moved or spoken so slowly that other people could have noticed, or been so fidgety/restless that you have moved around a lot more than usual  Ne idar kata iwuoyo mos ma jomamoko ne nyalo fwenyo ni ingi luoro kata ionge kwe kata wuodhi ngeny ma ok kaka pile |  |
| 9.Had thoughts that you would be better off dead, or of hurting yourself in some way  Ne ingi paro ni nyalo bet maber ka itho, kata kelo hinyruok ne in e yoo moro amora. |  |

| HLT07 | How difficult have these problems made it for you to do your work, take care of things at home, or get along with other people?  Chandruokgi osekeloni pek marom nadi e timo tijeni, rito gikmoko e dala, kata winjruok gi jok ma moko? | O Not Difficult at All  O Somewhat Difficult  O Very Difficult  O Don’t Know  O Refused to Answer |
| --- | --- | --- |

| **Section C3: General Sexual Activity (GSA)**  Interviewer: Now I have some questions about your sexual activity, in order to get a better understanding of some important aspects of your life. For these questions, “sexual activity” is defined as sexual penetration of the vagina or anus.  Interviewer: Koro an kod penjo moko e wi yoreni mag bet e I achiel e ringruok, mondo abedgi ng’eyo matut e wii moko kuom gik madongo e kik ngimani. Ne penjogi “yore mag bet e achiel e ringruok” ohulo ni soyo duong’ dichuo e duong’ miyo kata olunde | | |
| --- | --- | --- |
| GSA01 | During the past month, how many different sexual partners have you had?  E dwe ma okalo, gin johera mopogore opogore adi ma isebedogo e achiel e ringruok?  Enter 998: For DON’T KNOW, Enter 999: REFUSED TO ANSWER | ___ ___  O Don’t Know  O Refused to Answer |
| GSA02 | The last time you had sex, did you use a condom?  Chieng mogik mane ibet achiel e ringruok, be ne itiyo gi rabo yunga? | O Yes  O No  O Don’t Know  O Refused to Answer |

| **Section C4: Primary Partner (PRP)**  Interviewer: Now I have some questions about your **one** primary or regular sexual partner. A primary partner could be your husband, boyfriend, or someone you **regularly** have sex with. A primary partner is the ONE partner you feel like you love or like the most, or the one you can confide in the most. If you would like help deciding who your primary partner is, we can talk about that now.  Sani koro antie gi penjo moko kuom jaherani **achiel** mahie kat jaherani mapile. Jaherani mahie nyalo bedo jaodi, osiepni mawuoyi, kata ng’atno ma ijabedogago e achiel eringrook **mapile. Jaherani** mahie en e jaherani ma ineno ka ihero moloyo gi te, kata ng’atno ma inyalo pimo ne wach mopondo machando chunyi ahinya, Ka dipo ni diher mondo okonyi ng’eyo ni jaherani mahie en ng’a to mondo wawachi sani | | |
| --- | --- | --- |
| PRP01 | Do you currently have a primary partner, or have you had one in the past month?  Bende in kod johera mahie e kindeni, bende nyocha in kod moro e dwe ma okalo?Ma e ng’at ma wadwaro wuoyo e wiyi e penjo ma luwegi.  This is the man we will discuss in the following questions. | O Yes  O No (skip to NPP01)  O Refused to Answer (skip to NPP01) |
| PRP02 | Was this person also your primary partner 6 months ago?  Ng’atni be ne en jaherani mahie dwech 6 mokalo? | O Yes (skip to PRP05)  O No  O Refused to Answer |
| PRP03 | About how old is your current primary partner?  Jaherani mahie ma in go sani nyalo bedo jahigni adi?  *Interviewer: if participant is not sure, ask her to give best guess* | ___ ___ years  O Don’t Know  O Refused to Answer |
| PRP04 | How long have you had a sexual relationship with your current primary partner?  Isebedo e tudruok mar bedo e achiel e ringruok kod jaherani mahie e kinde ma rom nade?  *Interviewer: For relationships <1 month, record 01 months. (Ex: If participant says one week, record 01months)* | ___ ___ years, ___ ___ months  O Don’t Know  O Refused to Answer |
| PRP05 | Have you had sex with this partner in the past month?  Bende isebet e achiel e ringruok kod ng’atni e dwe ma okalo? | O Yes  O No 🡪Skip to PRP09  O Refused to Answer 🡪Skip to PRP09 |
| PRP06 | In the past month, how often did you use condoms with your primary partner?  Edwe ma okalo, nyadidi mane itiyo kod rabo yunga kod jaherani mahie? | O Never  O Less than Half the Time  O About Half the Time  O More than Half of the Time  O Always (skip to PRP08)  O Don’t Know  O Refused to Answer |
| PRP07 | Why did you not use condoms all the time with your primary partner within the last month? Please tell me all the reasons why.  E dwe mokalo ang’o ma omiyo ne ok utiyo gi rabo yunga e kindego duto gi jaherani mahie? Akwayi ni mondo iwachna gigo duto ma omiyo.  *Interviewer choose all that apply based on what the participant mentioned.* | O I Did Not Want To  O Partner Did Not Want To  O No Condom was Available  O I Am Trying To Become Pregnant  O I Have Another Form of Contraception (IUD, Implant, Injectable, Pill, etc.)  O I Knew My Primary Partner’s HIV Status and decided a condom was not necessary  O I trust my primary partner  O One or both of us is using PrEP  O Other (specify): __________________________  98 Don’t Know  99 Refused to Answer |
| PRP08 | In the past month, have you ever had difficulty *negotiating* condom use with your primary partner?  E dwe mokalo, be isegabedo kod pek wuoyo kod jaherani ma hie mondo uti kod rabo yunga? | O Yes, always  O Yes, sometimes  O I have no difficulty negotiating  O I never try to get my partner to use condoms  O Don’t Know  O Refused to Answer |
| PRP09 | In the past 6 months, has your primary partner had an HIV test?  Kuom dweche auchiel ma okalo, bende jaherani mahie osepimo kute mag Ayaki? | O Yes  O No 🡪 Skip to PRP11  O Don’t Know 🡪 Skip to PRP11  O Refused to Answer 🡪 Skip to PRP11 |
| PRP10 | Where did your partner get tested for HIV?  En Kanye ma jaherani nene opimoe kute mag Ayaki?  *Interviewer:* ***remind participant that HIV self-testing is also a possible answer to this question****. Mark all that apply.* | O VCT clinic  O Hospital  O HIV self-test  O Home Testing  O Other (specify): _____________  O Don’t Know  O Refused to Answer |
| PRP11 | **Ask if arm= “Intervention” OR if response to PRP10 is “HIV self-test”**  Did you offer your partner an HIV self-test in the past 6 months?  Bende nene ichiwo ne jaherani gir pim ma nga’to pimorego kende owuon e kinde mar dweche auchiel ma okalo? | O Yes  O No 🡪 Skip to PRP21  O Don’t know 🡪 Skip to PRP21  O Refused to answer 🡪 Skip to PRP21 |
| PRP12 | Did your partner accept the self-test from you?  Bende jaherani nene orwako gir pim ma nga’to pimorego kende owuon kane imiye? | O Yes  O No 🡪 skip to PRP21  O Refused to Answer 🡪 skip to PRP21 |
| PRP13 | When did your partner use the self-test most recently?  En karang’o machiegni mane jaherani otiyoe kod gir pim ma nga’to pimorego kende owuon?  *Interviewer: If partner used multiple HIV self-tests, record the time the most recent test was used*. | O Did not use 🡪 Skip to PRP21  O Less than 1 week ago  O Between 1 week and 1 month ago  O Between 1 and 3 months ago  O Between 3 and 6 months ago |
| PRP14 | Where did your partner use the self-test?  En Kanye mane jaherani otiyoe kod gir pim ma nga’to pimorego kende owuon? | O At our home  O At my home  O At his/her home  O At workplace  O At friend’s house  O Lodge/hotel room  O In or around a bar (car outside, alleyway,etc.)  O Other (specify): __________________________  O Don’t know  O Refused to answer |
| PRP15 | Please rate how easy or difficult it was for your partner to use the self-test? This could be based on what he told you, or what you directly observed.  Bende inyalo pimonwa pek kata yot mane jaherani obedogo e tiyo gir pim ma nga’to pimorego kende owuon? Maendi nyalo bedo gimane onyisi kata ne ineno iwuon  *INTERVIEWER: First get participant’s reaction and then read the choices that are most relevant and ask the participant which one fits best. This question is about use of self-test, not about acceptance of the self-test.* | O Very easy  O Somewhat easy  O Neutral  O Somewhat difficult  O Very difficult  O Don’t know  O Refused to answer |
| PRP16 | Were you present when your partner used the self-test?  Bende ne intie kama ne jaherani pimore kod gir pim ma nga’to pimorego kende owuon? | O YES – I was present when he used the self-test  O NO – But he told me he used the self-test  O Don’t know  O Refused to answer |
| PRP17 | Did you learn your primary partner’s HIV status following the self-test?  Bende ne ing’eyo chal mar jaherani mahie ka okalo e pimo ma ngato timo kende owuon? | O No  O Yes, Partner is Positive  O Yes, Partner is Negative 🡪 Skip to PRP20  O Yes, Refuse to Disclose Partner’s Status  O Refused to Answer |
| PRP18 | Did your partner go to a clinic or health facility in order to confirm the result that was obtained from the self-test (confirmatory testing)?  Bende jaherani ne odhi e kar thieth mondo onon duoko mar pim ma nga’to pimore kende owuon? | O Yes  O No  O Don’t know  O Refused to answer |
| PRP19 | Did your partner go to a clinic or health facility for HIV care?  Bende jaherani ne odhi e kar thieth mondo oyud kony ma imiyo joma oyud kod kute mag Ayaki? | O Yes  O No  O Don’t know  O Refused to answer |
| PRP20 | At the **same** time that your partner used the self-test, did you **also** use a self-test?  E seche mane jaherani otiyo kod gir pim ma nga’to pimorego kende owuon, in bende ne ipimori kod gir pim ma nga’ato pimorego kende owuon? | O Yes, we tested together at the same time  O Yes, we both tested but not together  O No  O Other (specify): ______________________________  O Don’t know  O Refused to answer |
| ***Note: PRP21- PRP24 should be asked only if arm= “Control”*** | | |
| PRP21 | **Control group:**  Did you give your partner a VCT referral card in the past 6 months?  Bende ne imiyo jaherani kad mar gwelo kuom dweche auchiel ma okalo? | O Yes  O No 🡪 Skip to PRP25  O Don’t know 🡪 Skip to PRP25  O Refused to answer 🡪 Skip to PRP25 |
| PRP22 | **Control group**:  Did your partner go to a clinic or health facility for HIV care?  Bende ne imiyo jaherani kad mar gwelo kuom dweche auchiel ma okalo? | O Yes  O No 🡪 skip to PRP25  O Don’t Know 🡪 skip to PRP25  O Refused to answer 🡪 skip to PRP25 |
| PRP23 | **Control group**:  At the **same** time that your partner got tested for HIV, did you also get tested?  Eseche mane jeherani opimore, in bende ne ipimori? | O Yes, we tested together at the same time  O Yes, we both tested but not together  O No  O Other (specify): ___________________________  O Don’t know  O Refused to answer |
| PRP24 | **Control group**:  Did you learn your primary partner’s HIV status following the HIV test?  Bende ne inge’eyo chal mar jaherani mahie mar kute mag Ayaki ka oluwore kod pimno? | O No  O Yes, Partner is Positive  O Yes, Partner is Negative 🡪 Skip to PRP25  O Yes, Refuse to Disclose Partner’s Status  O Refused to Answer |
| PRP25 | In the past 6 months, has there been a time when you took an HIV test and shared the result with your partner?  Kuom dweche auchiel ma okalo, bende nitiere thuolo mane upimoru kod jaherani ma ineno duokone en bende oneno duokoni? | O Yes  O No  O Don’t Know  O Refused to answer |
| PRP26 | Did your sexual behavior with your partner change in the past 6 months?  Edweche 6 mokalo Bende ne ichungo bedo e achiel e ringruok gi jaherani ni kuom ndalo mogwaro? | O Yes  O No 🡪 Skip to NPP01  O Don’t know  O Refused to answer 🡪 Skip to NPP01 |
| PRP27 | In the past 6 months, have you stopped having sex with this partner for extended periods of time?  Bende ne ichungo bedo e achiel e ringruok gi jaherani ni kuom ndalo mogwaro?  *Interviewer: this should not include cases where one person was away for multiple weeks* | O Yes  O No  O Don’t Know  O Refused to Answer |
| PRP28 | How else has your sexual behavior with this person changed in the past 6 months?  Ere kaka timbeni mag kisera kod nga’tni ne olokore kuom dweche auchiel ma okalo?  *Mark all that apply.* | O Increased Condom Use  O Decreased Condom Use  O Increase amount or frequency of sex  O Decrease amount or frequency of sex  O Other, specify (Open Ended Response): |
| **Section C5: Non-Primary Partners (NPP)**  Interviewer: Now I have some questions about any other sexual partners you may have had other than your primary partner. For these questions, I am referring to boyfriends, casual sexual partners, or someone with whom you engage in transactional sex, i.e. sex for money, goods gifts, food or housing.  Interviewer: Koro an kod penjo moko ewi jomoko ma ubetgago achiel e ringruok ka opogre gi jaherani ma hie. Ne penjogi, Awuoyo ewi osiepeni ma chuo ma ibetgo e achiel e ringruok, johera ma iromogo dichiel to ibetgo e acheil e ringruok, kata nga’t ma ibetgo e acheil e ringruok nikech chudo, i.e bet e achiel e ringruok ne pesa, mwandu, chiemo kata ot ma idakie. | | |
| NPP01 | How many non-primary sexual partners have you had in the past month?  E dwe ma okalo, ne ibet e achiel e ringruok kod johera ma nyalo romo adi ma ok gin joherani mahie?    *Interviewer: probe for an estimate if participant cannot immediately recall* | ___ ___ (If 00, skip to TRX01)  98 Don’t Know  99 Refused to Answer (skip to TRX01) |
| NPP02 | Of these, approximately how many did you have **multiple** sexual encounters with in the past month?  E dwe ma okalo, ne ibet e achiel e ringruok kod johera ma nyalo romo adi ma ok gin joherani mahie, kendo ibet kodgi e ringruok **ding’eny**? | ___ ___ (If 00, skip to NPP05)  O Don’t Know  O Refused to Answer |
| NPP03 | Among those with whom you had multiple sexual encounters in the past month how often did you use condoms?  Kuom jogo ma ne ibetgo e achiel e ringruok ding’eny e dwe ma okalo, nyadidi mane itiyo kod rabo yunga? | O Never  O Less than Half the Time  O About Half the Time  O More than Half of the Time  O Always (skip to NPP05)  O Don’t Know  O Refused to Answer |
| NPP04 | Why did you not always use condoms with those you shared multiple sexual encounters?  E dwe mokalo ang’o momiyo ne ok utiyo gi rabo yunga gi johera ma ok mahie mane ubetgo achiel e ringruok ding’eny?  *Open ended for participants. Interviewer choose all that apply based on what the participant mentioned.* | O I Did Not Want To  O Partner(s) Did Not Want To  O No Condom Was Available  O I Am Trying to Become Pregnant  O I Have Another Form of Contraception (IUD, Implant, Injectable, Pill, etc.)  O I Knew My Non-Primary Partner’s HIV Status  O I trust my non-primary partner(s)  O Partner offered me more money to not use a condom  O Other (specify)  O Don’t Know  O Refused to Answer |
| NPP05 | Approximately how many non-primary sexual partners in the past month did you have sex with **only once**?  Gin madirom johera adi ma ok gin joherani mahie ma ibetgago e achiel e ringruok kata johera moko ma ok ihiny betgo e achiel e ringruok mane ibetgo e achiel e ringruok dichiel kende e dwe mokalo? | ___ ___ (If 00, skip to NPP08)  O Don’t Know  O Refused to Answer |
| NPP06 | Among the men with whom you had only one sexual encounter in the past month, how often did you use condoms?  E kind chuo mane ibetgo e achiel e ringrouk dichiel kende e dwe ma okalo, ne itiyo kod rabo yunga mang’eny marom nadi ka ne ibete achiel e ringruok kodgi? | O Never  O Less than Half the Time  O About Half the Time  O More than Half of the Time  O Always (skip to NPP08)  O Don’t Know  O Refused to Answer |
| NPP07 | Why did you not always use condoms with those you had only one sexual encounter?  En ang’o mamiyo ne ok itiyo gi rabo yunga kod jogo mane iromogo kinde mane ibet e achiel e ringruok dichiel kende?  *Open ended for participants. Interviewer choose all that apply based on what participant mentioned.* | 1: I Did Not Want To  2: Partner(s) Did Not Want To  3. No Condom Was Available  4: I Am Trying To Become Pregnant  5: I Have Another Form of Contraception (IUD, Implant, Injectable, Pill, etc.)  6: I Knew My Non-Primary Partner/Partners’ HIV Status  7: I trust my non-primary partner(s)  8: Partner(s) offered me more money to not use a condom  9: Other (specify)  98: Don’t Know  99: Refused to Answer |
| Interviewer: I will now be asking you about all of the non-primary partners you have had in the past 6 months.  Koro abiro penji ewi joherani duto te ma ok gin johera mahie ma isebedogo e dweche 6 mokalo | | |
| NPP08 | Among the non-primary partners you have had in the past **6 MONTHS**, have you had sex without a condom with any of them?  Kuom joherani ma ok mahie ma isebogo **e dweche auchiel** ma okalo, bende isebet e achiel e ringruok kod moro amora kuomgi ma ok itiyo kod Rabo Oyunga? | O Yes  O No 🡪 Skip to NPP10 or NPP11  O Refused to answer 🡪 Skip to NPP10 or NPP11 |
| NPP09 | Among these men, have there been some whose HIV status you did **not** know?  Kuom joheragi, bende nitiere moko kuomgi mane ok inge’eyo chalgi mar kute mag Ayaki? | O Yes  O No  O Refused to answer |
| NPP10 | ***Ask only if arm= “Intervention”***  Did you offer a self-test to **any** of your non-primary partners in the past 6 months?  Bende ne ichiwo gir pim ma nga’to pimorego kende owuon ne joherani ma ok mahie moro amora e dweche 6 mokalo? | O Yes  O No  O Don’t know  O Refused to answer |
| NPP11 | ***Ask only if arm= “Control”***  Did you offer a VCT referral card to **any** of your non-primary partners in the past 6 months?  Bende ne ichiwo kadi mar ote ne moro amora kuom joherani ma ok mahie e dweche molalo? | O Yes  O No  O Don’t know  O Refused to answer |
| NPP12 | In the past 6 months, did you find out directly or indirectly that any of your non-primary partners tested for HIV and obtained an HIV-positive result?  E dweche auchiel ma okalo, bende ne inge’eyo ayanga koso e yoo ma opondo ni jaherani ma ok mahie ne oyud kod kute mag Ayaki?  *Interviewer*: give various examples of how this could happen | O Yes  O No  O Don’t know  O Refused to answer |
| NPP13 | How many non-primary partners of yours tested HIV-positive in the past 6 months.  Gin joherani ma ok mahie adi mane oyud kod kute mag Ayaki e dweche auchiel ma okalo? | Number ___ ___  O Don’t know  O Refused to answer |

| **Section C6: Transactional Sex (TRX)**  Interviewer: I would now like to ask questions about any sexual partners you may have had who paid you for sex or gave you something in exchange for sex. These may be men with whom you had only one sexual encounter, or men with whom you had multiple sexual encounters. Some of these questions may sound similar to ones I just asked, but please think only about those encounters where you exchanged sex.  Koro daher penji penjo e wi johera mane nyalo bet ni ochuli pesa kata omiyi gimoro nikech ibet kodgi e achiel e ringruok. Magi nyalo bet chou mane ibetgo e achiel e ringruok dichiel kende, kata chuo mane ibetgo e achiel e rinruok ding’eny. Moko kuom penjogi nyalo nenore ni chalre gi moko ma asepenjo, to kata kamano par mana kuom ndalo mane ibet e achiel e ringruok mondo ochuli. | | |
| --- | --- | --- |
| TRX01 | In the past 6 months, have you exchanged sex for money, goods, food, housing or services?  Kuom dweche auchiel ma osekalo,bende isega loko bet e achiel e ringruok mondo ochuli, omiyi muandu, otimni gimoro kata mondo omi gimoro timre? | O Yes  O No (Skip to TRX18)  O Refused to Answer (Skip to TRX18) |
| TRX02 | In the past month, with approximately how many men did you exchange sex?  E dwe ma okalo, ne ibet e achiel e ringruok kod chuo madirom adi nikech chudo? | _________ (If 0, skip to TRX18)  O Don’t Know  O Refused to Answer |
| TRX03 | In the past month, have you exchanged sex for **money**?  E dwe mokalo, bende isebedo e achiel e ringruok nikech idwaro pesa? | O Yes  O No (skip to TRX07)  O Refused to Answer (skip to TRX07) |
| TRX04 | In a typical month, how much of your income comes from exchanging sex for money?  E dwe achiel, en pesa maromo nade ma iyudoga ka owuok kuom bet e achiel e ringruok nikech chudo? | O None  O Less than half  O About half  O More than half, but not all  O All  O Don’t Know  O Refused to Answer |
| TRX05 | When you exchange sex for money, what is the average amount of money you charge per sexual encounter when a condom **is** used?  Ka ibet e achiel e ringruok nikech chudo to itiyo kod rabo oyunga, en pesa madirom adi ma idwaroga ni ochuli kuom sa asaya ma ibet e achiel e ringruok? | KES _________  O I Never Use a Condom  O Don’t Know  O Refused to Answer |
| TRX06 | When you exchange sex for money, what is the average amount of money per sexual encounter when a condom **is** **not** used?  Ka ibet e achiel e ringruok nikech chudo to ok itiyo kod rabo oyunga, en pesa madirom adi ma idwaroga ni ochuli kuom sa asaya ma ibet e achiel e ringruok? | KES _________  O I Always Use a Condom  O Don’t Know  O Refused to Answer |
| TRX07 | In the past month, have you exchanged sex for goods or services **other than** money?  E dwe ma okalo, bende isebet e achiel e ringruok mondo iyud mwandu kata kony ma moko to ok pesa? | O Yes  O No (skip to TRX10)  O Refused to Answer (skip to TRX10) |
| TRX09 | In the past month, what goods or services **other than** money have you exchanged for sex? Please tell me about all goods and services.  E dwe ma okalo, gin mwandu mage kata kony mage ma opogore kod pesa mane iyudo nikech bedo e achiel e ringruok? Akwayi ni iwachna ewi mwandugo duto kod kony.  *Choose all that apply.* | O Housing and/or utilities  O Food to eat  O Food to sell (example, fish)  O School fees  O To get a job, a work promotion, or to keep your job  O Other material goods (clothes, jewelry, makeup, electronics, etc.)  O Household items (soap, cleaning supplies, tools, etc.)  O Other (specify): __________  O Don’t Know  O Refused to Answer |
| TRX10 | ***Ask only if TRX03 and/or TRX07 = Yes***  In the past month, what is the approximate **total** *value* of the money, goods, or services you received in exchange for sex?  E dwe ma okalo, nyalo bedo nengo madirom pesa adi, mwandu kata kony madirom nade ma iyudo nikech bedo e achiel e ringruok?  *Interviewer: Make sure participant understands to include how much the non-monetary goods/services are worth.* | KES _______  O Don’t Know  O Refused to Answer |
| TRX11 | In the past 6 **MONTHS**, have you received money, goods, or services in exchange for sex **WITHOUT** a condom?  Kuom dweche auchiel ma okalo, bende iseyudo pesa, mwandu kata kony nikech loko bet e achiel e ringruok ka OK utiyo kod rabo oyunga? | O Yes  O No 🡪 Skip to TRX18  O Refused to Answer 🡪 Skip to TRX18 |
| TRX12 | Among the men with whom you had sex without a condom, did you know the HIV status of **ALL** these men?  Kuom chow mane ibetgo e achiel e ringruok ma ok itiyo kod rabo oyunga, bende ne inge’yo chalgi DUTO TE mar kute mag Ayaki  *Interviewer: Answer is “No” even if status of one partner was unknown.* | O Yes 🡪 Skip to TRX14  O No  O Refused to Answer 🡪 Skip to TRX14 |
| TRX13 | For how many men did you NOT know the HIV status?  Chwo adi mane ok inge’yo chalgi mar kute mag Ayak? | ___ ___ (number of men)  O Don’t know  O Refused to answer |
| TRX14 | How many of these men were known to be HIV-POSITVE?  Adi kuom chwogi mane ong’ere ni NITIE KOD KUTE MAG AYAKI | ___ ___ (number of men)  O Don’t know  O Refused to answer |
| TRX15 | ***Ask only if arm= “Intervention”***  In the past 6 months, have you **OFFERED** HIV self-tests to any of the men whom you exchanged sex for money, goods, or services?  Kuom dweche auchiel ma okalo, bende ISECHIWO gir pim ma nga’to pimorego owuon ne moko kuom chow mane ilokogo bet e achiel e ringruok nikech pesa, mwandu kata kony? | O Yes  O No  O Refused to Answer |
| TRX16 | ***Ask only if arm= “Control”***  In the past 6 months, have you **OFFERED** VCT referral cards to any of the men whom you exchanged sex for money, goods, or services?  Kuom dweche auchiel ma okalo, bende ISECHIWO kadi mar gwelo ne ne moko kuom chow mane ilokogo bet e achiel e ringruok nikech pesa, mwandu kata kony? | O Yes  O No  O Refused to Answer |
| TRX17 | Did you come to know the HIV status of any of these men specifically using the Jikinge tools  Bende ne ing’eyo chal mar moro amora kuom chuogi mana ka itiyo kod gik pim mag Jikinge?  *(Interviewer: the HIV self-test kits or the VCT referral cards)*? | O Yes  O No  O Refused to Answer |
| TRX18 | Over the last 6 months, has the number of partners you have had sex with for money, goods, or services changed?  Kuom dweche auchiel ma okalo, bende kwan mar johera ma isebetgo e achiel e ringruok nikech pesa, mwandu kata kony oselokore? | O No Change 🡪 Skip to Section D  O Increase 🡪 Do not ask TRX20  O Decrease 🡪 Skip to TRX20  O Don’t Know 🡪 Skip to Section D  O Refused to Answer 🡪 Skip to Section D |
| TRX19 | Why has the number of partners you had sex with in exchange for money, goods, or services increased?  En ang’o kwan mar johera ma isebetgo e achiel e ringruok nikech pesa, mwandu kata kony omedore? | *Open Ended:* |
| TRX20 | Why has the number of partners you had sex with in exchange for money, goods, or services decreased?  En ang’o ma kwan mar johera ma isebetgo e achiel e ringruok nikech pesa, mwandu kata kony odok chien? | *Open Ended:* |

| **Section D: Recent Transactional Encounters (RT#)** | | |
| --- | --- | --- |
| Interviewer: Now, I will be asking you a series of questions related to your recent sexual partners from whom you received money or something else in exchange for sex. I have asked some similar questions but now I will ask for some more details. I greatly appreciate your patience through the next series of questions. I want you to think about the most recent time you exchanged sex for money, goods, gift, food, or housing. I am going to ask you several questions about this exchange.  Koro adhi penji penjo moko maluwore gi bedoni e achiel e ringruok gijoherani, motimore machiegni, mane omiyi pesa kata gimoro machielo nikech bet e achiel e ringruok. Ne asepenji penjo moko machal kodgi to koro adwaro ng’eyo weche moko matut. Adwoko erokamano maduong, ne horuok ma idhi bedogo e penjo maluwe, kata obedo ni nyalo nenore ni onwoyogi. Adwaro ni mondo ipar kuom ndalo macheigni mogik mane ibet e acheil e ringruok ne pesa, mwandu, mich, chiemo kata kar dak. Adhi penji penjo mang’eny ewi lokoni. | | |
| **No.** | **Question** | **Coding** |
| RT1: Most Recent Exchange | | |
| RT1 Q01 | Would you like to tell me about the most recent time you exchanged sex in the past 6 months?  Be diher mar nyisa ewi bedo e achiel e ringruok manitimo mogik mondo iyud pesa, mwandu kata mich kuom dweche auchiel ma okalo? | O Yes  O No (skip to RT2 Q01) |
| RT1 Q02 | When did this sex exchange for money, goods, or gifts occur?  Ne en kar ang’o mogik mane iloko bedo e achiel e ringruok ne pesa, mwandu kata mich? | O I Know the Date  O Don’t Know (skip to RT1 Q04)  O Refused to Answer (skip to RT1 Q04) |
| RT1 Q03 | What was the date?  Ne en tarik mane? | ___ ___/___ ___ ___/ ___ ___ ___ ___  D D M M M Y Y Y Y |
| RT1 Q04 | Where did you exchange sex with this person?  En kanye mane mane iwilo bedo e achiel gi gimoro kod ng’atni? | O Street, car, or outside  O Bar or nightclub  O Hotel room paid by sex worker  O Hotel room paid by partner  O Brothel  O Woman’s home  O Man’s home  O Other (specify): __________________________  O Don't Know  O Refused to Answer |
| RT1 Q05 | Please tell me which of the following activities you did during this encounter:  E kindego, ne itimo mane kuom gik ma okwan kaegi?  *Interviewer: Read the list of responses aloud to the participant and choose all that apply. Explain any of the choices that the participant does not understand.* | O Kissing  O Danced or stripped for partner  O Massage  O Performed oral sex **with** a condom  O Performed oral sex **without** a condom  O Vaginal sex **with** a condom  O Vaginal sex **without** a condom  O Anal sex **with** a condom  O Anal sex **without** a condom  O Received oral sex  O Talking/company  O Other (specify) |
| RT1 Q06 | Was this the first time you have exchanged sex with this person?  Bende ma e chieng mokwongo mane ibedo e achiel e ringruok kod ngatni? | O Yes  O No  O Don’t Know O Refused to Answer |
| RT1 Q07 | Approximately how old was this person? If not sure. please estimate.  Ng’ani ne en jahigni adi kama? Ka ok in gi adier to wach awacha ma iparo ni chiegni kode | ___ ___ years  O Don’t know  O Refused to answer |
| RT1 Q08 | Does this person live in this County, or was he visiting?  Ng’atni odak ei County ni koso ne timo limbe? | O Lives in this County  O Just visiting  O Don’t know  O Refused to answer |
| RT1 Q09 | Did this person take alcohol or drugs around the time you had sex?  Bende ng’atni ne otiyo kod kong’o ka pok ubedo e achiel e ringruok, seche ma ubet e achiel e ringruok? | O Yes  O No  O Don’t know  O Refused to answer |
| RT1 Q10 | Did YOU take alcohol or drugs around the time you had sex with this person?  Bende ne itiyo kod yedhe ma mero ji ka pok ubet e achiel e ringruok, seche ma ubet e achiel e ringruok? | O Yes  O No  O Don’t know  O Refused to answer |
| RT1 Q11 | What was the **total value** of money, goods and gifts that the person gave you for this encounter?  Duto, pesa, mwandu kod mich mane jaherani omiyi chieng’no ne gin madirom pesa adi? | KES_________  O Don't Know  O Refused to Answer |
| RT1 Q12 | Did you know the HIV status of this person around the time you had sex?  Bende ne ing’eyo chal mar nga’tni mar kute mag Ayaki e seche mane ibet kode e achiel e ringruok? | O Yes  O No  O Refused to answer |
| NEXT SET OF QUESTIONS ARE FOR PARTICIPANTS IN **INTERVENTION** GROUP | | |
| RT1 Q13 | Did you offer an HIV self-test to this person?  Bende ne ichiwo gir pim mar kute mag Ayaki ma nga’to pimorego kende owuon ne nga’tni? | O Yes  O No 🡪 skip to RT1 Q28  O Don’t know 🡪 skip to RT1 Q28  O Refused to answer 🡪 skip to RT1 Q28 |
| RT1 Q14 | Did he accept the HIV self-test?  Bende ne orwako gir pim mar kute mag Ayaki ma nga’to pimorego kende owuon? | O Yes  O No 🡪 skip to RT1 Q28  O Don’t know 🡪 skip to RT1 Q28  O Refused to answer 🡪 skip to RT1 Q28 |
| RT1 Q15 | Did he use the HIV self-test?  Bende ne otiyo kod gir pim mar kute mag Ayaki ma nga’to pimorego kende owuon? | O Yes  O No 🡪 Skip to RT1 Q28  O Don’t know 🡪 skip to RT1 Q28  O Refused to answer 🡪 skip to RT1 Q28 |
| RT1 Q16 | When did he use the self-test?  En karang’o mane otiyo kod gir pim mar kute mag Ayaki ma nga’to pimorego kende owuon? | O Less than 1 week ago  O Less than 1 month ago  O Less than 3 months ago  O Less than 6 months ago  O Don’t know  O Refused to answer |
| RT1 Q17 | Were you present when he used the self-test?  Bende ne intiere kode seche mane otiyo kod gir pim mar kute mag Ayaki ma nga’to pimorego kende owuon? | O I was present when he used the self-test  O I was not present, but he told me that he used the self-test kit 🡪 skip to RT1 Q19  O Don’t know  O Refused to answer |
| RT1 Q18 | At the **same** time that your partner used the self-test, did you **also** use a self-test?  E seche mane jaherani otiyo kod gir pim mar kute mag Ayaki ma nga’to pimorego kende owuon, un bende ne itiyo kod gir pim mar kute mag Ayaki ma nga’to pimorego kende owuon? | O Yes, we tested together at the same time  O Yes, we both tested but not together  O No  O Other (specify)  O Don’t know  O Refused to answer |
| RT1 Q19 | What was the result of his HIV test?  Duoko mar gir pim mar kute mag Ayaki ma nga’to pimorego kende owuon ne en mane? | O Positive  O Negative  O Invalid / Indeterminate  O Don’t Know  O Refused |
| RT1 Q20 | Did he go to a clinic or health facility to confirm the result that was obtained from the self-test (confirmatory testing)?  Bende nga’tni ne odhi e klinik kata kar thieth mondo onon duoko mane oyudi e gir pim mar kute mag Ayaki ma nga’to pimorego kende owuon? | O Yes  O No  O Don’t know  O Refused to answer |
| RT1 Q21 | Did he go to a clinic or health facility for HIV care and treatment?  Bende nga’tni ne odhi e klinik kata kar thieth mondo ochak yudo kony mar joma ni kod kute mag Ayaki? | O Yes  O No  O Don’t know  O Refused to answer |
| NEXT SET OF QUESTIONS ARE FOR **CONTROL** GROUP ONLY | | |
| RT1 Q22 | Did you offer a VCT referral card to this person?  Bende ne imiye kadi mar ote mar VCT? | O Yes  O No 🡪 Skip to RT1 Q28  O Don’t know 🡪 Skip to RT1 Q28  O Refused to answer 🡪 Skip to RT1 Q28 |
| RT1 Q23 | Did he accept the VCT referral card?  Bende ne orwako kadi mar ote? | O Yes  O No 🡪 Skip to RT1 Q28  O Don’t know 🡪 Skip to RT1 Q28  O Refused to answer 🡪 Skip to RT1 Q28 |
| RT1 Q24 | Do you know whether he went for an HIV test at a VCT clinic?  Bende ne inge’yo ka ne odhi yudo pim mar kute mag Ayaki kama itimoe pim mar kute mag Ayaki? | O Yes  O No 🡪 Skip to RT1 Q28  O Don’t know 🡪 Skip to RT1 Q28  O Refused to answer 🡪 Skip to RT1 Q28 |
| RT1 Q25 | At the **same** time that your partner got tested for HIV, did you also test?  Eseche mane jaherani opimore kute mag Ayaki, in bende ne ipimori kute mag Ayaki? | O Yes, we tested together at the same time  O Yes, we both tested but not together  O No  O Other (specify)  O Don’t know  O Refused to answer |
| RT1 Q26 | What was the result of his HIV test?  Duokone mar pimo mar kute mag Ayaki ne en mane? | O Positive  O Negative 🡪 skip to RT1 Q28  O Invalid / Indeterminate  O Don’t Know 🡪 skip to RT1 Q28  O Refused 🡪 skip to RT1 Q28 |
| RT1 Q27 | Did he go to a clinic or health facility for HIV care and treatment?  Bende ne odhi e klinik kata kar thieth mondo oyud kony mar joma oyud kod kute? | O Yes  O No  O Don’t know  O Refused to answer |
| Interviewer READ: I will now ask you some questions about things this same person may have done to you during this encounter. These questions are about violence, which may make you feel uncomfortable or distressed. Please remember that your answers are completely confidential and no one will be able to associate this information with you or your sexual partners. You do not have to answer any question that makes you uncomfortable. Please take your time and if you are unclear about any question, just ask me. Are you ready to continue?  Koro abiro penji gik ma ng’ato achielni ne otimoni e kinde man ubedo e achielni. Penjo gi gin mawuoyo kuom tulo, ma nyalo miyo ok iwinj maber kata nyalo miyo ibedi gi parruok. Kiyie to par kendo ni weche ma iwacho ikano maling’ling’kendo onge ng’atmoro amora manyalo tudo weche ma iwacho kodi kata kod joherani ma ibedogago e achiel. Ok ochuno ni nyaka iduoki penjo moro amora ma miyo itho dich kata ibedo gi parruok. Kiyie kaw thuolo mari kendo ka ok iwinjo penjo moro amora maber, to penja apenja. Bend iikori mar dhi nyime? | | |
| RT1 Q28 | Did this partner threaten you with physical assault?  Bende jaherani ne obuogi ni onyalo hinyo dendi? | O Yes  O No  O Don’t know  O Refused to answer |
| RT1 Q29 | Did this partner hit, kick, strangle or otherwise physically assault you?  Bende jaherani ne ogoyi, ogweyi, odeyi kata ohinyo dendi e yoo moro amora? | O Yes  O No  O Don’t know  O Refused to answer |
| RT1 Q30 | Did this partner force or coerce you to participate in any sex act against your will?  Bende jaherani ne ochuni mondo ibed e achiel e ringruok e yoo moro amora ma ok ne idwar? | O Yes, to have sex with a condom  O Yes, to have sex without a condom  O Yes, to have anal sex  O Yes, Other____________________________  O No  O Don’t know  O Refused to answer |
| Interviewer: Now, I will be asking you the same questions as I just did, for your SECOND MOST RECENT encounter where you exchanged sex for something. This could be with a different partner as the one we just discussed, or the same.  Koro adhi penji penjo machalre mana kod amaa penji sani ka, e wi bedo ni a e achiel ma moror no nyocha oluwo to be ne omiyi gimoro bang’ bedo e achiel. Maendi nyalo bedo jaherani machielo mopogore kod morocha to bende onyalo bedo mawaa wuoye sani cha | | |
| RT2 Q01 | Would you like to tell me about the most recent time you exchanged sex in the past 6 months?  Be diher mar nyisa ewi bedo e achiel e ringruok manitimo mogik mondo iyud pesa, mwandu kata mich kuom dweche auchiel ma okalo? | O Yes  O No (skip to RT3 Q01) |
| RT2 Q02 | When did this sex exchange for money, goods, or gifts occur?  Ne en kar ang’o mogik mane iloko bedo e achiel e ringruok ne pesa, mwandu kata mich? | O I Know the Date  O Don’t Know (skip to RT2 Q04)  O Refused to Answer (skip to RT2 Q04) |
| RT2 Q03 | What was the date?  Ne en tarik mane? | ___ ___/___ ___ ___/ ___ ___ ___ ___  D D M M M Y Y Y Y |
| RT2 Q04 | Where did you exchange sex with this person?  En kanye mane mane iwilo bedo e achiel gi gimoro kod ng’atni? | O Street, car, or outside  O Bar or nightclub  O Hotel room paid by sex worker  O Hotel room paid by partner  O Brothel  O Woman’s home  O Man’s home  O Other (specify): __________________________  O Don't Know  O Refused to Answer |
| RT2 Q05 | Please tell me which of the following activities you did during this encounter:  E kindego, ne itimo mane kuom gik ma okwan kaegi?  *Interviewer: Read the list of responses aloud to the participant and choose all that apply. Explain any of the choices that the participant does not understand.* | O Kissing  O Danced or stripped for partner  O Massage  O Performed oral sex **with** a condom  O Performed oral sex **without** a condom  O Vaginal sex **with** a condom  O Vaginal sex **without** a condom  O Anal sex **with** a condom  O Anal sex **without** a condom  O Received oral sex  O Talking/company  O Other (specify): ________________________  O Don't Know  O Refused to Answer |
| RT2 Q06 | Was this the first time you have exchanged sex with this person?  Bende ma e chieng mokwongo mane ibedo e achiel e ringruok kod ngatni? | O Yes  O No  O Don’t Know O Refused to Answer |
| RT2 Q07 | Approximately how old was this person? If not sure. please estimate.  Ng’ani ne en jahigni adi kama? Ka ok in gi adier to wach awacha ma iparo ni chiegni kode | ___ ___ years  O Don’t know  O Refused to answer |
| RT2 Q08 | Does this person live in this County, or was he visiting?  Ng’atni odak ei County ni koso ne timo limbe? | O Lives in this County  O Just visiting  O Don’t know  O Refused to answer |
| RT2 Q09 | Did this person take alcohol or drugs around the time you had sex?  Bende ng’atni ne otiyo kod kong’o ka pok ubedo e achiel e ringruok, seche ma ubet e achiel e ringruok kata bang’ ka usebedo e achiel e ringruok? | O Yes  O No  O Don’t know  O Refused to answer |
| RT2 Q10 | Did YOU take alcohol or drugs around the time you had sex with this person?  Bende ne itiyo kod yedhe ma mero ji ka pok ubet e achiel e ringruok, seche ma ubet e achiel e ringruok kata bang’ ka usebedo e achiel e ringruok? | O Yes  O No  O Don’t know  O Refused to answer |
| RT2 Q11 | What was the **total value** of money, goods and gifts that the person gave you for this encounter?  Duto, pesa, mwandu kod mich mane jaherani omiyi chieng’no ne gin madirom pesa adi? | KES_________  O Don't Know  O Refused to Answer |
| RT2 Q12 | Did you know the HIV status of this person around the time you had sex?  Bende ne ing’eyo chal mar nga’tni mar kute mag Ayaki e seche mane ibet kode e achiel e ringruok? | O Yes  O No  O Refused to answer |
| NEXT SET OF QUESTIONS ARE FOR PARTICIPANTS IN **INTERVENTION** GROUP | | |
| RT2 Q13 | Did you offer an HIV self-test to this person?  Bende ne ichiwo gir pim mar kute mag Ayaki ma nga’to pimorego kende owuon ne nga’tni? | O Yes  O No 🡪 skip to RT2 Q28  O Don’t know 🡪 skip to RT2 Q28  O Refused to answer 🡪 skip to RT2 Q28 |
| RT2 Q14 | Did he accept the HIV self-test?  Bende ne orwako gir pim mar kute mag Ayaki ma nga’to pimorego kende owuon? | O Yes  O No 🡪 skip to RT2 Q28  O Don’t know 🡪 skip to RT2 Q28  O Refused to answer 🡪 skip to RT2 Q28 |
| RT2 Q15 | Did he use the HIV self-test?  Bende ne otiyo kod gir pim mar kute mag Ayaki ma nga’to pimorego kende owuon? | O Yes  O No 🡪 Skip to RT2 Q28  O Don’t know 🡪 skip to RT2 Q28  O Refused to answer 🡪 skip to RT2 Q28 |
| RT2 Q16 | When did he use the self-test?  En karang’o mane otiyo kod gir pim mar kute mag Ayaki ma nga’to pimorego kende owuon? | O Less than 1 week ago  O Less than 1 month ago  O Less than 3 months ago  O Less than 6 months ago  O Don’t know  O Refused to answer |
| RT2 Q17 | Were you present when he used the self-test?  Bende ne intiere kode seche mane otiyo kod gir pim mar kute mag Ayaki ma nga’to pimorego kende owuon? | O I was present when he used the self-test  O I was not present, but he told me that he used the self-test kit 🡪 skip to RT2 Q19  O Don’t know  O Refused to answer |
| RT2 Q18 | At the **same** time that your partner used the self-test, did you **also** use a self-test?  E seche mane jaherani otiyo kod gir pim mar kute mag Ayaki ma nga’to pimorego kende owuon, un bende ne itiyo kod gir pim mar kute mag Ayaki ma nga’to pimorego kende owuon? | O Yes, we tested together at the same time  O Yes, we both tested but not together  O No  O Other (specify)  O Don’t know  O Refused to answer |
| RT2 Q19 | What was the result of his HIV test?  Duoko mar gir pim mar kute mag Ayaki ma nga’to pimorego kende owuon ne en mane? | O Positive  O Negative  O Invalid / Indeterminate  O Don’t Know  O Refused |
| RT2 Q20 | Did he go to a clinic or health facility to confirm the result that was obtained from the self-test (confirmatory testing)?  Bende nga’tni ne odhi e klinik kata kar thieth mondo onon duoko mane oyudi e gir pim mar kute mag Ayaki ma nga’to pimorego kende owuon? | O Yes  O No  O Don’t know  O Refused to answer |
| RT2 Q21 | Did he go to a clinic or health facility for HIV care and treatment?  Bende nga’tni ne odhi e klinik kata kar thieth mondo ochak yudo kony mar joma ni kod kute mag Ayaki? | O Yes  O No  O Don’t know  O Refused to answer |
| NEXT SET OF QUESTIONS ARE FOR **CONTROL** GROUP ONLY | | |
| RT2 Q22 | Did you offer a VCT referral card to this person?  Bende ne imiye kadi mar ote mar VCT? | O Yes  O No 🡪 Skip to RT2 Q28  O Don’t know 🡪 Skip to RT2 Q28  O Refused to answer 🡪 Skip to RT2 Q28 |
| RT2 Q23 | Did he accept the VCT referral card?  Bende ne orwako kadi mar ote? | O Yes  O No 🡪 Skip to RT2 Q28  O Don’t know 🡪 Skip to RT2 Q28  O Refused to answer 🡪 Skip to RT2 Q28 |
| RT2 Q24 | Do you know whether he went for an HIV test at a VCT clinic?  Bende ne inge’yo ka ne odhi yudo pim mar kute mag Ayaki kama itimoe pim mar kute mag Ayaki? | O Yes  O No 🡪 Skip to RT2 Q28  O Don’t know 🡪 Skip to RT2 Q28  O Refused to answer 🡪 Skip to RT2 Q28 |
| RT2 Q25 | At the **same** time that your partner got tested for HIV, did you also test?  Eseche mane jaherani opimore kute mag Ayaki, in bende ne ipimori kute mag Ayaki? | O Yes, we tested together at the same time  O Yes, we both tested but not together  O No  O Other (specify)  O Don’t know  O Refused to answer |
| RT2 Q26 | What was the result of his HIV test?  Duokone mar pimo mar kute mag Ayaki ne en mane? | O Positive  O Negative 🡪 skip to RT2 Q28  O Invalid / Indeterminate  O Don’t Know 🡪 skip to RT2 Q28  O Refused 🡪 skip to RT2 Q28 |
| RT2 Q27 | Did he go to a clinic or health facility for HIV care and treatment?  Bende ne odhi e klinik kata kar thieth mondo oyud kony mar joma oyud kod kute? | O Yes  O No  O Don’t know  O Refused to answer |
| Interviewer READ: I will now ask you some questions about things this same person may have done to you during this encounter. These questions are about violence, which may make you feel uncomfortable or distressed. Please remember that your answers are completely confidential and no one will be able to associate this information with you or your sexual partners. You do not have to answer any question that makes you uncomfortable. Please take your time and if you are unclear about any question, just ask me. Are you ready to continue?  Koro abiro penji gik ma ng’ato achielni ne otimoni e kinde man ubedo e achielni. Penjo gi gin mawuoyo kuom tulo, ma nyalo miyo ok iwinj maber kata nyalo miyo ibedi gi parruok. Kiyie to par kendo ni weche ma iwacho ikano maling’ling’kendo onge ng’atmoro amora manyalo tudo weche ma iwacho kodi kata kod joherani ma ibedogago e achiel. Ok ochuno ni nyaka iduoki penjo moro amora ma miyo itho dich kata ibedo gi parruok. Kiyie kaw thuolo mari kendo ka ok iwinjo penjo moro amora maber, to penja apenja. Bend iikori mar dhi nyime? | | |
| RT2 Q28 | Did this partner threaten you with physical assault?  Bende jaherani ne obuogi ni onyalo hinyo dendi? | O Yes  O No  O Don’t know  O Refused to answer |
| RT2 Q29 | Did this partner hit, kick, strangle or otherwise physically assault you?  Bende jaherani ne ogoyi, ogweyi, odeyi kata ohinyo dendi e yoo moro amora? | O Yes  O No  O Don’t know  O Refused to answer |
| RT2 Q30 | Did this partner force or coerce you to participate in any sex act against your will?  Bende jaherani ne ochuni mondo ibed e achiel e ringruok e yoo moro amora ma ok ne idwar? | O Yes, to have sex with a condom  O Yes, to have sex without a condom  O Yes, to have anal sex  O Yes, Other____________________________  O No  O Don’t know  O Refused to answer |
| Interviewer: For the last time, I will be asking you the same questions as I just did about your third most recent encounter where you exchanged sex for something. This could be with a different partner as the one we just discussed, or the same.  “Koro abiro penji gik ma ng’ato achielni ne otimoni e kinde man ubedo e achielni. Penjo gi gin mawuoyo kuom tulo, ma nyalo miyo ok iwinj maber kata nyalo miyo ibedi gi parruok. Kiyie to par kendo ni weche ma iwacho ikano maling’ling’kendo onge ng’atmoro amora manyalo tudo weche ma iwacho kodi kata kod joherani ma ibedogago e achiel. Ok ochuno ni nyaka iduoki penjo moro amora ma miyo itho dich kata ibedo gi parruok . Kiyie kaw thuolo mari kendo ka ok iwinjo penjo moro amora maber, to penja apenja. Bend iikori mar dhi nyime?” | | |
| RT3 Q01 | Would you like to tell me about the most recent time you exchanged sex in the past 6 months?  Be diher mar nyisa ewi bedo e achiel e ringruok manitimo mogik mondo iyud pesa, mwandu kata mich kuom dweche auchiel ma okalo? | O Yes  O No (skip to HIV01) |
| RT3 Q02 | When did this sex exchange for money, goods, or gifts occur?  Ne en kar ang’o mogik mane iloko bedo e achiel e ringruok ne pesa, mwandu kata mich? | O I Know the Date  O Don’t Know (skip to RT3 Q04)  O Refused to Answer (skip to RT3 Q04) |
| RT3 Q03 | What was the date?  Ne en tarik mane? | ___ ___/___ ___ ___/ ___ ___ ___ ___  D D M M M Y Y Y Y |
| RT3 Q04 | Where did you exchange sex with this person?  En kanye mane mane iwilo bedo e achiel gi gimoro kod ng’atni? | O Street, car, or outside  O Bar or nightclub  O Hotel room paid by sex worker  O Hotel room paid by partner  O Brothel  O Woman’s home  O Man’s home  O Other (specify): __________________________  O Don't Know  O Refused to Answer |
| RT3 Q05 | Please tell me which of the following activities you did during this encounter:  E kindego, ne itimo mane kuom gik ma okwan kaegi?  *Interviewer: Read the list of responses aloud to the participant and choose all that apply. Explain any of the choices that the participant does not understand.* | O Kissing  O Danced or stripped for partner  O Massage  O Performed oral sex **with** a condom  O Performed oral sex **without** a condom  O Vaginal sex **with** a condom  O Vaginal sex **without** a condom  O Anal sex **with** a condom  O Anal sex **without** a condom  O Received oral sex  O Talking/company  O Other (specify) |
| RT3 Q06 | Was this the first time you have exchanged sex with this person?  Bende ma e chieng mokwongo mane ibedo e achiel e ringruok kod ngatni? | O Yes  O No  O Don’t Know O Refused to Answer |
| RT3 Q07 | Approximately how old was this person? If not sure. please estimate.  Ng’ani ne en jahigni adi kama? Ka ok in gi adier to wach awacha ma iparo ni chiegni kode | ___ ___ years  O Don’t know  O Refused to answer |
| RT3 Q08 | Does this person live in this County, or was he visiting?  Ng’atni odak ei County ni koso ne timo limbe? | O Lives in this County  O Just visiting  O Don’t know  O Refused to answer |
| RT3 Q09 | Did this person take alcohol or drugs around the time you had sex?  Bende ng’atni ne otiyo kod kong’o ka pok ubedo e achiel e ringruok, seche ma ubet e achiel e ringruok kata bang’ ka usebedo e achiel e ringruok? | O Yes  O No  O Don’t know  O Refused to answer |
| RT3 Q10 | Did YOU take alcohol or drugs around the time you had sex with this person?  Bende ne itiyo kod yedhe ma mero ji ka pok ubet e achiel e ringruok, seche ma ubet e achiel e ringruok kata bang’ ka usebedo e achiel e ringruok? | O Yes  O No  O Don’t know  O Refused to answer |
| RT3 Q11 | What was the **total value** of money, goods and gifts that the person gave you for this encounter?  Duto, pesa, mwandu kod mich mane jaherani omiyi chieng’no ne gin madirom pesa adi? | KES_________  O Don't Know  O Refused to Answer |
| RT3 Q12 | Did you know the HIV status of this person around the time you had sex?  Bende ne ing’eyo chal mar nga’tni mar kute mag Ayaki e seche mane ibet kode e achiel e ringruok? | O Yes  O No  O Refused to answer |
| NEXT SET OF QUESTIONS ARE FOR PARTICIPANTS IN **INTERVENTION** GROUP | | |
| RT3 Q13 | Did you offer an HIV self-test to this person?  Bende ne ichiwo gir pim mar kute mag Ayaki ma nga’to pimorego kende owuon ne nga’tni? | O Yes  O No 🡪 skip to RT3 Q28  O Don’t know 🡪 skip to RT3 Q28  O Refused to answer 🡪 skip to RT3 Q28 |
| RT3 Q14 | Did he accept the HIV self-test?  Bende ne orwako gir pim mar kute mag Ayaki ma nga’to pimorego kende owuon? | O Yes  O No 🡪 skip to RT3 Q28  O Don’t know 🡪 skip to RT3 Q28  O Refused to answer 🡪 skip to RT3 Q28 |
| RT3 Q15 | Did he use the HIV self-test?  Bende ne otiyo kod gir pim mar kute mag Ayaki ma nga’to pimorego kende owuon? | O Yes  O No 🡪 Skip to RT3 Q28  O Don’t know 🡪 skip to RT3 Q28  O Refused to answer 🡪 skip to RT3 Q28 |
| RT3 Q16 | When did he use the self-test?  En karang’o mane otiyo kod gir pim mar kute mag Ayaki ma nga’to pimorego kende owuon? | O Less than 1 week ago  O Less than 1 month ago  O Less than 3 months ago  O Less than 6 months ago  O Don’t know  O Refused to answer |
| RT3 Q17 | Were you present when he used the self-test?  Bende ne intiere kode seche mane otiyo kod gir pim mar kute mag Ayaki ma nga’to pimorego kende owuon? | O I was present when he used the self-test  O I was not present, but he told me that he used the self-test kit 🡪 skip to RT3 Q19  O Don’t know  O Refused to answer |
| RT3 Q18 | At the **same** time that your partner used the self-test, did you **also** use a self-test?  E seche mane jaherani otiyo kod gir pim mar kute mag Ayaki ma nga’to pimorego kende owuon, un bende ne itiyo kod gir pim mar kute mag Ayaki ma nga’to pimorego kende owuon? | O Yes, we tested together at the same time  O Yes, we both tested but not together  O No  O Other (specify)  O Don’t know  O Refused to answer |
| RT3 Q19 | What was the result of his HIV test?  Duoko mar gir pim mar kute mag Ayaki ma nga’to pimorego kende owuon ne en mane? | O Positive  O Negative  O Invalid / Indeterminate  O Don’t Know  O Refused |
| RT3 Q20 | Did he go to a clinic or health facility to confirm the result that was obtained from the self-test (confirmatory testing)?  Bende nga’tni ne odhi e klinik kata kar thieth mondo onon duoko mane oyudi e gir pim mar kute mag Ayaki ma nga’to pimorego kende owuon? | O Yes  O No  O Don’t know  O Refused to answer |
| RT3 Q21 | Did he go to a clinic or health facility for HIV care and treatment?  Bende nga’tni ne odhi e klinik kata kar thieth mondo ochak yudo kony mar joma ni kod kute mag Ayaki? | O Yes  O No  O Don’t know  O Refused to answer |
| NEXT SET OF QUESTIONS ARE FOR **CONTROL** GROUP ONLY | | |
| RT3 Q22 | Did you offer a VCT referral card to this person?  Bende ne imiye kadi mar ote mar VCT? | O Yes  O No 🡪 Skip to RT3 Q28  O Don’t know 🡪 Skip to RT3 Q28  O Refused to answer 🡪 Skip to RT3 Q28 |
| RT3 Q23 | Did he accept the VCT referral card?  Bende ne orwako kadi mar ote? | O Yes  O No 🡪 Skip to RT3 Q28  O Don’t know 🡪 Skip to RT3 Q28  O Refused to answer 🡪 Skip to RT3 Q28 |
| RT3 Q24 | Do you know whether he went for an HIV test at a VCT clinic?  Bende ne inge’yo ka ne odhi yudo pim mar kute mag Ayaki kama itimoe pim mar kute mag Ayaki? | O Yes  O No 🡪 Skip to RT3 Q28  O Don’t know 🡪 Skip to RT3 Q28  O Refused to answer 🡪 Skip to RT3 Q28 |
| RT3 Q25 | At the **same** time that your partner got tested for HIV, did you also test?  Eseche mane jaherani opimore kute mag Ayaki, in bende ne ipimori kute mag Ayaki? | O Yes, we tested together at the same time  O Yes, we both tested but not together  O No  O Other (specify)  O Don’t know  O Refused to answer |
| RT3 Q26 | What was the result of his HIV test?  Duokone mar pimo mar kute mag Ayaki ne en mane? | O Positive  O Negative 🡪 skip to RT3 Q28  O Invalid / Indeterminate  O Don’t Know 🡪 skip to RT3 Q28  O Refused 🡪 skip to RT3 Q28 |
| RT3 Q27 | Did he go to a clinic or health facility for HIV care and treatment?  Bende ne odhi e klinik kata kar thieth mondo oyud kony mar joma oyud kod kute? | O Yes  O No  O Don’t know  O Refused to answer |
| Interviewer READ: I will now ask you some questions about things this same person may have done to you during this encounter. These questions are about violence, which may make you feel uncomfortable or distressed. Please remember that your answers are completely confidential and no one will be able to associate this information with you or your sexual partners. You do not have to answer any question that makes you uncomfortable. Please take your time and if you are unclear about any question, just ask me. Are you ready to continue?  Koro abiro penji gik ma ng’ato achielni ne otimoni e kinde man ubedo e achielni. Penjo gi gin mawuoyo kuom tulo, ma nyalo miyo ok iwinj maber kata nyalo miyo ibedi gi parruok. Kiyie to par kendo ni weche ma iwacho ikano maling’ling’kendo onge ng’atmoro amora manyalo tudo weche ma iwacho kodi kata kod joherani ma ibedogago e achiel. Ok ochuno ni nyaka iduoki penjo moro amora ma miyo itho dich kata ibedo gi parruok. Kiyie kaw thuolo mari kendo ka ok iwinjo penjo moro amora maber, to penja apenja. Bend iikori mar dhi nyime? | | |
| RT3 Q28 | Did this partner threaten you with physical assault?  Bende jaherani ne obuogi ni onyalo hinyo dendi? | O Yes  O No  O Don’t know  O Refused to answer |
| RT3 Q29 | Did this partner hit, kick, strangle or otherwise physically assault you?  Bende jaherani ne ogoyi, ogweyi, odeyi kata ohinyo dendi e yoo moro amora? | O Yes  O No  O Don’t know  O Refused to answer |
| RT3 Q30 | Did this partner force or coerce you to participate in any sex act against your will?  Bende jaherani ne ochuni mondo ibed e achiel e ringruok e yoo moro amora ma ok ne idwar? | O Yes, to have sex with a condom  O Yes, to have sex without a condom  O Yes, to have anal sex  O Yes, Other____________________________  O No  O Don’t know  O Refused to answer |

| **Section E: HIV and HIV Testing** | | |
| --- | --- | --- |
| **Section E1: General HIV Knowledge (HIV)**  Interviewer: I would now like to ask you some questions about what you already know and feel about HIV.  Koro daher penji penjo ewi gik ma ing’eyo kata kaka iparo ewi kute mag Ayaki. | | |
| HIV01 | Are you currently taking any HIV medication in order to **prevent** acquiring HIV (PrEP)? This is usually a pill taken daily.  E kindegi, bende itiyogi yedhe mag gayo kute mag ayaki (PrEP)? Ma en yath ma imuonyo pile pile.  *Interviewer: Ensure participant understands the principles of PrEP before proceeding. This is specifically asking about PrEP medication, not traditional medicines taken to prevent HIV.* | O Yes  O No  O Don’t Know  O Refused to Answer |
| HIV02 | What do you think your chances are of acquiring HIV in the future?  Iparo ni thuoloni mag gamo kute mag Ayaki; onge, nipiny, nidiere, koso nimalo? | O None (Ask HIV03, not HIV04)  O Low (Ask HIV03, not HIV04)  O Moderate (skip to HIV04)  O High (skip to HIV04)  O Don’t Know (skip to HIV05)  O Refused to Answer (skip to HIV05) |
| HIV03 | *Only ask if HIV02 = None or Low*  Why do you think you have a low chance or no chance of acquiring HIV in the future?  Ang’o ma omiyo iparo ni in kod thuolo ma piny kata ionge thuolo mar gamo kute mag ayaki?  *Choose all that apply based on what participant mentioned.* | O Is Not Having Sex  O Uses Condoms  O Has Only One Partner  O Limits the Number of Partners  O Partner Has No Other Partners  O Knows Partner(s)’ HIV Status is Negative  O Trusts partner  O My Current Status is Negative  O Other _________________________________  O Don’t Know  O Refused to Answer |
| HIV04 | *Only ask if HIV02 = Moderate or High*  Why do you think you have a moderate or high chance of acquiring HIV in the future?  Ang’o ma omiyo iparo ni in kod thuolo man e diere kata ma malo mar gamo kute mag Ayaki?    *Choose all that apply based on what the participant mentioned.* | O Does Not Use Condoms Regularly or at all  O Woman Has More Than One Partner  O Has Transactional Sex  O Does Not Trust Partner  O Partner is HIV positive  O She or Partner Refuses to be Tested  O Uses Injection Drugs/ Needles  O Primary Partner has more than one partner  O Non-primary partner(s) have more than one partner  O Other _________________________________  O Don’t Know  O Refused to Answer |
| HIV05 | How often do you believe you should test for HIV? Feel free to give more than one answer.  Iparo ni onego bedi ni ipimo kute mag Ayaki bang’ thuolo marom nadi? Bedi thoulo mar chiwo duoko mokalo achiel.  *Interviewer - Choose all that apply. Responses can be frequency or situational.* | O Every week  O Every month  O Every 3 months  O Every 6 months  O Every year  O When I have sex without a condom  O When I have a new partner  O When my primary partner informs me or I know that he was unfaithful  O During pregnancy  O Other: ________________________________  O If I have been tested once, I do not need to be tested again  O Don’t Know  O Refused to Answer |
| **Section E2: Sexual Testing History (TST)**  Interviewer: I would now like to ask you some questions about testing for HIV and other sexually transmitted infections (STI) such as syphilis, gonorrhea, chlamydia, *Trichomonas vaginalis*, or bacterial vaginosis.  Koro adwaro penji penjo moko kuom pimruok mar kute mag ayaki to kod touché mamoko mag nyeye machalo kaka syphilis, gonorrhea, chlamydia, *Trichomonas vaginalis*, or bacterial vaginosis. | | |
| TST01 | Before the test today, when did you last get tested for HIV?  Ka waweyo pim makawuononi, bende osega pimi kute mag ayaki?  *Interviewer – remind participant that using a self-test is also an example of getting tested.* | O Less than 1 week ago  O Between 1 week and 1 month ago  O Between 1 and 3 months ago  O Between 3 and 6 months ago  O Don’t Know  O Refused to Answer |
| TST02 | In the past 6 months, i.e. since your first appointment with Jikinge, have you been diagnosed with a sexually transmitted infection (STI)?  Kuom dweche 6 ma okalo, i.e chakra ibed jachiwre e Jikinge, bende oseyudi kod tuoche mag nyeye? | O Yes  O No (skip to GBV01)  O Don’t Know (skip to GBV01)  O Refused to answer (skip to GBV01) |
| TST03 | Which STI(s) were you diagnosed with?  En tuo mane mar nyach ma ne oyudigo?  *Choose all that apply.*  *Interviewer: This is self-reported by the participant. If she does not remember the diagnosis, use “Don’t Know”. Do not try to diagnose her using symptoms she describes.* | O Trichomoniasis (Trich)  O Syphilis  O Gonorrhea  O Chlamydia  O Herpes  O Human papillomavirus (HPV)  O Genital warts  O Mycoplasma genitalium  O Bacterial vaginosis (BV)  O Other _______________________________  O Don’t Know  O Refused to answer |
| TST04 | Did you consult a doctor, pharmacist or other qualified healthcare provider in order to obtain treatment for this STI?  Bende ne idhi ir daktar, nga’ma uso yedhe, kata moro amora kuom jochiw kony mag ngima mondo iyud thieth mag tuoche mag nyeye?  *Interviewer: This includes traditional healers.* | O Yes  O No  O Don’t Know  O Refused to answer |

| **Section F: Gender-Based Violence (GBV)** | | |
| --- | --- | --- |
| *Interviewer*: The next questions are about things that happen within some relationships, and that your primary partner, or any other partners may have done to you. These questions are about violence, which may make you feel uncomfortable or distressed. You do not have to answer any question that makes you feel uncomfortable, and your responses are confidential. If you have any questions at any time, please ask me. Are you ready to continue?  Penjo maluwo gin kuom gik matimre e tudruoge moko, kod gima jaherani mahie kata joherani mamoko nyalo bedo ni osetimoni. Penjogi gin ewi tulo, manyalo miyo itho dich kata chunyi chandre. Ok ochuno ni nyaka iduok penjo moro amora mamiyo itho dich, kendo duokogi gin e yor maling’ ling’. Ka in kod penjo moro amora e saa moro amora, bed thuolo mar penja. Bende iikori mondo wadhi nyime? | | |
| **Section F1. PRIMARY PARTNERS** | | |
| GBV01:  Since you joined the Jikinge study( i.e. since [ENROLLMENT DATE]), has your **PRIMARY** sexual partner done any of the following to you?  Chakre ibed jachiwre e nonro mar JIKINGE (i.e since [ENROLLMENT DATE]), bende jaherani mahie osetimoni moko kuom magi?  1 No 🡪 skip to next item  2 Yes 🡪 ask GBV02 and GBV03  99 Refused to answer 🡪 skip to next item  *Interviewer: Read each option aloud one at a time and choose all that apply.* | GBV02:  In the past 6 months, has your **PRIMARY** partner done this often or only sometimes?  kinde mar dueche 6 mokalo, bende jaherani **MAHIE** osetimni ma monuore koso mana dichiel?  1 Often  2 Sometimes  98 Don’t Know  99 Refused to Answer | GBV03:  Did you ever tell anyone that your **PRIMARY** partner did this, and if so who did you tell?  Bande nene ipimo ne ng’ato moro amora ni jaherani **MAHIE** notim kama, to kane ne ipimo, en ng’a mane ipimone?  1 Yes: _____________  2 No  98 Don’t Know  99 Refused to Answer |
| 1 Insulted or made you feel bad about yourself  Yanyi kata miyo iwinj marach kuomi in iwuon __________ |  |  |
| 2 Belittled or humiliated you in front of other people  Okawi matin, kata onjawi onyisi achaye e nyim ji _________ |  |  |
| 3 Done anything to scare or intimidate you on purpose (by the way he looked at you, by yelling, smashing things, etc.)  Otimo gimoro mondo obuogi kata omiyi luoro kuom gimoro (Kaka ne ong’iyi, ka ogoni koko, kata otoyo gik moko gi mamoko.) ____________ |  |  |
| 4 Threatened to hurt you or someone you care about  Obuogi ni onyalo hinyi kata hinyo ng’atmoro ma igeno ______ |  |  |
| 5 Slapped, hit, or thrown something at you that could hurt you  Opami kata obai gi gimoro ma ne nyalo hinyi ________ |  |  |
| 6 Pushed or shoved you  Odhiri kata owiti oko ____________ |  |  |
| 7 Kicked, dragged, or beaten you  Ogweyi, oywayi piny kata ogoyi ___________\ |  |  |
| 8 Strangled or burnt you on purpose  Odeyi kata owang’i nikech gimoroka ong’eyo ________ |  |  |
| 9 Threatened or has actually used a gun, knife, or other weapon that could hurt you  Obwogi kata otiyo kod bunde, pala kata gir lweny moro ma ne nyalo hinyi _________ |  |  |
| 10 Fondled, groped, grabbed, or touched you in a way that was unwanted or made you feel unsafe  10 Ne omaki, oywayi, orundi kata omuli e yoo ma ok owinjore ma omiyo ibedo ma onge kwe. __________ |  |  |
| 11 Forced you to have sex when you did not want to or could not provide consent (ex. you were too drunk or passed out)  Ochuni mondo ibedi kode e achiel ka in chunyi ok dwar kata ok openji kaka ineno(kuom ranyisi e seche ma imer ahinya kata ma ok inyal golo paro) __________________ |  |  |
| **Section F2. NON-PRIMARY PARTNERS** | | |
| GBV04:  In the past 12 months has a **NON-PRIMARY** sexual partner done any of the following to you?  E kinde mar dweche 12 mokalo, bende jaherani MAOK MAHIE osetimo gik maluwogi ne in?  1 No 🡪 skip to next item  2 Yes 🡪 ask GBV05 and GBV06  99 Refused to answer 🡪 skip to next item  *Interviewer: Read each option aloud one at a time and choose all that apply.*  *Interviewer: Read each option aloud one at a time and choose all that apply.* | GBV05:  In the past 12 months, has your **NON-PRIMARY** partner done this often or only sometimes?    E kinde mar dweche 6 mokalo, bende jaherani **MA OK MAHIE** osetimni ma monuore koso mana dichiel?  1 Often  2 Sometimes  98 Don’t Know  99 Refused to Answer | GBV06:  Did you ever tell anyone that your **NON-PRIMARY** partner did this, and if so who did you tell?  Bende nene ipimo ne ng’ato moro amora ni jaherani **MA OK MAHIE** notim kama, to kane ne ipimo, en ng’a mane ipimone?  1 Yes: _____________  2 No  98 Don’t Know  99 Refused to Answer |
| 1 Insulted or made you feel bad about yourself  Oyanyi kata miyo iwinj marach kuomi in iwuon _______ |  |  |
| 2 Belittled or humiliated you in front of other people  Okawi matin, kata onjawi onyisi achaye e nyim ji __________ |  |  |
| 3 Done anything to scare or intimidate you on purpose (by the way he looked at you, by yelling, smashing things, etc.)  Otimo gimoro mondo obuogi kata omiyi luoro kuom gimoro (Kaka ne ong’iyi, ka ogoni koko, kata otoyo gik moko gi  mamoko.) ______________ |  |  |
| 4 Threatened to hurt you or someone you care about  Obuogi ni onyalo hinyi kata hinyo ng’atmoro ma igeno. _____ |  |  |
| 5 Slapped, hit, or thrown something at you that could hurt you  5Opami kata obai gi gimoro ma ne nyalo hinyi _________ |  |  |
| 6 Pushed or shoved you  Odhiri kata owiti oko ___________ |  |  |
| 7 Kicked, dragged, or beaten you  Ogweyi, oywayi piny kata ogoyi ________ |  |  |
| 8 Strangled or burnt you on purpose  Odeyi kata owang’i nikech gimoroka ong’eyo _______ |  |  |
| 9 Threatened or has actually used a gun, knife, or other weapon that could hurt you  9Obwogi kata otiyo kod bunde, pala kata gir lweny moro ma ne nyalo hinyi that could hurt you __________ |  |  |
| 10 Fondled, groped, grabbed, or touched you in a way that was unwanted or made you feel unsafe  10Ne omaki, oywayi, orundi kata omuli e yoo ma ok owinjore ma omiyo ibedo ma onge kwe. ________ |  |  |
| 11 Forced you to have sex when you did not want to or could not provide consent (ex. you were too drunk or passed out)  11Ne obet e achiel e ringruok kodi kata ne otemo bedo e achiel e ringruok kodi kane ok inyal chiwo yieni nikech ne imer ahinya, ne imer matin, oketni gima meri, kata pachi ne olal ________ |  |  |

| ***The next questions should be asked if participants responded “yes” to ANY of the items above in Sections F1 or F2.*** | | |
| --- | --- | --- |
| GBV07 | Do you think any of these things were the result of participating in Jikinge study?  Bende iparo ni moko kuom magi otudore kod chiwrukni e nonro mar jikinge? | O Yes  O No  O Don’t Know  O Refused to answer |
| GBV08 | ***Ask only if arm= “Intervention”***  Do you think any of these things were the result of discussing HIV testing or offering an HIV self-test to your partner?  Bende iparo ni moko kuom magi en nikech twak ewi pimo kute mag Ayaki kata chiwo gir pim mar kute mag Ayaki ma nga’to pimorego kende owuon ne jaherani? | O Yes  O No  O Don’t Know  O Refused to answer |
| GBV09 | ***Ask only if arm= “Control”***  Do you think any of these things were the result of discussing HIV testing or offering a VCT referral card to your partner?  Bende iparo ni moko kuom magi en nikech twak ewi pimo kute mag Ayaki kata chiwo kadi mar gwelo ne jaherani? | O Yes  O No  O Don’t Know  O Refused to answer |

| **Section G1: HIV self-testing (Intervention Group Only)** | | |
| --- | --- | --- |
| *Read to participants in* ***intervention*** *group:* “I would like to ask you about your experience with the HIV self-tests that you received as part of the Jikinge study in the past 6 months.”  Daher penji e wi gigo ma iseneno kata kale kuom gig pimo kute mag ayaki ma ng’ato pimore kende owuon mane omiyi kowuok kuom nonro mar Jikinge e dweche 6 mokalo | | |
| **NO.** | **QUESTIONS** | **CODING CATEGORIES** |
| HST01 | Please tell me how many self-tests you have received **in the past 6 months**?  Akwayi mondo inyisa kwan mar gik pim ma nga’to pimorego kende owuon ma iseyudo e kinde mar dweche auchiel ma okalo?  *Interviewer*: *If the woman says “Don’t know” please probe to see if she remembers receiving the tests, and if so, if she can make a guess about how many she received.* | ___ ___  O Don’t know/recall  O Refused to answer |
| HST02 | Please tell me how many self-tests you have received **in the past month**?  Akwayi mondo inyisa kwan mar gik pim ma nga’to pimorego kende owuon ma iseyudo e kinde mar dwe achiel ma okalo?  *Interviewer*: *If the woman says “Don’t know” please probe to see if she remembers receiving the tests, and if so, if she can make a guess about how many she received.* | ___ ___  O Don’t know/recall  O Refused to answer |
| HST03 | In the past 6 months, how many of HIV self-tests have you used to test yourself?  Kuom dweche auchiel ma okalo, gin gik pim ma nga’to pimorego kende owuon adi ma isetiyogo in iwuon? | ___ ___  O Don’t know  O Refused to answer |
| HST04 | In the past 6 months, have you given any HIV self-tests to somebody else, such as your husband, sexual partners, friends, or family members?  Kuom dweche auchiel ma okalo, bende isechiwo gik pim ma nga’to pimorego kende owuon ne ng’at machielo kaka chwori, joherani moko, osiepeni,kata anyuolani?  *Interviewer*: Probe and include friends, co-workers etc. Make sure to ask participant to remember **all** self-tests that she gave to somebody else (even if that person did not end up using it) | O YES  O NO 🡪Skip to HST11  O Don’t know  O Refused to answer |
| HST05 | In the past 6 months, about how many self-tests did you give to sexual partners?  Kuom dweche auchiel ma okalo, gin gik pim ma nga’to pimorego kende owuon adi ma isechiwo ne joherani?  *Interviewer: Remind participant that if she gave 2 tests to the same partner, that should count as 2 test given.* | ___ ___ (if 00, skip to HST08)  O Don’t know  O Refused to answer |
| HST06 | In the past 6 months, please tell me all the types of sexual partners whom you gave self-tests to.  Kuom dweche auchiel ma okalo, nyisa kuom johera ma opogore opogore ma isechiwonegi gik pim ma nga’to pimorego kende owuon?  *Interviewer – select all that apply* | O Primary sexual partner  O Sexual partner who is not your primary sexual partner  O Commercial sex partner (client/ transactional sex partner)  O Don’t know  O Refused to answer |
| HST07 | Of all the sexual partners whom you gave self-tests to in the past 6 months, how many received an HIV-positive result (either directly observed by you or otherwise)?  Kuom johera duto mane ichiwonegigik pim ma nga’to pimorego kende owuon kuom dweche auchiel ma okalo, adi ma duokogi ne nyiso ni kod kute mag Ayaki (ma ineno kata ma ing’eyo e yo machielo)? | ___ ___  O Don’t know  O Refused to answer |
| HST08 | In the past 6 months, about how many self-tests did you give to other individuals who are not your sexual partners?  Kuom dweche auchiel ma okalo, gin gik pim ma nga’to pimorego kende owuon adi mane imiyo jomoko ma ok gin jok ma ibetgo e achiel e ringruok? | ___ ___ (if 00, skip to HST11)  O Don’t know  O Refused to answer |
| HST09 | In the past 6 months, please tell me all the types of other individuals who you gave self-tests to.  Kuom dweche auchiel ma okalo, yie mondo ipimna ewi ji ma opogore opogore mane imiyo gik pim ma nga’to pimorego kende owuon ?  *Interviewer – select all that apply* | O A male adult family member  O A female adult family member  O A child in your family  O A male friend  O A female friend  O Coworker or Peer  O Don’t know  O Refused to answer |
| HST10 | Of all the other individuals whom you gave self-tests to in the past 6 months, how many received an HIV-positive result (either directly observed by you or otherwise)?  Kuom ji duto mamoko mane ichiwonegi gik pim ma nga’to pimorego kende owuon kuom dweche auchiel ma okalo, adi mane duokogi onyiso ni gin kod kute mag Ayaki (ma ineno kata ma ing’eyo e yo machielo)? | ___ ___  O Don’t know  O Refused to answer |
| **Relationship and sexual behavior change** | | |
| HST11 | In the past 6 months, have you or any of your partners decided to end a sexual relationship, either temporarily or permanently?  Kuom dweche auchiel ma okalo, bende in kata achiel kuom joherani osedwaro chungo tudruok mar bet e achiel e ringruok matin kata chutho? | O YES  O NO 🡪 skip to HST15  O Don’t know 🡪 skip to HST15  O Refused to answer 🡪 skip to HST15 |
| HST12 | In the past 6 months, with how many partners was the relationship ended?  Kuom dweche auchiel ma okalo, tudruogeni adi mane orumo chutho? | ___ ___ (cannot equal 00)  O Don’t Know  O Refused to Answer |
| HST13 | Who decided to end these relationships?  En ng’a mane onga’do wach mondo tudruok orum?  *Interviewer: Choose all that apply for all of the relationships mentioned.* | O Participant  O Partner  O Don’t Know  O Refused to Answer |
| HST14 | Why did you or your partner(s) decide to end these relationship?  En ang’o mane omiyo in kata jaherani onga’do ni mondo tudruok maru orum?  *Interviewer: Choose all that apply for all of the relationships mentioned.* | O He refused to use an HIV self-test  O He refused to go to VCT  O He tested HIV-positive  O Physical abuse  O Verbal abuse  O Financial support ended  O Relocation of the partner or the participant  O Non-HIV related relationship problems (outside partners, trust issues, new marriage, etc.)  O Other (specify): __________________  O Don’t Know  O Refused to Answer |
| HST15 | In the past 6 months, have there been any partners with whom you declined to have sex with after they refused to accept a self-test or tested HIV-positive?  Kuom dweche auchiel ma okalo, bende nitiere jahera ma ne itamori betgo e achiel e ringruok nikech ne gitamore rwako gir pim mar kute mag Ayaki nga’to pimorego kende owuon kata nikech duokogi ne onyiso ni gin kod kute mag Ayaki? | O YES  O NO 🡪 skip to HST17  O Don’t know 🡪 skip to HST17  O Refused to answer 🡪 skip to HST17 |
| HST16 | In the past 6 months, how many partners did you decline to have sex with after they refused to accept a self-test or tested HIV-positive?  Kuom dweche auchiel ma okalo, gin johera adi mane itamori bedtge e achiel e ringruok nikech ne gitamore rwako gir pim mar kute mag Ayaki nga’to pimorego kende owuon kata nikech duokogi ne onyiso ni gin kod kute mag Ayaki? | ___ ___  O Don’t Know  O Refused to Answer |
| HST17 | In the past 6 months, have there been any partners with whom you decided to use a condom when having sex after they refused to accept a self-test or tested HIV-positive?  Kuom dweche auchiel ma okalo, bende nitie johera mane ibetgo e achiel e ringruok kaitiyogo kod rabo oyungabang’ kagisetamore rwako gir pim mar kute mag Ayaki nga’to pimorego kende owuon kata nikech duokogi ne onyiso ni gin kod kute mag Ayaki? | O YES  O NO 🡪 skip to NOTE  O Don’t know 🡪 skip to NOTE  O Refused to answer 🡪 skip to NOTE |
| HST18 | In the past 6 months, how many partners did you decide to use a condom with after they refused to accept a self-test or tested HIV-positive?  Kuom dweche auchiel ma okalo, gin johera adi mane ibedogo e achiel e ringruok ka itiyo gi rabo oyunga bang’ kane gitamore rwako gir pim mar kute mag Ayaki nga’to pimorego kende owuon kata nikech duokogi ne onyiso ni gin kod kute mag Ayaki? | ___ ___  O Don’t Know  O Refused to Answer |

| **Section G2: VCT Referral Cards (Control Group Only)** | | |
| --- | --- | --- |
| *Read to participants in intervention group:* “I would like to ask you about your experience with the VCT referral cards that you received as part of the Jikinge in the past 6 months.”  Daher penji ewi lony kod kadi ma ioro go ji e VCT mane omiyi kowuok kuom nonro mar Jikinge e dweche 6 mokalo.” | | |
| **NO.** | **QUESTIONS** | **CODING CATEGORIES** |
| VCT01 | Please tell me how many VCT Referral Cards you have received **in the past 6 months**?  **Akwayo ni mondo inyisa kwan mag** kadi ma ioro go ji e VCT **ma iseyudo kuom dweche auchiel ma okalo**?  *Interviewer*: *If the woman says “Don’t know” please probe to see if she remembers receiving the tests, and if so, if she can make a guess about how many she received.* | ___ ___  O Don’t know/recall  O Refused to answer |
| VCT02 | Please tell me how many VCT Referral Cards you have received **in the past month**?  **Akwayo ni mondo inyisa kwan mag** kadi ma ioro go ji e VCT **ma iseyudo kuom dwe achiel ma okalo**?  *Interviewer*: *If the woman says “Don’t know” please probe to see if she remembers receiving the tests, and if so, if she can make a guess about how many she received.* | ___ ___  O Don’t know/recall  O Refused to answer |
| VCT03 | In the past 6 months, how many of HIV VCT Referral Cards have you used yourself to go test at a VCT clinic?  Kuom dweche auchiel ma okalo, gin kadi ma ioro go ji e VCT adi mane itiyogo in iwuon e kar pim mar kute mag Ayaki? | ___ ___  O Don’t know  O Refused to answer |
| VCT04 | In the past 6 months, have you given any VCT Referral Cards to somebody else, such as your husband, sexual partners, friends, or family members?  Kuom dweche auchiel ma okalo, bende isechiwo kadi ma ioro go ji e VCT ne nga’t machielo kaka chwori, joherani ma ubetgo e achiel e ringruok, osiepeni kata anyuolani?  *Interviewer: Probe and include friends, co-workers etc. Make sure to ask participant to remember* ***all*** *VCT Referral Cards that she gave to somebody else (even if that person did not end up using it)* | O YES  O NO 🡪 skip to VCT11  O Don’t know 🡪 skip to VCT11  O Refused to answer 🡪 skip to VCT11 |
| VCT05 | In the past 6 months, about how many VCT Referral Cards did you give to a sexual partner?  Kuom dweche auchiel ma okalo, gin madirom kadi ma ioro go ji e VCT adi mane imiyo jaherani ma ibetgo e achiel e ringruok?  *Interviewer: Remind participant that if she gave 2 cards to the same partner, that should count as 2 cards given.* | ___ ___  O Don’t know  O Refused to answer |
| VCT06 | In the past 6 months, please tell me all the types of sexual partners whom you gave VCT Referral Cards to.  Kuom dweche auchiel ma okalo, akwayi ni mondo inyisa kwan mar joherani ma opogore opogore mane imiyo kadi ma ioro go ji e VCT?  *Interviewer – select all that apply* | O Primary sexual partner  O Sexual partner who is not your primary sexual partner  O Commercial sex partner (client or transactional sex partner)  O Don’t know  O Refused to answer |
| VCT07 | Of all the sexual partners whom you gave VCT Referral Cards to in the past 6 months, how many received an HIV-positive result at the VCT clinic (either directly observed by you or otherwise)?  Kuom joherani duto ma ibetgo e achiel e ringruok mane imiyo kadi ma ioro go ji e VCT e dweche auchiel ma okalo, adi mane ma duokogi ne nyiso ni gin kod kute mag Ayaki e kar pimo kute mag Ayaki? (mane ineno kata ing’eyo e yo machielo) | ___ ___  O Don’t know  O Refused to answer |
| VCT08 | In the past 6 months, about how many VCT Referral Cards did you give to other individuals who are not your sexual partners (e.g. family and friends)?  Edweche 6 mokalo, ne ichiwo kadi ma ioro go ji e VCT madirom adi ne jomamoko ma ok gin joherani ma ibedogago e achiel e ringruok (kuom ranyisi joot kod osiepe)? | ___ ___ (if 00, skip to VCT11)  O Don’t know  O Refused to answer |
| VCT09 | In the past 6 months, please tell me all the types of other individuals who you gave VCT Referral Cards to.  Kuom dweche auchiel ma okalo, gin kadi ma ioro go ji e VCT adi mane imiyo jomamoko ma ok gin joherani ma ibetgo e achiel e ringruok(kuom ranyisi joot kata osiep)?  *Interviewer – select all that apply* | O A male adult family member  O A female adult family member  O A child in your family  O A male friend  O A female friend  O Coworker or Peer  O Don’t know  O Refused to answer |
| VCT10 | Of all the other individuals whom you gave VCT Referral Cards to in the past 6 months, how many received an HIV-positive result (either directly observed by you or otherwise)?  Kuom jomamoko ma ok gin joherani ma ibetgo e achiel e ringruok mane imiyo kadi ma ioro go ji e VCT e dweche auchiel ma okalo, adi mane ma duokogi ne nyiso ni gin kod kute mag Ayaki (mane ineno kata ing’eyo e yo machielo)? | ___ ___  O Don’t know  O Refused to answer |
| VCT11 | In the past 6 months, did any of your sexual partners refuse to accept self-tests from you?  Kuom dweche auchiel ma okalo, bende nitiere jaherani moro amora ma ibetgo e achiel e ringruok mane otamore rwako kadi ma ioro go ji e VCT mane imiye? | O YES  O NO  O Don’t know  O Refused to answer |
| VCT12 | In the past 6 months, have you received or used oral HIV self-tests kits?  Kuom dweche auchiel ma okalo, bende iseyudo kata tiyo gi gir pim mar kute mag Ayaki nga’to pimorego kende owuon? | O YES  O NO 🡪 Skip to VCT14  O Don’t know 🡪 skip to VCT14  O Refused to answer 🡪 skip to VCT14 |
| VCT13 | From which person or place did you receive the oral HIV self-test kits?  Ka owuok kuom ng’a kata Kanye mane iyudoe gir pim mar kute mag Ayaki nga’to pimorego kende owuon? | O Friend or family member  O Purchased  O Clinic or hospital  O Another research study  O Other (specify): __________________ |
| **Relationship and sexual behavior change** | | |
| VCT14 | In the past 6 months, have you or any of your partners decided to end a sexual relationship, either temporarily or permanently?  Kuom dweche auchiel ma okalo, bende in kata joherani moro amora ma ibetgo e achiel e ringruok ne ong’ado wach mondo oketh tudruok mari kode e kinde matin kata chutho? | O YES  O NO 🡪 skip to VCT18  O Don’t know 🡪 skip to VCT18  O Refused to answer 🡪 skip to VCT18 |
| VCT15 | In the past 6 months, with how many partners was the relationship ended?  Kuom dweche auchiel ma okalo, gin jaherani adi ma ibetgo e achiel e ringruok mane tudruokni kodgi okethore? | ___ ___ (cannot equal 00)  O Don’t Know  O Refused to Answer |
| VCT16 | Who decided to end these relationships?  En ng’a mane ong’ado wach mar ketho tudruok? | O Participant  O Partner  O Don’t Know  O Refused to Answer |
| VCT17 | Why did you or your partner decide to end the relationship(s)?  En ang’o mane omiyo in kata jaherani ne ong’ado wach mar ketho tudruok? | O He refused to use an HIV self-test  O He refused to go to VCT  O He tested HIV-positive  O Physical abuse  O Verbal abuse  O Financial support ended  O Relocation of the partner or the participant  O Non-HIV related relationship problems (outside partners, trust issues, new marriage, etc.)  O Other (specify)  O Don’t Know  O Refused to Answer |
| VCT18 | In the past 6 months, have there been any partners with whom you declined to have sex with after they refused to accept a VCT referral card or after they tested HIV-positive?  Kuom dweche auchiel ma okalo, bende nitiere jahera mane itamori betgo e achiel e ringruok nikech ne gitamore rwako kadi mar gwelo kata nikech duokogi ne onyiso ni gin kod kute mag Ayaki? | O YES  O NO🡪 skip to VCT20  O Don’t know 🡪 skip to VCT20  O Refused to answer 🡪skip to VCT20 |
| VCT19 | In the past 6 months, how many partners did you decline to have sex with after they refused to accept a VCT referral card or after they tested HIV-positive?  Kuom dweche auchiel ma okalo, gin johera adi maneitamori betgo e achiel e ringruok nikech ne gitamore rwako kadi ma ioro go ji e VCT kata nikech duokogi ne onyiso ni gin kod kute mag Ayaki? | ___ ___  O Don’t Know  O Refused to Answer |
| VCT20 | In the past 6 months, have there been any partners with whom you decided to use a condom when having sex after they refused to accept a VCT referral card or after they tested HIV-positive?  Kuom dweche auchiel ma okalo, bende nitiere jahera mane itiyogo gi rabo oyunga ka ubet e achiel e ringruok bang’ ka gisetamore rwako kadi mar gwelo kata nikech duokogi ne onyiso ni gin kod kute mag Ayaki? | O YES  O NO 🡪skip to NOTE  O Don’t know 🡪 skip to NOTE  O Refused to answer 🡪 skip to NOTE |
| VCT21 | In the past 6 months, how many partners did you decide to use a condom with after they refused to accept a VCT referral card or after they tested HIV positive?  Kuom dweche auchiel ma okalo, gin joherani adi mane itiyogo gi rabo oyunga ka ubet e achiel e ringruok bang’ ka gisetamore rwako kadi ma ioro go ji e VCT kata nikech duokogi ne onyiso ni gin kod kute mag Ayaki? | ___ ___  O Don’t Know  O Refused to Answer |

| **Section H: Interviewer’s Observations (NOTE)**  *Interviewer: Complete at the end of the interview* |
| --- |
|  |

| **Section I: HIV Rapid Test and DBS Collection**  Perform HIV antibody rapid testing and enter results. If positive, alert Study Coordinator to pull DBS card collected at Baseline for confirmatory testing. | | |
| --- | --- | --- |
| hiv | HIV rapid test result | O Negative  O Positive  O Indeterminate  O Woman refused testing |

**END OF FOLLOW-UP QUESTIONNAIRE**
