# Supplementary material for: Adoption of HIV pre-exposure prophylaxis among women at high risk of HIV infection in Kenya
Source: PLoS One. 2022 Sep 9;17(9):e0273409. doi: 10.1371/journal.pone.0273409 (PMC9462728; doi:10.1371/journal.pone.0273409)
Supplement: S5 File — (DOC) [file pone.0273409.s005.doc]

**6-monthly follow-up visit questionnaire**

| **Section A1** | | |
| --- | --- | --- |
| **No.** | **Question** | **Responses** |
| intid | Interviewer ID | ___ ___ |
| date | Date of Interview | ___ ___/___ ___ ___/ ___ ___ ___ ___  D D M M M Y Y Y Y |
| clusterid | Enter Cluster ID | ___ ___ ___ |
| cluster | Enter Name of Cluster |  |
| location | Enter Name of Hotspot or Beach |  |
| arm | Intervention or control cluster? | 1 Intervention  2 Control |
| month | What month of follow-up is this appointment? | O Month 6  O Month 12  O Month 18  O Month 24 |
| **Section A2: Participant Identification** | | |
| pid | Fingerprint scan of participant  *Enter the participant’s ID number. Be extremely careful when entering the number. Double and triple check that the number is correct before proceeding.* | ___ ­___ ___ ___ ___ ___ ___ ___ ___  If automatic fingerprint works, skip to section A3 |
| pid | Repeat manual Participant ID number (PID)  Verify the PID number | ___ ­___ ___ ___ ___ ___ ___ ___ ___ |

| **Section B: Demographic questions (DMG)** | | |
| --- | --- | --- |
| **No.** | **Question** | **Coding** |
| DMG01 | I’d like to ask you again, what is your age?  Ningependa kuku uliza tena, una umri wa miaka mingapi? | ___ ___  O Refused to Answer |
| DMG02 | Have you permanently moved or relocated away from this study area in the past 6 months?  Je umehama kabisa kutoka mahala pa utafiti kwa miezi 6 zilizopita?  *Interviewer: ensure that “study area” is clearly explained to mean the cluster/location where the participant was enrolled* | O Yes  O No 🡪 Skip to DMG04  O Refused to Answer 🡪 Skip to DMG04 |
| DMG03 | Where do you reside now?  Unaishi wapi kwa sasa? | Cluster code: ___ ___ ___  O Outside of study area: __________________  O Don’t Know  O Refused to Answer |
| DMG04 | Besides Jikinge, have you enrolled in any other research studies in the past 6 months where you were receiving an intervention such as income, medicine, HIV test kits, condoms, or counseling? Your answer to this question will not impact your continued participation in the Jikinge study.  Tofauti na Jikinge, umewahi jiunga na tafiti zozote kwa muda wa miezi sita zilizopita ambapo ulikuwa unapokea huduma kama mapato, dawa ,vifaa vya kujipima virusi vya ukimwi, kondomu au ushauri? Majibu yako kwa swali hili halitaadhiri kuendelea kwako katika utafiti wa Jikinge. | O Yes  O No 🡪 Skip to DMG06  O Don’t Know 🡪 Skip to DMG06  O Refused to Answer 🡪 Skip to DMG06 |
| DMG05 | What did you receive as part of your participation in the other HIV research study?  Je, ulipokea nini kama kipande cha ushiriki wako katika utafiti zingine za VVU?  *Choose all that apply* | O HIV test kits  O Condoms and/or lubricant  O PrEP  O Cash transfers  O Other (specify): _________________________  O Don’t Know  O Refused to Answer |
| DMG06 | What is your current marital status?  Je, hali yako yandoa kwa sasa ni gani? | O Married, Living Together  O Married, NOT Living Together  O Not Married, Living Together  O Relationship but Not Married, NOT Living Together (boyfriend, etc.)  O Single  O Divorced  O Widowed  O Don’t Know  O Refused to Answer |
| DMG07 | Has your marital status changed in the last 6 months?  Hali yako ya ndoa imewahi badilika kwa miezi sita zilizopita? | O No change 🡪Skip to DMG10  O I am no longer in a relationship  O I am now married  O I am now in a relationship but not married  O I am still married, but now live with my husband  O I am still married or in a relationship, but no longer live with my partner (separated)  O I have divorced  O I have become a widow 🡪Skip to DMG10  O Refused to Answer |
| DMG08 | Was the change in your marriage related to HIV testing in any way, such as finding out your HIV status or a partner’s HIV status?  Je, tofauti iliyopo katika ndoa yako inahusiana na upimaji wa VVU kwa njia yoyote kama vile kutambua hali yako ya virusi vya ukimwi au hali ya VVU ya mpenzi wako?  *Choose all that apply.* | O Yes, my HIV status  O Yes, my partner’s HIV status  O Yes, partner’s refusal to test for HIV  O Yes, other (specify)  O No  O Don’t Know  O Refused to Answer |
| DMG09 | Do you think your participation in Jikinge study played some role in the change in your marital status?  Je, unafikiria kushiriki kwako katika utafiti wa Jikinge ilichangia kwa baadhi ya mabadiliko katika hali yako ya ndoa?” | O Yes (specify)  O No  O Don’t Know  O Refused to Answer |
| DMG10 | What is your **primary** source of income?  Njia yako **kuu** ya mapato ni ipi? | O Professional/salaried  O Rental income (landlord, rent equipment)  O Sales and Service (Non-Fish)  O Skilled Manual  O Unskilled Manual  O Domestic Service  O Agriculture  O Fishing/ Fish Trade  O Sex Work  O Informal/Seasonal/ Piece Work  O Student  O Unemployed  O Other (specify)  98 Don’t Know  99 Refused to Answer |
| DMG11 | What is/are your OTHER source(s) of income, if any?  Njia yako nyingine ∕zako zingine za mapato ni zipi? Kama ziko?  *Interviewer: mark all that apply* | O Professional/salaried  O Rental income (landlord, rent equipment)  O Sales and Service (Non-Fish)  O Skilled Manual  O Unskilled Manual  O Domestic Service  O Agriculture  O Fishing/ Fish Trade  O Sex Work  O Informal/Seasonal/ Piece Work  O Student  O Other (specify)  O None/ Not Applicable  98 Don’t Know  99 Refused to Answer |
| DMG12 | How much income do you typically earn in one month, from all sources?  Je,mapato yako kwa jumla unayopata kwa mwezi mmoja ni ngapi, kutokana na njia zako zote za mapato ?  *Interviewer: Ask participant to give best estimate if not sure* | KES ___________________  O Don’t Know  O Refused to Answer |

| **Section C1: General Health (HLT)** | | |  |
| --- | --- | --- | --- |
| *Interviewer: I have some questions about your physical and mental health.* | | |  |
| **No.** | **Question** | **Coding** |  |
| HLT01 | How would you rate your overall health?  Je unaweza sema afya yako kwa jumla iko vipi? | O Very Good  O Good  O Fair  O Poor  O Don’t Know  O Refused to Answer |  |
| HLT02 | In the past 6 months, what is your best estimate of how often you drank alcohol?  Je, kwa miezi 6 iliyopita unaweza kadiria umekunywa pombe mara ngapi? | O Never (skip to HLT04)  O Less than once per month  O Once a month  O 2 to 3 times per month  O Once per week  O Twice per week  O 3 to 4 times a week  O 5 to 6 times per week  O Every Day  O Don’t Know  O Refused to Answer |  |
| HLT03 | In the past month, how often did you drink 5 or more alcoholic drinks in one night?  Je kwa mwezi moja uliopita, ni mara ngapi ulikunywa vinywaji vya kulewesha chupa 5 au zaidi kwa usiku mmoja? | O Never  O Only once  O 2 or 3 times  O Once per week  O Twice per week  O 3 to 4 times a week  O 5 to 6 times per week  O Every Day  O Don’t Know  O Refused to Answer |  |
| HLT04 | In the past 6 months, have you become pregnant?  Kwa miezi sita zilizopita umewahi kupata mimba? | O Yes  O No  O Don’t Know  O Refused to Answer | |

| **Section C2: Mental health (PHQ-9)**  **Skip this section if month=6 or 18; ask only if month=12 or 24** | |
| --- | --- |
| HLT05:  Over the last 2 weeks, have you been bothered by any of the following problems:  Kwa wiki 2 zilizopita, je, umewai sumbuliwa na shida zozote zifuatazo?    O No 🡪 skip to next item  O Yes 🡪 ask HLT06  O Don’t know 🡪 skip to next item  O Refused to answer 🡪 skip to next item  ***Interviewer:*** *Read each item aloud one at a time and read out the 5 frequency choices for each item.* | HLT 06:  Over the last 2 weeks, how often have you been bothered by this problem?  Kwa wiki 2 zilizopita, je, ni mara ngapi umesumbuliwa na shida zozote zifuatazo?  ***Interviewer:*** *Read each item aloud one at a time and read out the 5 frequency choices:* One Day, Less than Half the Days, Around Half the Days, More than Half the Days, Every Day  O One Day  O Less than Half the Days  O Around Half the Days  O More than Half the Days  O Every Day  O Don’t Know  O Refused to Answer |
| 1.Had little interest or pleasure in doing things    Kutokuwa na hamu ya kufanya vitu |  |
| 2. Felt down, depressed, or hopeless  Kujihisi uko chini, kuhuzunika, au kukosa matumaini |  |
| 3. Had trouble falling asleep/staying awake, or sleeping too much  Kuwa na shida kuanza kulala / kulala au kulala sana |  |
| 4. Felt tired or having little energy  Kujisikia mchovu au kuwa na nguvu kidogo. |  |
| 5. Had poor appetite or overeating  Kutokuwa na hamu ya kula au kula zaidi. |  |
| 6. Felt bad about yourself, felt that you are a failure, or felt that you let yourself or your family down  Kuhisi vibaya juu ya nafsi yako binafsi, kuhisi kuwa hujatimiza matakwa yako, ama ulijihisi kuwa haujafikisha matarajio yako au ya familia yako. |  |
| 7. Had trouble concentrating on things such as reading the newspaper or watching television  Kuwa na shida kuwa makini kwa vitu vyengine kama kusoma gazeti au kutazama runinga |  |
| 8. Moved or spoken so slowly that other people could have noticed, or been so fidgety/restless that you have moved around a lot more than usual  Kutembea au kuongea kwa upole mpaka watu wengine wanaweza kutambua, au kuwa na wasiwasi kwa kuwa umetembea sana kuliko kawaida |  |
| 9. Had thoughts that you would be better off dead, or of hurting yourself in some way  Kuwa na fikira kwamba heri ufe au kujiumiza kwa njia yoyote. |  |

| HLT07 | How difficult have these problems made it for your to do your work, take care of things at home, or get along with other people?  Je shida hizi zimekuzuia vipi kufanya kazi zako, kufanya vitu za nyumbani ama uhusiano wako na watu wengine? | O Not Difficult at All  O Somewhat Difficult  O Very Difficult  O Don’t Know  O Refused to Answer |
| --- | --- | --- |

| **Section C3: General Sexual Activity (GSA)**  Interviewer: Now I have some questions about your sexual activity, in order to get a better understanding of some important aspects of your life. For these questions, “sexual activity” is defined as sexual penetration of the vagina or anus.  Interviewer: Kwa sasa niko na maswali kadhaa kuhusu ushiriki wako wa ngono, ili nipate kuelewa vizuri maneno kadhaa muhimu ya maisha yako. Kwa maswali haya, “kushiriki ngono” inafafanuliwa kama ngono kupitia sehemu ya mwanamke ya siri au sehemu ya haja kubwa. | | |
| --- | --- | --- |
| GSA01 | During the past month, how many different sexual partners have you had?  Kwa mwezi uliopita umekuwa na wapenzi wangapi tofauti wa kushiriki ngono? | ___ ___  O Don’t Know  O Refused to Answer |
| GSA02 | The last time you had sex, did you use a condom?  Mara ya mwisho uliposhiriki ngono, je ulitumia mpira wa kondomu? | O Yes  O No  O Don’t Know  O Refused to Answer |

| **Section C4: Primary Partner (PRP)**  Interviewer: Now I have some questions about your **one** primary or regular sexual partner. A primary partner could be your husband, boyfriend, or someone you **regularly** have sex with. A primary partner is the ONE partner you feel like you love or like the most, or the one you can confide in the most. If you would like help deciding who your primary partner is, we can talk about that now.  Interviewer: Kwa sasa niko na maswali mengine kuhusu mshiriki wako mmoja wa karibu na wa mara kwa mara wa ngono. Mpenzi wakomkuu anaweza huenda akawa mumeo, mpenzi wako wa kiume au mtu unayeshiriki naye ngono . Mpenzi mkuu ni yule mshiriki unahisi unampenda sana, yule unayeweza mwelezea mambo yako ya kindani zaidi. Ikiwa unahitaji usaidizi kuamua ni nani mpenzi wako mkuu , tunaweza kuzungumzia hayo kwa wakati huu. | | |
| --- | --- | --- |
| PRP01 | Do you currently have a primary partner, or have you had one in the past month? This is the man we will discuss in the following questions.  Je kwa sasa una mpenzi mkuu wa ngono au umewahi kuwa na mmoja katika mwezi moja uliopita? Huyu mwanaume ndiye tutakayemuongelea kwa maswali yafwatayo | O Yes  O No (skip to NPP01)  O Refused to Answer (skip to NPP01) |
| PRP02 | Was this person also your primary partner 6 months ago?  Huyu mtu pia alikuwa mshiriki wako mkuu miezi sita zilizopita? | O Yes (skip to PRP05)  O No  O Refused to Answer |
| PRP03 | About how old is your current primary partner?  Je mpenzi wako mkuu wa sasa ana miaka mingapi?  *Interviewer: if participant is not sure, ask her to give best guess* | ___ ___ years  O Don’t Know  O Refused to Answer |
| PRP04 | How long have you had a sexual relationship with your current primary partner?  Je, umekuwa na uhusiano waki ngono na huyu mpenzi wako mkuu kwa muda gani?  *Interviewer: For relationships <1 month, record 01 months. (Ex: If participant says one week, record 01months)* | ___ ___ years, ___ ___ months  O Don’t Know  O Refused to Answer |
| PRP05 | Have you had sex with this partner in the past month?  Je umeshiriki ngono na huyu mpenzi kwa mwezi uliopita? | O Yes  O No 🡪Skip to PRP09  O Refused to Answer 🡪 skip to PRP09 |
| PRP06 | In the past month, how often did you use condoms with your primary partner?  Je ni mara ngapi umetumia condomu na mshiriki wako mkuu katika mwezi mmoja uliopita? | O Never  O Less than Half the Time  O About Half the Time  O More than Half of the Time  O Always (skip to PRP08)  O Don’t Know  O Refused to Answer |
| PRP07 | Why did you not use condoms all the time with your primary partner within the last month? Please tell me all the reasons why.  Kwa nini haukutumia mpira wa kondomu wakati wote na mpenzi wako mkuu kwa mwezi mmoja uliyopita? Tafadhali nielezee sababu zako zote  *Interviewer choose all that apply based on what the participant mentioned.* | O I Did Not Want To  O Partner Did Not Want To  O No Condom was Available  O I Am Trying To Become Pregnant  O I Have Another Form of Contraception (IUD, Implant, Injectable, Pill, etc.)  O I Knew My Primary Partner’s HIV Status and decided a condom was not necessary  O I trust my primary partner  O One or both of us is using PrEP  O Other (specify): __________________________  98 Don’t Know  99 Refused to Answer |
| PRP08 | In the past month, have you ever had difficulty *negotiating* condom use with your primary partner?  Kwa mwezi uliopita, je umewahi kuwa na ugumu wa kujadili /kushauriana juu ya matumizi ya mpira wa kondomu na mshiriki wako mkuu? | O Yes, always  O Yes, sometimes  O I have no difficulty negotiating  O I never try to get my partner to use condoms  O Don’t Know  O Refused to Answer |
| PRP09 | In the past 6 months, has your primary partner had an HIV test?  Kwa miezi 6 zilizopita, je, mpenzi wako mkuu amewahi enda kupimwa virusi vya ukimwi? | O Yes  O No 🡪 Skip to PRP11  O Don’t Know 🡪 Skip to PRP11  O Refused to Answer 🡪 Skip to PRP11 |
| PRP10 | Where did your partner get tested for HIV?  Mpenzi wako alipimwa VVU wapi?  *Interviewer:* ***remind participant that HIV self-testing is also a possible answer to this question****. Mark all that apply.* | O VCT clinic  O Hospital  O HIV self-test  O Home Testing  O Other (specify): _____________  O Don’t Know  O Refused to Answer |
| PRP11 | **Ask if arm= “Intervention” OR if response to PRP10 is “HIV self-test”**  Did you offer your partner an HIV self-test in the past 6 months?  Je ulimpatia mpenzi wako vifaa vya kujipima kibinafsi kwa miezi 6 zilizopita? | O Yes  O No 🡪 Skip to PRP21  O Don’t know 🡪 Skip to PRP21  O Refused to answer 🡪 Skip to PRP21 |
| PRP12 | Did your partner accept the self-test from you?  Je mpenzi alikubali kifaa cha kujipima kibinafsi kutoka kwako? | O Yes  O No 🡪 skip to PRP21  O Refused to Answer 🡪 skip to PRP21 |
| PRP13 | When did your partner use the self-test most recently?  Mpenzi wako alikitumia kifaa cha kujipima kibinafsi lini hivi karibuni?  *Interviewer: If partner used multiple HIV self-tests, record the time the most recent test was used.* | O Did not use 🡪 Skip to PRP21  O Less than 1 week ago  O Between 1 week and 1 month ago  O Between 1 and 3 months ago  O Between 3 and 6 months ago |
| PRP14 | Where did your partner use the self-test?  Mpenzi wako alikitumia kifaa cha kujipima kibinafsi wapi? | O At our home  O At my home  O At his/her home  O At workplace  O At friend’s house  O Lodge/hotel room  O In or around a bar (car outside, alleyway, etc.)”  O Other (specify): __________________________  O Don’t know  O Refused to answer |
| PRP15 | Please rate how easy or difficult it was for your partner to use the self-test? This could be based on what he told you, or what you directly observed.  Tafadhali nilielezee ilikuwa rahisi au ngumu kiasi kipi kwa mpenzi wako kutumia kifaa cha kujipima kibinafsi? Hii inaweza kuwa kitu alichokuambia au kile ulichokiona na macho  *INTERVIEWER: First get participant’s reaction and then read the choices that are most relevant and ask the participant which one fits best. This question is about use of self-test, not about acceptance of the self-test.* | O Very easy  O Somewhat easy  O Neutral  O Somewhat difficult  O Very difficult  O Don’t know  O Refused to answer |
| PRP16 | Were you present when your partner used the self-test?  Ulikuwepo wakati mpenzi wako alipokuwa akiki tumia kifaa cha kujipima kibinafsi? | O YES – I was present when he used the self-test  O NO – But he told me he used the self-test  O Don’t know  O Refused to answer |
| PRP17 | Did you learn your primary partner’s HIV status following the self-test?  Je uligundua hali ya mpenzi wako mkuu wa VVU kutokana na kujipima kibinafsi? | O No  O Yes, Partner is Positive  O Yes, Partner is Negative 🡪 Skip to PRP20  O Yes, Refuse to Disclose Partner’s Status  O Refused to Answer |
| PRP18 | Did your partner go to a clinic or health facility in order to confirm the result that was obtained from the self-test (confirmatory testing)?  Je, mpenzi wako alienda katika kliniki au kituo cha Afya kudhibitisha matokeo aliyopata akitumia kifaa cha kujipima kibinafsi(confirmatory testing)? | O Yes  O No  O Don’t know  O Refused to answer |
| PRP19 | Did your partner go to a clinic or health facility for HIV care?  Je, mpenzi wako alienda katika kliniki au kituo cha Afya kwa huduma ya VVU? | O Yes  O No  O Don’t know  O Refused to answer |
| PRP20 | At the **same** time that your partner used the self-test, did you **also** use a self-test?  Wakati mpenzi wako alipokitumia kifaa cha kujipima kibinafsi, je wewe pia ulikitumia chako? | O Yes, we tested together at the same time  O Yes, we both tested but not together  O No  O Other (specify): ______________________________  O Don’t know  O Refused to answer |
| ***Note: PRP21- PRP24 should be asked only if arm= “Control”*** | | |
| PRP21 | **Control group:**  Did you give your partner a VCT referral card in the past 6 months?  Je ulimpatia mpenzi wako kadi ya mwaliko kwa miezi 6 zilizopita? | O Yes  O No 🡪 Skip to PRP25  O Don’t know 🡪 Skip to PRP25  O Refused to answer 🡪 Skip to PRP25 |
| PRP22 | **Control group**:  Did your partner go to a clinic or health facility for HIV care?  Je, mpenzi wako alienda katika kliniki au kituo cha Afya kwa huduma ya VVU? | O Yes  O No 🡪 skip to PRP25  O Don’t Know 🡪 skip to PRP25  O Refused to answer 🡪 skip to PRP25 |
| PRP23 | **Control group**:  At the **same** time that your partner got tested for HIV, did you also get tested?  Wakati huo mpenzi wako alipopimwa VVU, wewe pia ulipimwa? | O Yes, we tested together at the same time  O Yes, we both tested but not together  O No  O Other (specify): ___________________________  O Don’t know  O Refused to answer |
| PRP24 | **Control group**:  Did you learn your primary partner’s HIV status following the HIV test?  Je uligundua hali ya mpenzi wako mkuu wa VVU kutokana na Upimaji wa VVU? | O No  O Yes, Partner is Positive  O Yes, Partner is Negative  O Yes, Refuse to Disclose Partner’s Status  O Refused to Answer |
| PRP25 | In the past 6 months, has there been a time when you took an HIV test and shared the result with your partner?  Kwa miezi 6 zilizopita, je kumekuwa na wakati ulipopimwa VVU na kumwambia mpenzi wako matokeo yako? | O Yes  O No  O Don’t Know  O Refused to answer |
| PRP26 | Did your sexual behavior with your partner change in the past 6 months?  Je tabia yako ya ngono na mpenzi wako ilibadilika kwa miezi 6 zilizopita? | O Yes  O No 🡪 Skip to NPP01  O Don’t know  O Refused to answer 🡪 Skip to NPP01 |
| PRP27 | In the past 6 months, have you stopped having sex with this partner for extended periods of time?  Kwa miezi 6 zilizopita ,umewacha kushiriki ngono na huyu mpenzi kwa muda?  *Interviewer: this should not include cases where one person was away for multiple weeks* | O Yes  O No  O Don’t Know  O Refused to Answer |
| PRP28 | How else has your sexual behavior with this person changed in the past 6 months?  Bainisha jinsi gani tena tabia yako ya ngono na huyu mtu ilibadilika katika miezi 6 zilizopita  *Mark all that apply.* | O Increased Condom Use  O Decreased Condom Use  O Increase amount or frequency of sex  O Decrease amount or frequency of sex  O Other, specify (Open Ended Response): |
| **Section C5: Non-Primary Partners (NPP)**  Interviewer: Now I have some questions about any other sexual partners you may have had other than your primary partner. For these questions, I am referring to boyfriends, casual sexual partners, or someone with whom you engage in transactional sex, i.e. sex for money, goods gifts, food or housing.  Sasa nina maswali mengine kuhusu mshiriki wako yeyote mwingine ambaye ulikuwa naye isipokuwa mshiriki wako wa karibu. Kwa maswali haya, namaanisha wapenzi wako wa kiume wapenzi wasiyokuwa wakuu, au mtu unayeshiriki naye ngono ya kufaidika ,kama vile pesa kwa ngono , bidhaa, zawadi, chakula au makazi . | | |
| NPP01 | How many non-primary sexual partners have you had in the past month?  Je,ni washiriki wangapi wasio washiriki wakuu wa ngono ulikuwa nao mwezi moja uliopita?  *Interviewer: probe for an estimate if participant cannot immediately recall* | ___ ___ (If 00, skip to TRX01)  O Don’t Know  O Refused to Answer (skip to TRX01) |
| NPP02 | Of these, approximately how many did you have **multiple** sexual encounters with in the past month?  Je kati ya hawa, ni kadri wangapi ulishiriki nao ngono **mara kadhaa kwa mwezi mmoja uliyopita?** | ___ ___ (If 00, skip to NPP05)  O Don’t Know  O Refused to Answer |
| NPP03 | Among those with whom you had multiple sexual encounters in the past month how often did you use condoms?  Kati ya wale ambao ulishiriki nao ngono mara kadhaa kwa mwezi uliyopita ni mara ngapi ulitumia mipira ya kondomu? | O Never  O Less than Half the Time  O About Half the Time  O More than Half of the Time  O Always (skip to NPP05)  O Don’t Know  O Refused to Answer |
| NPP04 | Why did you not always use condoms with those you shared multiple sexual encounters?  Je ni kwa nini haukutumia mpira wa kondomu kila mara na wale ulioshiriki nao ngono kwa mara kadhaa?  *Open ended for participants. Interviewer choose all that apply based on what the participant mentioned.* | O I Did Not Want To  O Partner(s) Did Not Want To  O No Condom Was Available  O I Am Trying to Become Pregnant  O I Have Another Form of Contraception (IUD, Implant, Injectable, Pill, etc.)  O I Knew My Non-Primary Partner’s HIV Status  O I trust my non-primary partner(s)  O Partner offered me more money to not use a condom  O Other (specify)  O Don’t Know  O Refused to Answer |
| NPP05 | Approximately how many non-primary sexual partners in the past month did you have sex with **only once**?  Je, ni kadri washiriki wangapi wasio washiriki wakuu wa ngono ulishiriki nao ngono mara **moja tu** kwa mwezi mmoja uliopita? | ___ ___ (If 00, skip to NPP08)  O Don’t Know  O Refused to Answer |
| NPP06 | Among the men with whom you had only one sexual encounter in the past month, how often did you use condoms?  Je, kati ya wanaume ambao ulishiriki nao ngono mara moja tu kwa mwezi, ni mara ngapi ulitumia kondomu? | O Never  O Less than Half the Time  O About Half the Time  O More than Half of the Time  O Always (skip to NPP08)  O Don’t Know  O Refused to Answer |
| NPP07 | Why did you not always use condoms with those you had only one sexual encounter?  Kwa nini haukuwa unatumia kondomu kila wakati na wale ambao ulishiriki nao ngono mara moja tu?  *Open ended for participants. Interviewer choose all that apply based on what participant mentioned.* | 1: I Did Not Want To  2: Partner(s) Did Not Want To  3. No Condom Was Available  4: I Am Trying To Become Pregnant  5: I Have Another Form of Contraception (IUD, Implant, Injectable, Pill, etc.)  6: I Knew My Non-Primary Partner/Partners’ HIV Status  7: I trust my non-primary partner(s)  8: Partner(s) offered me more money to not use a condom  9: Other (specify)  98: Don’t Know  99: Refused to Answer |
| Interviewer: I will now be asking you about all of the non-primary partners you have had in the past 6 months.  Sasa nitakuwa nakuuliza juu ya wapenzi wako wasio wakuu wenye umekuwa nao kwa miezi 6 iliyopita | | |
| NPP08 | Among the non-primary partners you have had in the past **6 MONTHS**, have you had sex without a condom with any of them?  Kati ya washiriki wasio wakuu umekuwa nao kwa MIEZI 6 zilizopita, umewahi shiriki ngono nao bila kutumia mpira wa kondomu? | O Yes  O No 🡪 Skip to NPP10 or NPP11  O Refused to answer 🡪 Skip to Npp10 or NPP11 |
| NPP09 | Among these men, have there been some whose HIV status you did **not** know?  Kati ya hawa wanaume, kumewahi kuwa na wale hujui hali yao ya VVU? | O Yes  O No  O Refused to answer |
| NPP10 | *Ask only if arm= “Intervention”*  Did you offer a self-test to **any** of your non-primary partners in the past 6 months?  Je ulipatiana kifaa cha kujipima kibinafsi kwa kwa mshiriki wako yeyote ambaye siye wa mkuu kwa miezi 6 zilizopita? | O Yes  O No  O Don’t know  O Refused to answer |
| NPP11 | *Ask only if arm= “Control”*  Did you offer a VCT referral card to **any** of your non-primary partners in the past 6 months?  Ulipatiana kadi ya mwaliko kwamshiriki wako yeyote asiye mkuu kwa miezi 6 zilizopita? | O Yes  O No  O Don’t know  O Refused to answer |
| NPP12 | In the past 6 months, did you find out directly or indirectly that any of your non-primary partners tested for HIV and obtained an HIV-positive result?  Kwa miezi 6 zilizopita,Je, uligundua moja kwa moja au kupitia njia ingine kama mpenzi wako mkuu ye-yote amepimwa VVU na kupatikana na virusi vya ukimwi?  *Interviewer*: give various examples of how this could happen | O Yes  O No  O Don’t know  O Refused to answer |
| NPP13 | How many non-primary partners of yours tested HIV-positive in the past 6 months.  Ni washiriki wangapi wasio wakuu wako waliopatikana na VVU miezi 6 zilizopita? | Number ___ ___  O Don’t know  O Refused to answer |

| **Section C6: Transactional Sex (TRX)**  Interviewer: I would now like to ask questions about any sexual partners you may have had who paid you for sex or gave you something in exchange for sex. These may be men with whom you had only one sexual encounter, or men with whom you had multiple sexual encounters. Some of these questions may sound similar to ones I just asked, but please think only about those encounters where you exchanged sex.  Interviewer: Kwa sasa ningependa kuuliza maswali kuhusu mshiriki wa ngono wowote ina uwezekano ulikuwa naye aliyekulipa kwa kushiriki ngono au alikupa chochote kwa kushiriki ngono. Wanaweza kuwa wanaume ambayo ulishiriki ngono mara moja tu au wanaume uliyeshiriki ngono mara kadhaa. Baadhi ya maswali haya yanaweza fanana nay ale niliyouliza hapo awali lakini fikiria juu ya wakati ulishiriki ngono kwa kitu fulani peke yake. | | |
| --- | --- | --- |
| TRX01 | In the past 6 months, have you exchanged sex for money, goods, food, housing or services?  Je kwa miezi 6 zilizopita, umebadilisha ngono kwa pesa, bidhaa, zawadi, chakula, makaazi, (malipo ya: stima,maji nyumba nkt) huduma au kushawishi? | O Yes  O No (Skip to TRX18)  O Refused to Answer (Skip to TRX18) |
| TRX02 | In the past month, with approximately how many men did you exchange sex?  Je, kwa miezi iliyopita, ni kadri wanaume wangapi ambao ulibadilisha nao ngono? | _________ (If 0, skip to TRX18)  O Don’t Know  O Refused to Answer |
| TRX03 | In the past month, have you exchanged sex for **money**?  Je, kwa miezi iliyopita, umebadilisha ngono kwa **pesa**? | O Yes  O No (skip to TRX07)  O Refused to Answer (skip to TRX07) |
| TRX04 | In a typical month, how much of your income comes from exchanging sex for money?  Je, kwa kawaida ni kiasi kipi cha mapato yako hutokana kwa kubadilisha ngono kwa pesa? | O None  O Less than half  O About half  O More than half, but not all  O All  O Don’t Know  O Refused to Answer |
| TRX05 | When you exchange sex for money, what is the average amount of money you charge per sexual encounter when a condom **is** used?  Je unapobadilisha ngono kwa pesa, ni takriban pesa ngapi unalipisha kwa kila wakati umeshiriki ngono ikiwa mpira wa kondomu **umetumika**? | KES _________  O I Never Use a Condom  O Don’t Know  O Refused to Answer |
| TRX06 | When you exchange sex for money, what is the average amount of money per sexual encounter when a condom **is** **not** used?  Je unapobadilisha ngono kwa pesa, ni takriban pesa ngapi unalipisha kwa kila wakati umeshiriki ngono ikiwa mpira wa kondomu **umetumika**? | KES _________  O I Always Use a Condom  O Don’t Know  O Refused to Answer |
| TRX07 | In the past month, have you exchanged sex for goods or services **other than** money?  Je katika mwezi mmoja uliyopita ushawahi badilisha ngono kwa bidhaa au huduma **mbali na pesa**? | O Yes  O No (skip to TRX10)  O Refused to Answer (skip to TRX10) |
| TRX09 | In the past month, what goods or services **other than** money have you exchanged for sex? Please tell me about all goods and services.  Je katika mwezi mmoja uliyopita ni bidhaaau huduma zipi ulibadilisha kwa ngono? Tafadhali nielezee kuhusi hizo bidhaa na huduma zote  *Choose all that apply.* | O Housing and/or utilities  O Food to eat  O Food to sell (example, fish)  O School fees  O To get a job, a work promotion, or to keep your job  O Other material goods (clothes, jewelry, makeup, electronics, etc.)  O Household items (soap, cleaning supplies, tools, etc.)  O Other (specify): __________  O Don’t Know  O Refused to Answer |
| TRX10 | ***Ask only if TRX03 and/or TRX07 = Yes***  In the past month, what is the approximate **total** *value* of the money, goods, or services you received in exchange for sex?  Je kwa mwezi mmoja uliyopita, ni takriban pesa ngapi kwa ujumla, bidhaa, au huduma uliopata ukibadilisha kwa ngono?  *Interviewer: Make sure participant understands to include how much the non-monetary goods/services are worth.* | KES _______  O Don’t Know  O Refused to Answer |
| TRX11 | In the past 6 **MONTHS**, have you received money, goods, or services in exchange for sex **WITHOUT** a condom?  Je kwa MIEZI 6 iliyopita, umepokea pesa, bidhaa or huduma uliopata ukibadilisha kwa ngono bila mpira wa kondomu kutumika? | O Yes  O No 🡪 Skip to TRX18  O Refused to Answer 🡪 Skip to TRX18 |
| TRX12 | Among the men with whom you had sex without a condom, did know the HIV status of **ALL** these men?  Kati ya wanaume ulioshiki nao kwa ngono bila mpira wa kondomu kutumika, je ulijua hali ya VVU kwa hao wanaume WOTE?  *Interviewer: Answer is “No” even if status of one partner was unknown.* | O Yes 🡪 Skip to TRX14  O No  O Refused to Answer 🡪 Skip to TRX14 |
| TRX13 | For how many men did you NOT know the HIV status?  Je, hukujua hali ya VVU kwa wanaume wangapi? | ___ ___ (number of men)  O Don’t know  O Refused to answer |
| TRX14 | How many of these men were known to be HIV-POSITVE?  Je, wangapi kwa hao wanaume walijulikana KUWA na VVU? | ___ ___ (number of men)  O Don’t know  O Refused to answer |
| TRX15 | ***Ask only if arm= “Intervention”***  In the past 6 months, have you **OFFERED** HIV self-tests to any of the men whom you exchanged sex for money, goods, or services?  Je, katika miezi 6 ziliyopita, umepeana vyombo vya Kipimo cha kibinafsivya VVU wanaume wo-wote uliobadilisha nao pesa, bidhaa ua huduma kwa ngono? | O Yes  O No  O Refused to Answer |
| TRX16 | ***Ask only if arm= “Control”***  In the past 6 months, have you **OFFERED** VCT referral cards to any of the men whom you exchanged sex for money, goods, or services?  Je, katika miezi 6 ziliyopita, umepeana kadi ya rufaa ya kituo cha upimaji wa VVU kwa hiari kwa wanaume wo-wote uliobadilisha nao pesa, bidhaa ua huduma kwa ngono? | O Yes  O No  O Refused to Answer |
| TRX17 | Did you come to know the HIV status of any of these men specifically using the Jikinge tools?  Je ulikuja kujua hali ya VVU ya ye yote kati ya hawa wanaume haswa ukitumia vyombo vya jikinge?  *(Interviewer: the HIV self-test kits or the VCT referral cards)*? | O Yes  O No  O Refused to Answer |
| TRX18 | Over the last 6 months, has the number of partners you have had sex with for money, goods, or services changed?  Je, katika miezi 6 ziliyopita, idadi ya wapenzi uliobadilisha nao pesa, bidhaa ua huduma kwa ngono imebadilika? | O No Change 🡪 Skip to Section D  O Increase 🡪 Do not ask TRX20  O Decrease 🡪 Skip to TRX20  O Don’t Know 🡪 Skip to Section D  O Refused to Answer 🡪 Skip to Section D |
| TRX19 | Why has the number of partners you had sex with in exchange for money, goods, or services increased?  Je, mbona idadi ya wapenzi uliobadilisha nao pesa, bidhaa ua huduma kwa ngono imeongezeka? | *Open Ended:* |
| TRX20 | Why has the number of partners you had sex with in exchange for money, goods, or services decreased? | *Open Ended:* |

| **Section D: Recent Transactional Encounters (RT#)** | | |
| --- | --- | --- |
| Interviewer: Now, I will be asking you a series of questions related to your recent sexual partners from whom you received money or something else in exchange for sex. I have asked some similar questions but now I will ask for some more details. I greatly appreciate your patience through the next series of questions. I want you to think about the most recent time you exchanged sex for money, goods, gift, food, or housing. I am going to ask you several questions about this exchange.  **Kwa sasa , nitakuuliza baadhi ya maswali inayo ambatana na ushiriki wako wa ngono ya hivi majuzi aliyekupatia pesa au kitu nyingine kwa kushiriki ngono. Nimeuliza maswali mengine sawa na hayo lakini kwa wakati huu nitauliza ufafanue zaidi. Nashukuru sana utulivu wako kupitia baadhi ya maswali yafuatayo. Ningependa ufikirie kuhusu ushiriki wako wa ngono ya hivi majuzi zaidi ulipobadilisha ngono na pesa, bidhaa, zawadi, chakula au nyumba. Niatkuuliza baadhi ya maswali kuhusu kubadilisha hiki** | | |
| **No.** | **Question** | **Coding** |
| RT1: Most Recent Exchange | | |
| RT1 Q01 | Would you like to tell me about the most recent time you exchanged sex in the past 6 months?  Je ungependa kunijulisha kuhusu wakati wa hivi karibuni ulipobadilisha ngono kwa miezi 6 zilizopita? | O Yes  O No (skip to RT2 Q01) |
| RT1 Q02 | When did this sex exchange for money, goods, or gifts occur?  Huku kubadilisha ngono kwa pesa, bidhaa au zawadi ilitendeka lini? | O I Know the Date  O Don’t Know (skip to RT1 Q04)  O Refused to Answer (skip to RT1 Q04) |
| RT1 Q03 | What was the date?  Ilikuwa tarehe gani? | ___ ___/___ ___ ___/ ___ ___ ___ ___  D D M M M Y Y Y Y |
| RT1 Q04 | Where did you exchange sex with this person?  Ni pahali gani ulibadilisha ngono na mtu huyu? | O Street, car, or outside  O Bar or nightclub  O Hotel room paid by sex worker  O Hotel room paid by partner  O Brothel  O Woman’s home  O Man’s home  O Other (specify): __________________________  O Don't Know  O Refused to Answer |
| RT1 Q05 | Please tell me which of the following activities you did during this encounter:  Tafadhali nielezee , ni vitendo vipi vifuatavyo ulivyotenda wakati wa tukio hiki:  *Interviewer: Read the list of responses aloud to the participant and choose all that apply. Explain any of the choices that the participant does not understand.* | O Kissing  O Danced or stripped for partner  O Massage  O Performed oral sex **with** a condom  O Performed oral sex **without** a condom  O Vaginal sex **with** a condom  O Vaginal sex **without** a condom  O Anal sex **with** a condom  O Anal sex **without** a condom  O Received oral sex  O Talking/company  O Other (specify) |
| RT1 Q06 | Was this the first time you have exchanged sex with this person?  Je hii ilikuwa mara yako ya kwanza kubadilisha ngono na mtu huyu? | O Yes  O No  O Don’t Know O Refused to Answer |
| RT1 Q07 | Approximately how old was this person? If not sure. please estimate.  Je, mtu huyu alikuwa takriban umri upi? Iwapo haun uhakika tafadhali kadiria. | ___ ___ years  O Don’t know  O Refused to answer |
| RT1 Q08 | Does this person live in this County, or was he visiting?  Je, mtu huyu anaishi katika eneo ya kaunti hii, au alikuja kwa matembezi? | O Lives in this County  O Just visiting  O Don’t know  O Refused to answer |
| RT1 Q09 | Did this person take alcohol or drugs around the time you had sex?  Je, mtu huyu alitumia pombe au miadarati kwa wakati mlishiriki ngono? | O Yes  O No  O Don’t know  O Refused to answer |
| RT1 Q10 | Did YOU take alcohol or drugs around the time you had sex with this person?  Je, ulikunywa pombe au kutumia miadarati wakati uliposhiriki ngono na mtu huyu? | O Yes  O No  O Don’t know  O Refused to answer |
| RT1 Q11 | What was the **total value** of money, goods and gifts that the person gave you for this encounter?  Ulipokea jumla ya pesa ngapi, bidhaa au zawadi ambayo mshiriki wako wa ngono alikupa katika tukio hiki? | KES_________  O Don't Know  O Refused to Answer |
| RT1 Q12 | Did you know the HIV status of this person around the time you had sex?  Je ulijua hali ya VVU ya huyu mtu wakati ulishiriki naye kwa ngono? | O Yes  O No  O Refused to answer |
| NEXT SET OF QUESTIONS ARE FOR PARTICIPANTS IN **INTERVENTION** GROUP | | |
| RT1 Q13 | Did you offer an HIV self-test to this person?  Je, ulipeana Kipimo cha kibinafsi ya VVU kwa huyu mtu | O Yes  O No 🡪 skip to RT1 Q28  O Don’t know 🡪 skip to RT1 Q28  O Refused to answer 🡪 skip to RT1 Q28 |
| RT1 Q14 | Did he accept the HIV self-test?  Je, alikubali Kipimo cha kibinafsi ya VVU? | O Yes  O No 🡪 skip to RT1 Q28  O Don’t know 🡪 skip to RT1 Q28  O Refused to answer 🡪 skip to RT1 Q28 |
| RT1 Q15 | Did he use the HIV self-test?  Je, alikutumia Kipimo cha kibinafsi ya VVU? | O Yes  O No 🡪 Skip to RT1 Q28  O Don’t know 🡪 skip to RT1 Q28  O Refused to answer 🡪 skip to RT1 Q28 |
| RT1 Q16 | When did he use the self-test?  Je, ni lini alikutumia Kipimo cha kibinafsi ya VVU? | O Less than 1 week ago  O Less than 1 month ago  O Less than 3 months ago  O Less than 6 months ago  O Don’t Know  O Refused to Answer |
| RT1 Q17 | Were you present when he used the self-test?  Je, uliwa wakati alikutumia Kipimo cha kibinafsi ya VVU? | O I was present when he used the self-test  O I was not present but he told me that he used the self-test kit 🡪 skip to RT1 Q19  O Don’t know  O Refused to answer |
| RT1 Q18 | At the **same** time that your partner used the self-test, did you **also** use a self-test?  Kwa wakati huo huyo mpenzi wako alitumia Kipimo cha kibinafsi ya VVU, je, wewe pia ulitumia mpimo-binafsi? | O Yes, we tested together at the same time  O Yes, we both tested but not together  O No  O Other (specify)  O Don’t know  O Refused to answer |
| RT1 Q19 | What was the result of his HIV test?  Je, nini ilikuwa matokeo ya kipimo cha VVU? | O Positive  O Negative  O Invalid / Indeterminate  O Don’t Know  O Refused |
| RT1 Q20 | Did he go to a clinic or health facility to confirm the result that was obtained from the self-test (confirmatory testing)?  Je, alienda kwa kliniki au kituo cha afya kuhakikisha matokeo aliopata kwa Kipimo cha kibinafsi(kipimo chauhakikisho)? | O Yes  O No  O Don’t know  O Refused to answer |
| RT1 Q21 | Did he go to a clinic or health facility for HIV care and treatment?  Je, alienda kwa kliniki au kituo cha afya kupata huduma ya matibabu ya VVU? | O Yes  O No  O Don’t know  O Refused to answer |
| NEXT SET OF QUESTIONS ARE FOR **CONTROL** GROUP ONLY | | |
| RT1 Q22 | Did you offer a VCT referral card to this person?  Je, ulipeana kadi ya rufaa ya kituo cha upimaji ya VVU kwa hiari kwa mtu huyu? | O Yes  O No 🡪 Skip to RT1 Q28  O Don’t know 🡪 Skip to RT1 Q28  O Refused to answer 🡪 Skip to RT1 Q28 |
| RT1 Q23 | Did he accept the VCT referral card?  Je, alikubali kadi ya rufaa ya kituo cha upimaji ya VVU kwa hiari? | O Yes  O No 🡪 Skip to RT1 Q28  O Don’t know 🡪 Skip to RT1 Q28  O Refused to answer 🡪 Skip to RT1 Q28 |
| RT1 Q24 | Do you know whether he went for an HIV test at a VCT clinic?  Je, unajua kama alienda kwa kliniki au kituo cha afya kupata huduma ya matibabu ya VVU? | O Yes  O No 🡪 Skip to RT1 Q28  O Don’t know 🡪 Skip to RT1 Q28  O Refused to answer 🡪 Skip to RT1 Q28 |
| RT1 Q25 | At the same time that your partner got tested for HIV, did you also test?  Kwa wakati huo huyo mpenzi wako alitumia Kipimo cha kibinafsi ya VVU, je, wewe pia ulitumia mpimo-binafsi? | O Yes, we tested together at the same time  O Yes, we both tested but not together  O No  O Other (specify)  O Don’t know  O Refused to answer |
| RT1 Q26 | What was the result of his HIV test?  Je, nini ilikuwa matokeo ya kipimo cha VVU? | O Positive  O Negative 🡪 skip to RT1 Q28  O Invalid / Indeterminate  O Don’t Know 🡪 skip to RT1 Q28  O Refused 🡪 skip to RT1 Q28 |
| RT1 Q27 | Did he go to a clinic or health facility for HIV care and treatment?  Je, alienda kwa kliniki au kituo cha afya kupata huduma ya matibabu ya VVU? | O Yes  O No  O Don’t know  O Refused to answer |
| Interviewer READ: I will now ask you some questions about things this same person may have done to you during this encounter. These questions are about violence, which may make you feel uncomfortable or distressed. Please remember that your answers are completely confidential and no one will be able to associate this information with you or your sexual partners. You do not have to answer any question that makes you uncomfortable. Please take your time and if you are unclear about any question, just ask me. Are you ready to continue?  Kwa sasa nitakuuliza baadhi ya maswali kuhusu vitu mtu huyu kuna uwezekano alikufanyia wakati wa ushiriki huu. Maswali haya ni juu ya vita, ambazo zinaweza kukufanya usihisi vizuri au kutaabika. Tafadhali kumbuka yakuwa jibu lako zimewekwa kwa hali ya siri na hakuna atakaye husisha ujumbe huu na wewe au mshiriki wako wa ngono. Siyo lazima ujibu swali lolote inayokufanya usihisi vizuri. Tafadhali chukua muda wako na usipoelewa swali lolote, niulize. Uko tayari tuendelee? | | |
| RT1 Q28 | Did this partner threaten you with physical assault?  Je, mpenzi huyu alikutishia kwa kukupiga | O Yes  O No  O Don’t know  O Refused to answer |
| RT1 Q29 | Did this partner hit, kick, strangle or otherwise physically assault you?  Je huyu mpenzi alikugonga, kukupiga teke, kukunyonga ama kukuumiza? | O Yes  O No  O Don’t know  O Refused to answer |
| RT1 Q30 | Did this partner force or coerce you to participate in any sex act against your will?  Je, huyu mpenzi alikulazimisha kushiriki katika tendo cha ngono hiari yako?  Choose all that apply | O Yes, to have sex with a condom  O Yes, to have sex without a condom  O Yes, to have anal sex  O Yes, Other____________________________  O No  O Don’t know  O Refused to answer |
| Interviewer: Now, I will be asking you the same questions as I just did, for your SECOND MOST RECENT encounter where you exchanged sex for something. This could be with a different partner as the one we just discussed, or the same.  *Interviewer*: Sasa, nitakuuliza maswali sawa na niliyokuuliza hapo awali, kwa ushiriki wako wako wa ngono ya karibu zaidi ya pili ulipobadilisha ngono na kitu. Hii inaweza kuwa na mshiriki tofauti kama taliyezungumzia au huyo. | | |
| RT2 Q01 | Would you like to tell me about the most recent time you exchanged sex in the past 6 months?  Je ungependa kunijulisha kuhusu wakati wa hivi karibuni ulipobadilisha ngono kwa miezi 6 zilizopita? | O Yes  O No (skip to RT3 Q01) |
| RT2 Q02 | When did this sex exchange for money, goods, or gifts occur?  Huku kubadilisha ngono kwa pesa, bidhaa au zawadi ilitendeka lini? | O I Know the Date  O Don’t Know (skip to RT2 Q04)  O Refused to Answer (skip to RT2 Q04) |
| RT2 Q03 | What was the date?  Ilikuwa tarehe gani? | ___ ___/___ ___ ___/ ___ ___ ___ ___  D D M M M Y Y Y Y |
| RT2 Q04 | Where did you exchange sex with this person?  Ni pahali gani ulibadilisha ngono na mtu huyu? | O Street, car, or outside  O Bar or nightclub  O Hotel room paid by sex worker  O Hotel room paid by partner  O Brothel  O Woman’s home  O Man’s home  O Other (specify): __________________________  O Don't Know  O Refused to Answer |
| RT2 Q05 | Please tell me which of the following activities you did during this encounter:  Tafadhali nielezee , ni vitendo vipi vifuatavyo ulivyotenda wakati wa tukio hiki:  *Interviewer: Read the list of responses aloud to the participant and choose all that apply. Explain any of the choices that the participant does not understand.* | O Kissing  O Danced or stripped for partner  O Massage  O Performed oral sex **with** a condom  O Performed oral sex **without** a condom  O Vaginal sex **with** a condom  O Vaginal sex **without** a condom  O Anal sex **with** a condom  O Anal sex **without** a condom  O Received oral sex  O Talking/company  O Other (specify): __________________  O Don't Know  O Refused to Answer |
| RT2 Q06 | Was this the first time you have exchanged sex with this person?  Je hii ilikuwa mara yako ya kwanza kubadilisha ngono na mtu huyu? | O Yes  O No  O Don’t Know O Refused to Answer |
| RT2 Q07 | Approximately how old was this person? If not sure. please estimate.  Je, mtu huyu alikuwa takriban umri upi? Iwapo haunq uhakika tafadhali kadiria. | ___ ___ years  O Don’t know  O Refused to answer |
| RT2 Q08 | Does this person live in this County, or was he visiting?  Je, mtu huyu anaishi katika eneo ya kaunti hii, au alikuja kwa matembezi? | O Lives in this County  O Just visiting  O Don’t know  O Refused to answer |
| RT2 Q09 | Did this person take alcohol or drugs around the time you had sex?  Je, mtu huyu alitumia pombe au miadarati kwa wakati mlishiriki ngono? | O Yes  O No  O Don’t know  O Refused to answer |
| RT2 Q10 | Did YOU take alcohol or drugs around the time you had sex with this person?  Je, ulikunywa pombe au kutumia miadarati wakati uliposhiriki ngono na mtu huyu? | O Yes  O No  O Don’t know  O Refused to answer |
| RT2 Q11 | What was the **total value** of money, goods and gifts that the person gave you for this encounter?  Ulipokea jumla ya pesa ngapi, bidhaa au zawadi ambayo mshiriki wako wa ngono alikupa katika tukio hiki? | KES_________  O Don't Know  O Refused to Answer |
| RT2 Q12 | Did you know the HIV status of this person around the time you had sex?  Je ulijua hali ya VVU ya huyu mtu wakati ulishiriki naye kwa ngono? | O Yes  O No  O Refused to answer |
| NEXT SET OF QUESTIONS ARE FOR PARTICIPANTS IN **INTERVENTION** GROUP | | |
| RT2 Q13 | Did you offer an HIV self-test to this person?  Je, ulipeana Kipimo cha kibinafsi ya VVU kwa huyu mtu | O Yes  O No 🡪 skip to RT2 Q28  O Don’t know 🡪 skip to RT2 Q28  O Refused to answer 🡪 skip to RT2 Q28 |
| RT2 Q14 | Did he accept the HIV self-test?  Je, alikubali Kipimo cha kibinafsi ya VVU? | O Yes  O No 🡪 skip to RT2 Q28  O Don’t know 🡪 skip to RT2 Q28  O Refused to answer 🡪 skip to RT2 Q28 |
| RT2 Q15 | Did he use the HIV self-test?  Je, alikutumia Kipimo cha kibinafsi ya VVU? | O Yes  O No 🡪 Skip to RT2 Q28  O Don’t know 🡪 skip to RT2 Q28  O Refused to answer 🡪 skip to RT2 Q28 |
| RT2 Q16 | When did he use the self-test?  Je, ni lini alikutumia Kipimo cha kibinafsi ya VVU? | O Less than 1 week ago  O Less than 1 month ago  O Less than 3 months ago  O Less than 6 months ago  O Don’t Know  O Refused to Answer |
| RT2 Q17 | Were you present when he used the self-test?  Je, uliwa wakati alikutumia Kipimo cha kibinafsi ya VVU? | O I was present when he used the self-test  O I was not present but he told me that he used the self-test kit 🡪 skip to RT2 Q19  O Don’t know  O Refused to answer |
| RT2 Q18 | At the **same** time that your partner used the self-test, did you **also** use a self-test?  Kwa wakati huo huyo mpenzi wako alitumia Kipimo cha kibinafsi ya VVU, je, wewe pia ulitumia mpimo-binafsi? | O Yes, we tested together at the same time  O Yes, we both tested but not together  O No  O Other (specify): _____________________  O Don’t know  O Refused to answer |
| RT2 Q19 | What was the result of his HIV test?  Je, nini ilikuwa matokeo ya kipimo cha VVU? | O Positive  O Negative  O Invalid / Indeterminate  O Don’t Know  O Refused |
| RT2 Q20 | Did he go to a clinic or health facility to confirm the result that was obtained from the self-test (confirmatory testing)?  Je, alienda kwa kliniki au kituo cha afya kuhakikisha matokeo aliopata kwa Kipimo cha kibinafsi(kipimo chauhakikisho)? | O Yes  O No  O Don’t know  O Refused to answer |
| RT2 Q21 | Did he go to a clinic or health facility for HIV care and treatment?  Je, alienda kwa kliniki au kituo cha afya kupata huduma ya matibabu ya VVU? | O Yes  O No  O Don’t know  O Refused to answer |
| NEXT SET OF QUESTIONS ARE FOR **CONTROL** GROUP ONLY | | |
| RT2 Q22 | Did you offer a VCT referral card to this person?  Je, ulipeana kadi ya rufaa ya kituo cha upimaji ya VVU kwa hiari kwa mtu huyu? | O Yes  O No 🡪 Skip to RT2 Q28  O Don’t know 🡪 Skip to RT2 Q28  O Refused to answer 🡪 Skip to RT2 Q28 |
| RT2 Q23 | Did he accept the VCT referral card?  Je, alikubali kadi ya rufaa ya kituo cha upimaji ya VVU kwa hiari? | O Yes  O No 🡪 Skip to RT2 Q28  O Don’t know 🡪 Skip to RT2 Q28  O Refused to answer 🡪 Skip to RT2 Q28 |
| RT2 Q24 | Do you know whether he went for an HIV test at a VCT clinic?  Je, unajua kama alienda kwa kliniki au kituo cha afya kupata huduma ya matibabu ya VVU? | O Yes  O No 🡪 Skip to RT2 Q28  O Don’t know 🡪 Skip to RT2 Q28  O Refused to answer 🡪 Skip to RT2 Q28 |
| RT2 Q25 | At the **same** time that your partner got tested for HIV, did you also test?  Kwa wakati huo huyo mpenzi wako alitumia Kipimo cha kibinafsi ya VVU, je, wewe pia ulitumia mpimo-binafsi? | O Yes, we tested together at the same time  O Yes, we both tested but not together  O No  O Other (specify)  O Don’t know  O Refused to answer |
| RT2 Q26 | What was the result of his HIV test?  Je, nini ilikuwa matokeo ya kipimo cha VVU? | O Positive  O Negative 🡪 skip to RT2 Q28  O Invalid / Indeterminate  O Don’t Know 🡪 skip to RT2 Q28  O Refused 🡪 skip to RT2 Q28 |
| RT2 Q27 | Did he go to a clinic or health facility for HIV care and treatment?  Je, alienda kwa kliniki au kituo cha afya kupata huduma ya matibabu ya VVU? | O Yes  O No  O Don’t know  O Refused to answer |
| Interviewer READ: I will now ask you some questions about things this same person may have done to you during this encounter. These questions are about violence, which may make you feel uncomfortable or distressed. Please remember that your answers are completely confidential and no one will be able to associate this information with you or your sexual partners. You do not have to answer any question that makes you uncomfortable. Please take your time and if you are unclear about any question, just ask me. Are you ready to continue?  Kwa sasa nitakuuliza baadhi ya maswali kuhusu vitu mtu huyu kuna uwezekano alikufanyia wakati wa ushiriki huu. Maswali haya ni juu ya vita, ambazo zinaweza kukufanya usihisi vizuri au kutaabika. Tafadhali kumbuka yakuwa jibu lako zimewekwa kwa hali ya siri na hakuna atakaye husisha ujumbe huu na wewe au mshiriki wako wa ngono. Siyo lazima ujibu swali lolote inayokufanya usihisi vizuri. Tafadhali chukua muda wako na usipoelewa swali lolote, niulize. Uko tayari tuendelee? | | |
| RT2 Q28 | Did this partner threaten you with physical assault?  Je, mpenzi huyu alikutishia kwa kukupiga | O Yes  O No  O Don’t know  O Refused to answer |
| RT2 Q29 | Did this partner hit, kick, strangle or otherwise physically assault you?  Je huyu mpenzi alikugonga, kukupiga teke, kukunyonga ama kukuumiza? | O Yes  O No  O Don’t know  O Refused to answer |
| RT2 Q30 | Did this partner force or coerce you to participate in any sex act against your will?  Je, huyu mpenzi alikulazimisha kushiriki katika tendo cha ngono hiari yako?  Choose all that apply | O Yes, to have sex with a condom  O Yes, to have sex without a condom  O Yes, to have anal sex  O Yes, Other____________________________  O No  O Don’t know  O Refused to answer |
| Interviewer: For the last time, I will be asking you the same questions as I just did about your THIRD MOST RECENT encounter where you exchanged sex for something. This could be with a different partner as the one we just discussed, or the same.  Je, ungependa kunielezea kuhusu mara ya tatu ya hivi karibuni ulpobadilisha ngono? | | |
| RT3 Q01 | Would you like to tell me about the most recent time you exchanged sex in the past 6 months?  Je ungependa kunijulisha kuhusu wakati wa hivi karibuni ulipobadilisha ngono kwa miezi 6 zilizopita? | O Yes  O No (skip to HIV01) |
| RT3 Q02 | When did this sex exchange for money, goods, or gifts occur?  Huku kubadilisha ngono kwa pesa, bidhaa au zawadi ilitendeka lini? | O I Know the Date  O Don’t Know (skip to RT3 Q04)  O Refused to Answer (skip to RT3 Q04) |
| RT3 Q03 | What was the date?  Ilikuwa tarehe gani? | ___ ___/___ ___ ___/ ___ ___ ___ ___  D D M M M Y Y Y Y |
| RT3 Q04 | Where did you exchange sex with this person?  Ni pahali gani ulibadilisha ngono na mtu huyu? | O Street, car, or outside  O Bar or nightclub  O Hotel room paid by sex worker  O Hotel room paid by partner  O Brothel  O Woman’s home  O Man’s home  O Other (specify): __________________________  O Don't Know  O Refused to Answer |
| RT3 Q05 | Please tell me which of the following activities you did during this encounter:  Tafadhali nielezee , ni vitendo vipi vifuatavyo ulivyotenda wakati wa tukio hiki:  *Interviewer: Read the list of responses aloud to the participant and choose all that apply. Explain any of the choices that the participant does not understand.* | O Kissing  O Danced or stripped for partner  O Massage  O Performed oral sex **with** a condom  O Performed oral sex **without** a condom  O Vaginal sex **with** a condom  O Vaginal sex **without** a condom  O Anal sex **with** a condom  O Anal sex **without** a condom  O Received oral sex  O Talking/company  O Other (specify): __________________  O Don't Know  O Refused to Answer |
| RT3 Q06 | Was this the first time you have exchanged sex with this person?  Je hii ilikuwa mara yako ya kwanza kubadilisha ngono na mtu huyu? | O Yes  O No  O Don’t Know O Refused to Answer |
| RT3 Q07 | Approximately how old was this person? If not sure. please estimate.  Je, mtu huyu alikuwa takriban umri upi? Iwapo haun uhakika tafadhali kadiria. | ___ ___ years  O Don’t know  O Refused to answer |
| RT3 Q08 | Does this person live in this County, or was he visiting?  Je, mtu huyu anaishi katika eneo ya kaunti hii, au alikuja kwa matembezi? | O Lives in this County  O Just visiting  O Don’t know  O Refused to answer |
| RT3 Q09 | Did this person take alcohol or drugs around the time you had sex?  Je, mtu huyu alitumia pombe au miadarati kwa wakati mlishiriki ngono? | O Yes  O No  O Don’t know  O Refused to answer |
| RT3 Q10 | Did YOU take alcohol or drugs around the time you had sex with this person?  Je, ulikunywa pombe au kutumia miadarati wakati uliposhiriki ngono na mtu huyu? | O Yes  O No  O Don’t know  O Refused to answer |
| RT3 Q11 | What was the **total value** of money, goods and gifts that the person gave you for this encounter?  Ulipokea jumla ya pesa ngapi, bidhaa au zawadi ambayo mshiriki wako wa ngono alikupa katika tukio hiki? | KES_________  O Don't Know  O Refused to Answer |
| RT3 Q12 | Did you know the HIV status of this person around the time you had sex?  Je ulijua hali ya VVU ya huyu mtu wakati ulishiriki naye kwa ngono? | O Yes  O No  O Refused to answer |
| NEXT SET OF QUESTIONS ARE FOR PARTICIPANTS IN **INTERVENTION** GROUP | | |
| RT3 Q13 | Did you offer an HIV self-test to this person?  Je, ulipeana Kipimo cha kibinafsi ya VVU kwa huyu mtu | O Yes  O No 🡪 skip to RT3 Q28  O Don’t know 🡪 skip to RT3 Q28  O Refused to answer 🡪 skip to RT3 Q28 |
| RT3 Q14 | Did he accept the HIV self-test?  Je, alikubali Kipimo cha kibinafsi ya VVU? | O Yes  O No 🡪 skip to RT3 Q28  O Don’t know 🡪 skip to RT3 Q28  O Refused to answer 🡪 skip to RT3 Q28 |
| RT3 Q15 | Did he use the HIV self-test?  Je, alikutumia Kipimo cha kibinafsi ya VVU? | O Yes  O No 🡪 Skip to RT3 Q28  O Don’t know 🡪 skip to RT3 Q28  O Refused to answer 🡪 skip to RT3 Q28 |
| RT3 Q16 | When did he use the self-test?  Je, ni lini alikutumia Kipimo cha kibinafsi ya VVU? | O Less than 1 week ago  O Less than 1 month ago  O Less than 3 months ago  O Less than 6 months ago  O Don’t Know  O Refused to Answer |
| RT3 Q17 | Were you present when he used the self-test?  Je, uliwa wakati alikutumia Kipimo cha kibinafsi ya VVU? | O I was present when he used the self-test  O I was not present but he told me that he used the self-test kit 🡪 skip to RT3 Q19  O Don’t know  O Refused to answer |
| RT3 Q18 | At the **same** time that your partner used the self-test, did you **also** use a self-test?  Kwa wakati huo huyo mpenzi wako alitumia Kipimo cha kibinafsi ya VVU, je, wewe pia ulitumia mpimo-binafsi? | O Yes, we tested together at the same time  O Yes, we both tested but not together  O No  O Other (specify): _____________________  O Don’t know  O Refused to answer |
| RT3 Q19 | What was the result of his HIV test?  Je, nini ilikuwa matokeo ya kipimo cha VVU? | O Positive  O Negative  O Invalid / Indeterminate  O Don’t Know  O Refused |
| RT3 Q20 | Did he go to a clinic or health facility to confirm the result that was obtained from the self-test (confirmatory testing)?  Je, alienda kwa kliniki au kituo cha afya kuhakikisha matokeo aliopata kwa Kipimo cha kibinafsi(kipimo chauhakikisho)? | O Yes  O No  O Don’t know  O Refused to answer |
| RT3 Q21 | Did he go to a clinic or health facility for HIV care and treatment?  Je, alienda kwa kliniki au kituo cha afya kupata huduma ya matibabu ya VVU? | O Yes  O No  O Don’t know  O Refused to answer |
| NEXT SET OF QUESTIONS ARE FOR **CONTROL** GROUP ONLY | | |
| RT3 Q22 | Did you offer a VCT referral card to this person?  Je, ulipeana kadi ya rufaa ya kituo cha upimaji ya VVU kwa hiari kwa mtu huyu? | O Yes  O No 🡪 Skip to RT3 Q28  O Don’t know 🡪 Skip to RT3 Q28  O Refused to answer 🡪 Skip to RT3 Q28 |
| RT3 Q23 | Did he accept the VCT referral card?  Je, alikubali kadi ya rufaa ya kituo cha upimaji ya VVU kwa hiari? | O Yes  O No 🡪 Skip to RT3 Q28  O Don’t know 🡪 Skip to RT3 Q28  O Refused to answer 🡪 Skip to RT3 Q28 |
| RT3 Q24 | Do you know whether he went for an HIV test at a VCT clinic?  Je, unajua kama alienda kwa kliniki au kituo cha afya kupata huduma ya matibabu ya VVU? | O Yes  O No 🡪 Skip to RT3 Q28  O Don’t know 🡪 Skip to RT3 Q28  O Refused to answer 🡪 Skip to RT3 Q28 |
| RT3 Q25 | At the **same** time that your partner got tested for HIV, did you also test?  Kwa wakati huo huyo mpenzi wako alitumia Kipimo cha kibinafsi ya VVU, je, wewe pia ulitumia mpimo-binafsi? | O Yes, we tested together at the same time  O Yes, we both tested but not together  O No  O Other (specify)  O Don’t know  O Refused to answer |
| RT3 Q26 | What was the result of his HIV test?  Je, nini ilikuwa matokeo ya kipimo cha VVU? | O Positive  O Negative 🡪 skip to RT3 Q28  O Invalid / Indeterminate  O Don’t Know 🡪 skip to RT3 Q28  O Refused 🡪 skip to RT3 Q28 |
| RT3 Q27 | Did he go to a clinic or health facility for HIV care and treatment?  Je, alienda kwa kliniki au kituo cha afya kupata huduma ya matibabu ya VVU? | O Yes  O No  O Don’t know  O Refused to answer |
| Interviewer READ: I will now ask you some questions about things this same person may have done to you during this encounter. These questions are about violence, which may make you feel uncomfortable or distressed. Please remember that your answers are completely confidential and no one will be able to associate this information with you or your sexual partners. You do not have to answer any question that makes you uncomfortable. Please take your time and if you are unclear about any question, just ask me. Are you ready to continue?  Kwa sasa nitakuuliza baadhi ya maswali kuhusu vitu mtu huyu kuna uwezekano alikufanyia wakati wa ushiriki huu. Maswali haya ni juu ya vita, ambazo zinaweza kukufanya usihisi vizuri au kutaabika. Tafadhali kumbuka yakuwa jibu lako zimewekwa kwa hali ya siri na hakuna atakaye husisha ujumbe huu na wewe au mshiriki wako wa ngono. Siyo lazima ujibu swali lolote inayokufanya usihisi vizuri. Tafadhali chukua muda wako na usipoelewa swali lolote, niulize. Uko tayari tuendelee? | | |
| RT3 Q28 | Did this partner threaten you with physical assault?  Je, mpenzi huyu alikutishia kwa kukupiga | O Yes  O No  O Don’t know  O Refused to answer |
| RT3 Q29 | Did this partner hit, kick, strangle or otherwise physically assault you?  Je huyu mpenzi alikugonga, kukupiga teke, kukunyonga ama kukuumiza? | O Yes  O No  O Don’t know  O Refused to answer |
| RT3 Q30 | Did this partner force or coerce you to participate in any sex act against your will?  Je, huyu mpenzi alikulazimisha kushiriki katika tendo cha ngono hiari yako?  *Choose all that apply* | O Yes, to have sex with a condom  O Yes, to have sex without a condom  O Yes, to have anal sex  O Yes, Other____________________________  O No  O Don’t know  O Refused to answer |

| **Section E: HIV and HIV Testing** | | |
| --- | --- | --- |
| **Section E1: General HIV Knowledge (HIV)**  Interviewer: I would now like to ask you some questions about what you already know and feel about HIV  Ningependa kukuuliza baadhi ya maswali kuhusu kile tayari unafahamu na kuhisi juu ya Ukimwi. | | |
| HIV01 | Are you currently taking any HIV medication in order to **prevent** acquiring HIV (PrEP)? This is usually a pill taken daily.  Je kwa sasa unameza dawa zozote za kuzuia maambukizi ya VVU (PrEP)? Hii kwa kawaida ni tembe inayomezwa kila siku.  *Interviewer: Ensure participant understands the principles of PrEP before proceeding. This is specifically asking about PrEP medication, not traditional medicines taken to prevent HIV.* | O Yes  O No  O Don’t Know  O Refused to Answer |
| HIV02 | What do you think your chances are of acquiring HIV in the future?  Je, unafikiri nafasi yako ya kuambukizwa virusi vya VVU ni ipi katika siku zijazo? | O None (Ask HIV03, not HIV04)  O Low (Ask HIV03, not HIV04)  O Moderate (skip to HIV04)  O High (skip to HIV04)  O Don’t Know (skip to HIV05)  O Refused to Answer (skip to HIV05) |
| HIV03 | ***Only ask if HIV02 = None or Low***  Why do you think you have a low chance or no chance of acquiring HIV in the future?  Je, ni kwa nini unafikiri kuwa una nafasi ya chini au hauna nafasi ya kuambukizwa VVU?  *Choose all that apply based on what participant mentioned.* | O Is Not Having Sex  O Uses Condoms  O Has Only One Partner  O Limits the Number of Partners  O Partner Has No Other Partners  O Knows Partner(s)’ HIV Status is Negative  O Trusts partner  O My Current Status is Negative  O Other _________________________________  O Don’t Know  O Refused to Answer |
| HIV04 | ***Only ask if HIV02 = Moderate or High***  Why do you think you have a moderate or high chance of acquiring HIV in the future?  Je, ni kwa nini unafikiri kuwa nafasi yako ya kuambukizwa VVU ni ya kadri au ya juu?  *Choose all that apply based on what the participant mentioned.* | O Does Not Use Condoms Regularly or at all  O Woman Has More Than One Partner  O Has Transactional Sex  O Does Not Trust Partner  O Partner is HIV positive  O She or Partner Refuses to be Tested  O Uses Injection Drugs/ Needles  O Primary Partner has more than one partner  O Non-primary partner(s) have more than one partner  O Other _________________________________  O Don’t Know  O Refused to Answer |
| HIV05 | How often do you believe you should test for HIV? Feel free to give more than one answer.  Je unaamini unafaa kupimwa vvu mara kwa mara mara ngapi? Jisikie huru kupeana majibu zaidi ya moja  *Interviewer - Choose all that apply. Responses can be frequency or situational.* | O Every week  O Every month  O Every 3 months  O Every 6 months  O Every year  O When I have sex without a condom  O When I have a new partner  O When my primary partner informs me or I know that he was unfaithful  O During pregnancy  O Other: ________________________________  O If I have been tested once, I do not need to be tested again  O Don’t Know  O Refused to Answer |
| **Section E2: Sexual Testing History (TST)**  Interviewer: I would now like to ask you some questions about testing for HIV and other sexually transmitted infections (STI) such as syphilis, gonorrhea, chlamydia, *Trichomonas vaginalis*, or bacterial vaginosis.  Ningependa kukuuliza baadhi ya maswali kuhusu upimaji wa virusi vya ukimwi na magonjwa mengine ya kisonono (STI) kama syphilis, gonorrhea, Chlamydia, Trichomonas vaginalis au bacterial vaginosis | | |
| TST0A | Before the test today, when did you last get tested for HIV?  Kabla ya kupimwa leo mara ya mwisho ulipimwa VVU?  *Interviewer – remind participant that using a self-test is also an example of getting tested.* | O Less than 1 week ago  O Between 1 week and 1 month ago  O Between 1 and 3 months ago  O Between 3 and 6 months ago  O Don’t Know  O Refused to Answer |
| TST02 | In the past 6 months, i.e. since your first appointment with Jikinge, have you been diagnosed with a sexually transmitted infection (STI)?  Kwa miezi 6 iliyopita, yaani tangu uanze kuja kutembelea Jikinge, umewahi patikana na magonjwa yoyote ya zinaa? | O Yes  O No (skip to GBV01)  O Don’t Know (skip to GBV01)  O Refused to answer (skip to GBV01) |
| TST03 | Which STI(s) were you diagnosed with?  Ni ugonjwa/ magonjwa ipi/zipi ya zinaaulipatikana nayo?  *Choose all that apply.*  *Interviewer: This is self-reported by the participant. If she does not remember the diagnosis, use “Don’t Know”. Do not try to diagnose her using symptoms she describes.* | O Trichomoniasis (Trich)  O Syphilis  O Gonorrhea  O Chlamydia  O Herpes  O Human papillomavirus (HPV)  O Genital warts  O Mycoplasma genitalium  O Bacterial vaginosis (BV)  O Other _______________________________  O Don’t Know  O Refused to answer |
| TST04 | Did you consult a doctor, pharmacist or other qualified healthcare provider in order to obtain treatment for this STI?  Je, ulimuona daktari, pharmacist au muudumu yeyote kwa matibabu ugonjwa wa zinaa?  *Interviewer: This includes traditional healers.* | O Yes  O No  O Don’t Know  O Refused to answer |

| **Section F: Gender-Based Violence (GBV)** | | |
| --- | --- | --- |
| *Interviewer*: The next questions are about things that happen within some relationships, and that your primary partner, or any other partners may have done to you. These questions are about violence, which may make you feel uncomfortable or distressed. You do not have to answer any question that makes you feel uncomfortable, and your responses are confidential. If you have any questions at any time, please ask me. Are you ready to continue?  Maswali yafuatatyo ni kuhusu vitu zinazofanyika ndani ya baadhi ya uhusiano na pia mshiriki wako mkuu au mshiriki yeyote anaweza kuwa alikufanyia. Maswali haya ni kuhusu vita amabazo zinaweza kukufanya usihisi vizuri au kutaabika. Siyo lazima kujibu maswali yoyote inayokufanya usihisi vizuri na majibu yako, yako kwa hali ya siri. Ukiwa na swali yoyote kwa wakati wowote tafadhali niulize. Uko tayari tuendelee? | | |
| **Section F1. PRIMARY PARTNERS** | | |
| GBV01:  Since you joined the Jikinge study [ENROLLMENT DATE] has your **PRIMARY** sexual partner done any of the following to you?  Je, tangu ujiunge na utafiti wa Jikinge [ENROLLMENT DATE] ni mara ngapi **mpenzi wako mkuu wa ngono** amekufanyia haya?  1 No 🡪 skip to next item  2 Yes 🡪 ask GBV02 and GBV03  99 Refused to answer 🡪 skip to next item  *Interviewer: Read each option aloud one at a time and choose all that apply.* | GBV02:  In the past 6 months, has your **PRIMARY** partner done this often or only sometimes?  Kwa muda wa miezi 6 iliyopita, je mshiriki wako **WA KARIBU** amekufanyia haya kila mara au mara zingine tu?  1 Often  2 Sometimes  98 Don’t Know  99 Refused to Answer | GBV03:  Did you ever tell anyone that your **PRIMARY** partner did this, and if so who did you tell?  Je, ulielezea mtu y kuwa mshiriki wako **WA KARIBU** alikufanyia haya na ikiwa ulieleza ni nani ulielezea?  1 Yes: _____________  2 No  98 Don’t Know  99 Refused to Answer |
| 1 Insulted or made you feel bad about yourself  Alikutusi au kukufanya hujihisi vibaya ____________ |  |  |
| 2 Belittled or humiliated you in front of other people  Kufanywa usiwe kama binadamu na kukuaibisha mbele ya watu ____________ |  |  |
| 3 Done anything to scare or intimidate you on purpose (by the way he looked at you, by yelling, smashing things, etc.)  Kufanya kitu ya kukushtua kwa kupenda kwake (vile alikutazama, kukupigia kelele, kuvunja vitu na kadhalalika) __________ |  |  |
| 4 Threatened to hurt you or someone you care about  Kutishia kukuumiza au mtu mwengine unayemjali _________ |  |  |
| 5 Slapped, hit, or thrown something at you that could hurt you  Kuzabwa kofi, au kukutupia kitu yenye inaweza kukuumiza _________ |  |  |
| 6 Pushed or shoved you  Kukuskuma au kukububurusha ____________ |  |  |
| 7 Kicked, dragged, or beaten you  Kukupiga teke, kukuvuta au kukupiga. ___________ |  |  |
| 8 Strangled or burnt you on purpose  kukunyonga au kukuchoma kwa kwakusudia ___________ |  |  |
| 9 Threatened or has actually used a gun, knife, or other weapon that could hurt you  Kukutishia ama ashatumia bastola, kwa hakika, kisu ama kifaa chochote yenye yaweza kukuumiza. __________ |  |  |
| 10 Fondled, groped, grabbed, or touched you in a way that was unwanted or made you feel unsafe  Kukushika shika kwa njia isiyofaa ama kukugusa kwa njia ambayo ilikufanya ujisikie hauko salama _________ |  |  |
| 11 Forced you to have sex when you did not want to or could not provide consent (ex. you were too drunk or passed out)  Alikulazimisha kufanya ngono kwa nguvu wakati hakutaka au bila idhini (kwa mfano ulikuwa mlevi au ulikuwa mlevi wa kupindukia) ______________ |  |  |
| **Section F2. NON-PRIMARY PARTNERS** | | |
| GBV04:  In the past 6 months has a **NON-PRIMARY** sexual partner done any of the following to you?  Je, kwa miezi 6 iliyopita mshiriki wako asiye mkuu amewahi kukufanyia vitu zifuatavyo?  1 No 🡪 skip to next item  2 Yes 🡪 ask GBV02 and GBV03  99 Refused to answer 🡪 skip to next item  *Interviewer: Read each option aloud one at a time and choose all that apply.*  *Interviewer: Read each option aloud one at a time and choose all that apply.* | GBV05:  In the past 6 months, has your **NON-PRIMARY** partner done this often or only sometimes?  Kwa muda wa miezi 6 iliyopita,je  mshiriki wako ASIYE WA KARIBU amekufanyia haya kila mara au mara zingine tu?  1 Often  2 Sometimes  98 Don’t Know  99 Refused to Answer | GBV06:  Did you ever tell anyone that your **NON-PRIMARY** partner did this, and if so who did you tell?  Je, ulielezea mtu y kuwa mshiriki wako ASIYE WA KARIBU alikufanyia haya na ikiwa ulieleza ni nani ulielezea?  1 Yes: _____________  2 No  98 Don’t Know  99 Refused to Answer |
| 1 Insulted or made you feel bad about yourself  Alikutusi au kukufanya hujihisi vibaya. _________ |  |  |
| 2 Belittled or humiliated you in front of other people  Kufanywa usiwe kama binadamu na kukuaibisha mbele ya watu. ____________ |  |  |
| 3 Done anything to scare or intimidate you on purpose (by the way he looked at you, by yelling, smashing things, etc.)  Kufanya kitu ya kukushtua kwa kusudia ( vile alivyo kutizama, kukupigia kelele , kuvunja vitu na kadhalalika) __________ |  |  |
| 4 Threatened to hurt you or someone you care about  Kutishia kukuumiza au mtu mwengine unaye mjali. ________ |  |  |
| 5 Slapped, hit, or thrown something at you that could hurt you  Kukuzaba kofi, au kukutupia kitu kinachoweza kukuumiza. __________ |  |  |
| 6 Pushed or shoved you  Kukuskuma au kukububurusha. ________ |  |  |
| 7 Kicked, dragged, or beaten you  Kukupiga teke, kukuvuta au kukupiga ________ |  |  |
| 8 Strangled or burnt you on purpose  kukunyonga au kukuchoma kwa kwakusudia. ________ |  |  |
| 9 Threatened or has actually used a gun, knife, or other weapon that could hurt you  Kukutishia ama ashatumia bastola, kwa hakika , kisu ama kifaa chochote yenye yaweza kukuumiza. _________ |  |  |
| 10 Fondled, groped, grabbed, or touched you in a way that was unwanted or made you feel unsafe  Kukushika shika kwa njia isiyofaa ama kukugusa kwa njia ambayo ilikufanya ujisikie hauko salama _________ |  |  |
| 11 Forced you to have sex when you did not want to or could not provide consent (ex. you were too drunk or passed out)  Alikulazimisha kufanya ngono kwa nguvu wakati hakutaka au bila idhini ( kwa mfano ulikuwa mlevi au ulikuwa mlevi wa kupindukia). ____________ |  |  |

| *The next questions should be asked if participants responded “yes” to* ***ANY*** *of the items above in Sections F1 or F2.* | | |
| --- | --- | --- |
| GBV07 | Do you think any of these things were the result of participating in Jikinge study?  Je unafikiri cho chote kwa vitu hivi vilisababishwa na kushiriki kwako kwa utafiti wa Jikinge?  *Ask all participants* | O Yes  O No  O Don’t Know  O Refused to answer |
| GBV08 | ***Ask only if arm= “Intervention”***  Do you think any of these things were the result of discussing HIV testing or offering an HIV self-test to your partner?  Je, unafikiri cho chote kwa vitu hivi vilisababishwa na kuzungumzia kupimwa kwa VVU au kupeana huduma ya Kipimo cha kibinafsiwa VVU? | O Yes  O No  O Don’t Know  O Refused to answer |
| GBV09 | ***Ask only if arm= “Control”***  Do you think any of these things were the result of discussing HIV testing or offering a VCT referral card to your partner?  Je, unafikiri cho chote kwa vitu hivi vilisababishwa na kuzungumzia kupimwa kwa VVU au kupeana kadi ya rufaa ya kituo cha upimaji wa VVU kwa hiari kwa mpenzi wako | O Yes  O No  O Don’t Know  O Refused to answer |

| **Section G1: HIV self-testing (Intervention Group Only)** | | |
| --- | --- | --- |
| *Read to participants in intervention group:* “I would like to ask you about your experience with the HIV self-tests that you received as part of the Jikinge study in the past 6 months.”  Ningependa kukuuliza juu ya uzoefu wako na vifaa vya kibinafsi vya upimaji VVU ulizozipokea kama sehemu utafiti wa Jikinge kwa miezi 6 zilizopita.” | | |
| **NO.** | **QUESTIONS** | **CODING CATEGORIES** |
| HST01 | Please tell me how many self-tests you have received **in the past 6 months**?  Tafadhali niambie ni vifaa vingapi vya upimaji umepokea kwa **miezi 6 iliyopita?**  *Interviewer*: *If the woman says, “Don’t know” please probe to see if she remembers receiving the tests, and if so, if she can make a guess about how many she received.* | ___ ___  O Don’t know/recall  O Refused to answer |
| HST02 | Please tell me how many self-tests you have received **in the past month**?  Tafadhali niambie ni vifaa vingapi vya kujipima umezipokea **kwa mwezi uliopita?**  *Interviewer*: *If the woman says, “Don’t know” please probe to see if she remembers receiving the tests, and if so, if she can make a guess about how many she received.* | ___ ___  O Don’t know/recall  O Refused to answer |
| HST03 | In the past 6 months, how many of HIV self-tests have you used to test yourself?  Kwa miezi 6 zilizopita, ni vifaa vingapi vya kujipima kibinafsi umetumia kujipima kibinafsi? | ___ ___  O Don’t know  O Refused to answer |
| HST04 | In the past 6 months, have you given any HIV self-tests to somebody else, such as your husband, sexual partners, friends, or family members?  Kwa miezi 6 zilizopita, umewahi patiana kifaa chochote cha kujipima kibinafsi kwa mtu mwingine , kama vile mumeo, washiriki wa ngono, marafiki, , au watu wa familia?  *Interviewer*: *Probe and include friends, co-workers etc. Make sure to ask participant to remember* ***all*** *self-tests that she gave to somebody else (even if that person did not end up using it)* | O YES  O NO 🡪Skip to HST11  O Don’t know  O Refused to answer |
| HST05 | In the past 6 months, about how many self-tests did you give to sexual partners?  Kwa miezi 6 zilizopita, ni kama vifaa vingapi vya upimaji wa kibinafsi ulizopatiana kwa washiriki wa ngono?    *Interviewer: Remind participant that if she gave 2 tests to the same partner, that should count as 2 tests given.* | ___ ___ (if 00, skip to HST08)  O Don’t know  O Refused to answer |
| HST06 | In the past 6 months, please tell me all the types of sexual partners whom you gave self-tests to.  Kwa miezi 6 zilizopita, tafadhali nieleze aina zote za washiriki wa ngono wale uliowapatia vifaa vya kujipima kibinafsi.  *Interviewer – select all that apply* | O Primary sexual partner  O Sexual partner who is not your primary sexual partner  O Commercial sex partner (client/ transactional sex partner)  O Don’t know  O Refused to answer |
| HST07 | Of all the sexual partners whom you gave self-tests to in the past 6 months, how many received an HIV-positive result (either directly observed by you or otherwise)?  Kwa washiriki wote wale uliowapatia vifaa vya kujipima kibinafsi kwa miezi 6 zilizopita, ni wangapi walipata matokeo ya kuwa na VVU (kwa njia ya moja kwa moja kama unaona au kwa vyengine vile)? | ___ ___  O Don’t know  O Refused to answer |
| HST08 | In the past 6 months, about how many self-tests did you give to other individuals who are not your sexual partners?  Kwa miezi 6 zilizopita, ni kama vifaa vingapi vya upimaji wa kibinafsi vya virusi vya Ukimwi ulipatiana kwa watu wengine ambao si washiriki wako wa ngono? | ___ ___ (if 00, skip to HST11)  O Don’t know  O Refused to answer |
| HST09 | In the past 6 months, please tell me all the types of other individuals who you gave self-tests to.  Kwa miezi 6 zilizopita, tafadhali niambie aina zote za washiriki wengine wa ngono wale uliwapa vifaa vya kujipima kibinafsi.  *Interviewer – select all that apply* | O A male adult family member  O A female adult family member  O A child in your family  O A male friend  O A female friend  O Coworker or Peer  O Don’t know  O Refused to answer |
| HST10 | Of all the other individuals whom you gave self-tests to in the past 6 months, how many received an HIV-positive result (either directly observed by you or otherwise)?  Kwa watu wote wale uliowapatia vifaa vya kujipima kibinafsi kwa miezi 6 zilizopita, ni wangapi walipokea matokeo ya kuwa na VVU (kwa njia ya moja kwa moja kama unaona au kwa vyengine vile)? | ___ ___  O Don’t know  O Refused to answer |
| **Relationship and sexual behavior change** | | |
| HST11 | In the past 6 months, have you or any of your partners decided to end a sexual relationship, either temporarily or permanently?  Kwa miezi 6 zilizopita,wewe au mpenzi wako ye yote ameamua kukomesha uhusiano wenu wa kimapenzi kwa muda tu au kabisa ? | O YES  O NO 🡪 skip to HST15  O Don’t know 🡪 skip to HST15  O Refused to answer 🡪 skip to HST15 |
| HST12 | In the past 6 months, with how many partners was the relationship ended?  Kwa miezi 6 zilizopita ni kwa wapenzi wangapi ulikomesha uhusiano nao ? | ___ ___ (cannot equal 00)  O Don’t Know  O Refused to Answer |
| HST13 | Who decided to end the relationship(s)?    Nani aliamua kukomesha uhusiano huo? | O Participant  O Partner  O Don’t Know  O Refused to Answer |
| HST14 | Why did you or your partner decide to end the relationship(s)?  Kwa nini wewe au mpezi wako mliamua kutamatisha uhusiano wenu?  *Choose all that apply.* | O He refused to use an HIV self-test  O He refused to go to VCT  O He tested HIV-positive  O Physical abuse  O Verbal abuse  O Financial support ended  O Relocation of the partner or the participant  O Non-HIV related relationship problems (outside partners, trust issues, new marriage, etc.)  O Other (specify)  O Don’t Know  O Refused to Answer |
| HST15 | In the past 6 months, have there been any partners with whom you declined to have sex with after they refused to accept a self-test or tested HIV-positive?  Kwa miezi 6 iliyopita, kumewahi kuwa na washiriki wowote ambao ulikataa kushiriki nao ngono baada yao kukataa kukubali kifaa cha kujipima kibinafsi au kupatikana na VVU? | O YES  O NO 🡪 skip to HST17  O Don’t know 🡪 skip to HST17  O Refused to answer 🡪 skip to HST17 |
| HST16 | In the past 6 months, how many partners did you decline to have sex with after they refused to accept a self-test or tested HIV-positive?  Kwa miezi 6 zilizopita, ni wapenzi wangapi uliyo kataa kushiriki nao ngono baada yao kukataa kukubali kujipima kibinafsi au walipopatikana na VVU? | ___ ___  O Don’t Know  O Refused to Answer |
| HST17 | In the past 6 months, have there been any partners with whom you decided to use a condom when having sex after they refused to accept a self-test or tested HIV-positive?  Kwa miezi 6 zilizopita, kumewahi kuwa na wapenzi wowote ambao uliamua kutumia mpira wa kondomu nao unaposhiriki ngono baada yao kukataa kukubali kifaa cha kujipima kibinafsi cha VVU au walipopatikana na VVU? | O YES  O NO 🡪 skip to NOTE  O Don’t know 🡪 skip to NOTE  O Refused to answer 🡪 skip to NOTE |
| HST18 | In the past 6 months, how many partners did you decide to use a condom with after they refused to accept a self-test or tested HIV-positive?  Kwa miezi 6 zilizopita, ni washiriki wangapi uliamua kutumia mpira wa kondomu nao baada yao kukataa kutumia mpira wa kondomu nao baada yao kukataa kukubali kujipima kibinafsi au kupatikana na VVU? | ___ ___  O Don’t Know  O Refused to Answer |

| **Section G2: VCT Referral Cards (Control Group Only)** | | |
| --- | --- | --- |
| *Read to participants in intervention group:* “I would like to ask you about your experience with the VCT referral cards that you received as part of Jikinge in the past 6 months.”  “Ningependa kukuuliza juu ya uzoefu wako na kupatiana kadi za mwaliko ulizozipata kama sehemu ya Jikinge kwa miezi 6 zilizopita.” | | |
| **NO.** | **QUESTIONS** | **CODING CATEGORIES** |
| VCT01 | Please tell me how many VCT Referral Cards you have received **in the past 6 months**?  Tafadhali niambie ni kadi ngapi za mwalikoo umepokea kwa **miezi 6 iliyopita?**  *Interviewer*: *If the woman says “Don’t know” please probe to see if she remembers receiving the tests, and if so, if she can make a guess about how many she received.* | ___ ___  O Don’t know/recall  O Refused to answer |
| VCT02 | Please tell me how many VCT Referral Cards you have received **in the past month**?  Tafadhali niambie ni kadingapi za rufaa za kuenda kwa kituo cha VCT ka **mwezi mmoja iliyopita**?  *Interviewer*: *If the woman says “Don’t know” please probe to see if she remembers receiving the tests, and if so, if she can make a guess about how many she received.* | ___ ___  O Don’t know/recall  O Refused to answer |
| VCT03 | In the past 6 months, how many of HIV VCT Referral Cards have you used yourself to go test at a VCT clinic?  Kwa miezi 6 zilizopita, ni kadi napi za mwaliko za kupima VVU umetumia kibinafsi kwenda kujipima katika kituo (kliniki)cha kupima VVU ? | ___ ___  O Don’t know  O Refused to answer |
| VCT04 | In the past 6 months, have you given any VCT Referral Cards to somebody else, such as your husband, sexual partners, friends, or family members?  Kwa miezi 6 zilizopita , umewahi pattiana kadi ya mwaliko kwa mtu mwingine , kama vile mumeo, washiriki wa ngono, marafiki, , au watu wa familia?  *Interviewer*: *Probe and include friends, co-workers etc. Make sure to ask participant to remember* ***all*** *VCT Referral Cards that she gave to somebody else (even if that person did not end up using it)* | O YES  O NO 🡪 skip to VCT11  O Don’t know 🡪 skip to VCT11  O Refused to answer 🡪 skip to VCT11 |
| VCT05 | In the past 6 months, about how many VCT Referral Cards did you give to a sexual partner?  Kwa miezi 6 zilizopita, ni kama kadi za mwaliko ngapi ulizopatiana kwa mshiriki wa ngono?  *Interviewer: Remind participant that if she gave 2 cards to the same partner, that should count as 2 cards given.* | ___ ___  O Don’t know  O Refused to answer |
| VCT06 | In the past 6 months, please tell me all the types of sexual partners whom you gave VCT Referral Cards to.  Kwa miezi 6 zilizopita, tafadhali nieleze aina zote za washiriki wa ngono wale uliowapatia za kwenda katika kituo cha kupima VVU.  *Interviewer – select all that apply* | O Primary sexual partner  O Sexual partner who is not your primary sexual partner  O Commercial sex partner (client or transactional sex partner)  O Don’t know  O Refused to answer |
| VCT07 | Of all the sexual partners whom you gave VCT Referral Cards to in the past 6 months, how many received an HIV-positive result at the VCT clinic (either directly observed by you or otherwise)?  Kwa miezi 6 zilizopita, tafadhali nieleze aina zote za washiriki wa ngono wale uliowapatia za kwenda katika kituo cha kupima VVU. | ___ ___  O Don’t know  O Refused to answer |
| VCT08 | In the past 6 months, about how many VCT Referral Cards did you give to other individuals who are not your sexual partners (e.g. family and friends)?  Kwa miezi 6 zilizopita, ni kama kadi ngapi za mwaliko za kwenda katika kituo cha kupima VVU ulipatiana kwa watu wengine ambao si washiriki wako wa ngono?(kwa mfano jamii na marafiki) | ___ ___ (if 00, skip to VCT11)  O Don’t know  O Refused to answer |
| VCT09 | In the past 6 months, please tell me all the types of other individuals who you gave VCT Referral Cards to.  Kwa miezi 6 zilizopita, tafadhali niambie aina zote za washiriki wengine wa ngono wale uliwapa kadi za mwaliko za kwenda kujipima katika kituo cha kupima VVU?  *Interviewer – select all that apply* | O A male adult family member  O A female adult family member  O A child in your family  O A male friend  O A female friend  O Coworker or Peer  O Don’t know  O Refused to answer |
| VCT10 | Of all the other individuals whom you gave VCT Referral Cards to in the past 6 months, how many received an HIV-positive result (either directly observed by you or otherwise)?  Kwa watu wote wale uliowapatia kadi za mwaliko za kwenda katika kituo cha kupima VVU kwa miezi 6 zilizopita, ni wangapi walipokea matokeo ya kuwa na VVU (kwa njia ya moja kwa moja kama unaona au kwa vyengine vile)? | ___ ___  O Don’t know  O Refused to answer |
| VCT11 | In the past 6 months, did any of your sexual partners refuse to accept VCT referral cards from you?  Kwa miezi 6 zilizopita,je mshiriki wako wa ngono yeyote alikataa kukubali kifaa cha kujipima kibinafsi kutoka kwako? | O YES  O NO  O Don’t know  O Refused to answer |
| VCT12 | In the past 6 months, have you received or used oral HIV self-tests kits?  Kwa miezi 6 zilizopita, umewahi pokea au kutumia vifaa vya kujipima kibinafsi vya VVU? | O YES  O NO 🡪 Skip to VCT14  O Don’t know 🡪 skip to VCT14  O Refused to answer 🡪 skip to VCT14 |
| VCT13 | From which person or place did you receive the oral HIV self-test kits?  Ni kwa mtu yupi au mahali ulipokea vifaa vya kujipima kibinafsi vya VVU?  *Choose all that apply.* | O Friend or family member  O Purchased  O Clinic or hospital  O Another research study  O Other (specify): __________________ |
| **Relationship and sexual behavior change** | | |
| VCT14 | In the past 6 months, have you or any of your partners decided to end a sexual relationship, either temporarily or permanently?  Kwa miezi 6 zilizopita, je wewe au mshiriki wako yeyote mmeamua kutamatisha uhusiano wa ngono kwa muda au kabisa? | O YES  O NO 🡪 skip to VCT18  O Don’t know 🡪 skip to VCT18  O Refused to answer 🡪 skip to VCT18 |
| VCT15 | In the past 6 months, with how many partners was the relationship ended?  Kwa miezi 6 zilizopita, uhusiano ulitamatishwa na washiriki wangapi? | ___ ___ (cannot equal 00)  O Don’t Know  O Refused to Answer |
| VCT16 | Who decided to end the relationship(s)?  Ni nani aliamua kutamatisha uhusiano? | O Participant  O Partner  O Don’t Know  O Refused to Answer |
| VCT17 | Why did you or your partner decide to end the relationship(s)?  Kwa nini wewe au mpezi wako mliamua kutamatisha uhusiano wenu? | O He refused to use an HIV self-test  O He refused to go to VCT  O He tested HIV-positive  O Physical abuse  O Verbal abuse  O Financial support ended  O Relocation of the partner or the participant  O Non-HIV related relationship problems (outside partners, trust issues, new marriage, etc.)  O Other (specify)  O Don’t Know  O Refused to Answer |
| VCT18 | In the past 6 months, have there been any partners with whom you declined to have sex with after they refused to accept a VCT referral card or after they tested HIV-positive?  Kwa miezi 6 iliyopita, kumewahi kuwa na washiriki wowote ambao ulikataa kushiriki nao ngono baada yao kukataa kukubali kadi ya mwaliko ya kujipima VVU katika ktuo cha kupima VVU au baada yao kupatikana na VVU? | O YES  O NO🡪 skip to VCT20  O Don’t know 🡪 skip to VCT20  O Refused to answer 🡪skip to VCT20 |
| VCT19 | In the past 6 months, how many partners did you decline to have sex with after they refused to accept a VCT referral card or after they tested HIV-positive?  Kwa miezi 6 zilizopita, ni washiriki wangapi ulikataa kushiriki nao ngono baada ya kukataa kukubali kadi ya mwaliko ya kupima VVU au baada yao kupatikana na VVU? | ___ ___  O Don’t Know  O Refused to Answer |
| VCT20 | In the past 6 months, have there been any partners with whom you decided to use a condom when having sex after they refused to accept a VCT referral card or after they tested HIV-positive?  Kwa miezi 6 zilizopita, kumewahi kuwa na washiriki wowote ambao uliamua kushiriki ngono nao ukitumia mpira wa kondomu baada yao kukataa kukubali kadi ya mwaliko ya kupima VVU katika kituo cha kupima VVU au baada yao kupatikana na VVU? | O YES  O NO 🡪skip to NOTE  O Don’t know 🡪 skip to NOTE  O Refused to answer 🡪 skip to NOTE |
| VCT21 | In the past 6 months, how many partners did you decide to use a condom with after they refused to accept a VCT referral card or after they tested HIV positive?  Kwa miezi sita zilizopita,ni washiriki wangapi uliamua kutumia nao kondomu walipokataa kukubali kadi ya mwaliko ya kupima VVu katika kituo cha kupima VVU au baada yao kupatikana na VVU? | ___ ___  O Don’t Know  O Refused to Answer |

| **Section H: Interviewer’s Observations (NOTE)**  *Interviewer: Complete at the end of the interview* |
| --- |
|  |

| **Section I: HIV Rapid Test and DBS Collection**  Perform HIV antibody rapid testing and enter results. If positive, alert Study Coordinator to pull DBS card collected at Baseline for confirmatory testing. | | |
| --- | --- | --- |
| hiv | HIV rapid test result | O Negative  O Positive  O Indeterminate  O Woman refused testing |

**END OF FOLLOW-UP QUESTIONNAIRE**
